# Supplementary material for: The genetic legacy of the expansion of Bantu-speaking peoples in Africa
Source: Nature. 2023 Nov 29;625(7995):540–7. doi: 10.1038/s41586-023-06770-6 (PMC10794141; doi:10.1038/s41586-023-06770-6)
Supplement: Supplementary file 1 — Supplementary Methods, Notes 1–12, Figures 1–107, and references. [file 41586_2023_6770_MOESM1_ESM.pdf]

nature portfolio

https://doi.org/10.1038/s41586-023-06770-6

Supplementary information

The genetic legacy of the expansion of Bantu-speaking peoples in Africa

In the format provided by the authors and unedited

Supplementary Information for

The genetic legacy of the expansion of Bantu-speaking peoples in Africa

Cesar A. Fortes-Lima[1,27], Concetta Burgarella[1,27], Rickard Hammarén[1,27], Anders Eriksson[2], Mário Vicente[3,4], Cecile Jolly[1], Armando Semo[5,6,7], Hilde Gunnink[8,9], Sara Pacchiarotti[8], Leon Mundeke[10], Igor Matonda[10], Joseph Koni Muluwa[11], Peter Coutros[8], Terry S. Nyambe[12], Cirhuza Cikomola[13], Vinet Coetzee[14], Minique de Castro[15], Peter Ebbesen[16], Joris Delanghe[17], Mark Stoneking[18,19], Lawrence Barham[20], Marlize Lombard[21], Anja Meyer[22], Maryna Steyn[22], Helena Malmström[1,21], Jorge Rocha[5,6,7], Himla Soodyall[23,24], Brigitte Pakendorf[25], Koen Bostoen[8] & Carina M. Schlebusch[1,21,26]

[1] Human Evolution Program, Department of Organismal Biology, Uppsala University, Uppsala, Sweden. [2] cGEM, Institute of Genomics, University of Tartu, Tartu, Estonia. [3] Centre for Palaeogenetics, University of Stockholm, Stockholm, Sweden. [4] Department of Archaeology and Classical Studies, Stockholm University, Stockholm, Sweden. [5] CIBIO, Centro de Investigação em Biodiversidade e Recursos Genéticos, Universidade do Porto, Vairão, Portugal. [6] BIOPOLIS Program in Genomics, Biodiversity and Land Planning, CIBIO, Campus de Vairão, Vairão, Portugal. [7] Departamento de Biologia, Faculdade de Ciências, Universidade do Porto, Porto, Portugal. [8] UGent Centre for Bantu Studies (BantUGent), Department of Languages and Cultures, Ghent University, Ghent, Belgium. [9] Leiden University Centre for Linguistics, Leiden, The Netherlands. [10] University of Kinshasa, Kinshasa, Democratic Republic of Congo (DRC). [11] Institut Supérieur Pédagogique de Kikwit, Kikwit, DRC. [12] Livingstone Museum, Livingstone, Zambia. [13] Faculty of Medicine, Catholic University of Bukavu, Bukavu, DRC. [14] Department of Biochemistry, Genetics and Microbiology, University of Pretoria, Pretoria, South Africa. [15] Biotechnology Platform, Agricultural Research Council, Onderstepoort, Pretoria, South Africa. [16] Department of Health Science and Technology, University of Aalborg, Aalborg, Denmark. [17] Department of Diagnostic Sciences, Ghent University, Ghent, Belgium. [18] Department of Evolutionary Genetics, Max Planck Institute for Evolutionary Anthropology, Leipzig, Germany. [19] Université Lyon 1, CNRS, Laboratoire de Biométrie et Biologie Evolutive, UMR 5558, Villeurbanne, France. [20] Department of Archaeology, Classics & Egyptology, University of Liverpool, Liverpool, UK. [21] Palaeo-Research Institute, University of Johannesburg, Johannesburg, South Africa. [22] Human Variation and Identification Research Unit, School of Anatomical Sciences, Faculty of Health Sciences, University of the Witwatersrand, Johannesburg, South Africa. [23] Division of Human Genetics, School of Pathology, Faculty of Health Sciences, University of the Witwatersrand, Johannesburg, South Africa. [24] Academy of Science of South Africa, Pretoria, South Africa. [25] Research unit «Dynamique Du Langage», UMR5596, CNRS & Université de Lyon, Lyon, France. [26] SciLifeLab, Uppsala, Sweden. [27] These authors contributed equally to this work: Cesar A. Fortes-Lima, Concetta Burgarella, and Rickard Hammarén.

Table of contents 1
1. Supplementary Methods 5
1.1. Ethics approval 5
1.2. Genotyping procedure 5
1.3. Ancient DNA samples from sub-Saharan Africa 7
1.3.1. Description of human remains 7
1.3.2. Sampling procedure 7
1.3.3. DNA Extraction from human remains 8
1.3.4. Library building and sequencing 8
1.3.5. Sequence data processing 8
1.4. Assembling genome-wide genotype datasets 9
1.5. Population structure analyses 10
1.6. Unsupervised clustering analyses 10

1.7. Testing analysis of f3- or f4-statistics 11
1.8. Local ancestry deconvolution approach 11
1.9. Runs of homozygosity and inbreeding coefficients 12
1.10. Detection of shared segments inherited from a common ancestor 13
1.11. Estimating the timing of and strength of founder events 13
1.12. Haplotype diversity and linkage disequilibrium analyses 14
1.13. Phylogenetic analyses 14
1.14. Testing models of isolation-by-distance 15
1.15. Testing spatially explicit models of the BSP expansion 15
1.16. Estimating effective migration rates 16
1.17. Gene-flow barriers analysis on a grid 16
1.18. Admixture timing inference 17
1.19. Correlations between linguistic, geographical, and genetic data 17
1.20. Comparisons between ancient and present-day populations 17
2. Supplementary Notes 18
Supplementary Note 1. Further background on the BSP expansion 18
Supplementary Note 2. Patterns of population structure and genetic diversity 19
Supplementary Note 3. Ancestry-specific analyses in BSP 21
Supplementary Note 4. Patterns of consanguinity and founder events 21
Supplementary Note 5. Effective population size and founder events in BSP 22
Supplementary Note 6. Patterns of isolation-by-distance among BSP 23
Supplementary Note 7. Patterns of haplotype diversity and LD among BSP 23
Supplementary Note 8. Phylogenetic analyses of BSP 25
Supplementary Note 9. Patterns of pairwise genetic distances and admixture graphs 26
Supplementary Note 10. Estimation of effective migration surfaces 26
Supplementary Note 11. Admixture dating and model-testing of the BSP expansion 27
Supplementary Note 12. Comparisons between ancient and present-day populations 28
3. Supplementary Figures 31
3.1. Extended introduction and geographical locations of studied populations 31
Supplementary Fig. 1 | Linguistic hypotheses proposed to explain the expansion of BSP. 31
Supplementary Fig. 2 | Geographical locations of African and Eurasian populations. 32
Supplementary Fig. 3 | All the groups and populations included in the AfricanNeo dataset. 34
Supplementary Fig. 4 | All the populations included in the Only-Africa and Only-BSP datasets. 35
3.2. Dimensionality reduction methods (DRM) 37
Supplementary Fig. 5 | Workflow for the assembled datasets. 37
Supplementary Fig. 6 | UMAP approach applied on the basis of genotype data. 38
Supplementary Fig. 7 | PCA plots for only Bantu-speaking populations. 39
Supplementary Fig. 8 | PCA plots for selected sub-Saharan African populations. 40
Supplementary Fig. 9 | Procrustes rotated PCA for the Only-African dataset. 42
Supplementary Fig. 10 | PCA for all available sub-Saharan African populations. 43
Supplementary Fig. 11 | PCA for each group included in the AfricanNeo dataset. 44
Supplementary Fig. 12 | PCA-UMAP approach applied for the AfricanNeo dataset. 45
Supplementary Fig. 13 | GCAE approach for the AfricanNeo and Only-African datasets. 46
3.3. Unsupervised clustering analyses 47
Supplementary Fig. 14 | Contour map of ADMIXTURE at K=12. 47
Supplementary Fig. 15 | Bar plots of ADMIXTURE results for K=12. 48
Supplementary Fig. 16 | Cross-validation test from the ADMIXTURE analyses for each K-group. 49
Supplementary Fig. 17 | Pie charts of ADMIXTURE results for K=2. 50
Supplementary Fig. 18 | Pie charts of ADMIXTURE results for K=4. 51

Supplementary Fig. 19 | Pie charts of ADMIXTURE results for K=6. 52
Supplementary Fig. 20 | Pie charts of ADMIXTURE results for K=12. 53
Supplementary Fig. 21 | Pie charts of ADMIXTURE results for K=16. 54
Supplementary Fig. 22 | Pie charts of ADMIXTURE results for only studied BSP. 55
Supplementary Fig. 23 | Pie charts of ADMIXTURE results for K=16 only for BSP. 56
Supplementary Fig. 24 | Ternary diagrams of ADMIXTURE results at K=6. 58
Supplementary Fig. 25 | Bar plots of ADMIXTURE results for K=4. 59
Supplementary Fig. 26 | Bar plots of ADMIXTURE results for K=16. 60
Supplementary Fig. 27 | Pie charts of ADMIXTURE results for K=16 only for DRC and Zambia. 61
3.4. F-statistics analyses 62
Supplementary Fig. 28 | F3- and f4-statistics tests used to estimate Afro-Asiatic admixture in BSP. 62
Supplementary Fig. 29 | F3- and f4-statistics tests used to estimate wRGH admixture in BSP. 63
Supplementary Fig. 30 | F3- and f4-statistics tests used to estimate Khoe-San admixture in BSP. 64
Supplementary Fig. 31 | Genetic affinity of BSP to non-BSP estimated using f4-statistics. 65
Supplementary Fig. 32 | F3- and f4-statistics tests used to estimate hunter-gatherer admixture in BSP. 66
3.5. Ancestry-specific analyses 67
Supplementary Fig. 33 | Comparisons between estimated admixture fractions67
Supplementary Fig. 34 | Ancestry-specific (AS-)PCA for the masked Only-BSP dataset. 68
3.6. Genome-wide runs of homozygosity (ROH) estimates 70
Supplementary Fig. 35 | Total sum of short ROH length for the AfricanNeo dataset. 70
Supplementary Fig. 36 | Total sum of long ROH length for the AfricanNeo dataset. 71
Supplementary Fig. 37 | Mean of long ROH for the AfricanNeo dataset. 72
Supplementary Fig. 38 | Total length of long ROH for the AfricanNeo dataset. 73
Supplementary Fig. 39 | Genomic inbreeding coefficient for the AfricanNeo dataset. 74
Supplementary Fig. 40 | All categories of ROH length for the AfricanNeo dataset. 75
Supplementary Fig. 41 | All categories of ROH length for the Only-BSP dataset. 76
Supplementary Fig. 42 | Violin plots for category 1 of ROH length. 77
Supplementary Fig. 43 | Violin plots for category 2 of ROH length. 78
Supplementary Fig. 44 | Violin plots for category 3 of ROH length. 79
Supplementary Fig. 45 | Violin plots for category 4 of ROH length. 80
Supplementary Fig. 46 | Violin plots for category 5 of ROH length. 81
Supplementary Fig. 47 | Violin plots for category 6 of ROH length. 82
Supplementary Fig. 48 | FROH estimates after masking the AfricanNeo dataset. 83
3.7. Estimated effective population sizes and demographic founder events 84
Supplementary Fig. 49 | IBDNe results for selected BSP included in the AfricanNeo dataset. 84
Supplementary Fig. 50 | IBDNe results for BSP from African regions. 86
Supplementary Fig. 51 | Ancestry-specific IBDNe results for selected BSP. 87
Supplementary Fig. 52 | Intensity of founder events in sub-Saharan African populations. 88
Supplementary Fig. 53 | Timing of founder ages in sub-Saharan African populations. 89
3.8. Patterns of isolation-by-distance for the unmasked and masked datasets 90
Supplementary Fig. 54 | SpaceMix for the unmasked dataset with no population text overlays.90
Supplementary Fig. 55 | SpaceMix for the unmasked dataset with text instead of dots. 91
Supplementary Fig. 56 | SpaceMix for the unmasked dataset with sources of admixture. 92
Supplementary Fig. 57 | SpaceMix for the unmasked dataset with correlations of each tested model. 93
Supplementary Fig. 58 | SpaceMix for the masked dataset with no population text overlays. 94
Supplementary Fig. 59 | SpaceMix for the masked dataset with text instead of dots. 95
Supplementary Fig. 60 | SpaceMix for the masked dataset with sources of admixture. 96
Supplementary Fig. 61 | SpaceMix for the masked dataset with correlations of each model. 97
3.9. Patterns of haplotype diversity 98

Supplementary Fig. 62 | Haplotype richness (HR) for the AfricanNeo dataset. 98
Supplementary Fig. 63 | Haplotype heterozygosity (HH) for the AfricanNeo dataset. 99
Supplementary Fig. 64 | Maps of haplotype diversity for the AfricanNeo data. 100
Supplementary Fig. 65 | Linkage-disequilibrium decay of the unmasked AfricanNeo dataset. 101
Supplementary Fig. 66 | Haplotype richness (HR) with masked AfricanNeo dataset. 102
Supplementary Fig. 67 | Haplotype heterozygosity (HH) with masked AfricanNeo dataset. 103
Supplementary Fig. 68 | Haplotype richness (HR) for the masked Only-BSP dataset. 104
Supplementary Fig. 69 | Haplotype heterozygosity (HH) for the masked Only-BSP. 105
Supplementary Fig. 70 | Maps of haplotype diversity for the Only-BSP dataset. 106
Supplementary Fig. 71 | Haplotype richness plotted against distance from Cameroon. 107
Supplementary Fig. 72 | Haplotype heterozygosity plotted against distance from Cameroon. 108
Supplementary Fig. 73 | Linkage-disequilibrium (LD)-decay of Only-BSP dataset. 109
Supplementary Fig. 74 | Spatial distribution of LD-decay in each studied dataset. 110
Supplementary Fig. 75 | Increase of LD patterns with geographical distances in studied BSP. 111
3.10. Maximum likelihood trees based on population allele frequencies 112
Supplementary Fig. 76 | Coancestry matrix for the masked and imputed Only-BSP dataset. 112
Supplementary Fig. 77 | TreeMix analysis for the unmasked AfricanNeo dataset. 113
Supplementary Fig. 78 | Coancestry matrix for the unmasked AfricanNeo dataset. 114
Supplementary Fig. 79 | TreeMix analysis for the unmasked Only-BSP dataset. 115
Supplementary Fig. 80 | Coancestry matrix for the unmasked Only-BSP dataset. 116
3.11. Visualizing migration routes in sub-Saharan Africa 117
Supplementary Fig. 81 | Demographic scenarios used in our spatially explicit framework 117
Supplementary Fig. 82 | FST matrix for the masked Only-BSP dataset. 118
Supplementary Fig. 83 | FST map for the masked Only-BSP dataset. 119
Supplementary Fig. 84 | EEMS for the unmasked Only-African dataset. 120
Supplementary Fig. 85 | EEMS for the masked Only-African dataset. 121
Supplementary Fig. 86 | EEMS for the unmasked Only-BSP dataset. 122
Supplementary Fig. 87 | EEMS on the basis of the masked Only-BSP dataset. 123
Supplementary Fig. 88 | FEEMS of the unmasked AfricanNeo dataset. 124
Supplementary Fig. 89 | FEEMS on the basis of the unmasked Only-BSP dataset. 125
Supplementary Fig. 90 | Spatial visualization of genetic barriers analysis on a grid. 126
Supplementary Fig. 91 | Dispersal surfaces based on shared IBD tracts. 127
3.12. Estimated admixture dates in BSP 128
Supplementary Fig. 92 | MOSAIC results for BSP with admixture. 128
Supplementary Fig. 93 | Admixture dates versus geographical distances from Cameroon. 129
3.13. Comparisons between aDNA individuals and modern-day African populations 130
Supplementary Fig. 94 | Nucleotide misincorporation patterns of 12 aDNA individuals. 130
Supplementary Fig. 95 | Geographical locations of aDNA individuals included in this study. 131
Supplementary Fig. 96 | PCA of aDNA and modern African populations. 132
Supplementary Fig. 97 | PCA-UMAP of aDNA individuals and present-day African populations. 134
Supplementary Fig. 98 | ADMIXTURE results at K=4 of ancient and modern African populations. 135
Supplementary Fig. 99 | Genetic affinity of UPS013, UPS017a and UPS029 to modern BSP. 136
Supplementary Fig. 100 | Genetic affinity of WUD034, WUD037 and WUD038b to modern BSP. 137
Supplementary Fig. 101 | PCA and PCA-UMAP of ancient individuals and BSP from South Africa. 138
Supplementary Fig. 102 | Genetic affinity of WUD003, WUD004 and WUD008 to modern BSP. 139

Supplementary Fig. 103 | Genetic affinity of WUD010, WUD012 and WUD018 to modern BSP. 140
Supplementary Fig. 104 | PCA of ancient individuals and BSP from present-day Zambia. 141
3.14. PCA results after masking datasets 142
Supplementary Fig. 105 | PCA of BSP and six selected reference panels before using masking. 142
Supplementary Fig. 106 | PCA of masked BSP and six unmasked selected reference panels. 143
Supplementary Fig. 107 | PCA of masked non-BSP and six unmasked selected reference panels. 145
4. Supplementary Information References 146

1. Supplementary Methods

1.1. Ethics approval

New samples presented in this study were collected in large-scale sampling campaigns conducted in fourteen sub-Saharan African countries: Angola (*n* = 34 individuals), Botswana (*n* = 56), Central African Republic (CAR; *n* = 81), the Democratic Republic of the Congo (DRC; *n* = 597), Lesotho (*n* = 8), Mozambique (*n* = 36), Namibia (*n* = 130), Rwanda (*n* = 25), South Africa (*n* = 389), Swaziland (present-day the Kingdom of Eswatini; *n* = 17), Tanzania (*n* = 38), Uganda (*n* = 71), Zambia (*n* = 241), and Zimbabwe (*n* = 40) (Supplementary Table 1). Each participant gave informed consent before donating their samples. Where information was available, only individuals whose parents and grandparents came from the same ethnolinguistic group were included in the study.
This study was conducted according to the Declaration of Helsinki [75]. This study as a whole was approved by the Swedish Ethical Review Authority (Ministry of Education, Sweden). Biological samples for this study were in part supplied by our collaborators who obtained the original ethical permission for the sampling in African countries. Himla Soodyall was granted ethics approval by the Human Research Ethics Committee (Medical) (University of the Witwatersrand, South Africa; protocol Nr. M180656). Brigitte Pakendorf was granted ethics approval by the Biomedical Research Ethics Board (University of Zambia, Zambia; protocol number: 004-08-07). Vinet Coetzee was granted ethics approval by the Faculty of Natural and Agricultural Sciences Ethics Committee (University of Pretoria, South Africa; protocol number: EC160429-024 and 259/2016). Koen Bostoen was granted ethics approval by the Swedish National Ethics Committee (Sweden; protocol number: Dnr 2019-05244), as well as permission for sampling in DRC from the Minister of Arts and Culture (DRC; protocol number: Nr 091/CAB/MIN/CA/PKB/2018). For the analyses of the ancient individuals, Maryna Steyn received clearance from the Raymond A. Dart Archeological Human Remains Collection (12/10/2017) and obtained permits to sample and export specimens from the South African Heritage Resources Agency (SAHRA).

1.2. Genotyping procedure

For this study, we genotyped DNA samples of 1,763 individuals encompassing 163 African populations (Extended Data Fig. 1, Supplementary Fig.s 2–5, and Supplementary Table 1), including 1,526 Bantu-speaking individuals from 147 African populations spanning 14 sub-Saharan African countries, 8 Khoe-San populations from four African countries (in Angola: Khwe (*n* = 17 individuals) and Xun (*n* = 2); in Botswana: GuiGhanaKgal (*n* = 11); in Namibia: Damara (*n* = 23), Nama (*n* = 16), and Ju'hoansi (*n* = 11; population label: “Namibia_TsumkweKung”); in

SouthAfrica: Karretjie (*n* = 10) and Khomani (*n* = 32); 122 Khoe-San-speaking individuals in total), five Ubangi-speaking populations from CAR (Banda (*n* = 10), DzangaShanga people (*n* = 22), Gbaya (*n* = 24), Nzakara (*n* = 8), and Zande (*n* = 6); 70 Ubangi-speaking individuals in total), and South African Coloured individuals (*n* = 45). The DNA samples of the first seven batches were genotyped on the Illumina Infinium H3Africa Consortium array, designed by the H3Africa Consortium [76]. This genotyping array was specially designed for SNP-genotyping of 2,271,503 SNPs, to account for the larger genetic diversity and smaller haplotype segments in African populations [76]. Seven of the Khoe-San groups and the Coloured individuals were previously included in Schlebusch et al. [12], where they were typed on the Illumina Human Omni 2.5M BeadChip. Here the same individuals were re-genotyped on the Illumina Infinium H3Africa Consortium array to increase overlap with the current study as well as to include additional individuals from the same populations.

Genotyping was performed at the SNP&SEQ Technology Platform, NGI/SciLifeLab Genomics (Sweden), and the results were analyzed using the software GenomeStudio (v2.0.3, Illumina Inc). Genotype data were exported from the forward strand according to polymorphism data from dbSNP v131 [77] and using the human reference genome build version 37 (or hg19).

Samples were genotyped in seven batches, as follows:

- The first batch (called "SE-2209_191110") included 2,267,346 variants and 1,157 individuals, and was generated for this study using BeadChip type: H3Africa_2017_20021485_A2. The average SNP call rate per sample was 97.11% (range: 21.58%-99.64%), and the reproducibility was 99.95% (6,201 conflicts in 13,547,660 duplicate tests). Fifty-one samples were identified as being above our threshold of 80% similarity in genotype data in the test.

- The second batch (called "TE-2567_201023_2017") included 2,267,346 variants and 4 individuals for this study and was generated using BeadChip type: H3Africa_2017_20021485_A2. The average SNP call rate per sample was 95.46% (range: 18.10-99.60%), and no samples were not identified as being above the threshold of 80% in this test.

- The third batch (called "TE-2567_201023_2019") included 2,271,503 variants and 298 individuals for this study and was generated using BeadChip type: H3Africa_2019_20037295_B1. The average SNP call rate per sample was 99.11% (range: 53.49-99.52%), and the reproducibility was 99.98% (1,796 conflicts in 9,023,008 duplicate tests). Two pairs of samples were identified as being above the threshold of 80% similarity (between 069KKT and 030KKT; and between 026KKT and 073KKT).

- The fourth batch (called "TI-2658_201112") included 2,271,503 variants and 135 individuals and was generated for this study using BeadChip type: H3Africa_2019_20037295_B1. The average SNP call rate per sample was 97.74% (range: 34.32%-99.48%). Two pairs of samples were identified as being above the threshold of 80% similarity (LD162 and LD132; SK013 and SK021).

- The fifth batch (called "RK-2011_190308") included 2,267,346 variants and 46 individuals for this study and was generated using BeadChip type: H3Africa_2017_20021485_A2. The average SNP call rate per sample was 99.31% (range: 93.46%-99.61%), and the reproducibility was 99.99% (911 conflicts in 6,710,668 duplicate tests). No pair of samples was identified as being above the threshold of 80%.

- The sixth and seventh batches (called “TC-2508_200401_A” and “TC-2508_200401_B”, respectively) were genotyped at the University of Pretoria (South Africa), and both batches included 2,267,346 variants and 100 individuals in total for this study, 41 individuals genotyped in the sixth batch and 58 genotyped in the seventh batch.

- The eighth batch included 29 individuals from Ghanzi in Botswana (called "OE-0808_150625"), genotyped using Illumina HumanOmni2.5-Octo BeadChip. Two individuals were removed due to kinship.

1.3. Ancient DNA samples from sub-Saharan Africa

To compare the genetic diversity of ancient and present-day BSP, we merged the Only-African dataset with 95 ancient DNA (aDNA) individuals (Supplementary Fig. 95). Among them, 12 individuals were newly sequenced for this study (Supplementary Table 14) and 83 individuals were retrieved from previous studies [23,57–62] (Supplementary Table 13).

1.3.1. Description of human remains

The 12 new ancient human remains in this study came from various caves and rock shelters in Zambia and South Africa (Supplementary Table 14). We obtained permission from the South African Heritage Resources Agency (SAHRA) to sample and export bones for ancient DNA analyses. Nine WUD samples (permit number: 2789) are from the Raymond A. Dart Archaeological Human Remains Collection (Dart Collection) located at the School of Anatomical Sciences, University of the Witwatersrand (Johannesburg, South Africa). Three UPS samples (permit number: 2804) are from the Archeological Human Remains Collection (Pretoria Bone Collection) situated within the Department of Anatomy, University of Pretoria (Pretoria, South Africa). For both collections, Prof. Maryna Steyn is the permit holder. The archeological context, morphological assessments, and dating of the remains were described before for six of the samples: WUD034 and WUD037 (C1 and C9 in Meyer et al. [48]); and WUD003, WUD004, WUD008, and WUD010 [8]. WUD038b sample originated from an archeological site in KwaZulu-Natal but is curated in the Dart Collection. WUD012 (Chipongwe Caves) was collected in 1930 by Raymond Dart [78]. WUD012 and WUD018 were originally collected in current-day Zambia and are curated in the Dart Collection, while UPS013, UPS017a, and UPS029 are kept in the Pretoria Bone Collection. Little is known about their archeological contexts.

Six samples (WUD038b, WUD012, WUD018, UPS013, UPS017a, and UPS029) were accelerator mass spectrometry (AMS) radiocarbon dated at the Tandem Laboratory (Department of Physics and Astronomy, Uppsala University, Sweden). Radiocarbon dates were calibrated with OxCal 4.4 [79] using the atmospheric curve SHCal20 [80] and are given at 95.4% probability (2σ), see Supplementary Table 14.

1.3.2. Sampling procedure

The clean sampling of the bones was done on-site in South Africa and only the bone samples were transported to Uppsala University (Sweden). Following recommendations proposed by Schlebusch et al. [57], we used a bleach-decontaminated (RNase AWAY, ThermoScientific) enclosed sampling tent with adherent gloves (Captair Pyramid portable isolation enclosure (Erlab)). Prior to sampling and DNA extraction, the bones were cleaned to prevent contamination of the samples with modern DNA. First, the samples were ultraviolet irradiated (254 nm) for 20 minutes on each side, then the outer surface of the bones was removed by gentle scraping at low speed using a Dremel 8100 and wiped with 0.5% bleach (NaOH) and sterile water (HPLC grade, Sigma-Aldrich). The cleaned bones were ultraviolet irradiated (254 nm) again for 20 minutes per side. A piece of each bone, of between 50 and 80 mg, was cut off for aDNA analysis, using either a circular diamond cutting wheel for the tooth roots or a core drill for the petrous portion of temporal bones. Whenever possible, at least two bones from the same individual were sampled (Supplementary Table 14).

1.3.3. DNA Extraction from human remains

To increase the amount of endogenous aDNA, we extracted DNA from the sampled bone/tooth root pieces instead of extracting DNA from powdered bone [81]. The bone samples were pre-digested in 1 mL EDTA (0.5M, pH 8) for 30 min at 37°C, the EDTA was then discarded and an overnight digestion in 1 ml of EDTA (0.5M, pH 8.0) and 25 µl proteinase K (10 mg/ml) at 37°C were conducted. Then, 10.4ml of Binding buffer (245 ml of PB buffer from Qiagen, 7.5 ml of sodium acetate, 3M, and 0.625 ml of sodium chloride, 5M) were added to the DNA digests, and the DNA molecules were purified using a silica-columns method and eluted in 110 µl of EB buffer, following a protocol as in Dabney et al. [82] with modifications as in Rohland et al. [83]. Two extraction negative controls were processed for every 10 samples extracted.

1.3.4. Library building and sequencing

Double-indexed blunt-end DNA libraries were prepared from 20 µl of extract using P5 and P7 adapters as in Meyer and Kircher [84], with the shearing step omitted. Two library blanks were processed for every 12 samples extracted (including the extraction blanks). The optimal number of PCR cycles used for library amplification was determined using quantitative PCR (qPCR). The 25 µl qPCR reactions were set up in duplicates and contained 1 µl of DNA library, 1X Maxima SYBR Green Mastermix and 200 nM of each IS7 and IS8 primers (10 uM). To calculate the number of cycles for PCR amplification, two cycles were added to the qPCR Cq value (value at which the qPCR amplification was saturated and reached a plateau). Each library was then amplified using between 12 and 21 PCR cycles. The PCR reactions were set up in duplicates using 6µl of library DNA, Index primers P7 and P5 (10 uM) and 1µl of AmpliTaq Gold™ polymerase (Thermofisher Scientific). One negative PCR control was set up for every four reactions. The duplicate reactions were amplified according to the supplier recommendations (ThermoFisher Scientific) with an annealing temperature of 60°C. The amplified duplicates were first pooled and then purified using AMPure XP Beads (Agencourt). The resulting libraries were quantified on a TapeStation using a High Sensitivity kit (Agilent Technologies). Library layouts were paired-end, and the library construction protocol used blunt-end with dual indexing libraries for Illumina sequencing. Equimolar pools of 25-30 libraries were prepared for WGS at the Swedish National Genomics Infrastructure (NGI)-SciLifeLab in Uppsala (Sweden) on Illumina NovaSeq 6000 SP lane (one pool per lane), with a paired-end 150 bp chemistry. The negative controls did not yield any DNA and were therefore not sequenced.

1.3.5. Sequence data processing

The initial data processing of the shotgun data was as follows. Adapters were trimmed with cutadapt v. 2.3 [85] and the pair-end reads of each library were merged if the two reads overlapped at least 11 base pairs using FLASH v. 1.2.11 [86]. Bwa aln 0.7.13 [87] was then used to map them as single-end reads to the human reference genome (hg19). Non-default parameters for bwa were "-l 16500 -n 0.01 -o 2" [50,88]. Reads with identical start and end positions were identified as PCR duplicates and collapsed using a modified version of 'FilterUniqSAMCons_cc.py' [89], which ensures the random assignment of bases in a 50/50 case. Reads with less than 10% mismatches to the human reference genome and longer than 35 base pairs were retained for further analysis. Reads were authenticated by looking at read length distribution and damage patterns (Supplementary Fig. 94).

Sample contamination estimates were performed with two methods. We first ran the likelihood-based method ContamMix [90], which uses phylogenetically informative sites on the mitochondrial genome to estimate the proportion of authentic DNA ("clean" DNA). Briefly, the consensus sequence obtained from each ancient sample is aligned with 311 reference mt genomes [91] and

then each read is tested against all these 312 sequences. If reads map better to one of the 311 reference mt sequences, they might be caused by contamination. Second, we run verifyBAMid [92], which checks for autosomal contamination. The method checks if the target reads match a set of known reference genotypes and if they constitute a mixture of two samples. We used the 1000 Genomes Project Phase 3 data as potential reference contaminants. Results are shown in Supplementary Table 14.
To determine biological sex, we implemented the sex determination method previously described [93,94]. It uses reads with a mapping quality of at least 30 and calculates the ratio of reads mapping to the Y-chromosome and those reads mapping to both X- and Y-chromosomes. Strict mitochondrial consensus sequences were generated with SAMtools v1.5 [95] options 'mpileup' and 'vcfutils.pl'. Base and mapping quality scores were set to a minimum of 30 and only SNPs with at least 3-fold coverage were used. Mitochondrial haplogroups were assigned with HaploGrep v2.1.16 [96] using the mitochondrial tree built by PhyloTree Build 17 [97]. To account for low coverage data, the SNPs in the ancient individuals were called as follows: at each SNP site, a random read with minimum mapping and base quality 30 was drawn and the allelic status at that read was coded to be the hemizygous genotype of the individual (file-formats require diploid genotypes and we use the homozygote code for the record, but the data are treated as hemizygote in all downstream analyses). Sites showing additional alleles, indels or missing data were removed as well as transitions sites with T or A.

1.4. Assembling genome-wide genotype datasets

For autosomal data, quality control (QC) steps were performed using PLINK v1.90b6.4 [51], to keep autosomal biallelic variants with a high genotyping rate (>90%). We used the same filtering before and after merging for each dataset (as follows: "plink --mind 0.15 --geno 0.1 --hwe 0.0000001"), and samples were removed due to their low genotyping rate (mind> 0.15). To estimate recent genetic relatedness, we used KING v1.4 [98] and the PC-Relate tool included in the R GENESIS package [99], and we removed 67 samples due to their low genotyping rate, and 106 individuals were removed due to first- or second-degree kinship. After QC steps, the genotyped dataset consists of 2,221,827 autosomal SNPs.
To merge the newly genotyped dataset with publicly available datasets, we first collected data from datasets of whole-genome sequencing studies [100], and datasets previously genotyped on Illumina HumanOmni5, Illumina HumanOmni2.5 or Illumina H3Africa Consortium arrays. Then, genome-wide SNP data were merged with reference populations presented in previous studies of our group [12,15,101] or by other previous studies [13,34,102–108] (details and accessory numbers are included in Supplementary Table 2). Authorized NIH Data Access Committees (DAC) granted data access to C.M.S. for the controlled-access genetic data analyzed in this study for the Hadza and Sabue populations that were previously deposited by Crawford et al. [104] in the NIH dbGAP repository (accession code phs001396.v1.p1; project ID: 19895; date of approval: 2018-12-18). After merging all newly genotyped data and quality control (QC) steps, we assembled the full dataset that contains 482,459 SNPs and 5,341 individuals from 227 populations, and two sub-datasets with selected populations (Supplementary Fig. 5). Due to the small sample size in some populations, BSP with less than 10 individuals were removed from the dataset, and we obtained 4,950 individuals from 124 African and Eurasian populations in the "AfricanNeo" dataset (Supplementary Table 2 and Supplementary Fig.s 3); 3,902 individuals in the "Only-African" dataset for only 111 sub-Saharan African populations included in the AfricanNeo dataset (Supplementary Fig.s 4a,c); and 2,108 individuals in the "Only-BSP" dataset for 67 BSP included in the AfricanNeo dataset (Supplementary Fig.s 4b,d). We also used PLINK to prune SNPs under

high LD (plink "--indep-pairwise 100 10 0.2") for analyses that assume unlinked variation. After LD-pruning, the AfricanNeo LD-pruned dataset contains 223,473 variants and 268 individuals.
Of note, in our plots the Damara population from Namibia is included as BSP although they speak a Khoe-San language (hereafter "Damara-KSP"), while the Baka population from Cameroon and Gabon is included in the group wRHG although they speak Bantu languages. This is because these groups have genetic backgrounds that distinguish them from the language they currently speak and they are likely to have undergone language shifts in the past [109–111].
To avoid sample-size biases, for some analyses (e.g., local ancestry inference, and analyses using the masked and imputed dataset) BSP with large sample sizes were randomly downsampled to 30 individuals, and we obtained 1,495 individuals from 124 populations in the downsampled AfricanNeo dataset.

1.5. Population structure analyses

To explore patterns of population structure within the studied populations, we applied four dimensionality reduction methods (DRM) for genotype data. First, we used the uniform manifold approximation and projection (UMAP) approach [52] directly on genome-wide SNP data. We used an in-house script (available in GitHub: https://github.com/Hammarn/Scripts/blob/master/POPGEN/ UMAP_plot_bed.py). Second, we performed PCA for the data assembled in each dataset using *smartpca* software from the Eigensoft package v7.2.1 [53]. We then plotted PCA results between PC projections from PC1 to PC10. Third, we performed PCA-UMAP to combine the information of the first ten PC projections [54]. We used an in-house Python script (available in GitHub: https://github.com/Hammarn/Scripts/blob/master/POPGEN/UMAP_plot.py). This method considers neighboring samples around each data point in the PCA and seeks a lower-dimensional representation that preserves the distances between the points in the neighborhood. Fourth, we applied a deep learning framework for dimensionality reduction based on genotype convolutional autoencoder (GCAE) [55].
To better visualize the results, we provided plots highlighting each group and each studied population with different colours and markers. We plotted the results by using in-house Python scripts and the bokeh visualization library [112] for interactive plots (in *.html format) that are available online at Github (https://github.com/Schlebusch-lab/Expansion_of_BSP_peer-reviewed_article) [113].

1.6. Unsupervised clustering analyses

Admixture fractions were estimated using ADMIXTURE software v1.3.0 [56,114]. To cluster individuals based on SNP genotypes, we carried out an unsupervised ADMIXTURE clustering analysis. First, we investigated African populations from the AfricanNeo dataset. To investigate the geographical distributions of ADMIXTURE results at K=4, we plotted estimated admixture proportions on a geographic map. We used the grid-based mapping Surfer software v15 (Golden Software Cartographic Reference Information, LLC) and applied the Kriging method for spatial interpolation. Unsupervised ADMIXTURE analyses were also performed on the basis of only the AfricanNeo dataset from K=2 to K=25 using 10 independent runs with a random seed for each K-group, and default settings. A cross-validation (CV) test was performed for each run of each K-group. To visualize ADMIXTURE results for all the K-groups, we used PONG v1.5 [115]; however, population labels were difficult to plot and read for most of the 124 labels. To better visualize the major mode of the ADMIXTURE result, we plotted the Q matrix for the major mode in the 10 runs in bar plots using the AncestryPainter graphic program v5.0 [116]. For a better

comparison, the width of each population was set to be equal regardless of its sample size. We selected the results for the K-groups that are more informative for the studied populations (K=2, K=4, K=6, K=12, and K=16). In the main text of the article, we show K=12 rather than K=16, which had the lowest CV estimate, since the CV estimate for K=12 was very low as well (Supplementary Fig. 16). Moreover, the additional ancestry components that appear at K=16 are not relevant for the genetic variation within BSP, as they identify genetic structure within Nilo-Saharan speakers from Chad (teal component), Afro-Asiatic speakers from Ethiopia (green component), and North and South Khoe-San speakers (rose-brown and pink components, respectively) (see Supplementary Fig. 21). For each selected K-group, we used custom R and Python scripts to plot the averages for each estimated component in each population in pie charts on geographic maps, bar plots, and for ADMIXTURE results at K=6 in the ternary diagram with a hexagonal shape.

1.7. Testing analysis of f3- or f4-statistics

We formally tested the hypothesis of admixture in the present-day BSP using the f3- and f4-statistics in the form *f3(Yoruba; admixture-source*, *Target*) and *f4(Target, Yoruba; admixture-source, CHB)*, respectively. The *f3*-statistics test assessed if any of the BSP (*Target* population) had significant genetic affinities with a local non-BSP as the source of admixture. The *f4*-statistics test assessed if any of the BSP (the target population) was more closely related to either the outgroup Chinese-Han (CHB) or a local non-BSP as the admixture source. To disentangle the differential contribution of different hunter-gatherer groups into western and southern BSP, we also performed *f3*-statistics tests in the form *f3(Ju'hoansi; Baka, Target)* and f4-statistics tests in the form *f4(Target, Yoruba; Baka,Ju'hoansi)*. For these analyses, we used the package ADMIXTOOLS v2 (https://github.com/uqrmaie1/admixtools) under the R environment and functions *qp3pop* and *qpdstat*, respectively, with default options. To maximize the amount of information available, we run the test on the SNPs common to each triplet (respectively, quadruplet) of attested populations.

To assess the affinity of each studied ancient individual to a present-day BSP, we used f3-statistics analysis in the form *f3(Yoruba; ancient-sample, BSP)* for the 12 new aDNA individuals. To maximize the number of SNPs available for each sample in each analysis, we merged each ancient individual with the unmasked AfricanNeo dataset separately, and performed f3-statistics analysis for each aDNA individual together with the comparative populations.

1.8. Local ancestry deconvolution approach

To estimate the continental and subcontinental haplotypic admixture along with phased chromosomal segments (ancestry tracts), we applied a local admixture inference approach using RFMix software v1.5.4 [37]. To maximize phasing accuracy, the dataset was phased using the Haplotype Reference Consortium reference panel [117], and the HapMap Phase II b37 reference map as a genetic map [65]. To deconstruct genomes of BSP into six ancestry tracts, we compared haplotypes of BSP with the haplotype diversity of six panels of putative source populations. Each panel included 25-30 randomly selected individuals from:

(*i)* 30 Niger-Congo-speaking individuals (8 Esan-ESN, 13 Yoruba-YRI, and 9 Igbo);
(*ii)* 30 Western hunter-gatherer individuals (14 Cameroon_BakawRHG and 16 CameroonGabon_BakawRHG);
(*iii)* 30 Eastern hunter-gatherer and Nilo-Saharan-speaking individuals (13 Hadza, 7 Sabue, and 10 Gumuz);
(*iv)* 30 Afro-Asiatic-speaking individuals (9 Amhara, 6 Oromo, and 15 Somali);

(v) 25 Khoe-San-speaking individuals (15 Ju'hoansi, 4 GuiGhanaKgal, and 6 Karretjie); and (*vi)* 30 Eurasian individuals (10 Iberian-IBS, 10 European descendants-CEU, 5 Lebanese, and 5 Yemeni individuals).

To avoid the influence of admixture patterns in BSP in our ancestry-specific analyses, we removed haplotypes without West-Central African (WCA)-related ancestry in each haploid genome of each Bantu-speaking individual using a masking approach. Haplotypes with WCA-related ancestry were previously identified using RFMix with two expectation-maximization (EM) runs ("--use-reference-panels-in-EM -e 2" option). We used 25 generations ago since the admixture event ("-G 25" option) because that was the median number obtained in the admixture timing analyses (see below: *"Admixture timing inference"*). To avoid issues with missing data after masking, we phased and imputed the masked haploid genomes of studied Bantu-speaking individuals using SHAPEIT2 for phasing, IMPUTE v2.3.2 [118,119] for imputation and Niger-Congo-speaking populations from Nigeria (YRI, ESN, and Igbo genomes; 306 individuals in total) as a reference panel for this phasing and imputation. We then merged masked, phased, and imputed data of BSP with unmasked data of reference populations from previous studies to compare the outcome of this approach for masking, phasing, and imputation (Supplementary Fig.s 105–106).

To look at the affinities of the removed fragments we conducted Ancestry-specific (AS-)PCA of the removed fragments in BSP projected onto the reference groups (Supplementary Fig. 107). We caution however about over-interpretations of the plots of removed fragments since we observed previously that ancestry assignments do not work well when shared ancestry with a parental group is below 70% ancestry.

We also compared our admixture fractions from RFMix and ADMIXTURE estimated for each studied Bantu-speaking individual (Supplementary Fig. 33). The correlation between the estimated values using both methods is generally very good, except for two cases where RFMix assigns larger admixed ancestry fractions compared to ADMIXTURE: East-African and Khoe-San ancestries. Thus, these two ancestries might be overmasked in studied BSP. However, because our aim was to remove as much as possible non-Bantu admixture from BSP genomes, over-masking is a conservative scenario for our purpose.

1.9. Runs of homozygosity and inbreeding coefficients

To calculate runs of homozygosity (ROH), we used a sliding-window approach and followed recommendations from Ceballos et al. [120]. First, we extracted all the individuals from each population, and we performed more restrictive filtering separately for each population (to remove SNPs due to "--maf 0.01" and "--hwe 0.001"). For each population, we then used PLINK v1.9 to apply the following parameters: 30 was the minimum number of SNPs that one ROH was required to have ("--homozyg-snp 30"); 300 was the length in kb of the sliding window ("--homozyg-kb 300"); 30 was the required minimum density to consider one ROH that means one SNP in each 30 kb ("--homozyg-density 30"); 30 was the number of SNPs that the sliding window must have ("--homozyg-window-snp 30"); 1000 was the length in kb between two SNPs to be considered in two different segments ("--homozyg-gap 1000"); 1 was the number of heterozygous SNPs allowed in each window ("--homozyg-window-het 1"); 5 was the number of missing calls allowed in a window ("--homozyg-window-missing 5"); 0.05 was the proportion of overlapping window that must be called homozygous to define a given SNP as in a "homozygous" segment ("--homozyg-window-threshold 0.05").

For ROH longer than 1.5 Mb, we calculated the following parameters: mean ROH size, sum of long ROH, and total length of ROH. For ROH shorter than 1.5 Mb, we calculated the sum of short ROH. For each population, we also calculated six ROH length classes: class 1 (between 0.3<ROH<0.5 Mb), class 2 (between 0.5<ROH<1 Mb), class 3 (between 1<ROH<2 Mb), class

4 (between 2<ROH<4 Mb), class 5 (between 4<ROH<8 Mb), and class 6 (between 8 Mb<ROH<10Mb). The genomic inbreeding coefficient based on ROH (or F_{ROH}) was obtained as the total sum of ROH >1.5 Mb divided by the total length of the autosomal genome (3 Gb) [121,122].

1.10. Detection of shared segments inherited from a common ancestor

To investigate recent demographic events, we analyzed identity-by-descent (hereafter IBD) segments for each population included in the AfricanNeo dataset. We employed the fastIBD algorithm in the Beagle package v4.1 [123] to detect shared identical-by-descent segments between pairwise populations. Firstly, we calculated the total amount of IBD shared between individuals in tested populations. Then we calculated the average amount of shared IBD in centimorgans (cM) between individuals in tested populations. We removed IBD segments of 3cM to avoid the conflation effect of short IBD segments [124]. To estimate effective population sizes over the last 50 generations, we used IBDNe [63]. To investigate the effect of admixture on IBDNe segments, we used ancestry-specific (AS-)IBDNe [125]. We followed recommendations from [125], and we selected Bantu-speaking populations from western, eastern, and southern Africa with different sample sizes and influenced by different sources.

1.11. Estimating the timing of and strength of founder events

To infer both the age and strength of demographic founder events in BSP, we used ASCEND v10 [64]. This method estimates the correlation in allele sharing across the genome between pairs of individuals to recover signatures of past bottlenecks in each studied population. We performed ASCEND analysis for each population included in the AfricanNeo dataset, using default settings for all the autosomal chromosomes (i.e., distance bins from 0.1cM to 30.0cM by steps of 0.1cM) and default settings for plotting the results. We estimated the age since the founder event (*Tf*, in generations before the present, BP), the strength of the founder event (% *If*), and the 'normalized root mean squared deviation' (NRMSD) between the empirical decay curve and the theoretical decay curve. To convert the inferred dates since the founder event from generations to years, we used the following equation: 1950 –(g * 29) , where g is the estimated number of generations and 29 is the number of years for one generation [126]. To identify significant founder events, we followed the four criteria recommended by Tournebize et al. [64]: (*i*) the 95% confidence intervals of the estimated founder age and intensity do not include 0; (*ii*) the estimated founder age is lower than 200 generations and its associated standard error is lower than 50 generations; (*iii*) the estimated founder intensity is greater than 0.5%; and (*iv*) the NRMSD is lower than 0.29.

1.12. Haplotype diversity and linkage disequilibrium analyses

To further investigate the diversity of our study populations, we calculated haplotype heterozygosity (HH) and haplotype richness (HR) following recommendations from Schlebush et al. [12]. We used in-house scripts to estimate those parameters in haplotype windows ranging in size from 1 kb to 100 kb (with 1 kb increments). These metrics measure the number of different haplotypes in each population, and uneven sample size in a population can have a large influence on the calculation. To circumvent this issue, we excluded populations with fewer than 10 individuals and randomly subsampled the remaining populations to 10 individuals. Each calculation was also repeated 10 times and only the average result was reported. For each population and subsample, we removed SNPs with more than 10% missing data and minimum allele frequency (MAF) lower than the threshold of 10%. Following the recommendations of Schlebusch et al. [12], windows with 5 or more SNP were downsampled to 5, and windows with

fewer SNPs were excluded from the analysis. Values were calculated per chromosome and then averaged across the genome.

To characterize linkage disequilibrium (LD) patterns of all populations with more than 10 individuals (in total 124 populations), we measured the correlation coefficient (r^2) between all pairs of SNPs within 500 Kbp windows using PLINK. To control for uneven sample sizes, all populations with more than 10 individuals (119 out of 124 populations) were sub-sampled to 20 chromosomes (randomly without replacement). We repeated this process ten times, calculating each summary statistic in each replicate, and taking the average over replicates as the final estimate. For each population and subsample, we removed SNPs with more than 10% missing data and a minimum allele frequency (MAF) of less than 10%. For plotting, we computed the mean r2 and the mean distance between pairs of SNPs for all SNP pairs within bins of size 10 Kbp (50 bins in total). The effect of the choices of MAF-cutoff and bin size has previously been shown to have no impact on observed patterns of LD and relative levels of LD [127].

We repeated this procedure for the unmasked and masked AfricanNeo and Only-BSP datasets. In the unmasked dataset, populations have a minimum sample size of 10 individuals and a maximum size of 30 individuals. For the masked dataset, we computed the LD only for populations with a minimum sample size of 7.

To assess whether LD patterns of BSP were consistent with a history of expansion from the homeland in Cameroon/Nigeria, we calculated the correlation between LD and geographic distance from Cameroon, assuming that the BSP expansion started there. The geographic distance was calculated as spherical distance with the function *distGeo* of the *geosphere* R package [69]. In Cameroon, the position around the middle of the country (longitud=11.831477; and latitud=5.291058) was chosen to calculate the distance.

1.13. Phylogenetic analyses

To investigate phylogenetic relationships between populations included in the AfricanNeo dataset, we used the maximum-likelihood (ML)-based software TreeMix v1.13 [66]. To find the best-supported tree and infer the best number of migration events, we generated a scenario with no migration events and then tested for a range of migration events between 1 and 10. Each proposed TreeMix topology was accessed by bootstrapping blocks of 500 SNPs and assigning a Khoe-San population (Ju'hoansi) as the root of the population tree [12,33]. ML-based population trees were plotted using MEGA v11 [128].

1.14. Testing models of isolation-by-distance

To investigate patterns of isolation-by-distance (hereafter IBD) between BSP and test four distinct population genetic models, we used SpaceMix software v0.13 [74]. SpaceMix uses geographic information coupled with genetic data to generate so-called “geo-genetic maps”. In these maps, the distance between populations is based on genetic distance rather than geographical distances between studied individuals/populations. The resulting geo-genetic maps are thus analogous to PC projections, in that variations in a 2D plane correspond to genetic variation. In short, the general underlying assumption evaluated with SpaceMix is that under an IBD pattern, geographic and geo-genetic positions will be similar, which is consistent with a pattern of isolation-by-distance.

We used SpaceMix to test model-testing four different isolation-by-distance models underlying the following population histories:

(*i*) Model A for populations without either migration or admixture (i.e., a full IBD model);

(*ii*) Model B where no migration, but admixture was allowed (i.e., an IBD with admixture model);

(*iii*) Model C where migration was allowed but not admixture (i.e., an IBD with migration model);
(*iv*) Model D where both migration and admixture were allowed (i.e., an IBD with both migration and admixture model).

Each tested isolation-by-distance model was run for 10^6 iterations and 8 fast runs were performed with 10^5 generations. The "best" fitting model was evaluated using Pearson correlations between the expected and the observed data. Only the same individuals that were masked and imputed were analyzed for the "unmasked" dataset and the "masked" dataset.

1.15. Testing spatially explicit models of the BSP expansion

To test different demographic scenarios for the BSP expansion, we used a spatiotemporally explicit population genetic framework [9,41]. Briefly, this framework represents the world as a grid of hexagonal cells, approximately 100 km wide. Each cell represents a local, panmictic population with simple population dynamics characterized by colonization of empty cells from occupied neighbors; simple population growth and exchange of migrants between occupied cells; and a maximum population size informed by paleo-climate reconstructions of net primary productivity [9,41]. In the original version of the model, Eriksson et al. [9] considered only the initial peopling of the world by anatomically modern humans (AMH) 70–50 kya. We here adapted the extension of the model to multiple local expansions as proposed by Raghavan et al. [41] to different scenarios of BSP expansions.

Following the initial expansion of AMH within Africa, we assume that the ancestral homeland of BSP was situated in a region of West Africa from which BSP migrated across a wider region of sub-equatorial Africa (Supplementary Fig. 81), experiencing sequential population bottlenecks and potentially mixing with local non-BSP along the way. From this region, we excluded areas of dense rainforests motivated by the harsh living conditions and relative unsuitability for farming in these areas. As described in the main text, we considered three demographic scenarios in which the BSP expansion proceeded north of the rainforest, south through a rainforest corridor, or using both northern and southern routes. To this end, we modified the basic scenario in two ways, by either (*i*) creating a corridor through the rainforest that allowed expansion south, or (*ii*) blocking expansion north of the rainforest by excluding a section of the territory of BSP (Supplementary Fig. 81). For each demographic scenario, we ran one million simulations with parameters drawn from an independent uniform distribution for parameters characterizing the BSP expansion, and from a log-uniform distribution for parameters describing the initial global expansion of AMH taken from Raghavan et al. [41]. The timing of the origins of BSP and onset of their expansion was assumed to be a randomly drawn generation, uniformly distributed in the range from 80 to 400 generations ago (corresponding to 2,000 to 10,000 years ago, respectively). In each simulation, we generated gene genealogies for 500 unlinked loci (using the Wright-Fisher model) for 131 individuals from 14 selected African populations previously published by the H3Africa Consortium [30] (Supplementary Table 9), for which we have whole-genome sequencing (WGS) data (mean coverage between 10x and 30x). Access to WGS data from the H3Africa Consortium was granted to C.M.S. (accessory numbers: EGAD0000100422, EGAD00001004316, EGAD00001004334, EGAD00001004393, EGAD00001004448, EGAD00001004505, EGAD00001004533, EGAD00001004557, and EGAD00001005076). R^2 values were calculated between predicted and observed genome-wide differences between pairs of individuals. The simulation framework was implemented in C++ and all the outputs were analyzed using Matlab (9.9.0.1718557 (R2020b) Update 6), and custom scripts. All code and scripts are available from the authors upon request.

1.16. Estimating effective migration rates

To investigate migration rates, we used three approaches. First, we used EEMS software [71]. Analysis was performed with the following settings: diploid=true; numMCMCIter=2000000; numBurnIter=1000000; and numThinIter=9999. The number of individuals, sites and demes varied between the different analyses depending on the dataset and sampling area. For the analysis of BSP, we used the following settings: “mean nIndiv=4003”; “nSites=191292”, and “nDemes=200”. Each analysis was repeated three times and an average was taken as input for the visualization as recommended in the EEMS manual. Then, the results were visualized in R v3.6.1 using the accompanying R library rEEMSplots [71]. For the plots of only BSP, the colors were added according to the following linguistic groups: north-western BSP, west-western BSP, south-western BSP, and eastern BSP.

Second, we used a recent implementation of EEMS called Fast Estimation of Effective Migration Surfaces software (FEEMS) [72], to further investigate spatial population structure across Africa. Unlike EEMS, FEEMS applies a Gaussian Markov Random Field (GMRF) model in a penalized-likelihood-based framework to infer whether populations are exchanging gene-flow with neighboring populations in a spatial graph of a “stepping-stone” model of first migration later followed by genetic drift. Third, we inferred dispersal surfaces based on shared IBD tracts using the program MAPS [73]. We investigated IBD length categories of 2–4cM for older generations and >6cM for recent generations, around 56 and 13 generations ago respectively [129]. Shared IBD segments were estimated using hap-ibd v1.0 [130], and repaired with the tool merge-ibd-segments (‘merge-ibd-segments.17Jan20.102.jar’). To visualize migration patterns and population sizes, we used the R package plotmaps (available at Github: https://github.com/halasadi/plotmaps).

1.17. Gene-flow barriers analysis on a grid

To further investigate the routes of migration for the BSP expansion, we applied the approach from Pagani et al. [70], here called GenGrad. In short, this approach allows a visual representation of spatial genetic barriers inferred from genome-wide genetic distances and displays a gradient of spatially interpolated allele frequencies. As we are investigating a much smaller area than all of Africa, Eurasia, and Oceania, a few adjustments were made to certain parameters. Namely the sigma value was decreased to 0.1 and the color bar was adjusted to only incorporate the measured values. The calculations were performed in MatLab version 9.12.0.1884302 (R2022a), and we used F_{ST} as the distance metric.

1.18. Admixture timing inference

To estimate admixture dates, we applied haplotype-based admixture inference methods. First, we used MOSAIC v1.4 [45] for two- and three-way admixture models on the basis of the phased AfricanNeo dataset. For haplotype phasing the AfricanNeo dataset, we used SHAPEIT v2.r904 [65], and the Haplotype Reference Consortium as a reference panel [117,131]. Recombination maps were interpolated from the HapMap Phase 2 genetic map. To minimize switch error rates [65], we used the following parameters: 500 states, 50 MCMC main steps, 10 burnin, and 10 pruning steps.

1.19. Correlations between linguistic, geographical, and genetic data

To test the correlations between matrices of genetic, linguistic, and geographical distances, we performed Mantel tests and partial Mantel tests using the R package ncf v1.3-2 [67]. For each test, we computed Pearson's correlation analysis using 100,000 permutations between the two matrices. For the matrix of genetic distances, we computed pairwise F_{ST} between BSP included in the ancestry-masked Only-BSP dataset using the EIGENSOFT package v7.2.1 [68]. For the matrix of geographic distances, pairwise distances were calculated as pairwise great circle distances (in km) between the studied populations using the R package *geosphere* [69].
To compare genetic and geographic distances between BSP with the linguistic distances between their languages, we collected sets of basic vocabulary in 62 different varieties of 44 different Bantu languages. Basic vocabulary is part of a language's lexicon which is considered the most stable and the least susceptible to borrowing because it bears upon concepts that are universally shared amongst human societies [132]. We compiled vocabulary for 92 such basic concepts, which have also been used in recent phylogenetic studies on BSP [18,29,133,134]. Most of the lexical data in our dataset have also been used in one or more of these linguistic studies. Supplementary Table 12 provides a comprehensive list of our linguistic dataset including a list of the original sources from which the linguistic data for each variety originate and the phylogenetic studies in which they have previously been used. It also includes representative geo-coordinates for each sampled Bantu language variety. Cognates are lexical roots originating in one and the same ancestral lexeme which related languages share through inheritance from a common ancestor. We have reviewed and updated all cognacy judgments (5,355 in total) and extracted a binary-coded root-meaning association matrix using Lexedata [135]. In total, 38 BSP matched between the linguistic dataset and our imputed Only-BSP dataset, thus each matrix of pairwise linguistic, geographical, and genetic distances matched the subset of 38 BSP. Our complete lexical dataset including cognacy judgments is freely accessible online (available at Github: https://github.com/Schlebusch-lab/Expansion_of_BSP_peer-reviewed_article) [113]. Linguistic data are available in Cross-Linguistic Data Format (CLDF; see files: cognates.csv, cognatesets.csv, forms.csv, languages.csv, parameters.csv, sources.bib, and Wordlist-metadata.json available at Github) [136].

1.20. Comparisons between ancient and present-day populations

We used *smartPCA* to project ancient individuals onto a background of present-day populations included in the Only-African dataset, as well as for a sub-dataset including BSP-related aDNA and Only-BSP and selected West-Central African populations (using "YES" option for the following parameters: allsnps, lsqproject, newshrink, and killr2). After merging haplodized modern samples and pseudo-haplodized aDNA individuals and LD-pruning steps, we used PLINK to remove variants due to minor allele threshold ("plink --maf 0.1") and LD-pruning ("plink --indep-pairwise 100 10 0.2"). Also, we used unsupervised ADMIXTURE analysis from K=2 to K=12 to infer clusters and ancestry proportions from the modern-day dataset that were then provided as input used to project the aDNA data using the projection approach (admixture "-P" option) of ADMIXTURE software [56,114].

2. Supplementary Notes

Supplementary Note 1. Further background on the BSP expansion

The BSP expansion was a complex pattern of movement of peoples, cultures, and languages spanning several thousands of years, and there are still many aspects and intricacies left to investigate, from the first expansion from the BSP homeland until the last expansion into southern Africa. Preliminary suggestions of these complexities are seen in a few recent studies. Semo et al. [15] investigated BSP in Mozambique and found a north-to-south cline in genetic relationship, complemented by a north-to-south gradient of decreasing genetic diversity. This supports a north-to-south dispersal of BSP along the Indian Ocean coast possibly associated with the Iron Age assemblages of the Kwale archeological tradition [137]. In another fine-scale study, Sengupta et al. [14] studied South African BSP and found a well-defined genetic substructure among southeastern BSP from South Africa. Speakers of eight of the nine major Bantu languages in South Africa were included in the study and could genetically be distinguished from each other. The genetic substructure correlated to both geography and language. Seidensticker et al. [20] found archeological evidence for several waves of habitation by BSP in the central African rainforest, and suggested that the BSP expansion might have involved several spread-over-spread events [20].

Further evidence of possible spread-over-spread events from genetic data was reported by Sengupta et al. [14] for southeastern BSP from South Africa. The study reported deeper admixture dates with local San groups in the Tsonga and Venda than in all studied Sotho- and Nguni-speaking groups (including Pedi, Tswana, Southern Sotho, Swazi, Zulu, and Xhosa). This indicates complex events during the settling of BSP in southern Africa, where the Tsonga and Venda might be the descendants of earlier waves of migration and the other BSP of later waves. Archeological sources indicate that the ancestors of Nguni speakers migrated to Southern Africa from eastern Africa about one thousand years ago (kya) [138], while the Tsonga are mentioned to be among the waves of earlier BSP that migrated along the east coast of Africa [139]. Linguistically speaking, however, all Southern Bantu (Guthrie Zone S) languages, including Tsonga, Venda, Nguni and Sotho groups, descend from a most recent common ancestor, which is unique to them within eastern BSP [29]. Thus, linguistic data is at odds with genetic and archeological inferences and might indicate that the ancestors of Tsonga and Venda shifted to more recently arrived languages (closely related to Sotho and Nguni languages). Alternatively, only part of the Tsonga and Venda ancestry might link to earlier migrations into the area, while the other part of their ancestry might have come with Tsonga and Venda speakers.

Supplementary Note 2. Patterns of population structure and genetic diversity

To assess the genetic relationships between BSP, we first performed principal component analysis (PCA) for each assembled dataset. Following linguistic classifications, we grouped BSP into four major linguistic groups: north-western Bantu 2 (in brown), west-western Bantu (in green), south-western Bantu (in dark blue), and eastern Bantu (in red) (Supplementary Fig.s 3a, 4a and 4b). In this classification, we also included as south-western BSP the Damara-KSP from Namibia noting that this population speak a Khoe-Kwadi language, and we excluded BSP such

as the Baka sampled in Cameroon and Gabon because they were included in the western rainforest hunter-gatherer group (wRHG), following indications from previous studies [4,103,110].

To visualize genetic variation in the African continent, we applied four dimensionality reduction methods (DRM) for genetic data. First, we used the uniform manifold approximation and projection (UMAP) method directly on the genotype data [52]. UMAP results highlight separate groups for African populations from western and eastern hunter-gatherer groups (e.g., Baka, Hadza, and Sabue) and Nilo-Saharan-speaking populations (e.g., Gumuz) and East Asian populations (Supplementary Fig.s 6a–b). After zooming in on the results, we observed a good similarity between the position of the populations and their geographical distribution (Supplementary Fig.s 6c–d). We then applied the UMAP approach to a sub-dataset after removing the outlier groups from Supplementary Fig.s 6a–b (e.g., Gumuz, Hadza, Sabue, Baka, and East Asian populations). In the UMAP plot of selected populations (Fig. 1b), we also observed patterns of population structure and admixture. For instance, in the UMAP plot Fula individuals from Gambia are between western African populations and Eurasian populations, as previously reported [26,140–142]. In addition, we observed complex population structure among Nilo-Saharan-speaking populations from the north and south regions of Chad, as previously reported [105,141,142]. Interestingly, populations from southern Chad [105] might have admixture with Ubangi-speaking populations from CAR from this study.

PCA results of populations included in the Only-BSP dataset evidenced separate major BSP groups in our dataset (Supplementary Fig. 7). We then selected sub-Saharan African populations from the African-Neo dataset. PCA results for selected populations (Supplementary Fig. 8) better highlight a strong genetic differentiation between Khoe-San-speakers and other African populations on the first principal component (PC1), between Afro-Asiatic-speaking populations and other African populations on PC2, between wRHG populations and the other African groups on PC3 and PC4, and between BSP from Namibia (Himba and Herero) and the other African groups on PC6. Interestingly, new Ubangi-speaking populations from CAR included in the AfricanNeo dataset evidence genetic affinities with Afro-Asiatic-speaking populations and Nilo-Saharan-speaking populations from Chad and the southern region of Sudan (Supplementary Fig. 8). After rotating according to geographic sampling through Procrustes projection, the PCA for all sub-Saharan African populations better highlights the genetic diversity and differentiation among sub-Saharan African groups that have different linguistic backgrounds, lifestyles, or geographical distribution (Fig. 1c and Supplementary Fig. 9). We also performed PCA for all newly genotyped samples from Africa that were included in the Full-Genotyped dataset (Supplementary Table 1) plus reference sub-Saharan African populations included in the AfricanNeo dataset (Supplementary Table 2) after merging and quality control (Supplementary Fig. 10). PCA results of all available sub-Saharan African populations further highlight the observed patterns described before.

To investigate the genetic relationships between BSP and worldwide populations, we then performed PCA based on the AfricanNeo dataset (Supplementary Fig. 11). As expected, the PCA plot for all the populations included in the AfricanNeo dataset highlights continental ancestries of African versus Eurasian populations on each side of PC1, and between European and East Asian populations on PC2, and individuals from each major BSP group are close in the multidimensional space (Supplementary Fig. 11). On PC3, we observed the split of Khoe-San populations from southern Africa. We then used the UMAP algorithm to combine the information of the first 10 PCs of the PCA estimated for the AfricanNeo dataset (Supplementary Fig. 11). PCA-UMAP results (Supplementary Fig. 12) showed BSP grouping together, and splitting away from other African populations that were separated into different groups in the multidimensional space, except for BSP which seem to have a continuous connection that is in agreement with

the geographical locations of the populations (see zoomed region in Supplementary Fig.s 12c–d).

Lastly, we applied a deep learning framework for dimensionality reduction based on genotype convolutional autoencoder (GCAE) [55]. GCAE results further support the clustering of populations at the continental level between all studied worldwide populations included in the AfricanNeo database, and also some degree of differentiation among sub-Saharan African populations (Supplementary Fig.s 13a–b), notably different than the lack of differentiation between African populations on PC1 in Supplementary Fig. 11a. In addition, we also observed a better split of populations at the sub-continental level in Africa for the GCAE analysis on the basis of sub-Saharan African populations (Supplementary Fig.s 13c,d). GCAE results highlighted patterns of admixture and population structure that further supported our previous results. In general, dimensionality reduction methods (e.g., UMAP, PCA-UMAP, and GCAE) are shown to be able to capture finer population substructure among African populations than the first two projections of the PCA alone. Taken altogether, in the multidimensional space of all these results we observed genetic differentiation between and within BSP. In addition, there are different genetic patterns among eastern BSP who are geographically distant but who belong to the same linguistic group. Among northeastern BSP we observed the highest amounts of Afro-Asiatic-related admixture, while among southeastern BSP we observed the highest amounts of Khoe-San-related admixture. North-western, west-western, and south-western BSP were more similar genetically, except for three populations: the Himba and Herero populations, which have undergone more genetic drift, and the Damara-KSP, which has individuals with higher proportions of Khoe-San-related admixture (Supplementary Fig. 8c).

To further assess the human genetic landscape in Africa, we investigated the genetic diversity across worldwide populations using unsupervised ADMIXTURE analyses. We performed ADMIXTURE analyses from K=2 to K=25 on the basis of the AfricanNeo dataset. To better visualize patterns of population structure, we plotted ADMIXTURE results for selected K-groups using pie charts (for K=2, K=4, K=6, K=12, and K=16; Supplementary Fig.s 17–23), ternary diagrams (for K=6; Supplementary Fig. 24), and bar plots (for K=4 and K=16; Supplementary Fig.s 23–24). ADMIXTURE results at K=2 and K=4 (Supplementary Table 3) further indicated possible gene-flow from Eurasian ancestry into eastern and southern African populations (Supplementary Fig.s 17–18), in particular in eastern African populations such as Swahili-speakers from eastern Kenya [102] and in the South African Coloured groups [12,143]. In ternary diagrams of ADMIXTURE results at K=6 (Supplementary Fig. 24), we also observed a genetic cline between Niger-Congo-speaking populations from western to west-central African countries. ADMIXTURE results at K=12 (Extended Data Fig. 3 and Supplementary Fig.s 14–15) better highlight different components for hunter-gatherer populations in western Africa (yellow component), eastern Africa (black component), and southern Africa (purple component) (Supplementary Fig. 20). Those components are also present in BSP from different regions with different proportions of possible admixture. We furthermore observed population substructure among sub-Saharan African populations, in agreement with previous research[7,13,26]. Eastern BSP had indications of admixture in several regions with eastern African populations (brown component; Extended Data Fig. 3b), Middle Eastern populations (gray component; Extended Data Fig. 3c), and eastern hunter-gatherer population (e.g., Hadza; black component; Extended Data Fig. 3i).

At K=16, the K-group with the lowest cross-validation (CV) value (Supplementary Fig. 16), we observed different clusters between and within groups of western and eastern BSP (e.g., cyan, green and orange components in Supplementary Fig. 26), in agreement with their genetic cluster sharing with other sub-Saharan African groups (Fig. 2b, Supplementary Fig.s 21, 23 and 26, and Supplementary Table 4). We formally tested the hypothesis of admixture and its regional

character using f3- and f4-statistics (Supplementary Fig.s 28–32 and Supplementary Tables 5–6). We detected evidence of admixture in some BSP with Afro-Asiatic speaking groups (Supplementary Fig. 28), western RHG groups (Supplementary Fig.s 29 and 32), or Khoe-San groups (Supplementary Fig.s 30 and 32).

Supplementary Note 3. Ancestry-specific analyses in BSP

To eliminate the influence of admixture on our analyses of BSP, we only analyzed haplotype segments with West-Central African-related (WCA) ancestry previously estimated using a local ancestry inference approach. Ancestry-specific (AS-)PCA results based on the masked, phased, and imputed dataset of Bantu-speaking individuals with haplotypes with at least 70% WCA ancestry (thereafter "masked Only-BSP" dataset) together with the unmasked dataset of reference panels evidence the null influence of admixture patterns in BSP that were grouped in all PC projections (Supplementary Fig. 34). AS-PCA results on the basis of the Only-BSP dataset evidenced fine population structure among BSP, with Himba and Herero populations from Namibia separate from other studied BSP on PC1 and PC2, while PC3 split northwestern BSP and western BSP (Supplementary Fig.s 34a–b). After removing Himba and Herero individuals from the analysis, AS-PCA better highlighted the geographical distribution of BSP in the multidimensional space without the influence of the admixture (Extended Data Fig. 5). Interestingly, after masking admixture in studied BSP, the Himba and Herero populations present the highest values of ROH-based genomic inbreeding coefficient (on average F_{ROH}=0.02), which is notably different from other BSP (Supplementary Fig. 48).

Supplementary Note 4. Patterns of consanguinity and founder events

To shed additional light on the demographic history and cultural practices of BSP, we analyzed eleven patterns of runs of homozygosity (ROH) (Supplementary Table 7 and Supplementary Fig.s 35–47). In general, BSP have low values of the total sum of short ROH (Supplementary Fig. 35). The kurtosis and skewness of the violin plots also provide additional information. BSP are relatively homogeneous with very short tails and an almost normal distribution, while other African groups like Afro-Asiatic-speaking populations and Khoe-San-speaking populations, present other types of shapes. For long ROH segments (Supplementary Fig. 36), the highest values among all studied worldwide populations were detected in the Hadza population in Tanzania and the Sabue population in Ethiopia (on average: 0.48; Supplementary Table 7). Among BSP, the Himba and Herero populations in Namibia have the highest values of ROH-based inbreeding coefficient (F_{ROH}= 0.021 ±0.012SD and 0.015 ±0.007SD, respectively; see Supplementary Table 7 and Supplementary Fig. 39), highlighting genetic isolation in these populations likely after the BSP expansion to southwestern Africa. We also analyzed in each population their average for six ROH length categories (Supplementary Fig.s 40–47). Interestingly, the highest averages among BSP were detected in the Himba and Herero populations for categories 4 and 5 of ROH length (Supplementary Fig.s 41, and 45–46). In agreement with previous studies [144,145], they have the highest values of the mean long ROH (Supplementary Fig. 37), total length of long ROH (Supplementary Fig. 38), and genomic inbreeding coefficient (Supplementary Fig. 39), highlighting patterns of strong genetic isolation or genetic drift.

An additional cause of the increased F_{ROH} and the apparent bottleneck in the Herero population might be attributed to the Herero genocide of the early 1900s, a campaign of ethnic extermination where over 100,000 Herero individuals were murdered by the German government at the time (in German South West Africa, present-day Namibia). Although this event certainly had a severe impact on the Herero population, we do not think that this event was the cause of the observed ROH signal. Since the Herero population included here was sampled in the 1980s (and individuals were adults), the sampled individuals would correspond to the first or second generation after the genocide. We do not expect this recent event to impact the ROH statistic, because after such a recent bottleneck, the individuals remaining after the bottleneck still retain the diversity of the population before the bottleneck. This is one of the reasons why methods for demographic inference using the same kind of information (e.g., approaches based on sequential Markov coalescence (SMC) [146]) are not sensitive to recent population size changes. Thus, we expect that the ROH patterns observed for the Herero population are due to older and potentially prolonged processes. In addition, these ROH patterns are not specific to the Herero population only; similar ROH patterns are observed in the Himba and Damara populations, which suggests that common features, such as the pastoralist way of life (as commented in the main text), maybe the main factor.

Supplementary Note 5. Effective population size and founder events in BSP

To infer population changes over the last millennium, we estimated effective population sizes (Ne) for the last 50 generations using IBDNe (“ibdne.19Sep19.268.jar”) [63]. In general, IBDNe results highlight population expansions in the last 10-20 generations for all the studied populations (Supplementary Fig.s 49–50). Among BSP, we detected different patterns between populations from different countries or within populations from the same country (Supplementary Fig. 50). For instance, eastern BSP from Uganda show an early expansion around 28 generations ago, and declines in more recent generations. Admixture from different can also influence the overall effective population size in some BSP (Supplementary Fig. 51), in agreement with previous studies in admixed populations [125].

Founder events were investigated using ASCEND v10.0 [64]. Following the four criteria recommended by Tournebize et al. [64], among BSP the results evidenced significant founder events in 19 populations in Namibia (Himba and Herero), Eswatini (Swazi), Zambia (Fwe), Tanzania (Swahili population in Zanzibar), Botswana (Ghanzi), South Africa (Bhaca, SEBantu, Xhosa, Zulu, and ZuluAGDP), and Mozambique (Bitonga, Chopi, Makhuwa, Ndau, Nyanja, Tewe, Tswa, and Yao) (Supplementary Table 8). Among BSP, the highest intensity was found for the founder event in the Himba (I_f=1.6%, 95% CI:1.5–1.7%; T_f=21 generations, 95% CI: 18.7–24.1) and Herero populations (I_f=1.2%, 95% CI:1.1–1.3%; T_f=29 generations, 95% CI: 25.6–31.6) (Supplementary Fig. 52 and Supplementary Table 8), suggesting that these populations have been more genetically isolated from other groups (Supplementary Fig. 53). Among the BSP with significant founder events, the oldest date was estimated in the Chopi population from Mozambique (T_f=87 generations; 95% CI: 73–100.5), followed by other populations in Mozambique (Supplementary Table 8).

Supplementary Note 6. Patterns of isolation-by-distance among BSP

One of the simplest models for relationships between populations is the isolation-by-distance model (IBD in this section). Under an IBD model, populations close to each other are genetically more similar than populations far apart and this differentiation should increase with greater geographic distance. To investigate these patterns we used the software SpaceMix [74] on both the unmasked and masked Only-BSP datasets. Four models were tested: (a) No migration and no admixture; (b) No migration but admixture; (c) Migration but no admixture; and (d) Both migration and admixture. To better understand the SpaceMix results, we plotted each model separately with no population text overlays, with text instead of dots, with the sources of admixture, and with the correlations of each tested model. For all four models, including the IBD model, there was a high correlation between the observed data and the data estimated from the model (Supplementary Fig. 57 and 61). In the unmasked Only-BSP dataset (Supplementary Fig. 54–57), we see a strong adherence to IBD patterns although the model with admixture (b) or migration (c) has slightly higher correlations (Supplementary Fig. 57). In the dataset where we masked out the admixed parts of the genomes of BSP (Supplementary Fig.s 58–61) the IBD model receives the highest likelihood. The Herero population is outside the plot area in for instance Supplementary Fig. 59c but can be seen in the wider plot in Supplementary Fig. 60c. This is likely due to the demographic bottleneck observed in this population (Supplementary Fig. 52).

In addition, SpaceMix analyses suggested patterns of IBD within the BSP in our dataset, even when masking out admixture in BSP. Previous genetic studies have indicated that the Khoe-San populations of Southern Africa follow a very strong pattern of IBD [101] and that these IBD patterns extend to hunter-gatherer populations across the African continent [10]. It might thus be suggested that IBD patterns observed within BSP can be influenced or even explained by admixture with local hunter-gatherer groups. SpaceMix results however indicate that IBD patterns between Bantu-speaking groups remain and even become stronger when admixed parts of the genome were removed after masking and only the Bantu-speaking-related component was considered.

Supplementary Note 7. Patterns of haplotype diversity and LD among BSP

To further investigate genetic diversity within our dataset, we calculated haplotype richness (HR) and haplotype heterozygosity (HH) following recommendations from Schlebusch et al. [12]. These statistics were calculated for increasing window sizes starting at 2000 bp and increasing by 1000 up to a window size of 100,000. As previously reported [12], sub-Saharan African populations are the most genetically diverse human populations in the world (Supplementary Fig.s 62–64). Among BSP, HH and HR followed a pattern of decreasing diversity along the suggested paths of the BSP expansions, with increases in diversity in regions with high patterns of genetic admixture, such as South African BSP and around the Central African BSP (Supplementary Fig. 70). South African BSP generally have a high level of Khoe-San admixture, while RHG admixture is present in BSP close to the Congo rainforest in Central Africa.

Population structure and genetic drift have large effects on these diversity metrics, as is apparent when looking at the Himba population of Namibia (Supplementary Fig. 71). Furthermore, the masking, phasing and imputation approach to individually analyze west-central African-related haplotypes comes at the cost of a lack of haplotype diversity (Supplementary Fig. 70), as the

admixed haplotypes are replaced with West African ones.

To further examine the genetic patterns of the BSP expansion, we correlated these statistics with the distance from the homeland of BSP (Supplementary Fig.s 71–72). We expect that these metrics would generally decrease the further a population is from the homeland of BSP as genetic variation is lost along with the expansion/migration i.e., a serial-founder effect. For the haplotype heterozygosity metric estimated on the basis of the Only-BSP dataset without masking and imputation, we observe a correlation with the distance from Cameroon (R^2=0.126, *P*-value=0.0006; Supplementary Fig. 72a), as well as for the masked and imputed Only-BSP dataset (R^2=0.165, *P*-value=0.0040; Supplementary Fig. 72b). In agreement, for the haplotype richness metric, we also observed a correlation (R^2=0.136; *P*-value=0.0002; Supplementary Fig. 71a) for the original data, and a correlation of 0.267 (*P*-value=0.0002; Supplementary Fig. 71b) for the masked and imputed Only-BSP dataset. The predicted genetic pattern is in general observed in BSP with high admixture with hunter-gatherer groups (higher than expected) or genetic drift (lower than expected values). Masking out admixture thus increases the correlation between distance from the homeland of BSP and a decrease in genetic variation.

In general, short-range LD patterns provide information on ancient events (long-term N_e and LD) [147]. When looking at short-range LD in the unmasked dataset (Supplementary Fig. 65), East Asian populations have the highest values, followed by European and Middle-Eastern populations and lastly African populations. Among sub-Saharan African populations, South African Khoe-San populations showed the lowest LD, as previously shown [12]. Long-range LD (up to 500 Kbp) reflects more recent events. When looking at long-range LD, a different pattern was visible in some studied populations. Interestingly, some sub-Saharan African populations (e.g., Sabue in Ethiopia and Hadza in Tanzania) have long-range LD slightly higher than European populations. Sabue and Hadza have notably higher LD (Supplementary Fig. 65), due to their observed high genomic inbreeding (Supplementary Fig. 39).

An LD gradient was observed among BSP (Supplementary Fig.s 65a and 73a), in which South African BSP had the lowest short-range LD and BSP in Mozambique had the highest one. Admixture with hunter-gatherer groups could account for this pattern in South Africa, while BSP in Mozambique show very small evidence of RHG admixture, in agreement with a previous study [15]. To evaluate the effect of admixture on LD patterns in BSP, LD calculations were performed on the masked Only-BSP dataset. We selected BSP with more than 70% of West-Central African-related ancestry and plotted them together with six reference populations (Supplementary Fig. 65b and 73b). After masking, LD was higher in several BSP (max value=0.275 in the Pedi population from South Africa, while 0.155 was estimated in the unmasked dataset). Also, we see a spatial pattern of LD increasing from west-central Africa towards the east and the south (Supplementary Fig. 73b), which is more coherent with a history of expansion from their homeland between the border of Nigeria and Cameroon than the patterns observed in the unmasked dataset (Supplementary Fig. 73a). Assuming that BSP started their migration in this region, we would expect a genome-wide increase of LD in BSP situated farther away from the origin of the expansion in Cameroon. For that reason, we estimated the correlations between LD patterns and geographic distances in BSP (Supplementary Fig. 74). The correlation was positive and significant. LD at 50 Kb increased with spatial distance from Cameroon in BSP (Supplementary Fig. 75a; adjusted R-squared: 0.2258; *P*-value=0.00002). The correlation is still significant, albeit less strong, for the masked dataset (Supplementary Fig. 75b; adjusted R-squared=0.0946; *P*-value=0.0180). The difference between the unmasked and the masked dataset seems to be mainly due to Mozambican populations, whose LD decreases with imputation, and South African BSP, largely absent from the masked dataset because of their small sample sizes. BSP in Mozambique showed higher values of homozygosity than populations from nearby regions (visible in short ROH length Supplementary Fig. 35 and

Supplementary Table 7), so it is reasonable to think that the imputation introduced some diversity disrupting the range of LD. By comparison, the correlation was not significant when all African populations (n = 111) were included in the analysis (adjusted R-squared=0.0039; P-value=0.2329).
The observed gradual decline of genetic diversity from West Africa to other regions in Africa provides support for a serial founder model rather than an isolation-by-distance model (Supplementary Note 6) or a simple bottleneck model. Additionally, the gradual decline in admixture dates with local groups (Fig 2c), as we move further away from West Africa, reinforces the notion that IBD or single bottleneck models are unlikely. In this respect, the spread of BSP mirrors the spread of modern humans out of Africa, which involved humans spreading over substantial distances and involving multiple generations, and is best modeled by a serial bottleneck.

Supplementary Note 8. Phylogenetic analyses of BSP

To investigate patterns of population splits and migration events among BSP, we used TreeMix-based phylogenetic trees [66]. For the AfricanNeo dataset, African and Eurasian populations split into separate branches in the phylogenetic tree, Khoe-San populations root the tree, and sub-Saharan African populations separate into different branches in agreement with their geographical distributions, lifestyles and linguistic backgrounds (Supplementary Fig.s 76–79). For TreeMix results based on the unmasked Only-BSP dataset (Supplementary Fig.s 79–80), northwestern BSP were at the base of the phylogenetic tree, while western and eastern BSP split from each other. The main branches are north-western Bantu-speakers, west-western Bantu-speakers, south-western Bantu-speakers, and eastern Bantu-speakers. Among eastern Bantu-speakers, there are two main branches for north-eastern BSP and the south-eastern BSP. Therefore, the separation of BSP was in agreement with their observed admixture patterns with other African groups described above, and also with the linguistic classification of BSP [148], and their geographical distribution. For the masked Only-BSP dataset (Extended Data Fig. 7 and Supplementary Fig. 76), eastern BSP were together in the same branch without the subdivision due to admixture patterns with other African groups.

Supplementary Note 9. Patterns of pairwise genetic distances and admixture graphs

For the masked Only-BSP dataset, F_{ST} values evidenced the lowest values among BSP from West-Central Africa to the rest of sub-Saharan Africa (Supplementary Fig. 82). We used the lowest F_{ST} values between neighboring BSP to infer the putative migration routes during the BSP expansion (Fig. 5a and Supplementary Fig.s 83a–b). To do so, for each African country we selected one BSP that is most (geographically) distant from the BSP in Cameroon and by depicting one arrow from the target population to the BSP with the lowest pairwise F_{ST} value in our dataset (Supplementary Fig. 83). After finding the direction of the first arrow, we continue the analysis using as a target the destination of the arrow and excluding for the new analysis the previous target population. For instance, we started with the Zulu population from South Africa, and the lowest pairwise F_{ST} value of this BSP was with the Tsonga population from South Africa,

and for the Tsonga population the lowest value was with the Lozi population from Zambia (after excluding the Zulu for the analysis), and we repeat the same procedure until reaching the Nzime population in Cameroon. Using this approach repeatedly, we depicted the arrows connecting the BSP that are more distant from the homeland of BSP, and we reconstructed the putative migration routes (Fig. 5a and Supplementary Fig. 83a). We evaluated these migration routes using qpGraph to build an admixture graph of the putative routes (Supplementary Fig. 83c). BSP from western DRC seem to be the starting point of the migration route of BSP to southern Africa, in particular the Lozi population from Zambia, and from the Lozi to South Africa, Mozambique and Zimbabwe (Supplementary Fig. 83a). We took this exploratory approach with caution, and we repeated the analysis after removing the Lozi population from Zambia due to the wide distribution of this population in Zambia (Supplementary Fig. 83b). With or without including the Lozi population in the analyses (Supplementary Fig.s 83a and 83b, respectively), in both analyses the results showed one migration wave from Cameroon to western DRC, and different migration waves from western DRC to eastern and southern BSP. The connection between BSP from western DRC and southwestern BSP was also observed, suggesting that western DRC is likely to have been an important crossroad region for the expansion of BSP to most of the sub-equatorial African regions.

Supplementary Note 10. Estimation of effective migration surfaces

To further investigate migration rates over geographic space, we applied EEMS and FEEMS [71,72] (Supplementary Fig.s 84–89). In EEMS analyses with the masked Only-African dataset (Fig. 5c), low effective migration rates were observed at geographical barriers such as the eastern African Great Lakes regions, the southern African Kalahari desert, and the west-central African rainforest (Supplementary Fig.s 84–85). These areas also coincide with locations where groups with very different genetic ancestries co-exist with BSP. High genetic differentiation between BSP and non-BSP likely drives the patterns of low migration rates in these regions. EEMS analysis was also carried out on the unmasked and masked Only-BSP dataset (Supplementary Fig.s 86–87). The unmasked Only-BSP dataset recapitulates the findings of the masked Only-African dataset, where admixture within BSP from genetically distant neighboring groups is likely associated with inferred low effective migration rates. In the masked Only-BSP dataset (Fig. 5c), patterns of relationships restricted to the BSP genetic component are highlighted. There are regions of high migration rates along the Indian Ocean coast from Kenya to eastern South Africa. In contrast, we identify a region of low migration in Tanzania between three eastern African lakes (Lake Victoria, Lake Tanganyika, and Lake Malawi). Another region of low migration stretches up from South Africa and Zimbabwe, through Zambia and the DRC, possibly associated with a separation between western and eastern BSP. In Zambia, the barrier is between the western and the eastern regions and in between the western and eastern branches of the Bantu languages (Fig. 5c). In Zambia and the DRC, we have higher resolution analyses of the region in the form of ADMIXTURE profiles that include more populations with small sample sizes (less than 10 samples per population). In Supplementary Fig. 27, the ADMIXTURE profiles were consistent with population structure being present in these countries and different cluster contributions can be seen between populations from western and eastern regions in both countries, which aligns with the migration barriers inferred by EEMS. Regarding the area of low migration more to the south, the Botswana (Ghanzi) population shows similar ADMIXTURE profiles to the Mozambique/South Africa BSP populations. This aligns with the fact that this population is likely Tswana (southeastern BSP). It might be the reason why a blue “island” of

high gene flow around this population is visible, surrounded by areas of low gene flow. It might be that the barrier indicated here is rather driven by the many southwestern BSP (bright blue dots in Fig. 5c) present in Namibia, Angola and western Zambia. Finer resolution with more populations from Botswana and Zimbabwe might help to refine inferences across this region in the future.

Taken together, EEMS results identified an eastern coastal path as a region of high migration rates for the expansion of BSP, together with longitudinal corridors of lower migration rates in the central parts of the continent.

We also ran MAPS analyses (Al-Asadi et al. PLoS Genet 2019) to provide further insights into the observed migration rates at different times in the past (Supplementary Fig. 91). The MAPS approach estimates dispersal surfaces based on shared IBD tracts of different length categories and transforms the symmetric migration rate (m) estimated using EEMS into dispersal distance (σ) by scaling with the grid step-size (Al-Asadi et al. 2019). There are similarities between the migration surfaces of EEMS results (Supplementary Fig. 87) and the MAPS results for both the IBD category representing older generations (2–4cM representing ~56 generations ago) and the category representing more recent generations (>6cM, ~13 generations ago). The results of both categories indicate regions of lower gene flow overlapping with the geographic location of Zambia and higher gene flow along the coasts (although not completely overlapping). Interestingly the MAPS analyses of younger generations show barriers in the DRC across the rainforest, while in the analyses representing older generations there seems to be increased gene flow across the rainforest. For populations toward the periphery of the BSP expansion, it is unclear what the older generation MAPS analyses indicate as these populations might not all have reached their current geographic locations yet. Thus comparing time serial migration surfaces might make more sense for the areas around the BSP homeland.

Supplementary Note 11. Admixture dating and model-testing of the BSP expansion

To estimate the population sources and admixture dates in BSP, we used two approaches. First, we used MOSAIC [45]. Admixture testing using MOSAIC inferred the dates of admixture events between BSP and local populations from different regions (Fig. 2b, Supplementary Fig. 92 and Supplementary Table 10). As suggested by previous results, southwestern BSP have admixture with local populations of Khoe-San-related ancestry (ranging from 11 to 20 generations ago). Northeastern BSP have higher amounts of admixture with populations of Afro-Asiatic-speaking related ancestry than southeastern BSP (Supplementary Fig. 92). In South Africa, southeastern BSP have admixture with populations of Khoe-San-related ancestry (range: 20-24). Admixture dates correlate significantly with geographic distance from Cameroon (R^2=0.13, *P*-value=2.6e-05) with earlier dates in the Bantu-core region and more recent dates toward the extremes of the expansion (Fig. 2b and Supplementary Fig. 93).

Supplementary Note 12. Comparisons between ancient and present-day populations

We produced whole-genome sequencing data of 12 ancient individuals from six archeological sites in South Africa and three in Zambia (Supplementary Table 14). The age of the samples spanned the last 800 years according to radiocarbon dating presented in Supplementary Table 14 and in previous studies [8,48]. Ancient DNA sequences have a high frequency of cytosine to thymine (C to T) transitions at the 5' ends and of guanidine to adenine (G to A) at 3' ends due to post-mortem deamination. These typical damage patterns increase with the age of the sample and its conservation status. The nucleotide misincorporation patterns of our samples are shown in Supplementary Fig. 94 and Supplementary Table 14. In general, the damage patterns of our samples are consistent with their age, with seven out of 12 samples showing damage patterns higher than 10%. Mitochondrial contamination estimates were low for all samples, ranging from 0 to 0.03 (Supplementary Table 14), and showed no relation with damage patterns. Coverage ranged from 0.02X to 1X for the autosomal genome, and from 0.8 to 71X for the mitochondrial (mtDNA) genome. For all but one sample, we were able to retrieve the mtDNA-haplogroups, which all belonged to major African mtDNA-haplogroups: L0a, L0d, L1c, L2a, L3b, and L3e (Supplementary Table 14). All the identified mtDNA-haplogroups are haplogroups associated with BSP except the L0d haplogroup [149]. L0d is a Khoe-San-associated haplogroup [150] and was carried by three out of the six ancient individuals from current-day South Africa. The presence of this haplogroup in the Iron Age individuals is likely due to Khoe-San admixture, as was observed in previous ancient DNA studies [57] and studies on modern-day groups [149,150].

To compare the autosomal genetic diversity of ancient and present-day BSP, we merged the AfricanNeo dataset with 83 ancient DNA (aDNA) individuals from previous studies in Africa [23,57–62] (Supplementary Tables 13–14). Both, PCA and PCA-UMAP results evidenced genetic affinities between aDNA and modern African samples from the same geographic location (Fig. 1d, Extended Data Fig. 8 and Supplementary Fig.s 95–98), suggesting similar patterns of shared ancestry between samples from different time periods. Among BSP, we also observed population structure in DRC between the ancient individuals from western DRC with more Niger-Congo-speaking-related ancestry (in the two Kindoki samples from 150-230BP) and the eastern DRC sample with more eastern African-related ancestry (Matangai Turu from 750BP), as previously reported [23]. ADMIXTURE results at K=4 (Supplementary Fig. 98) and K=12 (Extended Data Fig. 8) for modern-day populations and aDNA individuals evidence complex patterns of admixture between Niger-Congo-speaking populations and ancient Pastoral populations in Kenya [58,59]. In southeastern Africa, Iron Age aDNA individuals and modern-day BSP provide evidence for genetic continuity in this region (Supplementary Fig. 101), as previously reported [14].

In our newly reported ancient individuals, we observe different cluster assignment profiles for the samples originating from current-day South Africa (Supplementary Fig.s 99–101) and current-day Zambia (Supplementary Fig.s 102–104). The South African samples are more homogenous and contain more of the "green" component maximized in modern-day southeast BSP from South Africa (SouthAfrica_SEBantu). Their cluster assignments are also similar to previously reported Iron Age individuals from the region, including Mfongosi, ElandCave, Newcastle and ChampagneCastle [57]. The four previously published and six new Iron Age samples from current-day South Africa also show more indications of Khoe-San admixture (gray component) compared to the six Zambia Iron Age samples. Compared to the South African Iron Age samples, the Zambian samples seem to have a more heterogeneous cluster assignment

and they contain more of the “dark blue” component maximized in the BSP Shi population from the eastern DRC as well as the “dark yellow” component maximized in the Niger-Congo speaking Esan population from Nigeria.

To quantify their affinity to present-day BSP, we estimated the f3-statistics for all our 12 new ancient individuals (Extended Data Table 2 and Supplementary Table 15). Among the aDNA individuals from current-day South Africa, five out of six aDNA individuals showed the two highest affinity values for the BSP from Ghanzi in Botswana and the Sotho population from South Africa, while one aDNA sample (WUD038b) had its highest affinity for the South African Sotho and Bhaca populations. For the six samples from current-day South Africa the top 4-6 highest affinity values were always to a current-day South African population or the Botswana ‘Ghanzi’ population. The ethnolinguistic affiliation of the Ghanzi population is not available - however they were likely Sotho-Tswana speakers, closely related to Sotho speakers from South Africa.

Samples originating from modern-day Zambia, instead, showed more heterogeneous affinities (Supplementary Fig.s 102–104), consistent with the notion that the region was a crossroad of interactions. For the six samples, two (WUD008, WUD0012) showed the strongest affinities with Swahili from Kenya, and the other four with the Chopi population from Mozambique (WUD003), Tonga- and Nyengo populations from Zambia (WUD004 and WUD018), and the Sotho population from South Africa (WUD010). The top 4-6 populations with the highest affinity to the six samples originating from current-day Zambia also vary substantially more in terms of geographic location, relative to the results for the six samples from current-day South Africa.

We reported morphological analyses, radiocarbon dating and dietary isotope analyses before for the four samples originating from Mumbwa Cave in Zambia, WUD003, WUD004, WUD008, WUD010 [8]. For WUD003, WUD004 and WUD010 (museum IDs A339, A340 and A346) the morphological sex could not be determined. Their biological sex based on DNA analyses were all XY. WUD008 (A344) was predicted to be male based on morphology and this was confirmed by the DNA analyses (Supplementary Table 14). The dietary isotope analyses found that most of the individuals from Mumbwa had similar diets to recent southern African farmers who depended largely on C4 crops and/or plant foods with relatively limited dairy and meat supplements. The WUD008 (A344) dietary isotope profile was different from the other individuals in the study in that his δ13C values indicated that he had a higher C3 plant intake, more similar to inland hunter-gatherer groups from southern Africa. His δ13N values however were not as high as hunter-gatherer groups [8]. Our genetic analyses confirmed that this individual is genetically associated with immigrant BSP and not with local hunter-gatherers. Within the heterogeneous profiles from the Zambian ancient individuals, the cluster assignment profile of WUD008 shows more of the eastern African BSP associated “dark blue” (or “DRC_Shi”-related) component and west African associated “dark olive” (or “Nigeria_Esan-ESN”-related) component, and less of the southeast BSP associated “light green” (or “SouthAfrica_SEBantu”) component in comparison with other ancient individuals from Zambia (Extended Data Fig. 8). In the f3-statistic analysis (Supplementary Fig. 102), we detected genetic sharing between this ancient individual and Swahili populations from Kenya.

We also reported radiocarbon dates and archeological descriptions for two ancient individuals from current-day South Africa (WUD034 - museum ID A294 and WUD037 - museum ID A302) [48]. The A294 individual was partially mummified and buried in a cave, Kaybar’s Cave, near Cathkin Peak in the Drakensberg. Morphological analyses on pelvis bones indicated a male individual and this is confirmed by the DNA analyses. The A302 individual was buried in Robinson’s Shelter 2, not far from Kaybar’s Cave and composed a nearly complete skeleton. This individual was also classified as male based on morphological analyses, which is confirmed here by DNA analyses. The details around the two burials are fully described in [48]. The two

individuals had similar cluster assignment profiles (Extended Data Fig. 8) that show a large proportion of Southeast BSP associated “light green” component. Their f3-statistics (Supplementary Fig. 100) associate them with the South African Sotho population and the Botswana Ghanzi group.

3. Supplementary Figures

3.1. Extended introduction and geographical locations of studied populations

a **“Early-split” hypotheses** **b** **“Late-split” hypotheses**

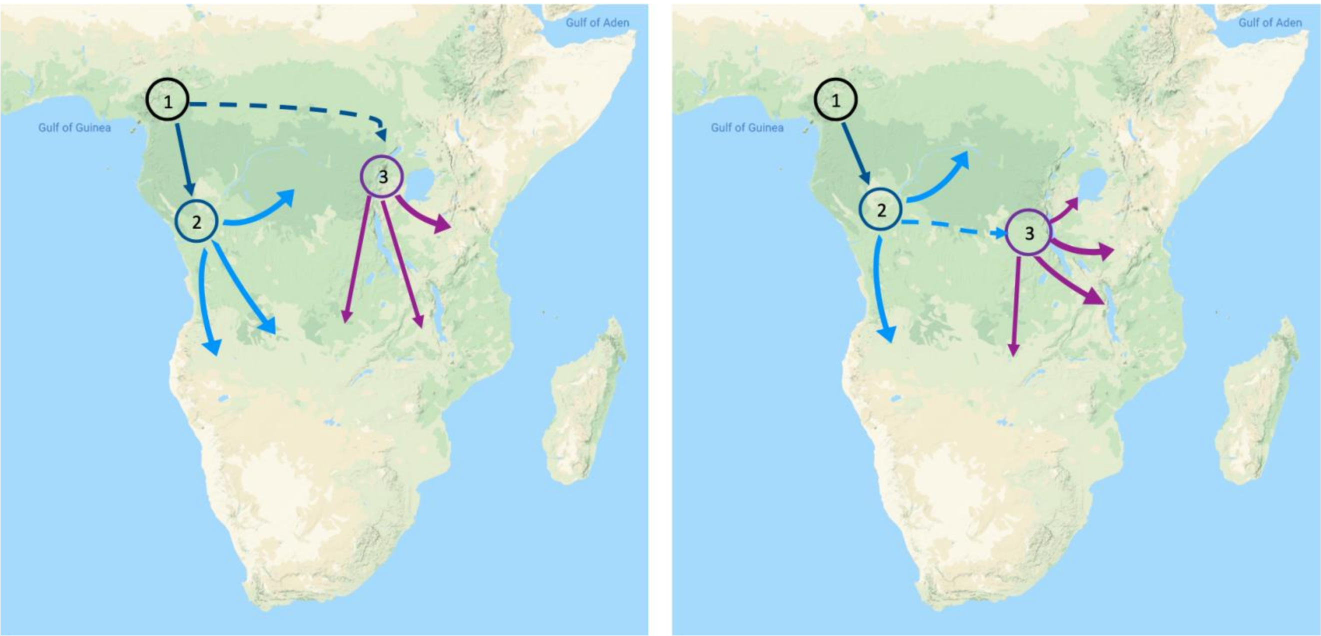

Supplementary Fig. 1 | Linguistic hypotheses proposed to explain the expansion of BSP. Illustration of the **a**, “Early-split” (or “northern route”) hypothesis and **b**, “Late-split” (or “southern route”) hypothesis based on linguistic sources. The main difference between the two is whether the eastern Bantu branch is a direct off-shoot of the Narrow Bantu languages (left) or a sub-branch of the western Bantu branch (right). The circles represent the presumed locations of (1) proto-Bantu, (2) the nucleus of the western Bantu branch, and (3) the nucleus of the eastern branch of the Bantu languages. Map data by GoogleMaps (©2023 Google).

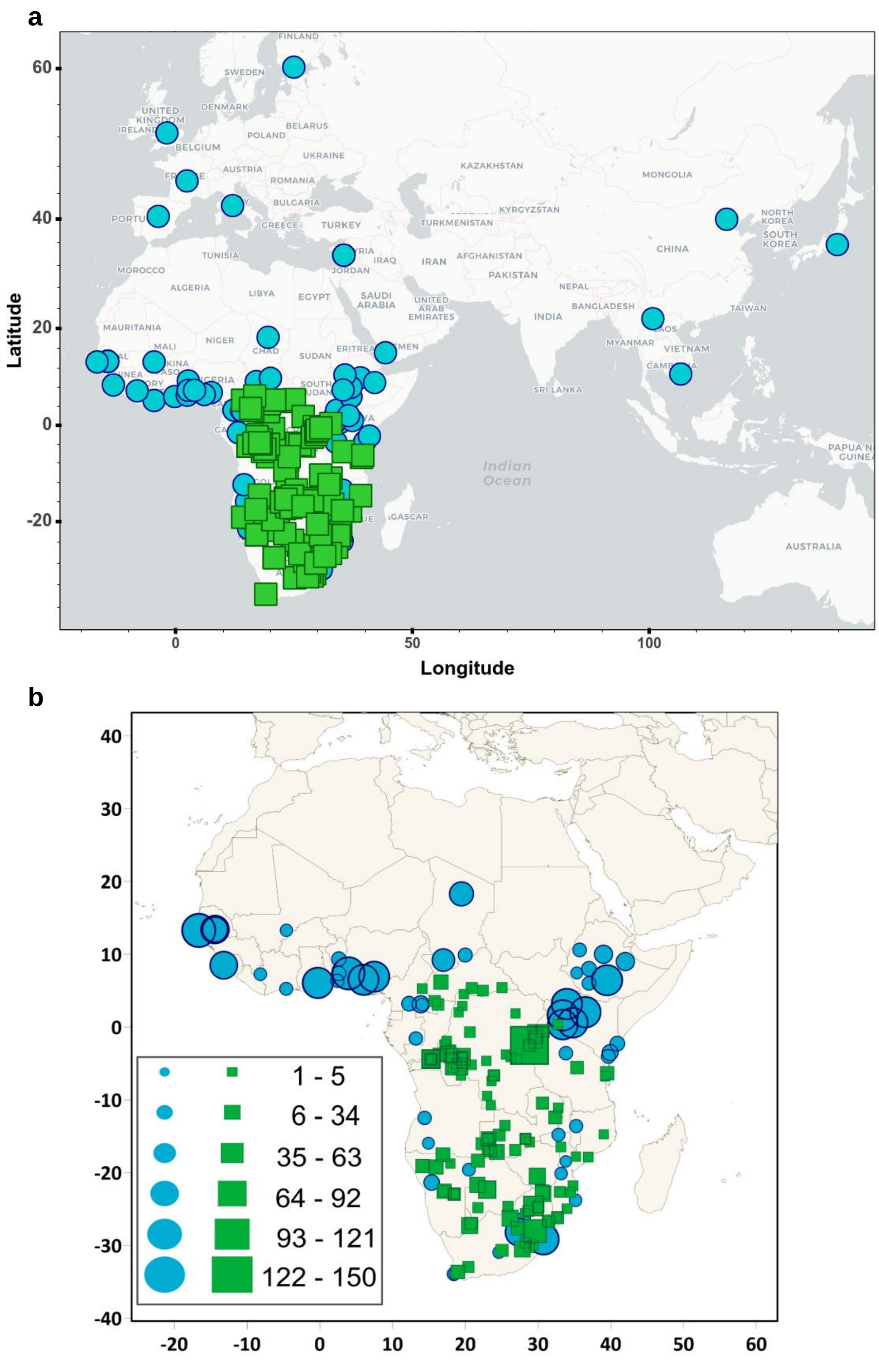

Supplementary Fig. 2 | Geographical locations of African and Eurasian populations.
Panel figure showing locations of 227 populations (5,341 individuals) included in the dataset after quality filtering and merging. This included the newly genotyped dataset (green squares) and reference populations from previous studies (blue circles) for a total of 214 African and 13 Eurasian populations. Details about the populations were presented in Supplementary Table 1–2. **a**, To better visualize the locations of each population, interactive plots are available at Github [113] (Suppl_Fig_2a_Map.html). Vector basemap and map tiles were provided by CartoDB (© CARTO 2023). **b**, Figure showing only sub-Saharan African populations. The size of the markers is in relation to the sample size of each population.

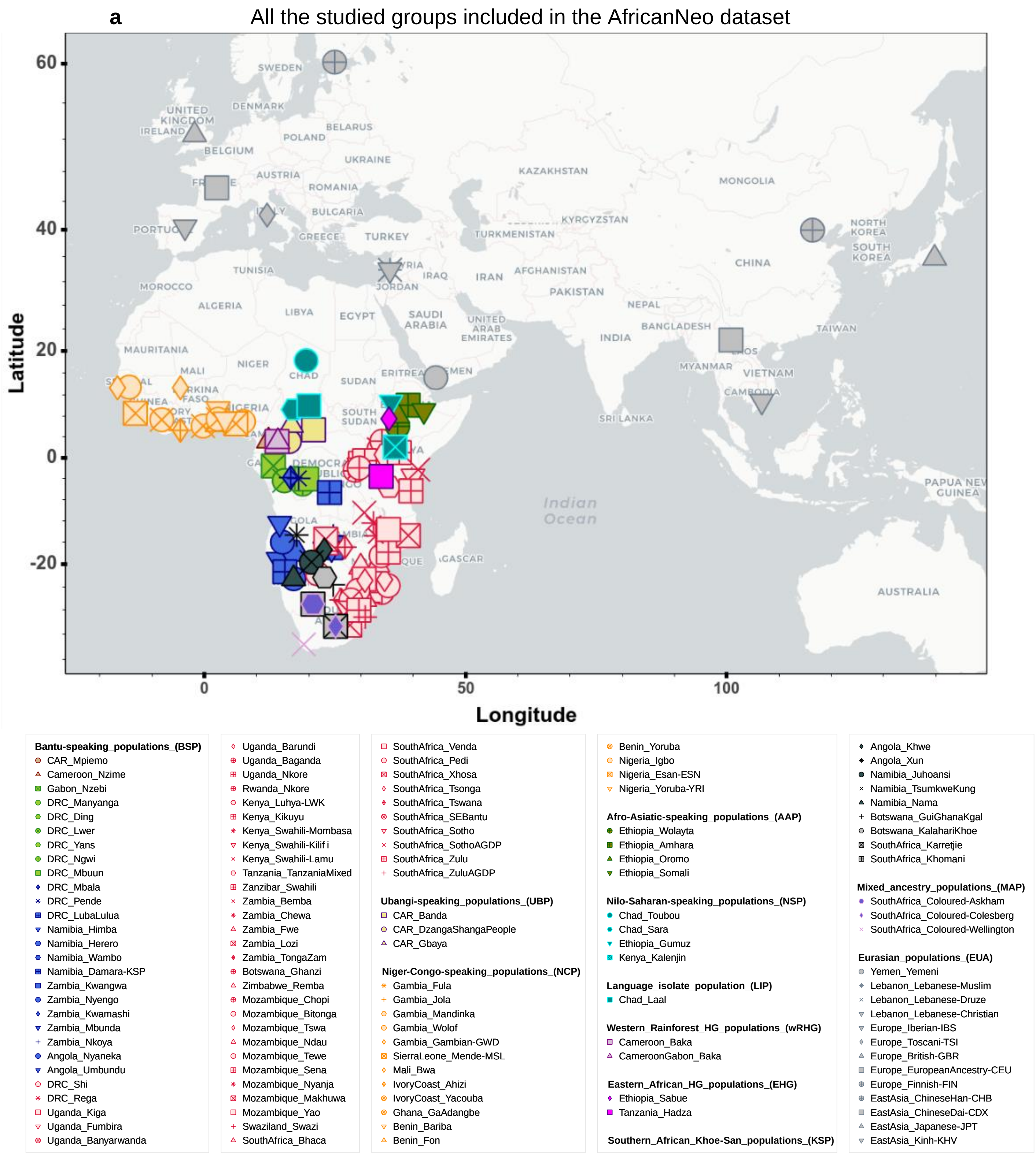

a
All the studied groups included in the AfricanNeo dataset
Latitude
Longitude
60
40
20
0
-20
0
50
100
SWEDEN
UNITED KINGDOM
IRELAND
DENMARK
BELARUS
POLAND
BELGIUM
UKRAINE
AUSTRIA
ROMANIA
BULGARIA
GREECE
TURKEY
KAZAKHSTAN
MONGOLIA
KYRGYZSTAN
TURKMENISTAN
NORTH KOREA
SOUTH KOREA
CHINA
TUNISIA
MOROCCO
ALGERIA
LIBYA
EGYPT
IRAQ
JORDAN
IRAN
AFGHANISTAN
PAKISTAN
SAUDI ARABIA
UNITED ARAB EMIRATES
NEPAL
BANGLADESH
INDIA
TAIWAN
MAURITANIA
MALI
NIGER
CHAD
SUDAN
ERITREA
MYANMAR
VIETNAM
SOUTH SUDAN
SRI LANKA
Indian Ocean
PAPUA NEW GUINEA
AUSTRALIA
Bantu-speaking_populations_(BSP)
CAR_Mpiemo
Cameroon_Nzime
Gabon_Nzebi
DRC_Manyanga
DRC_Ding
DRC_Lwer
DRC_Yans
DRC_Ngwi
DRC_Mbuun
DRC_Mbala
DRC_Pende
DRC_LubaLulua
Namibia_Himba
Namibia_Herero
Namibia_Wambo
Namibia_Damara-KSP
Zambia_Kwangwa
Zambia_Nyengo
Zambia_Kwamashi
Zambia_Mbunda
Zambia_Nkoya
Angola_Nyaneka
Angola_Umbundu
DRC_Shi
DRC_Rega
Uganda_Kiga
Uganda_Fumbira
Uganda_Banyarwanda
Uganda_Barundi
Uganda_Baganda
Uganda_Nkore
Rwanda_Nkore
Kenya_Luhya-LWK
Kenya_Kikuyu
Kenya_Swahili-Mombasa
Kenya_Swahili-Kilif i
Kenya_Swahili-Lamu
Tanzania_TanzaniaMixed
Zanzibar_Swahili
Zambia_Bemba
Zambia_Chewa
Zambia_Fwe
Zambia_Lozi
Zambia_TongaZam
Botswana_Ghanzi
Zimbabwe_Remba
Mozambique_Chopi
Mozambique_Bitonga
Mozambique_Tswa
Mozambique_Ndau
Mozambique_Tewe
Mozambique_Sena
Mozambique_Nyanja
Mozambique_Makhuwa
Mozambique_Yao
Swaziland_Swazi
SouthAfrica_Bhaca
SouthAfrica_Venda
SouthAfrica_Pedi
SouthAfrica_Xhosa
SouthAfrica_Tsonga
SouthAfrica_Tswana
SouthAfrica_SEBantu
SouthAfrica_Sotho
SouthAfrica_SothoAGDP
SouthAfrica_Zulu
SouthAfrica_ZuluAGDP
Ubangi-speaking_populations_(UBP)
CAR_Banda
CAR_DzangaShangaPeople
CAR_Gbaya
Niger-Congo-speaking_populations_(NCP)
Gambia_Fula
Gambia_Jola
Gambia_Mandinka
Gambia_Wolof
Gambia_Gambian-GWD
SierraLeone_Mende-MSL
Mali_Bwa
IvoryCoast_Ahizi
IvoryCoast_Yacouba
Ghana_GaAdangbe
Benin_Bariba
Benin_Fon
Benin_Yoruba
Nigeria_Igbo
Nigeria_Esan-ESN
Nigeria_Yoruba-YRI
Afro-Asiatic-speaking_populations_(AAP)
Ethiopia_Wolayta
Ethiopia_Amhara
Ethiopia_Oromo
Ethiopia_Somali
Nilo-Saharan-speaking_populations_(NSP)
Chad_Toubou
Chad_Sara
Ethiopia_Gumuz
Kenya_Kalenjin
Language_isolate_population_(LIP)
Chad_Laal
Western_Rainforest_HG_populations_(wRHG)
Cameroon_Baka
CameroonGabon_Baka
Eastern_African_HG_populations_(EHG)
Ethiopia_Sabue
Tanzania_Hadza
Southern_African_Khoe-San_populations_(KSP)
Angola_Khwe
Angola_Xun
Namibia_Juhoansi
Namibia_TsumkweKung
Namibia_Nama
Botswana_GuiGhanaKgal
Botswana_KalahariKhoe
SouthAfrica_Karretjie
SouthAfrica_Khomani
Mixed_ancestry_populations_(MAP)
SouthAfrica_Coloured-Askham
SouthAfrica_Coloured-Colesberg
SouthAfrica_Coloured-Wellington
Eurasian_populations_(EUA)
Yemen_Yemeni
Lebanon_Lebanese-Muslim
Lebanon_Lebanese-Druze
Lebanon_Lebanese-Christian
Europe_Iberian-IBS
Europe_Toscani-TSI
Europe_British-GBR
Europe_EuropeanAncestry-CEU
Europe_Finnish-FIN
EastAsia_ChineseHan-CHB
EastAsia_ChineseDai-CDX
EastAsia_Japanese-JPT
EastAsia_Kinh-KHV

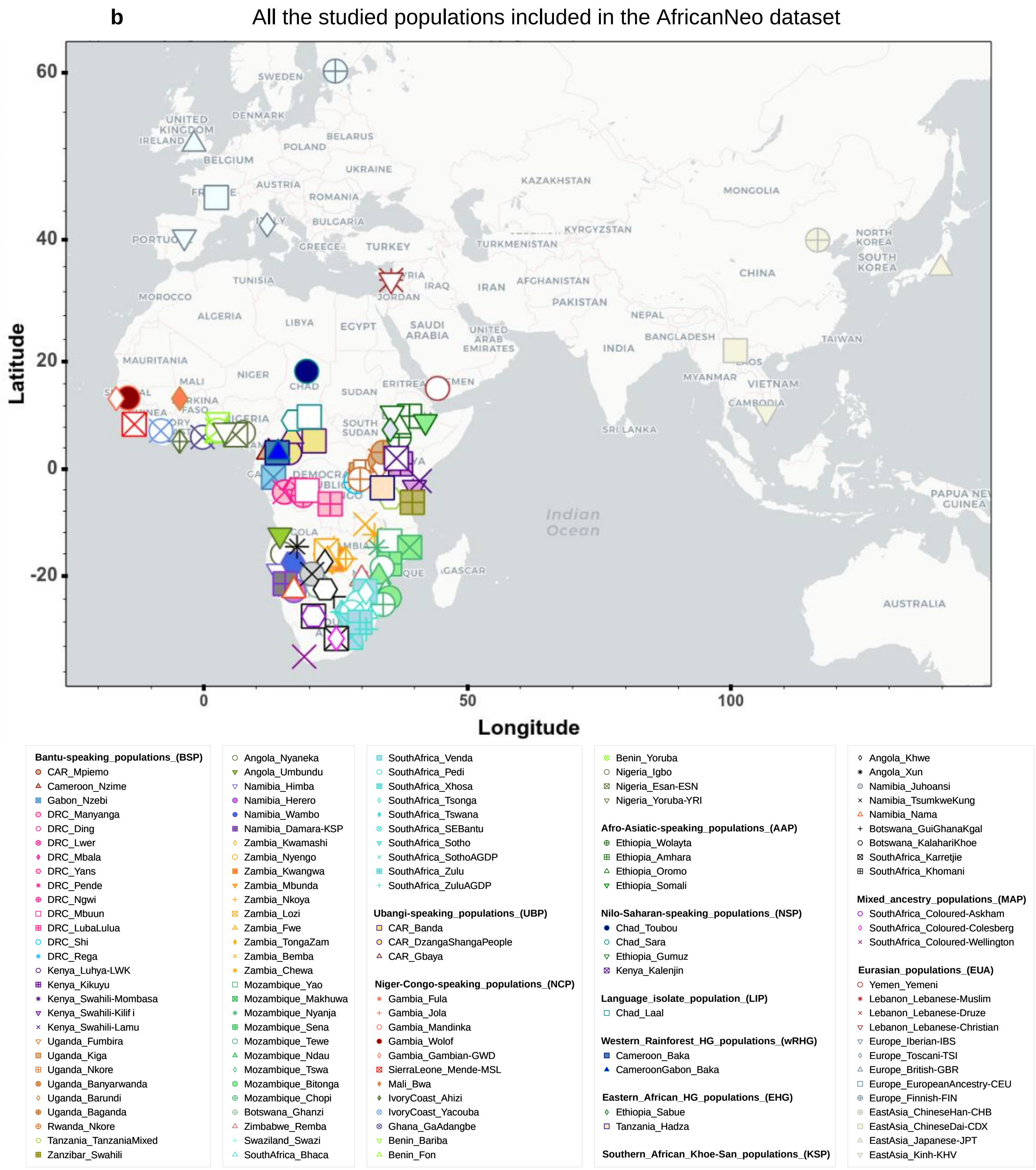

Supplementary Fig. 3 | All the groups and populations included in the AfricanNeo dataset. Figure showing the labels of **a**, each group and **b**, each population that was included in the AfricanNeo dataset for populations with at least 10 individuals (Supplementary Table 2). BSP were grouped in (a) into four major linguistic groups: north-western Bantu 2 (in brown), west-western Bantu (in green), south-western Bantu (in dark blue), and eastern Bantu speakers (in red). To better visualize the locations of each group and each population, interactive plots are available at Github [113] (Suppl_Fig_3a_Map.html and Suppl_Fig_3b_Map.html, respectively). Vector basemap and map tiles were provided by CartoDB (© CARTO 2023).

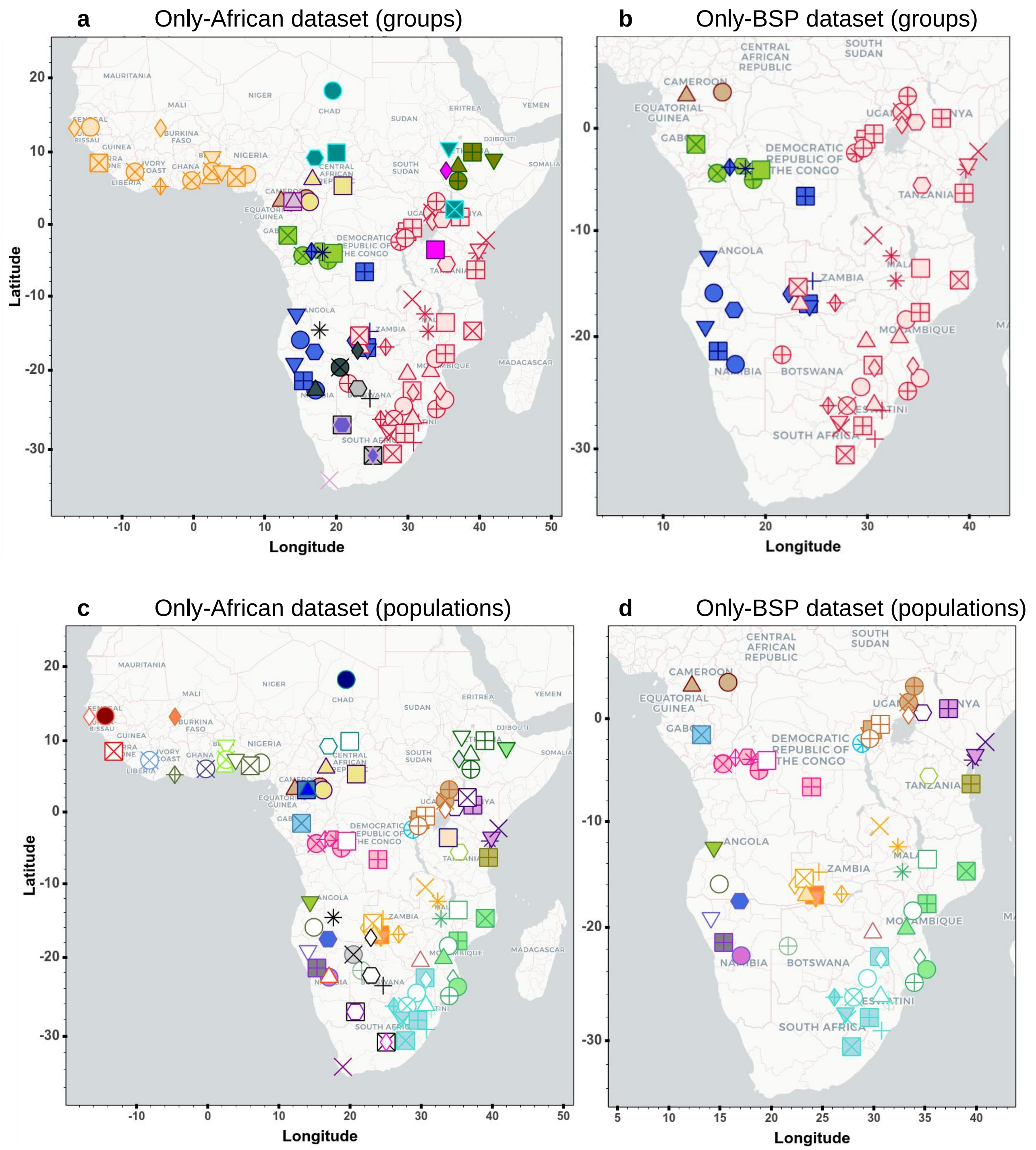

Supplementary Fig. 4 | All the populations included in the Only-Africa and Only-BSP datasets. Panel figure showing the labels of the populations that were included in the Only-African dataset for **a**, each group (also Supplementary Fig. 1a) and **c**, each population; and populations included in the Only-BSP dataset for **b**, each group and **d**, each population. Population labels are matching the labels presented in Supplementary Fig. 3. Further details about the populations are presented elsewhere (Supplementary Table 2). To better visualize the locations of each group and each population, interactive plots are available at Github [113] (Suppl_Fig_4[a–d]_Map.html). Vector basemap and map tiles were provided by CartoDB (© CARTO 2023). Legends were included in the next page.

CAR_Mpiemo
Cameroon_Nzime
Gabon_Nzebi
DRC_Manyanga
DRC_Ding
DRC_Lwer
DRC_Yans
DRC_Ngwi
DRC_Mbuun
DRC_Mbala
DRC_Pende
DRC_LubaLulua
Namibia_Himba
Namibia_Herero
Namibia_Wambo
Namibia_Damara-KSP
Zambia_Kwangwa
Zambia_Nyengo
Zambia_Kwamashi
Zambia_Mbunda
Zambia_Nkoya
Angola_Nyaneka

Angola_Umbundu
DRC_Shi
DRC_Rega
Uganda_Kiga
Uganda_Fumbira
Uganda_Banyarwanda
Uganda_Barundi
Uganda_Baganda
Uganda_Nkore
Rwanda_Nkore
Kenya_Luhya-LWK
Kenya_Kikuyu
Kenya_Swahili-Mombasa
Kenya_Swahili-Kilif i
Kenya_Swahili-Lamu
Tanzania_TanzaniaMixed
Zanzibar_Swahili
Zambia_Bemba
Zambia_Chewa
Zambia_Fwe
Zambia_Lozi
Zambia_TongaZam

Botswana_Ghanzi
Zimbabwe_Remba
Mozambique_Chopi
Mozambique_Bitonga
Mozambique_Tswa
Mozambique_Ndau
Mozambique_Tewe
Mozambique_Sena
Mozambique_Nyanja
Mozambique_Makhuwa
Mozambique_Yao
Swaziland_Swazi
SouthAfrica_Bhaca
SouthAfrica_Venda
SouthAfrica_Pedi
SouthAfrica_Xhosa
SouthAfrica_Tsonga
SouthAfrica_Tswana
SouthAfrica_SEBantu
SouthAfrica_Sotho
SouthAfrica_SothoAGDP
SouthAfrica_Zulu

SouthAfrica_ZuluAGDP
CAR_Banda
CAR_DzangaShangaPeople
CAR_Gbaya
Gambia_Fula
Gambia_Jola
Gambia_Mandinka
Gambia_Wolof
Gambia_Gambian-GWD
SierraLeone_Mende-MSL
Mali_Bwa
IvoryCoast_Ahizi
IvoryCoast_Yacouba
Ghana_GaAdangbe
Benin_Bariba
Benin_Fon
Benin_Yoruba
Nigeria_Igbo
Nigeria_Esan-ESN
Nigeria_Yoruba-YRI
Ethiopia_Wolayta
Ethiopia_Amhara

Ethiopia_Oromo
Ethiopia_Somali
Chad_Toubou
Chad_Sara
Ethiopia_Gumuz
Kenya_Kalenjin
Chad_Laal
Cameroon_Baka
CameroonGabon_Baka
Ethiopia_Sabue
Tanzania_Hadza
Angola_Khwe
Angola_Xun
Namibia_Juhoansi
Namibia_TsumkweKung
Namibia_Nama
Botswana_GuiGhanaKgal
Botswana_KalahariKhoe
SouthAfrica_Karretjie
SouthAfrica_Khomani
SouthAfrica_Coloured-Askham
SouthAfrica_Coloured-Colesberg
SouthAfrica_Coloured-Wellington

Legend for each group included in Supplementary Fig. 4a and 4b.

CAR_Mpiemo
Cameroon_Nzime
Gabon_Nzebi
DRC_Manyanga
DRC_Ding
DRC_Lwer
DRC_Mbala
DRC_Yans
DRC_Pende
DRC_Ngwi
DRC_Mbuun
DRC_LubaLulua
DRC_Shi
DRC_Rega
Kenya_Luhya-LWK
Kenya_Kikuyu
Kenya_Swahili-Mombasa
Kenya_Swahili-Kilif i
Kenya_Swahili-Lamu
Uganda_Fumbira
Uganda_Kiga
Uganda_Nkore

Uganda_Banyarwanda
Uganda_Barundi
Uganda_Baganda
Rwanda_Nkore
Tanzania_TanzaniaMixed
Zanzibar_Swahili
Angola_Nyaneka
Angola_Umbundu
Namibia_Himba
Namibia_Herero
Namibia_Wambo
Namibia_Damara-KSP
Zambia_Kwamashi
Zambia_Nyengo
Zambia_Kwangwa
Zambia_Mbunda
Zambia_Nkoya
Zambia_Lozi
Zambia_Fwe
Zambia_TongaZam
Zambia_Bemba
Zambia_Chewa

Mozambique_Yao
Mozambique_Makhuwa
Mozambique_Nyanja
Mozambique_Sena
Mozambique_Tewe
Mozambique_Ndau
Mozambique_Tswa
Mozambique_Bitonga
Mozambique_Chopi
Botswana_Ghanzi
Zimbabwe_Remba
Swaziland_Swazi
SouthAfrica_Bhaca
SouthAfrica_Venda
SouthAfrica_Pedi
SouthAfrica_Xhosa
SouthAfrica_Tsonga
SouthAfrica_Tswana
SouthAfrica_SEBantu
SouthAfrica_Sotho
SouthAfrica_SothoAGDP
SouthAfrica_Zulu

SouthAfrica_ZuluAGDP
CAR_Banda
CAR_DzangaShangaPeople
CAR_Gbaya
Gambia_Fula
Gambia_Jola
Gambia_Mandinka
Gambia_Wolof
Gambia_Gambian-GWD
SierraLeone_Mende-MSL
Mali_Bwa
IvoryCoast_Ahizi
IvoryCoast_Yacouba
Ghana_GaAdangbe
Benin_Bariba
Benin_Fon
Benin_Yoruba
Nigeria_Igbo
Nigeria_Esan-ESN
Nigeria_Yoruba-YRI
Ethiopia_Wolayta
Ethiopia_Amhara

Ethiopia_Oromo
Ethiopia_Somali
Chad_Toubou
Chad_Sara
Ethiopia_Gumuz
Kenya_Kalenjin
Chad_Laal
Cameroon_Baka
CameroonGabon_Baka
Ethiopia_Sabue
Tanzania_Hadza
Angola_Khwe
Angola_Xun
Namibia_Juhoansi
Namibia_TsumkweKung
Namibia_Nama
Botswana_GuiGhanaKgal
Botswana_KalahariKhoe
SouthAfrica_Karretjie
SouthAfrica_Khomani
SouthAfrica_Coloured-Askham
SouthAfrica_Coloured-Colesberg
SouthAfrica_Coloured-Wellington

Legend for each population included in Supplementary Fig. 4c and 4d.

3.2. Dimensionality reduction methods (DRM)

- **All genotyped samples:** 1,763 individuals and 163 populations in total for this study in the Illumina H3Africa array and HumanOmni2.5 array, ~2,271,503 SNPs (**Supplementary Table 1**).

  ↓ *Quality control (QC) using PLINK (--mind 0.15 --geno 0.1 --hwe 0.0000001)*

- **Database after the initial QC:** 2,221,827 autosome SNPs and 1,591 individuals.

  ↓ *Merging with datasets previously genotyped in:*

  Whole-genome sequencing studies (1000 Genomes Project Consortium et al. 2015),

  Illumina HumanOmni5 array (Fortes-Lima et al. 2017),

  Illumina HumanOmni2.5 array (Schlebusch et al. 2012; Vicente et al. 2019; Semo et al. 2020; Fortes-Lima et al. 2017; Gurdasani et al. 2015; Pagani et al. 2015; Haber et al. 2016; Crawford et al. 2017; Brucato et al. 2018; and Sjöstrand et al. 2021).

- **"Full-Genotyped" merged dataset**: 5,341 individuals from 227 populations and 482,459 SNPs.

  ↓ *Removed 103 BSP with less than 10 individuals per population.*

- **"AfricanNeo" dataset**: 4,950 individuals from 124 populations and 482,459 SNPs.
  ["AfricanNeo LD-pruned" dataset: 223,473 SNPs]

  ↓ *Removed 13 Eurasian populations.*

- **"Only-African" dataset**: 3,902 individuals from 111 sub-Saharan African populations and 482,459 SNPs.

  ↓ *Removed 57 non-Bantu-speaking populations (BSP).*

- **"Only-BSP" dataset**: 2,108 individuals from 67 BSP and 482,459 SNPs.

  ↓ *Large populations randomly downsampled to 30 individuals.*

- **"Masked Only-BSP" dataset**: 1,495 individuals from 67 BSP and 482,459 SNPs.

- **"Africa-aDNA" dataset**: Only-African dataset, 12 new aDNA individuals, and 83 aDNA individuals from previous studies (Skoglund et al. 2017; Schlebusch et al. 2017; Prendergast et al. 2019; Lipson et al. 2020, 2022; Wang et al. 2020; Coutinho et al. 2021). 5,036 individuals from and 482,459 SNPs.

  ↓ *Removed 57 non-Bantu-speaking populations (BSP).*

- **"aDNA-and-Only-BSP" dataset**: 2,194 individuals and 482,459 SNPs.

Supplementary Fig. 5 | Workflow for the assembled datasets.
Panel figure summarizing the quality control (QC) and data merging for the databases assembled for this study.

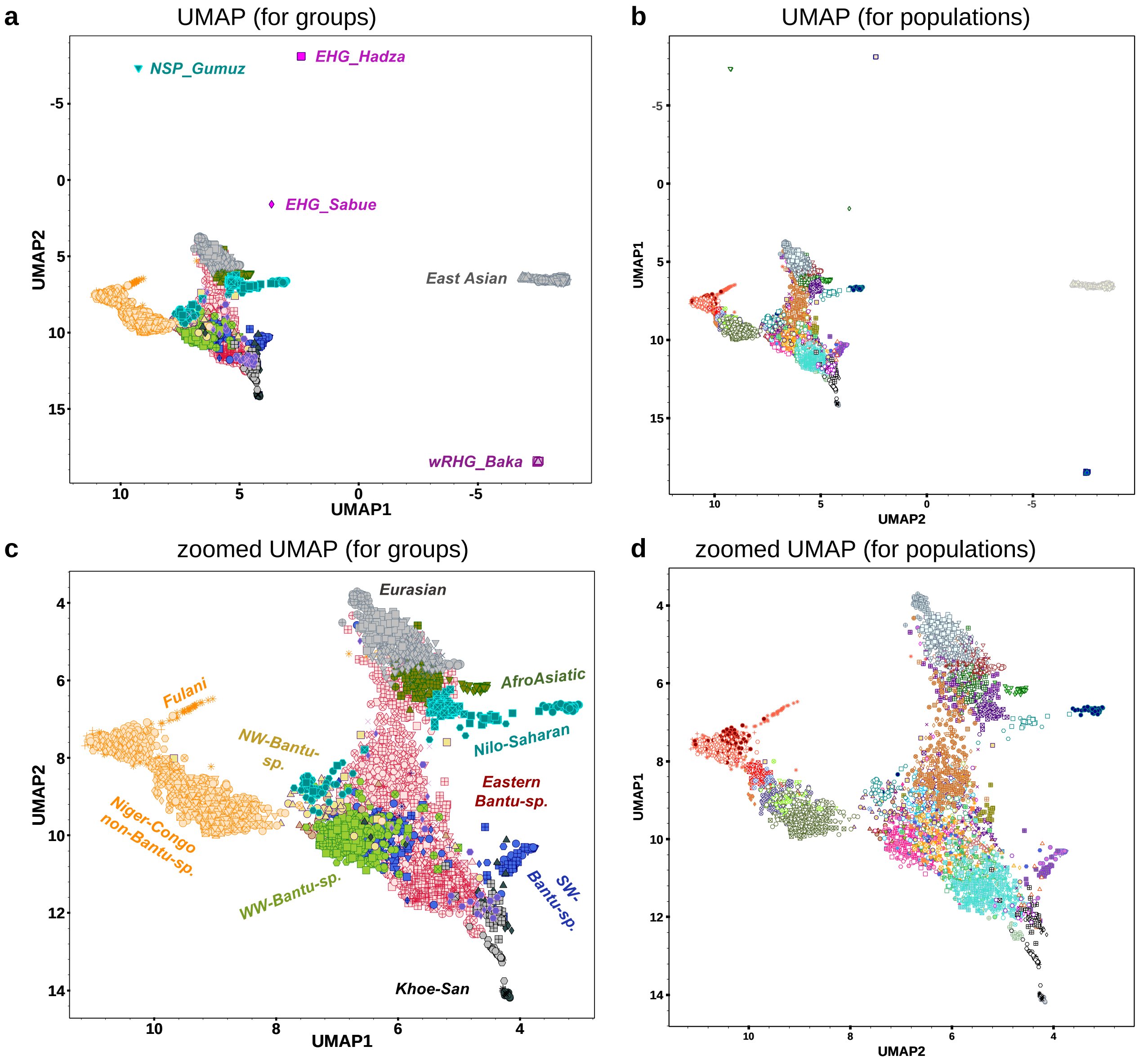

Supplementary Fig. 6 | UMAP approach applied on the basis of genotype data.
Panel figure showing UMAP results for all African and Eurasian populations included in the AfricanNeo dataset (Supplementary Table 2) using the Uniform Manifold Approximation and Projection (UMAP) algorithm directly on the genotype data without performing a PCA. This panel figure highlights results for **a**, each group listed in Supplementary Fig. 3a and **b**, each population listed in Supplementary Fig. 3b. The legend of this figure is the same legend as Supplementary Fig. 3. To better see the results for the studied BSP, we zoom in on each plot highlighting **c**, each group (also Supplementary Fig. 1b) and **d**, each population. To better visualize the results of each group and each population, interactive plots are available at Github [113] (Suppl_Fig_6[a–b]_UMAP.html).

a

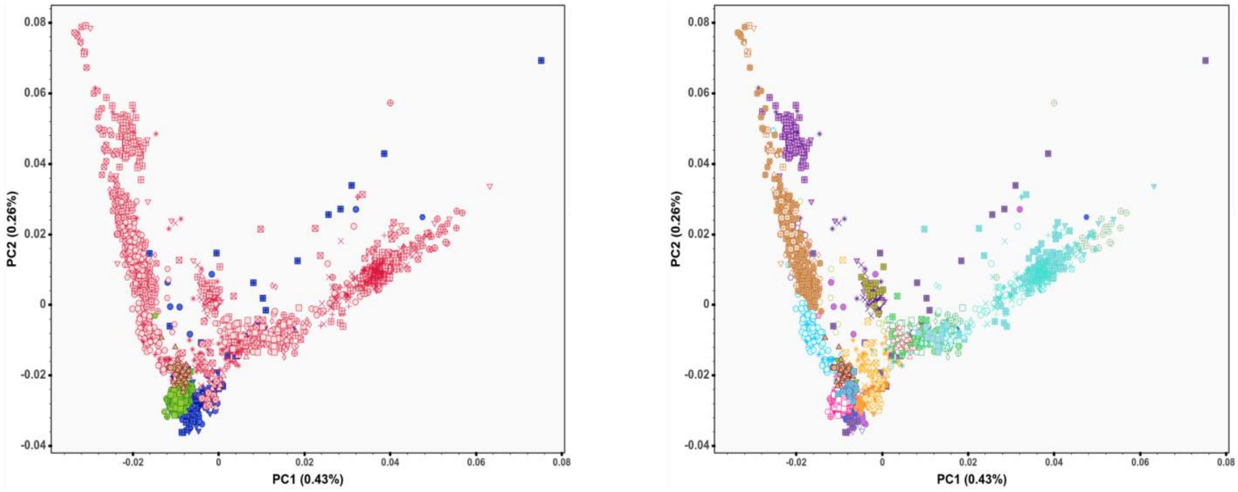

b

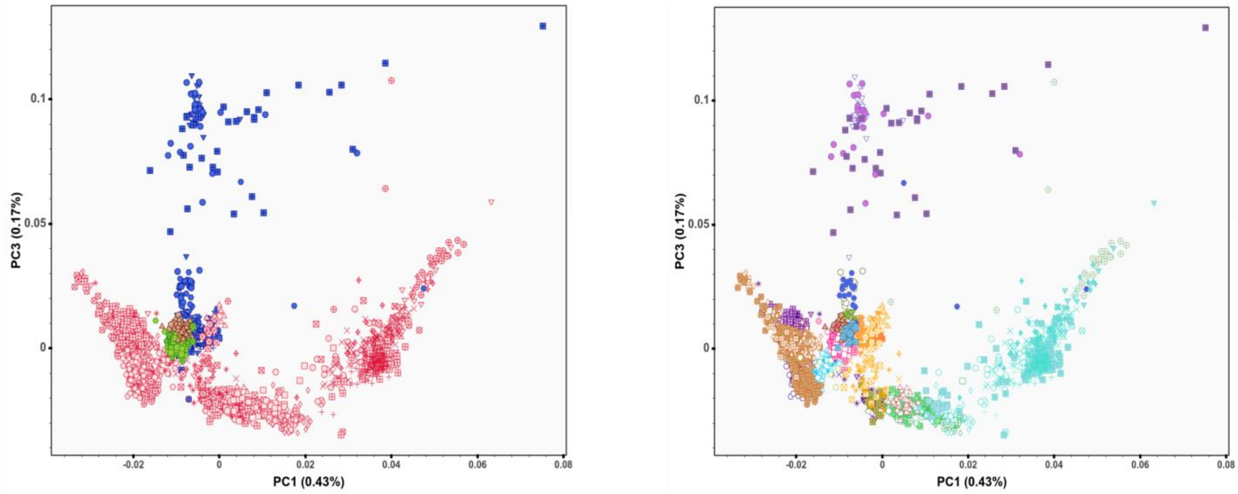

c

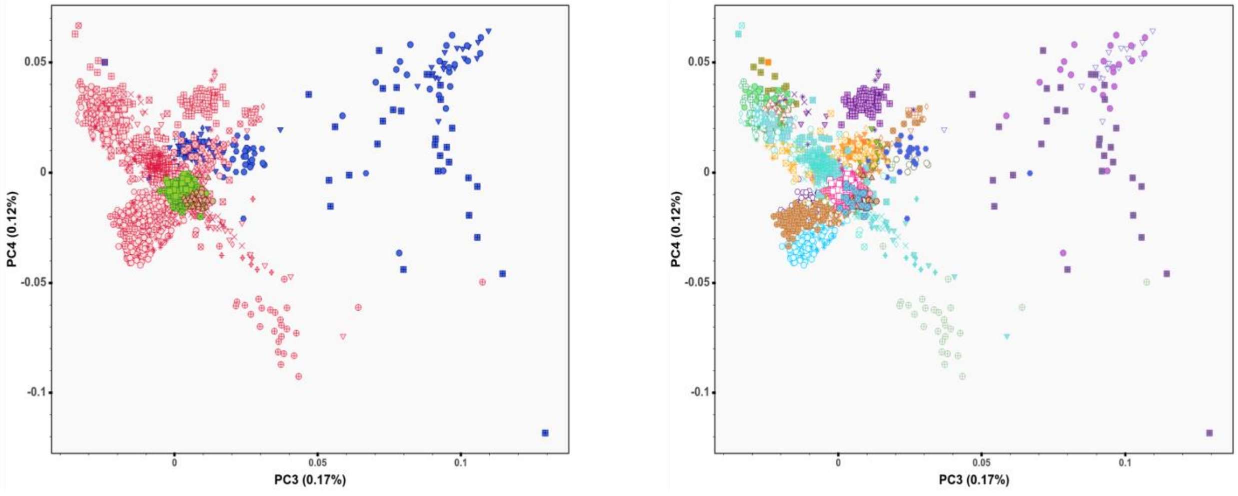

Supplementary Fig. 7 | PCA plots for only Bantu-speaking populations.

Panel figure showing PCA results of only individuals from the Only-BSP dataset (2,108 Bantu-speaking individuals; Supplementary Table 2), for PC projections obtained for each group (left column) and for each population (right column) between: **a**, PC1 vs PC2; **b**, PC1 vs PC3; and **c**, PC3 vs PC4. The legend is the same as in Supplementary Fig. 6c (left column) and Supplementary Fig. 6d (right column). The first ten PC projections were plotted as interactive plots at Github [113] (Suppl_Fig_7_PCA_Only-BSP_[Groups or Populations].html).

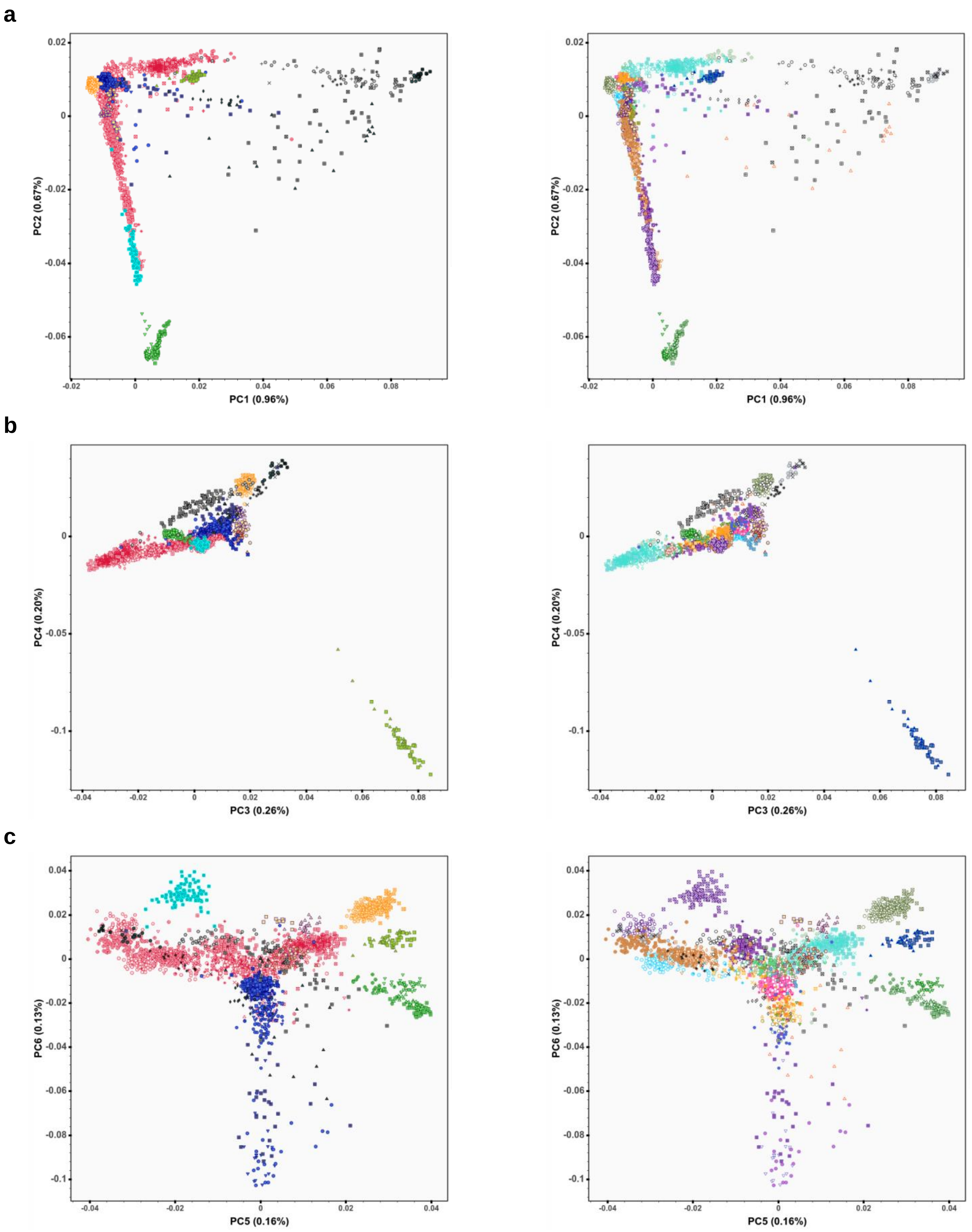

Supplementary Fig. 8 | PCA plots for selected sub-Saharan African populations.
Panel figure showing PCA results of selected sub-Saharan African populations that were included in the AfricanNeo dataset (Supplementary Table 2), for PC projections obtained for each group (left column) and for each population (right column) between: **a**, PC1 vs PC2; **b**, PC3 vs PC4; and **c**, PC5 vs PC6. To better visualize the results, interactive plots for the first ten PC projections are available at Github [113] (Suppl_Fig_8_PCA_SSA_[Groups or Populations].html).

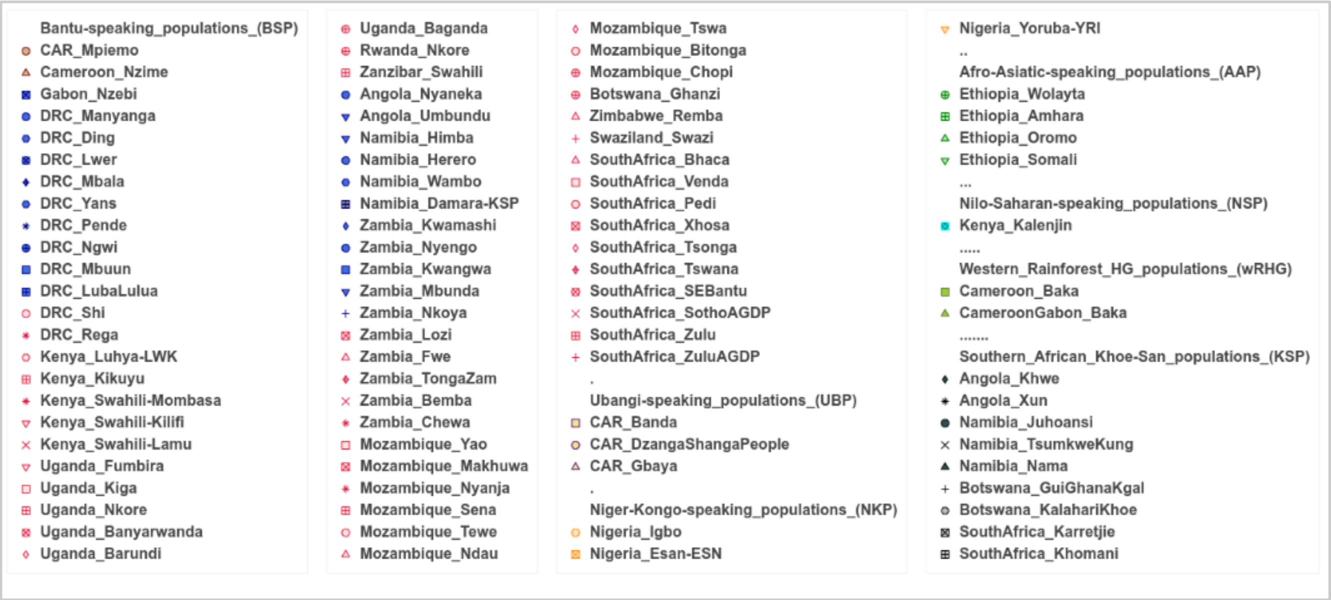

Legend for each group (left column) in panel Supplementary Fig. 8.

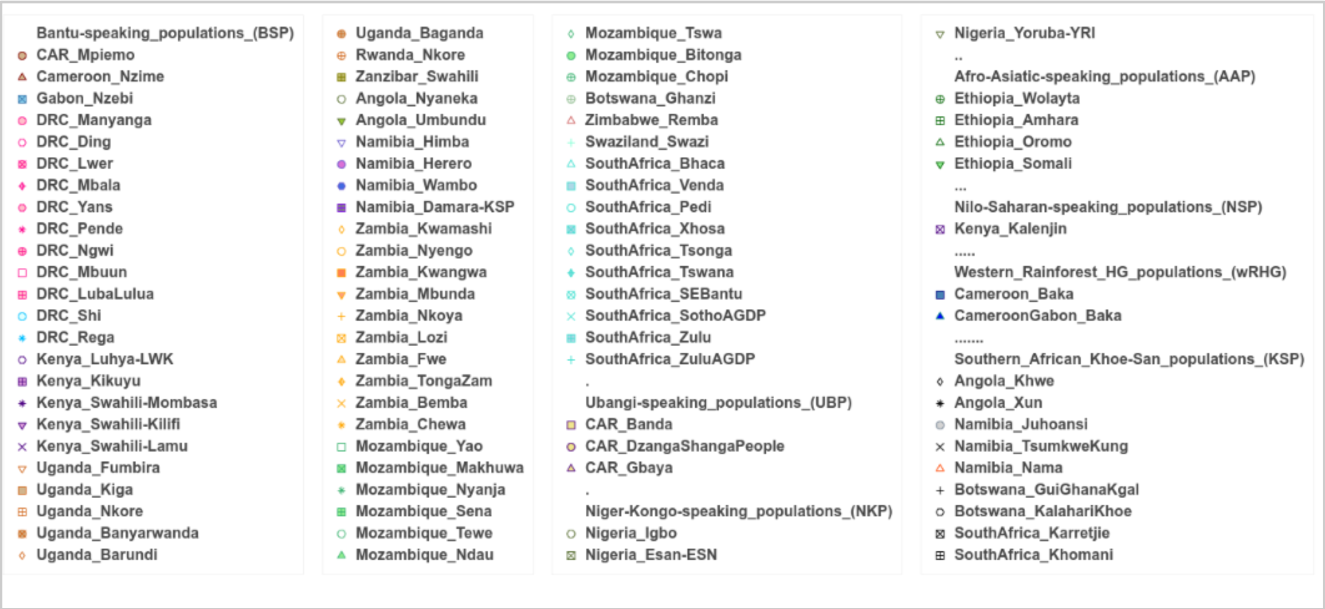

Legend for each population (right column) in panel Supplementary Fig. 8.

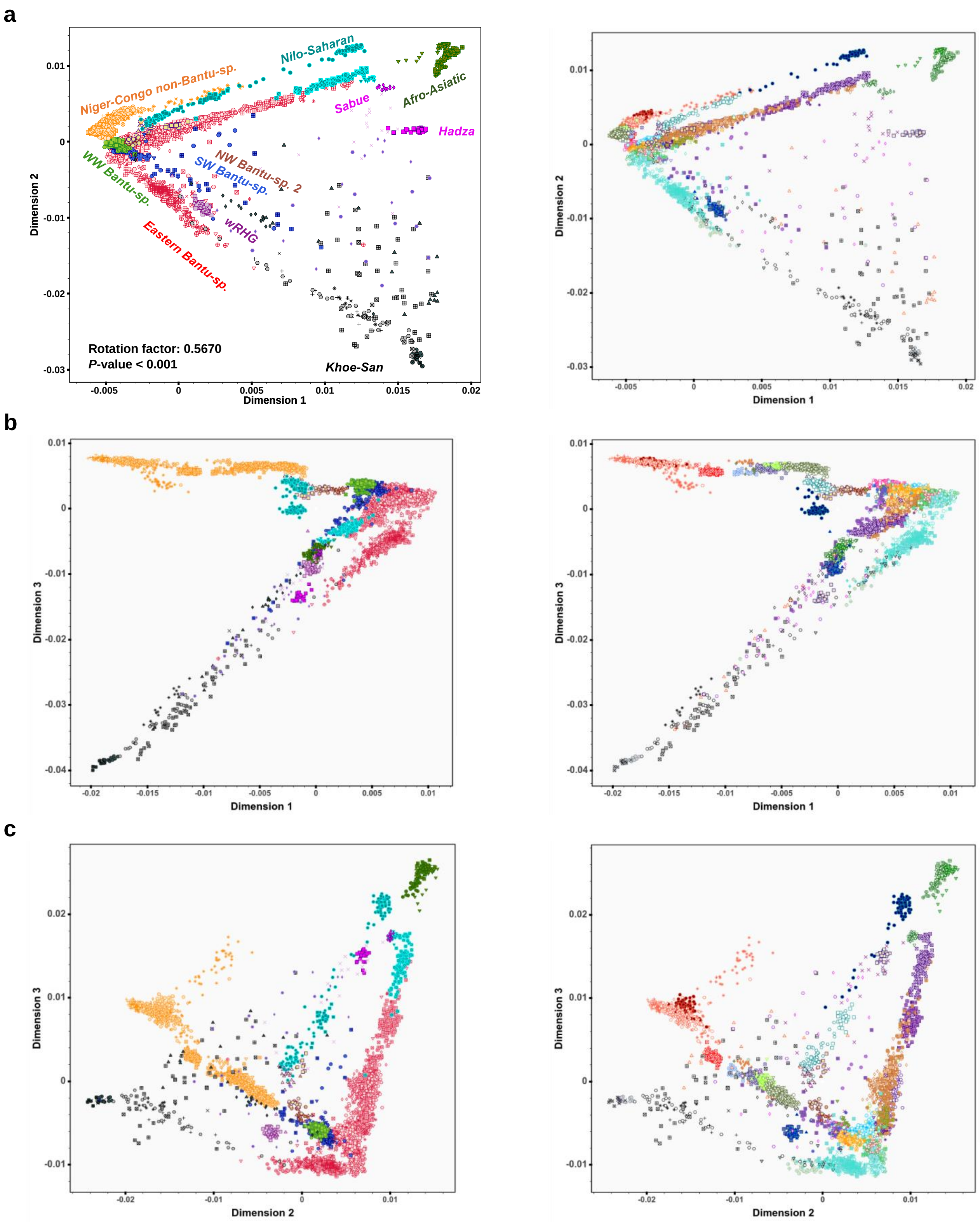

Supplementary Fig. 9 | Procrustes rotated PCA for the Only-African dataset.

Figure showing procrustes rotated PCA for sub-Saharan African populations (3,902 individuals) included in the Only-African dataset (Supplementary Fig. 4), for projections obtained for each group (left column) and for each population (right column) between: **a**, Dim1 vs Dim2; **b**, Dim1 vs Dim3; and **c**, Dim2 vs Dim3. Estimated correlations in a Procrustes rotation were: 0.5671, 0.7105 and 0.7676, respectively; all of them were significant (*P*-value < 0.001). Interactive plots are available at Github [113] (Suppl_Fig_9[a–c]_Procrustes_[Groups or Populations].html).

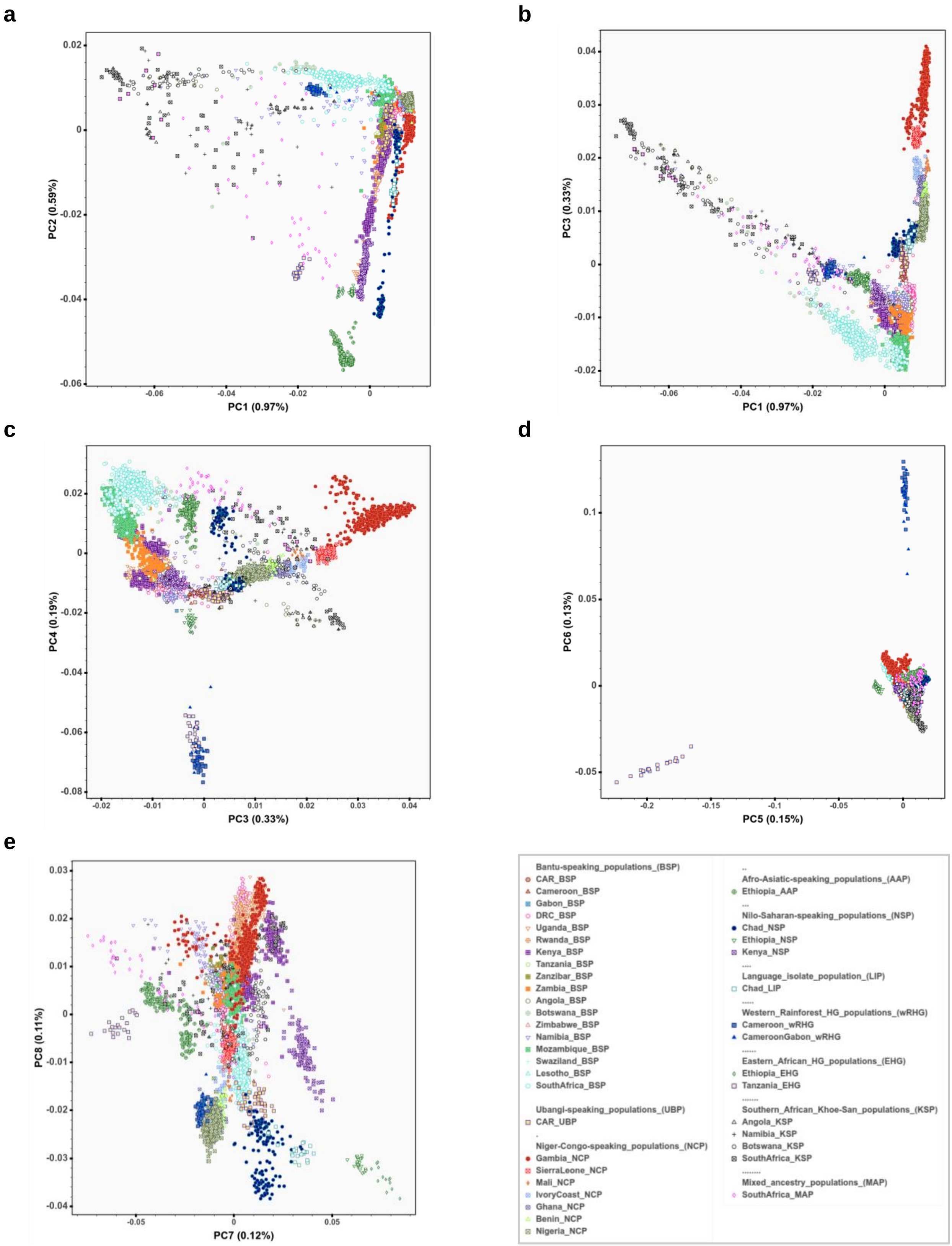

Supplementary Fig. 10 | PCA for all available sub-Saharan African populations.
Panel figure showing PCA results of all newly genotyped samples from Africa that were included in the Full-Genotyped dataset plus reference sub-Saharan African populations included in the AfricanNeo dataset (Supplementary Tables 1–2) after QC and merging. Panel figure showing each PC projection: **a**, PC1 vs PC2; **b**, PC1 vs PC3; **c**, PC3 vs PC4; **d**, PC5 vs PC6; and **e**, PC7 vs PC8. Interactive plots are available at Github [113] (Suppl_Fig_10_PCA_Full-Genotyped.html).

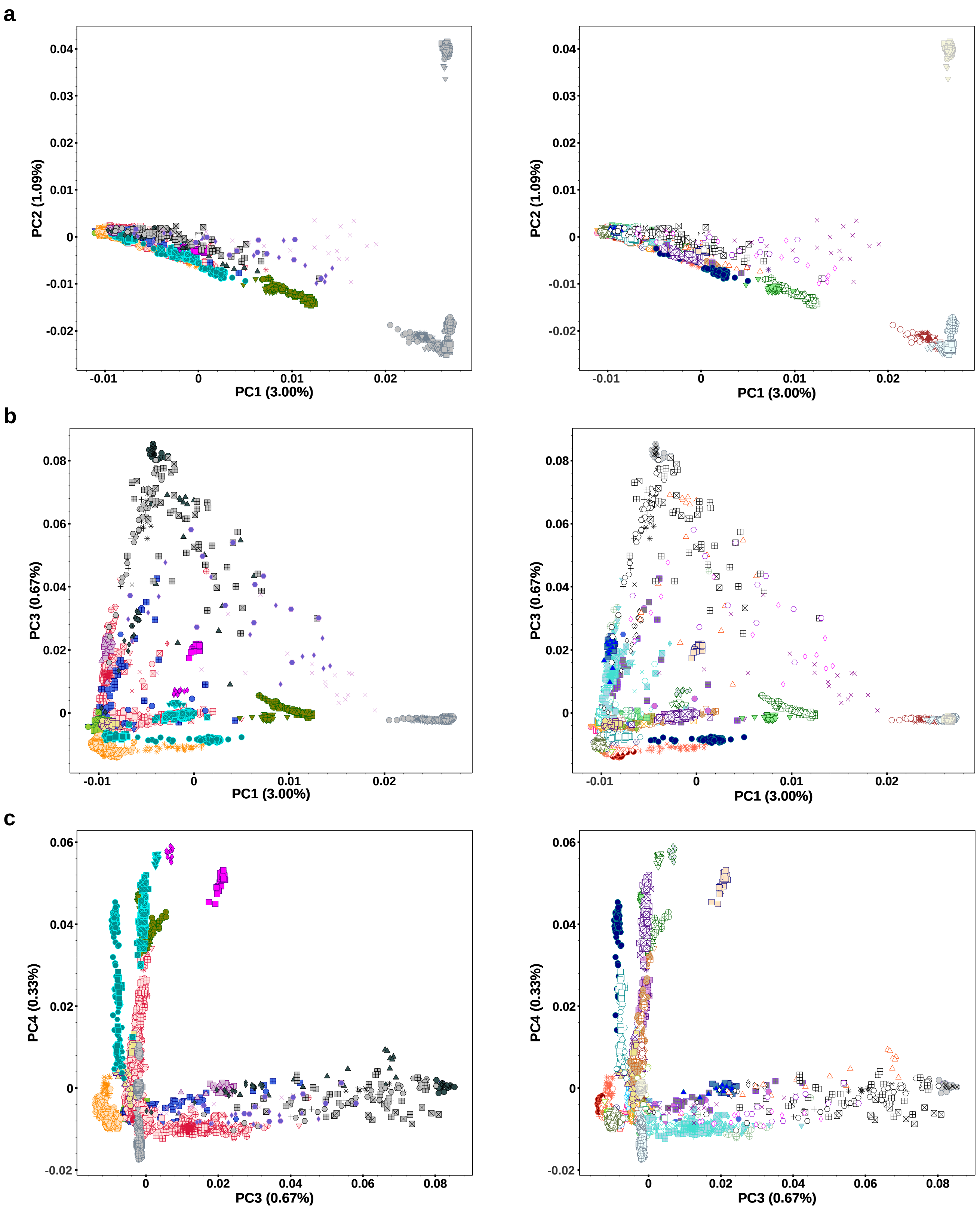

Supplementary Fig. 11 | PCA for each group included in the AfricanNeo dataset.
Panel figure showing PCA of worldwide groups included in the AfricanNeo dataset (4,950 individuals; in total from 124 populations; Supplementary Table 2), for PC projections obtained for each group (left column) and for each population (right column) between: **a**, PC1 vs PC2; **b**, PC1 vs PC3; and **c**, PC3 vs PC4. The legend of this figure is the same legend as in Supplementary Fig. 3. Interactive plots are available at Github [113] (Suppl_Fig_11_PCA_AfricanNeo_[Groups or Populations].html).

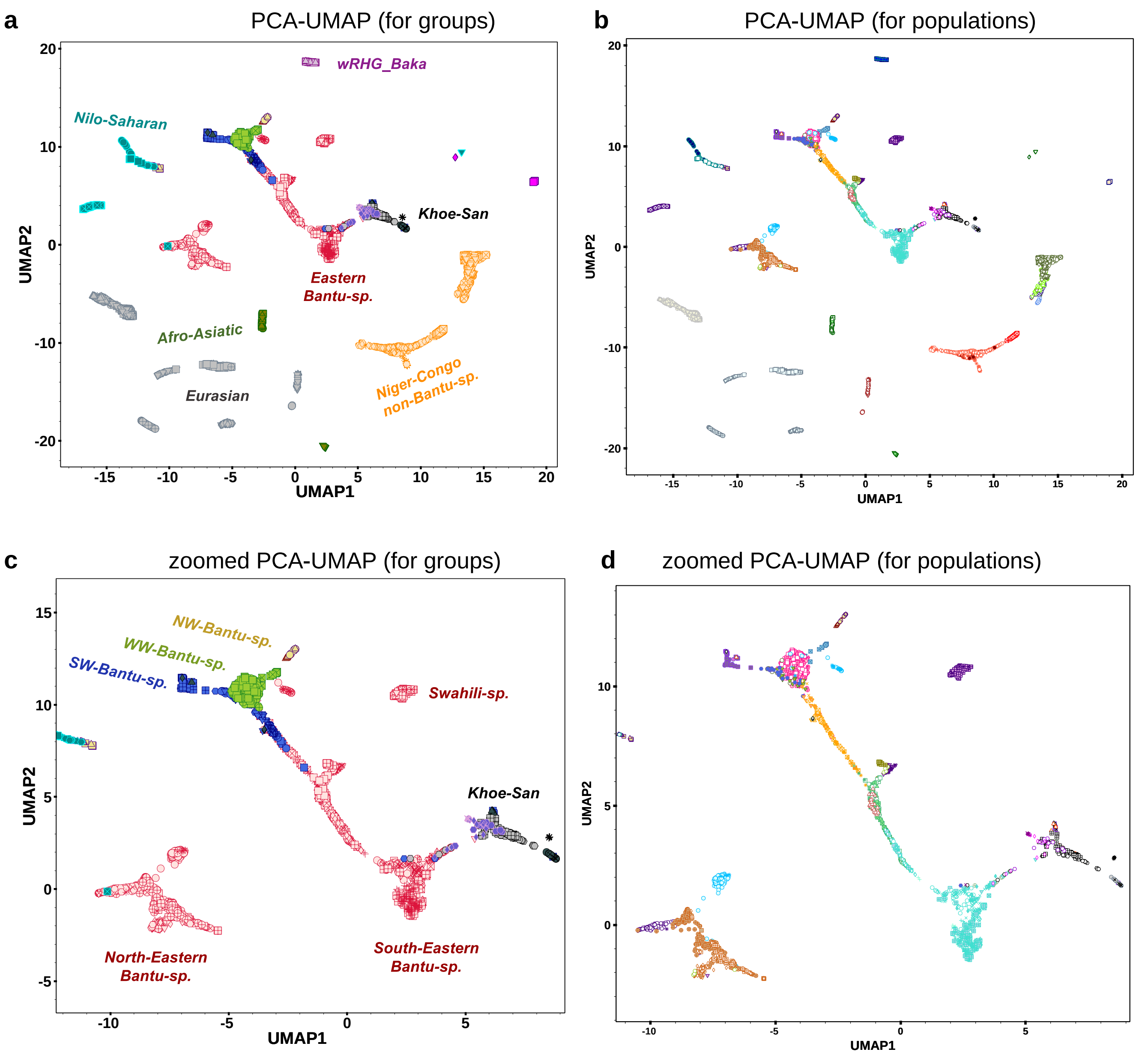

Supplementary Fig. 12 | PCA-UMAP approach applied for the AfricanNeo dataset.
Panel figure showing PCA-UMAP results of populations included in the AfricanNeo dataset using UMAP algorithm to combine the information of the first 10 PCs of the PCA (Supplementary Fig. 11). This panel figure highlights results for **a**, each group listed in Supplementary Fig. 3a and **b**, each population listed in Supplementary Fig. 3b. To better see the results for the studied BSP, we zoom in on each plot highlighting **c**, each Bantu-speaking group and **d**, each Bantu-speaking population. The legend of this figure is the same legend as in Supplementary Fig. 3. To better visualize the result, interactive plots are available at Github [113] (Suppl_Fig_12[a–b]_PCA-UMAP.html).

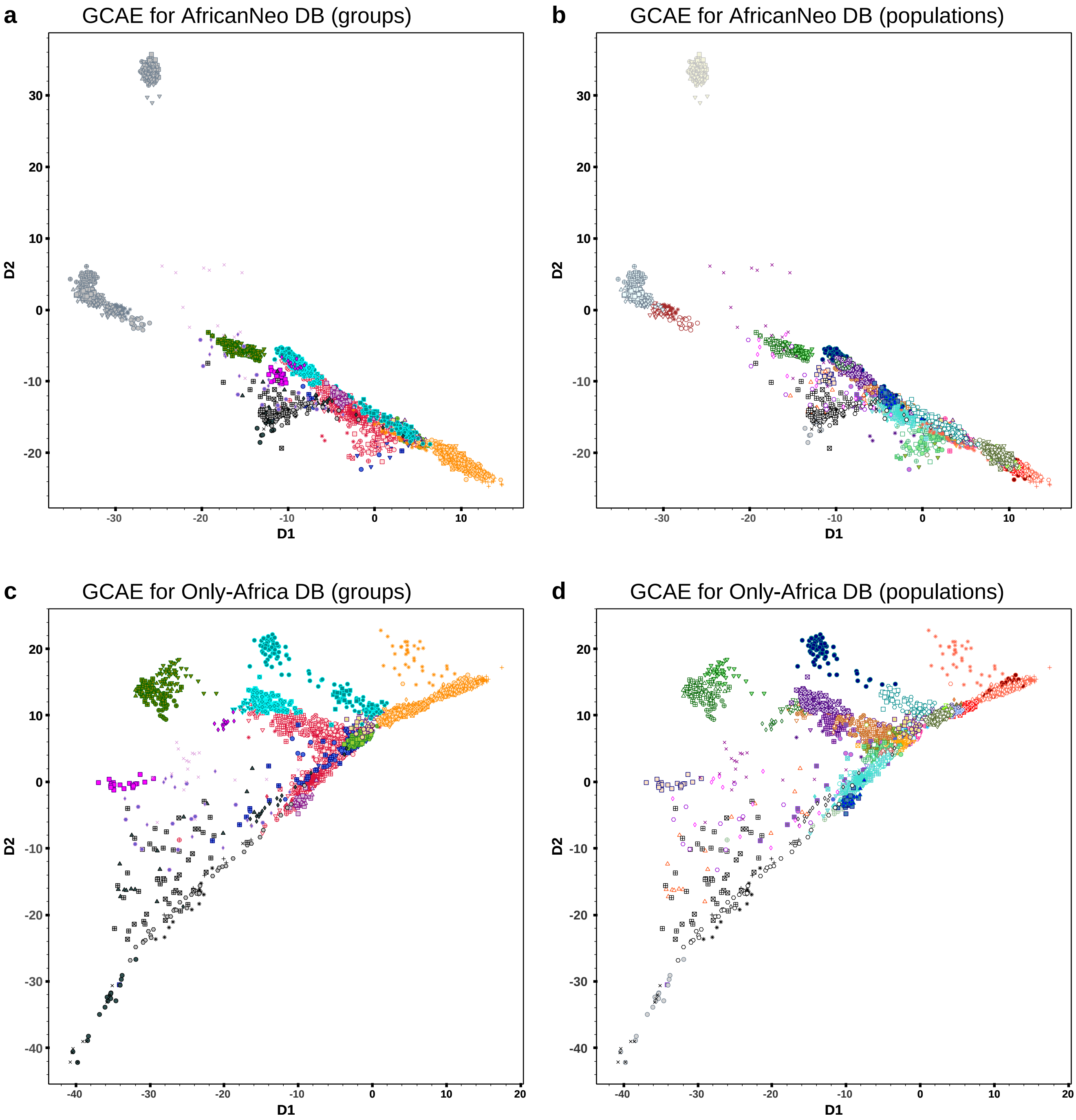

Supplementary Fig. 13 | GCAE approach for the AfricanNeo and Only-African datasets.
Panel figure showing GCAE results of all African and Eurasian populations included in the AfricanNeo dataset (4,950 individuals; in total from 124 populations; Supplementary Table 2) were estimated using Genotype Convolutional Autoencoder (GCAE) approach, a deep learning framework for dimensionality reduction. This panel figure highlights **a**, each group listed in Supplementary Fig. 3a and **b**, each population listed in Supplementary Fig. 3b. Figure also shows GCAE results of all populations included in the Only-African dataset (Supplementary Table 2), **c**, for each group and **d**, for each population. The legend of this figure is the same legend as in Supplementary Fig. 3. To better visualize the results of each group and each population, interactive plots are available at Github [113] (Suppl_Fig_13[a–d]_GCAE.html).

3.3. Unsupervised clustering analyses

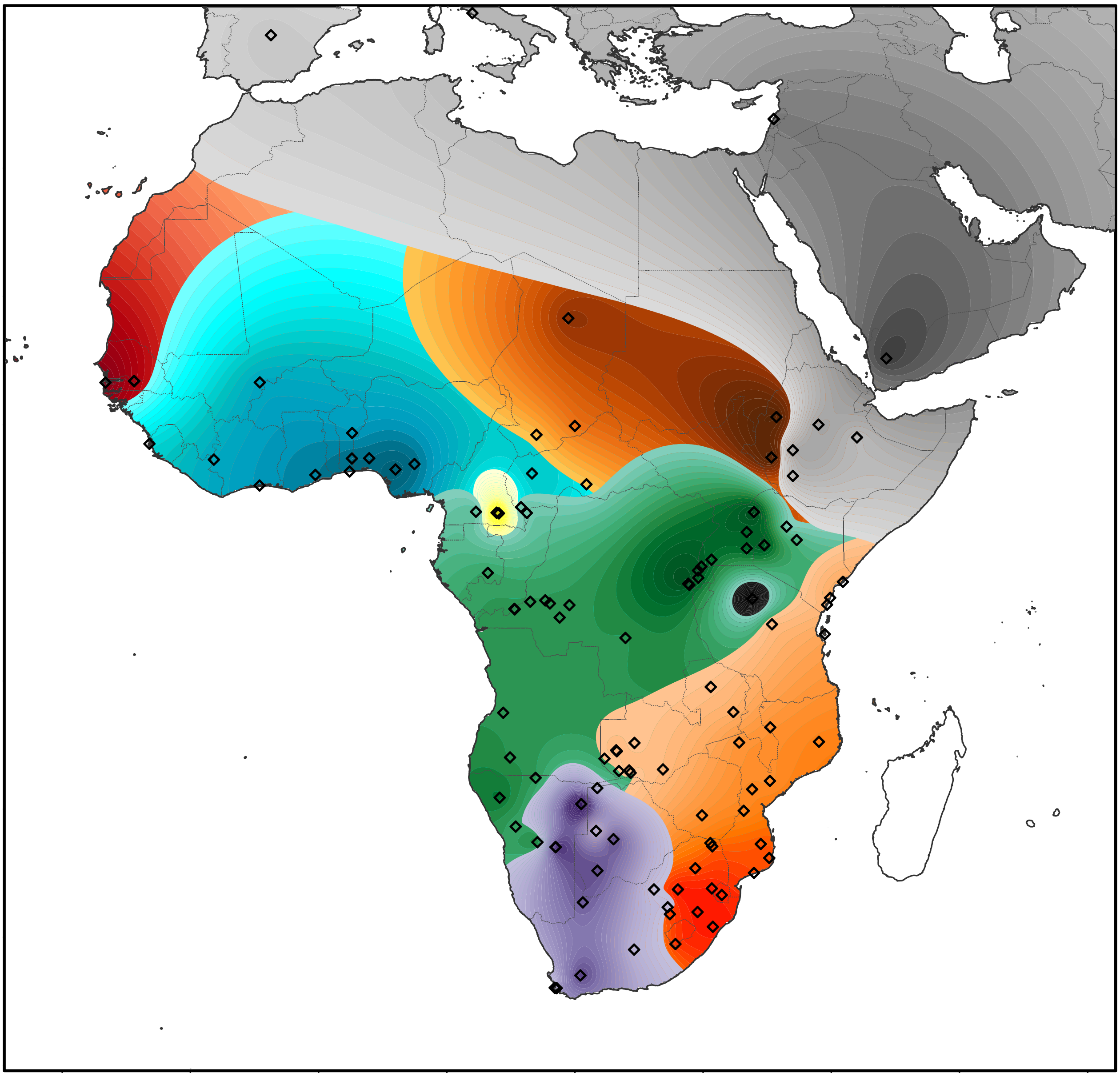

Supplementary Fig. 14 | Contour map of ADMIXTURE at K=12.
Panel figure showing a contour map overlapping unsupervised ADMIXTURE results at K=12 created using the Kriging method for all the populations included in the AfricanNeo dataset (Supplementary Fig. 3). The geographical distributions of average admixture proportions were represented for nine ancestries found in high frequencies in African populations. Ancestry components with values under 25% are not represented in this figure, while all the estimated values (from 0% to 100%) for each represented ancestry component are shown in Extended Data Fig. 3. Grid layers for each ancestry were created using Surfer Golden Software (© Golden Software, Inc 2023).

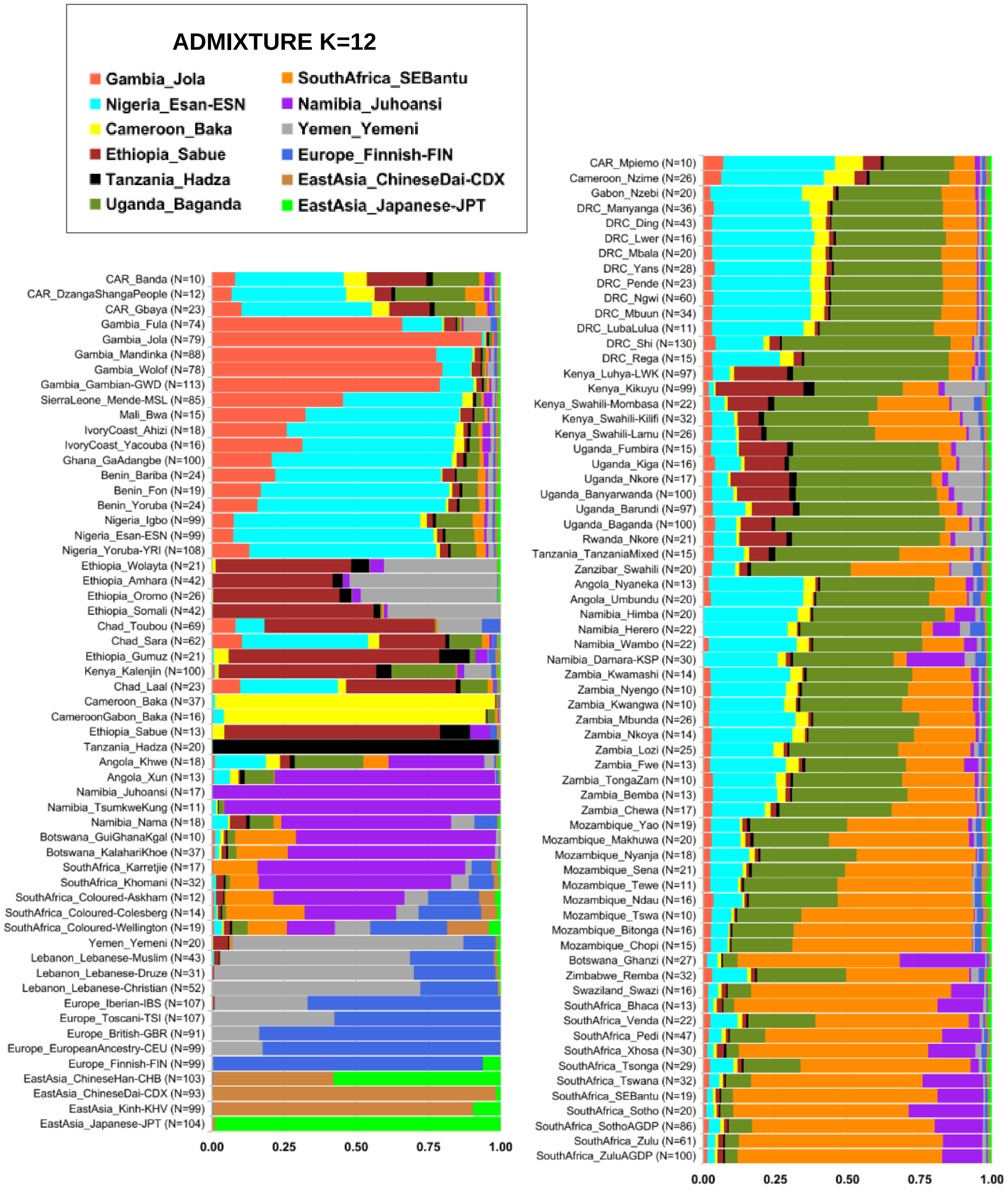

Supplementary Fig. 15 | Bar plots of ADMIXTURE results for K=12.
Panel figure showing average admixture results estimated using unsupervised ADMIXTURE plot at K=12 for all the populations included in the AfricanNeo dataset.

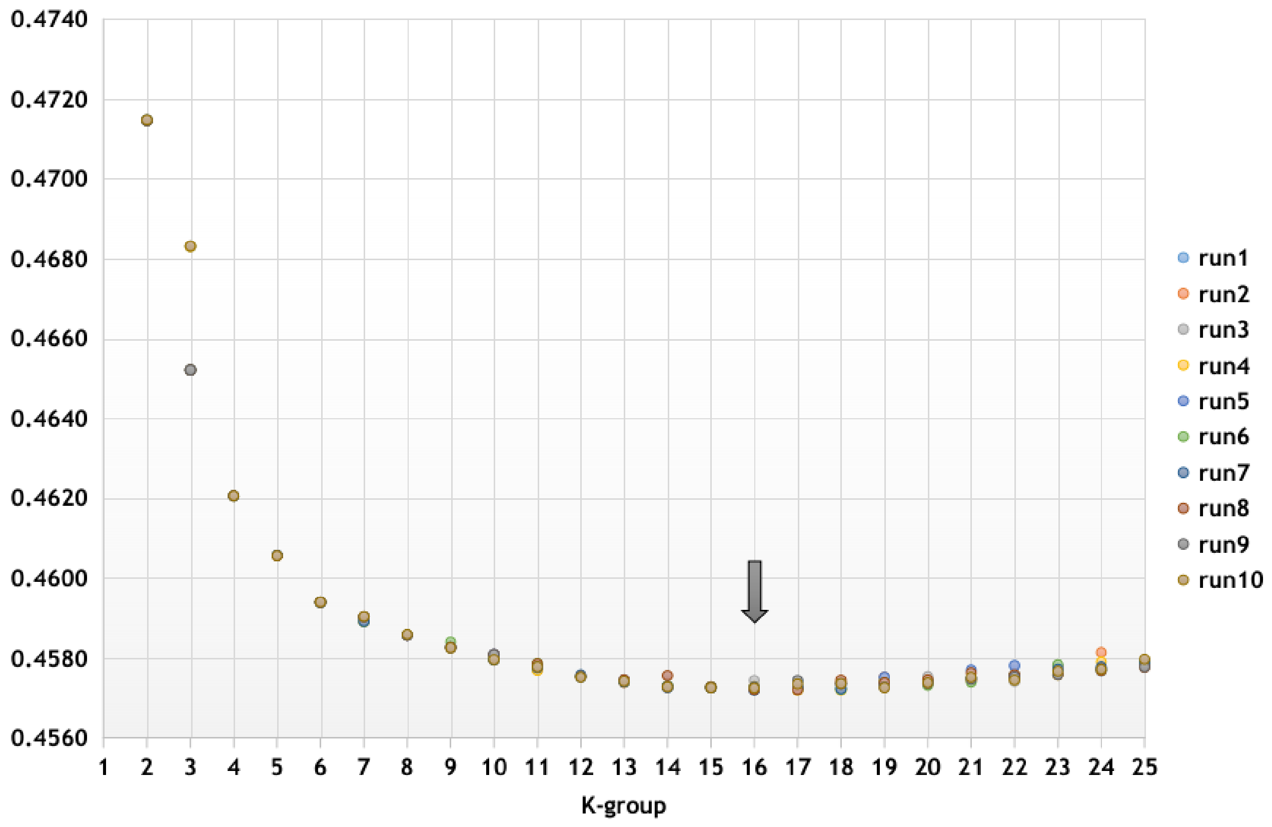

Supplementary Fig. 16 | Cross-validation test from the ADMIXTURE analyses for each K-group. Figure showing cross-validation test from the ADMIXTURE analyses from K=2 to K=25, after using 10 independent runs with a random seed for each K-group. We highlighted with a black arrow the K-group with the lowest CV value (K=16). Each run from each K-group is one dot, and the dots from the same run have the same colour.

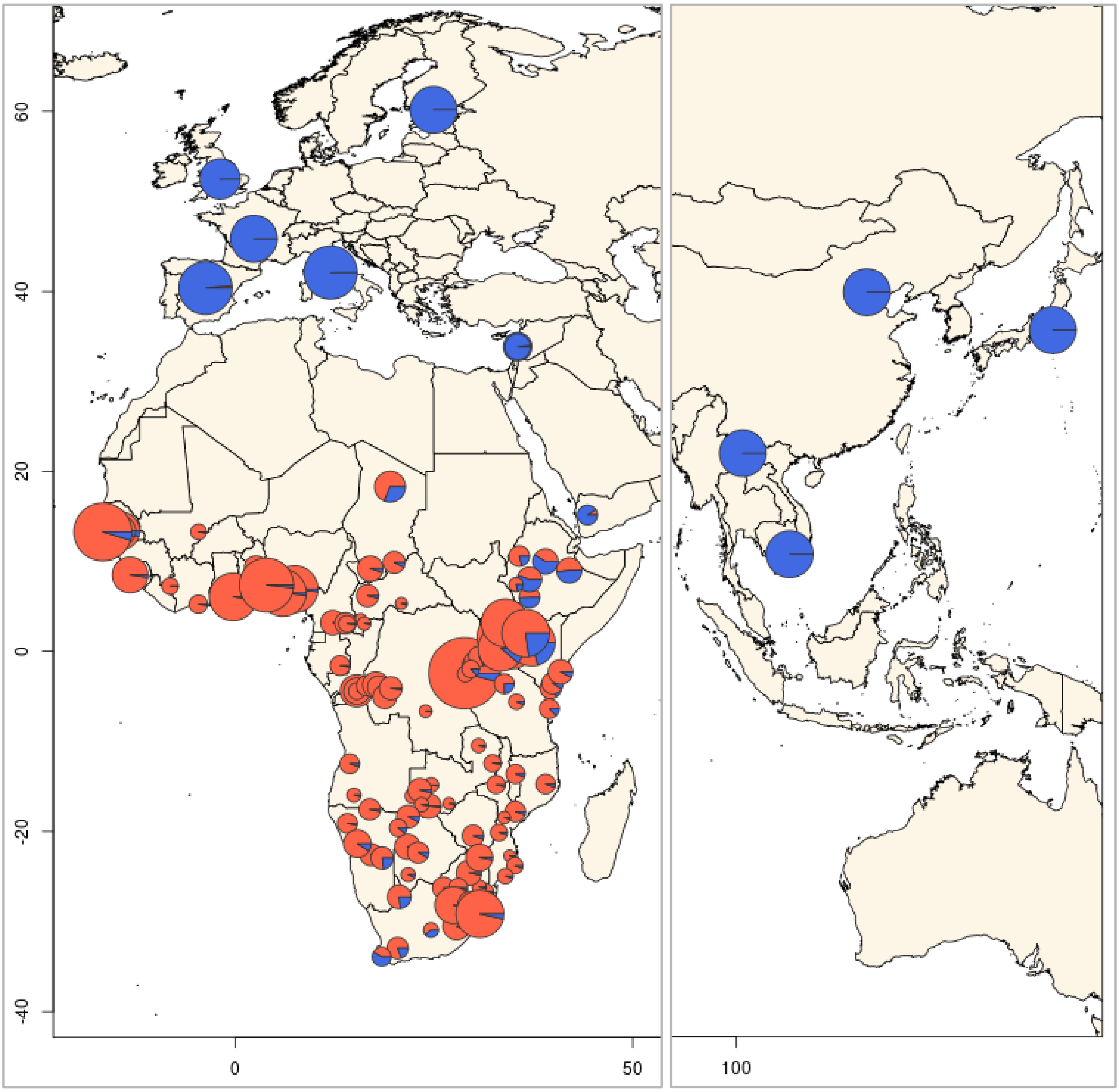

Supplementary Fig. 17 | Pie charts of ADMIXTURE results for K=2.

Panel figure showing unsupervised ADMIXTURE plot at K=2 for all the populations included in the AfricanNeo dataset. Results highlight the African-related component (in red) and the Eurasian-related component (in blue). The size of the pie charts is in relation to the sample size of each studied population. To better visualize the plot, central Asia was removed from the map.

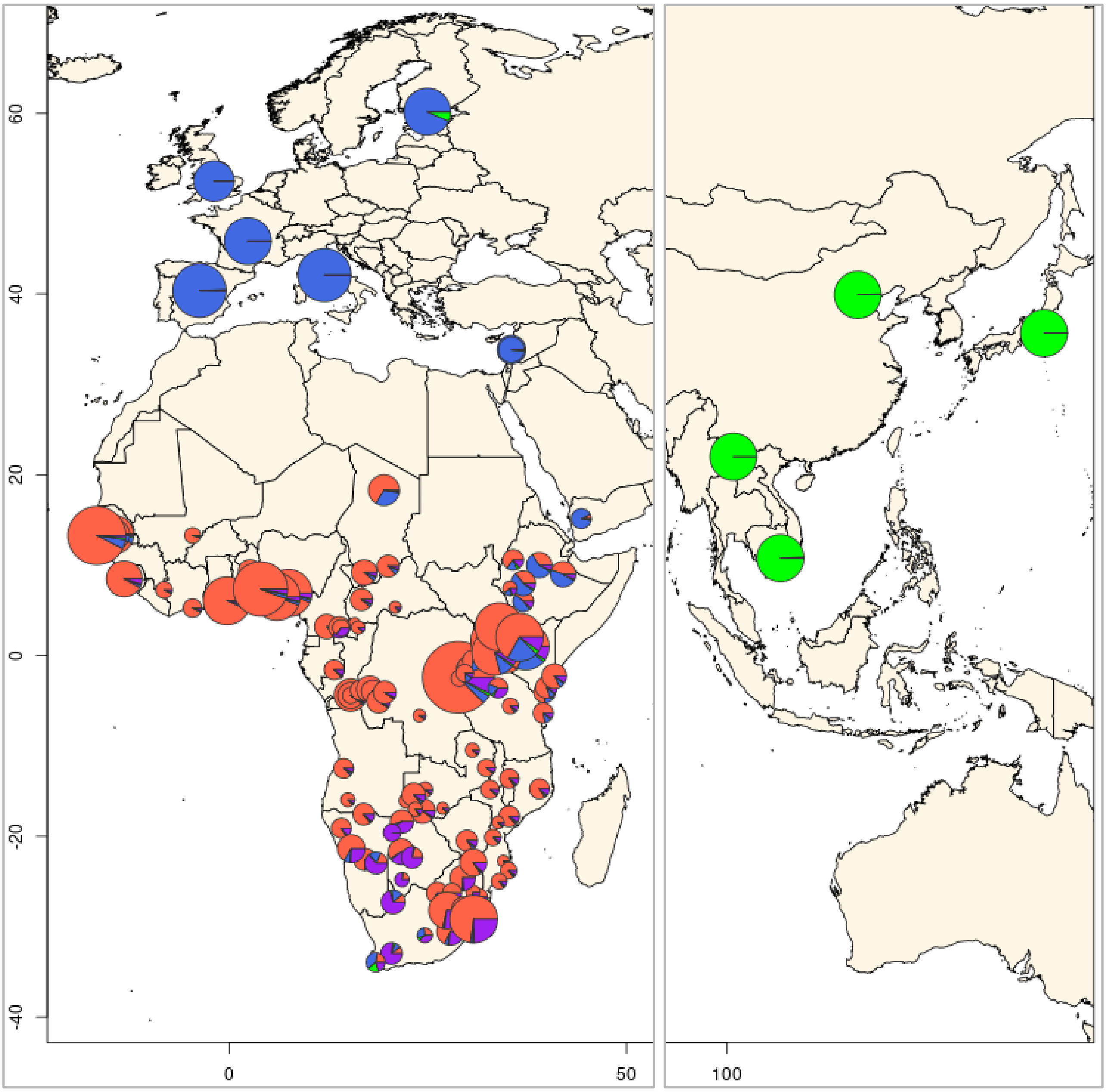

Supplementary Fig. 18 | Pie charts of ADMIXTURE results for K=4.
Panel figure showing unsupervised ADMIXTURE results at K=4 for all the populations included in the AfricanNeo dataset. Average admixture proportions of each African population are presented in Supplementary Table 3. Results highlight the African-related component (in red), the hunter-gatherer-related component (in purple), the European-related component (in blue), and the East Asian-related component (in green; as expected in the Coloured populations in South Africa and the Finnish population). The size of the pie charts is in relation to the sample size of each studied population. To better visualize the plot, central Asia was removed from the map. Further details of the results of each individual in each population were included in Supplementary Fig. 25.

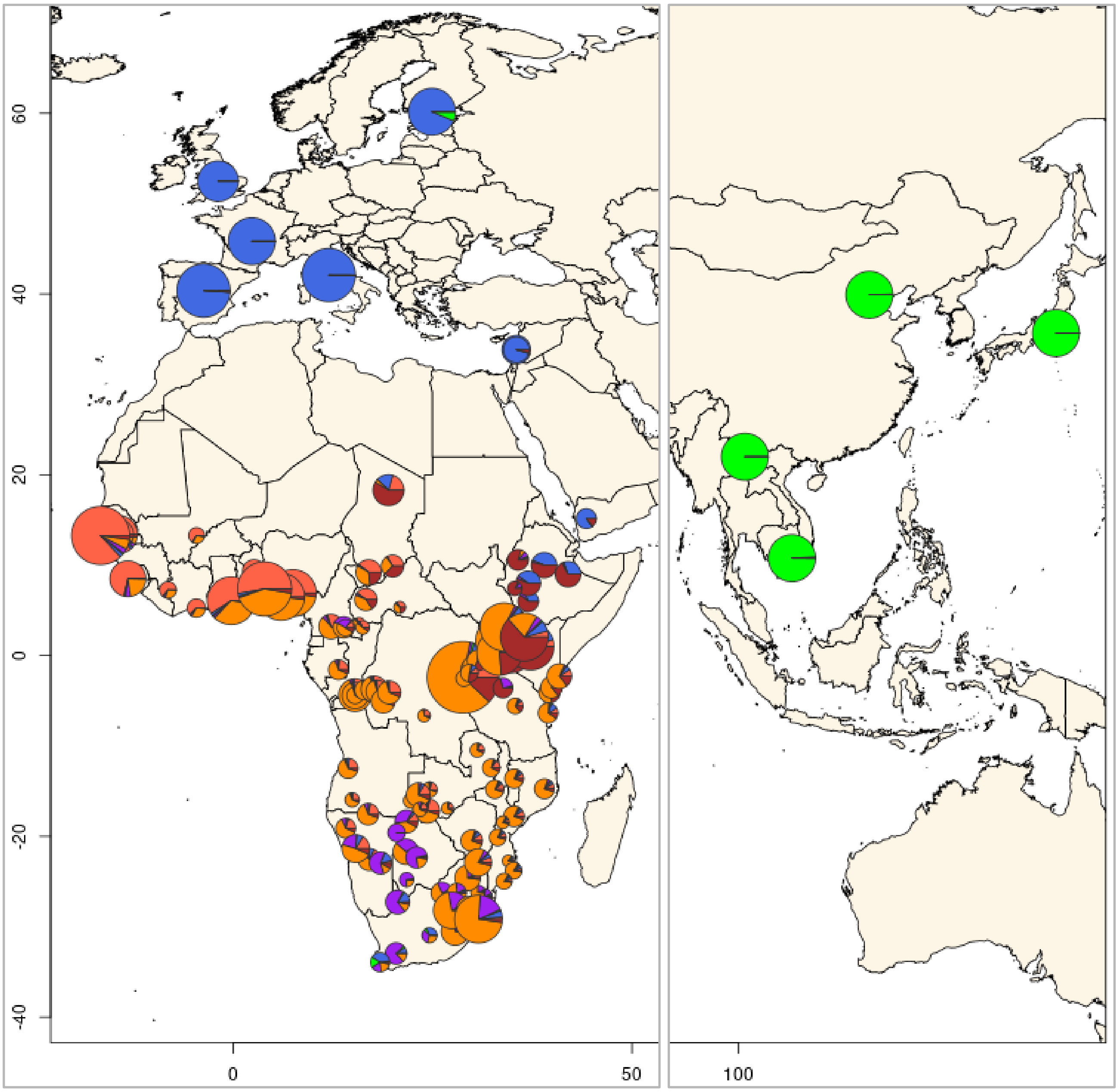

Supplementary Fig. 19 | Pie charts of ADMIXTURE results for K=6.

Panel figure showing unsupervised ADMIXTURE plot at K=6 for all the populations included in the AfricanNeo dataset. The size of the pie charts is in relation to the sample size of each studied population. To better understand the results, ADMIXTURE results at K=6 were also plotted the results in ternary diagrams, see Supplementary Fig. 24. To better visualize the plot, central Asia was removed from the map.

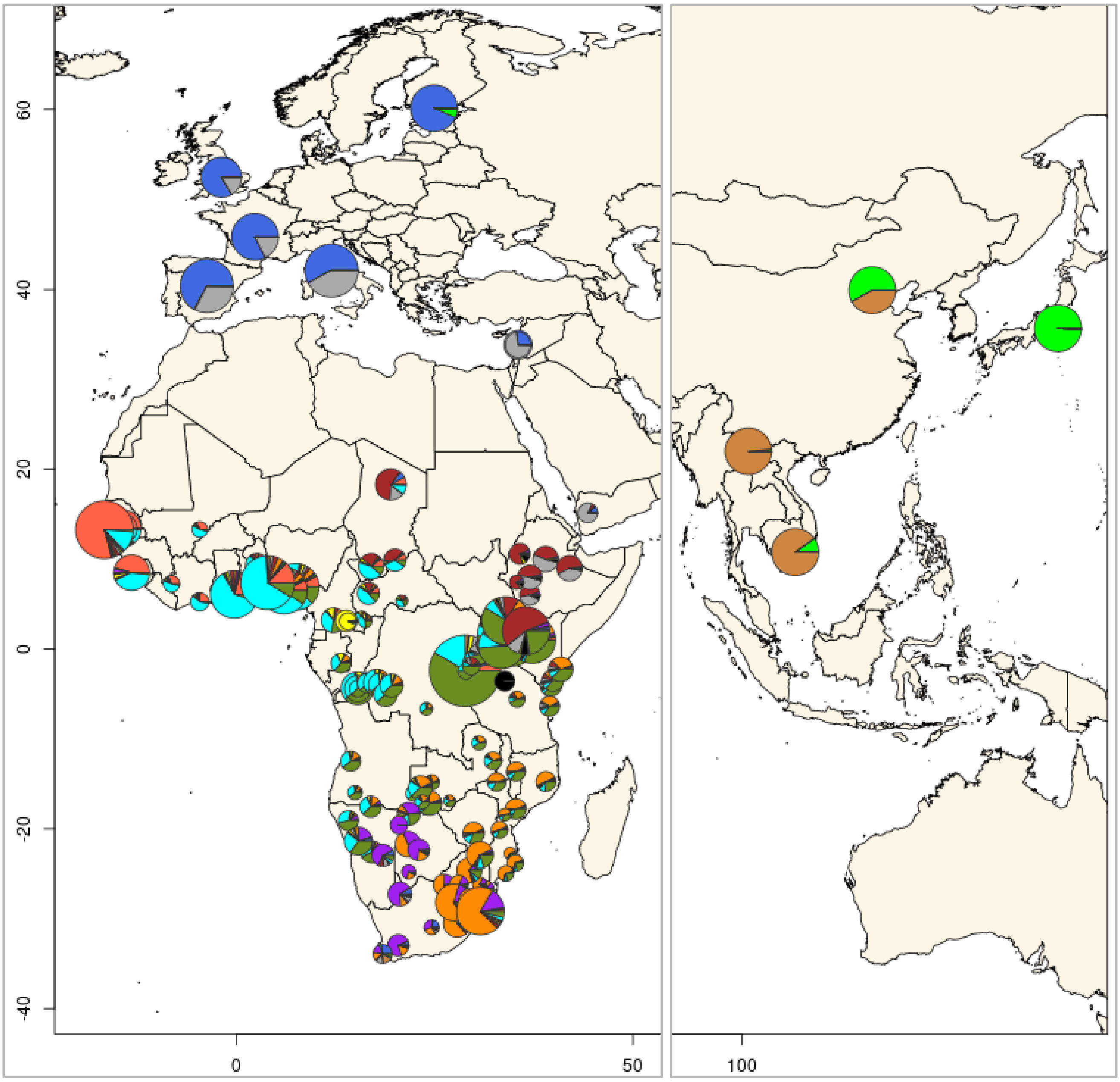

Supplementary Fig. 20 | Pie charts of ADMIXTURE results for K=12.

Panel figure showing unsupervised ADMIXTURE plot at K=12 for all the populations included in the AfricanNeo dataset. The size of the pie charts is in relation to the sample size of each studied population. The highest values for each component were found in the following populations: red component in Jola from Gambia; cyan component in Esan from Nigeria; yellow component in Baka from Cameroon and Gabon; brown component in Sabue from Ethiopia; dark green component in Baganda from Uganda; orange component in SE Bantu speakers from South Africa; purple component in Ju'hoansi from Namibia; grey component in Yemeni from Yemen; blue component in European populations; light brown component in Chinese (Dai-CDX) population; and light green component Japanese population. To better visualize the plot, central Asia was removed from the map.

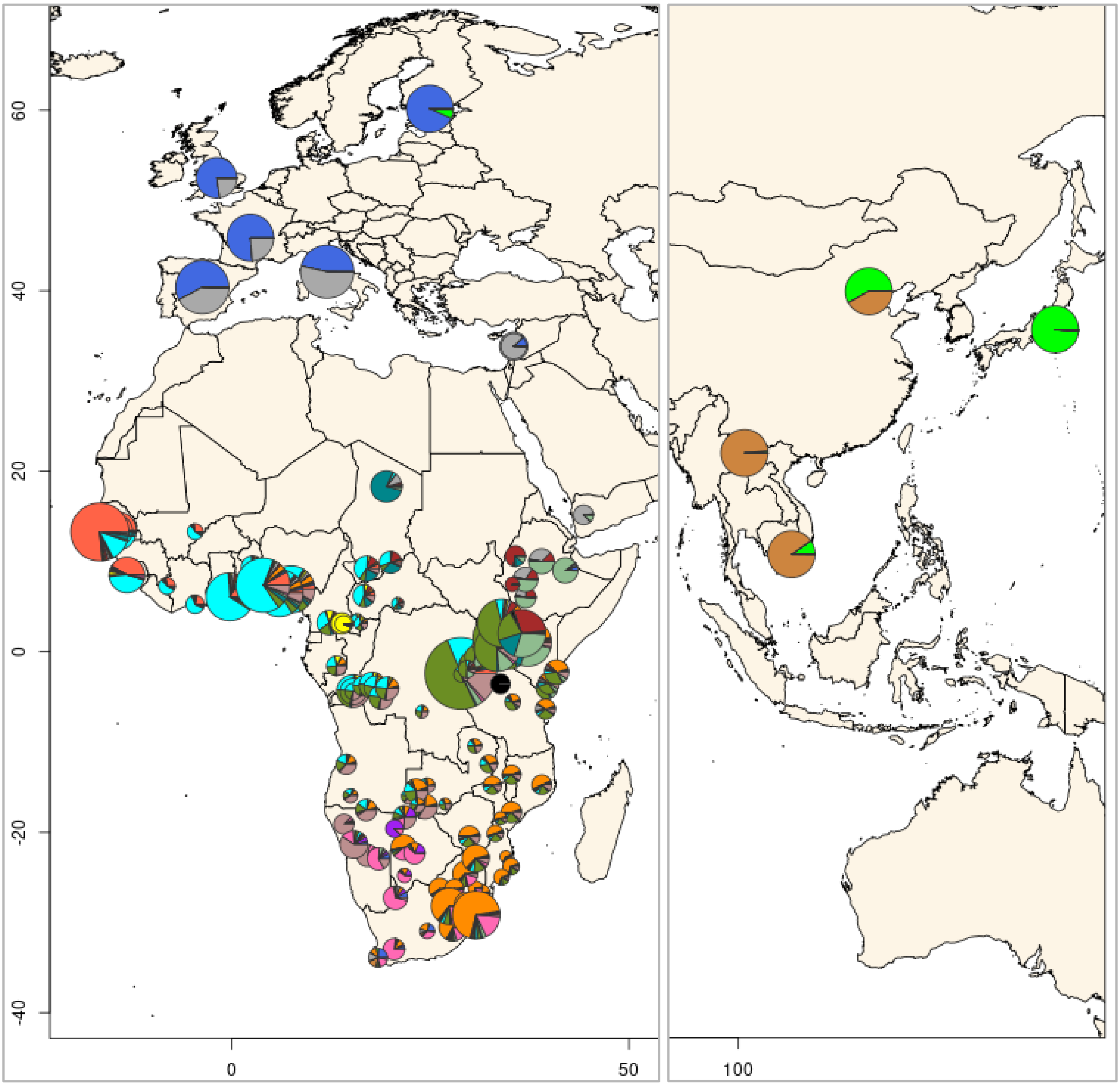

Supplementary Fig. 21 | Pie charts of ADMIXTURE results for K=16.
Unsupervised ADMIXTURE plot at K=16 for all the populations included in the AfricanNeo dataset. Further details of the results of each individual in each population were included in Supplementary Fig. 23 and Supplementary Table 4. The size of the pie charts is in relation to the sample size of each studied population. The highest values for each component were found in the following populations: red component in Jola from Gambia; cyan component in Esan from Nigeria; yellow component in Baka from Cameroon and Gabon; brown component in Sabue from Ethiopia; dark green component in Baganda from Uganda; orange component in SE Bantu speakers from South Africa; purple component in Ju'hoansi from Namibia; teal component in Nilo-Saharan speakers from Chad; green component in Afro-Asiatic speakers from Ethiopia; North and South Khoe-San speakers (rosybrown and pink components, respectively); grey component in Yemeni from Yemen; blue component in European populations; light brown component in Chinese (Dai-CDX) population; and light green component Japanese population. To better visualize the plot, central Asia was removed from the map.

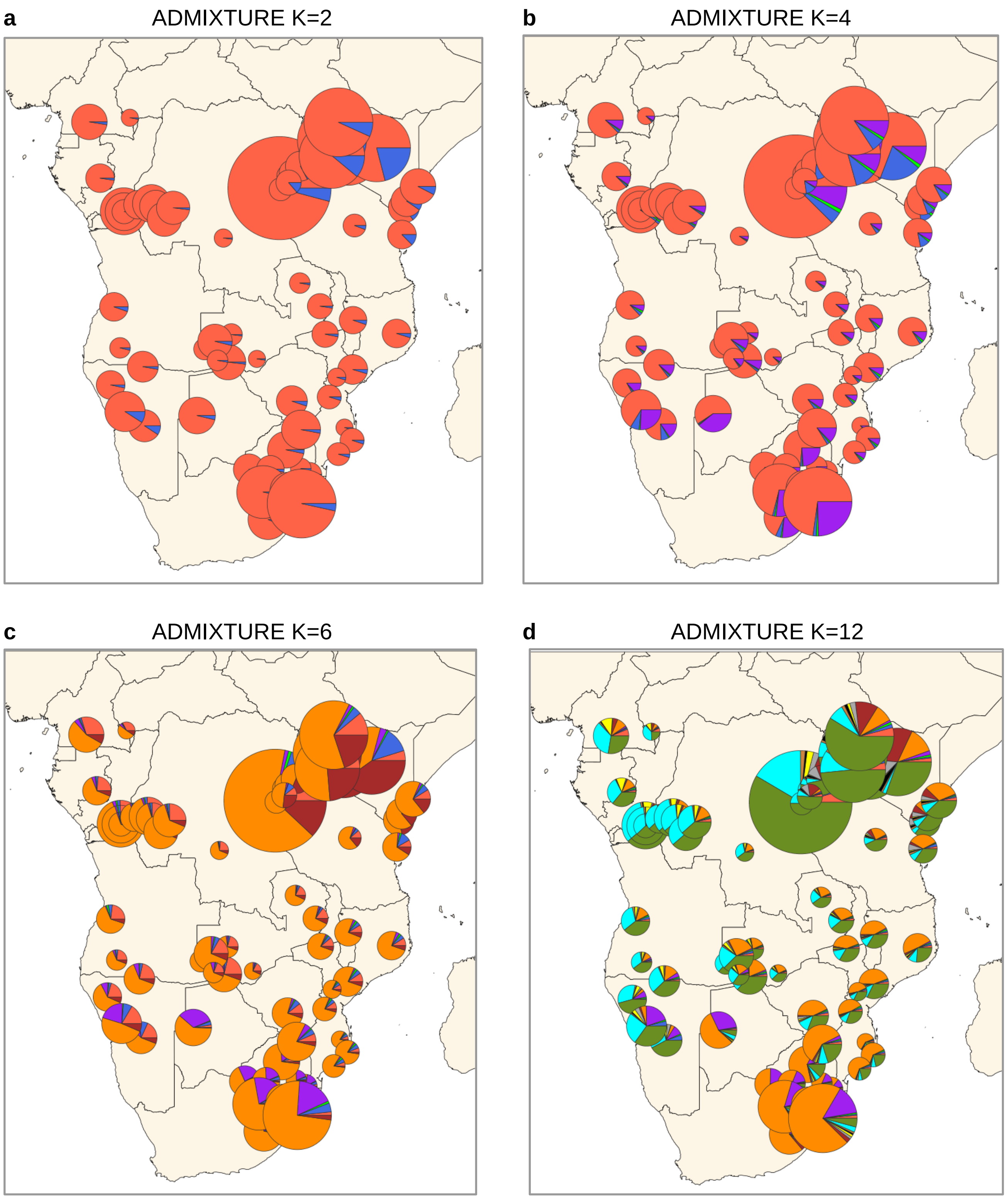

Supplementary Fig. 22 | Pie charts of ADMIXTURE results for only studied BSP.
Panel figure showing unsupervised ADMIXTURE results only for studied BSP for **a,** K=2; **b**, K=4; **c**, K=6; and **d**, K=12. Results for BSP and all comparative populations were included for each K-group separately in figures above (Supplementary Fig.s 17–20).

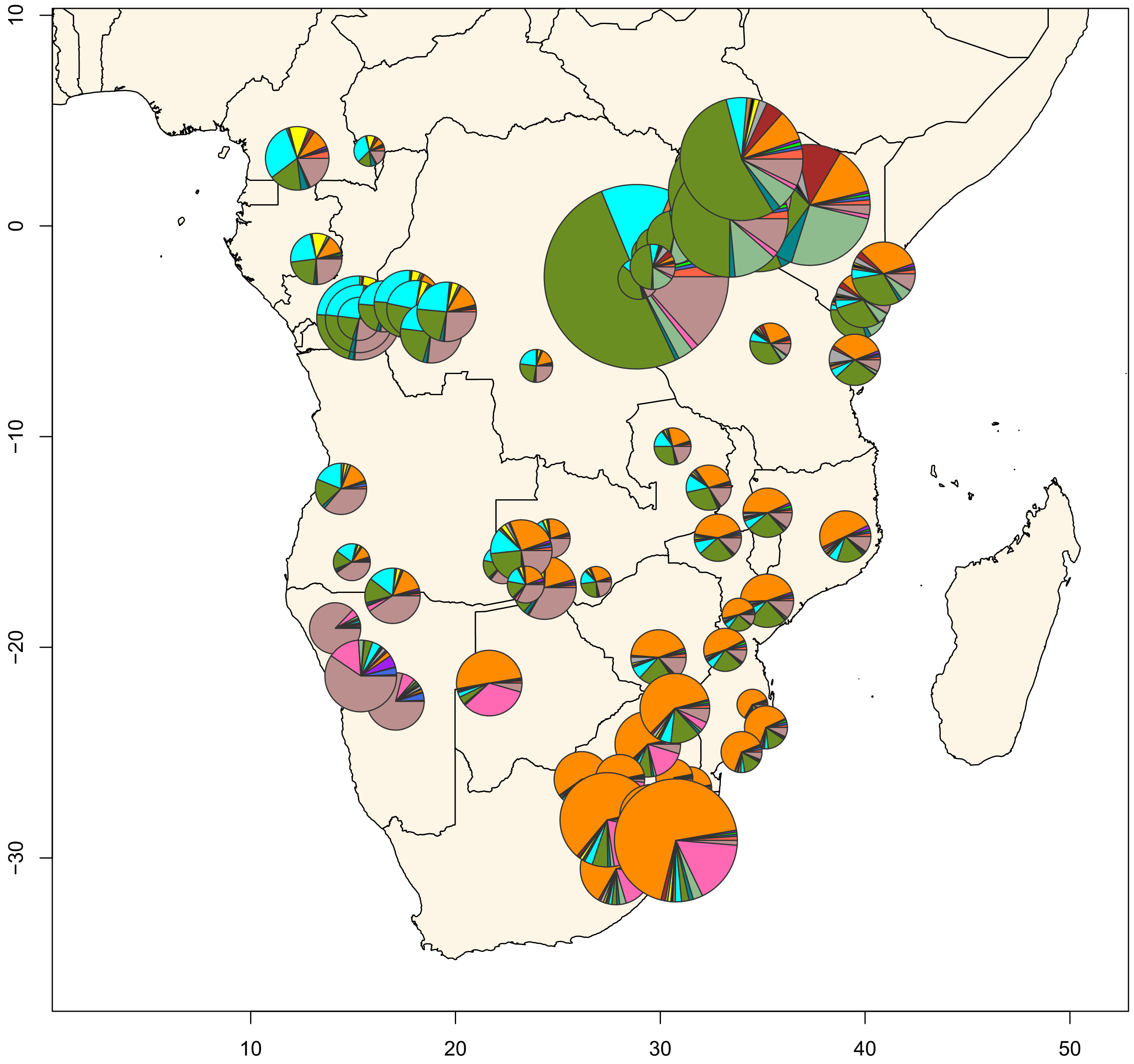

Supplementary Fig. 23 | Pie charts of ADMIXTURE results for K=16 only for BSP.
Panel figure showing unsupervised ADMIXTURE plot at K=16 only for BSP included in the AfricanNeo dataset, and comparative populations were not included in this plot. Results for BSP and all comparative populations were included in Supplementary Fig. 21.

a

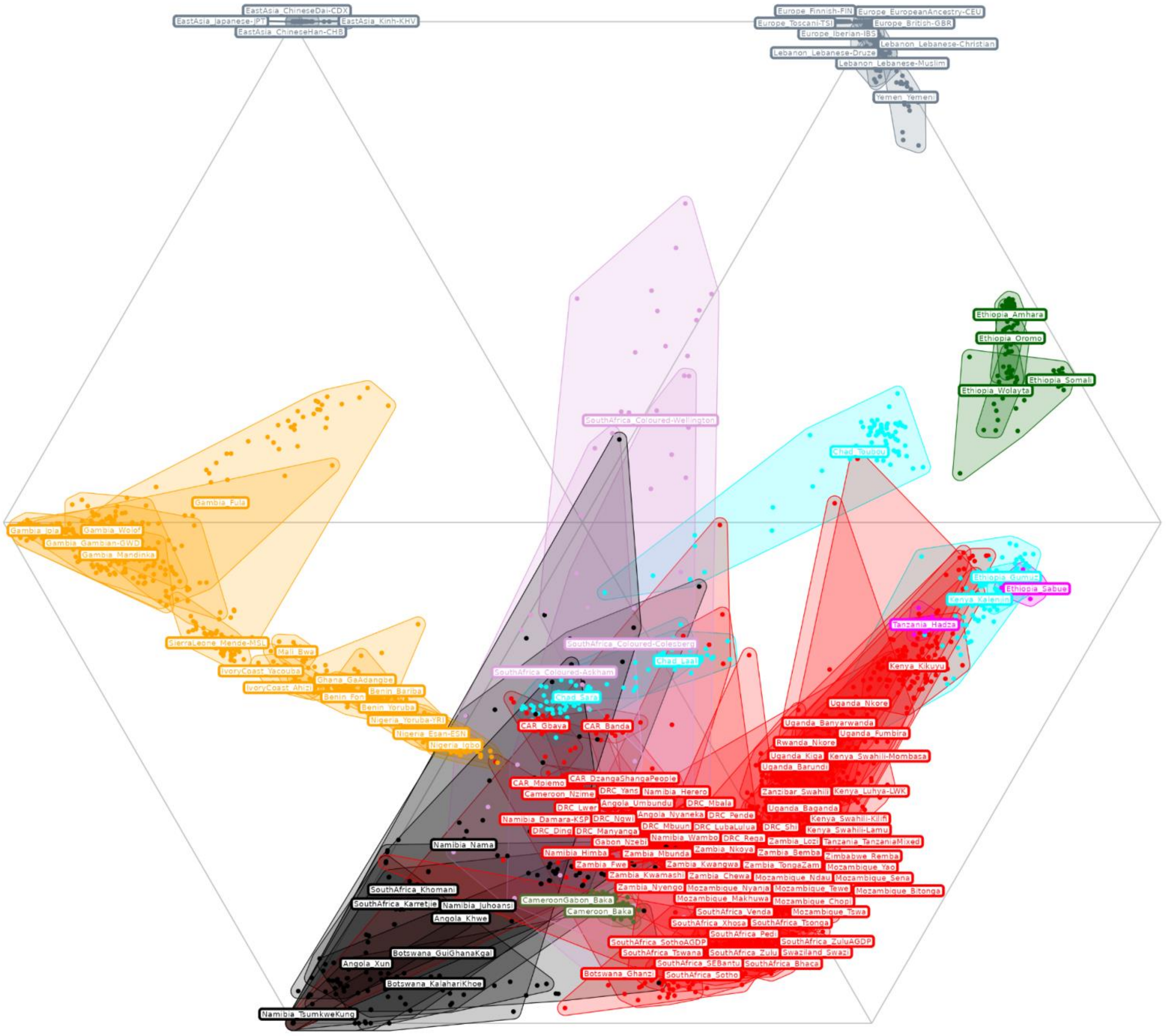

EastAsia_ChineseDai-CDX
EastAsia_Japanese-JPT
EastAsia_Kinh-KHV
EastAsia_ChineseHan-CHB
Europe_Finnish-FIN
Europe_EuropeanAncestry-CEU
Europe_Toscani-TSI
Europe_British-GBR
Europe_Iberian-IBS
Lebanon_Lebanese-Christian
Lebanon_Lebanese-Druze
Lebanon_Lebanese-Muslim
Yemen_Yemeni
Ethiopia_Amhara
Ethiopia_Oromo
Ethiopia_Somali
Ethiopia_Wolayta
SouthAfrica_Coloured-Wellington
Chad_Toubou
Gambia_Fula
Gambia_Jola
Gambia_Wolof
Gambia_Gambian-GWD
Gambia_Mandinka
Ethiopia_Gumuz
Ethiopia_Sabue
Kenya_Kalenjin
Tanzania_Hadza
SierraLeone_Mende-MSL
Mali_Bwa
SouthAfrica_Coloured-Colesberg
Chad_Laal
IvoryCoast_Yacouba
SouthAfrica_Coloured-Askham
Ghana_GaAdangbe
IvoryCoast_Ahizi
Benin_Bariba
Benin_Fon
Benin_Yoruba
Chad_Sara
Kenya_Kikuyu
Nigeria_Yoruba-YRI
Nigeria_Esan-ESN
Nigeria_Igbo
CAR_Gbaya
CAR_Banda
Uganda_Nkore
Uganda_Banyarwanda
Uganda_Fumbira
Rwanda_Nkore
Uganda_Kiga
Kenya_Swahili-Mombasa
Uganda_Barundi
CAR_Mpiemo
CAR_DzangaShangaPeople
Cameroon_Nzime
DRC_Yans
Namibia_Herero
Zanzibar_Swahili
Kenya_Luhya-LWK
DRC_Lwer
Angola_Umbundu
DRC_Mbala
Uganda_Baganda
Namibia_Damara-KSP
DRC_Ngwi
Angola_Nyaneka
DRC_Pende
Kenya_Swahili-Kilifi
DRC_Ding
DRC_Manyanga
DRC_Mbuun
DRC_LubaLulua
DRC_Shi
Kenya_Swahili-Lamu
Gabon_Nzebi
Namibia_Wambo
DRC_Rega
Zambia_Lozi
Tanzania_TanzaniaMixed
Namibia_Nama
Namibia_Himba
Zambia_Mbunda
Zambia_Nkoya
Zambia_Bemba
Zimbabwe_Remba
Zambia_Fwe
Zambia_Kwangwa
Zambia_TongaZam
Mozambique_Yao
Zambia_Kwamashi
Zambia_Chewa
Mozambique_Ndau
Mozambique_Sena
SouthAfrica_Khomani
Zambia_Nyengo
Mozambique_Nyanja
Mozambique_Tewe
Mozambique_Bitonga
SouthAfrica_Karretjie
Namibia_Juhoansi
CameroonGabon_Baka
Mozambique_Makhuwa
Mozambique_Chopi
Angola_Khwe
Cameroon_Baka
SouthAfrica_Venda
Mozambique_Tswa
SouthAfrica_Xhosa
SouthAfrica_Tsonga
SouthAfrica_Pedi
SouthAfrica_SothoAGDP
SouthAfrica_ZuluAGDP
Botswana_GuiGhanaKgal
SouthAfrica_Tswana
SouthAfrica_Zulu
Swaziland_Swazi
Angola_Xun
SouthAfrica_SEBantu
SouthAfrica_Bhaca
Botswana_Ghanzi
SouthAfrica_Sotho
Botswana_KalahariKhoe
Namibia_TsumkweKung

b

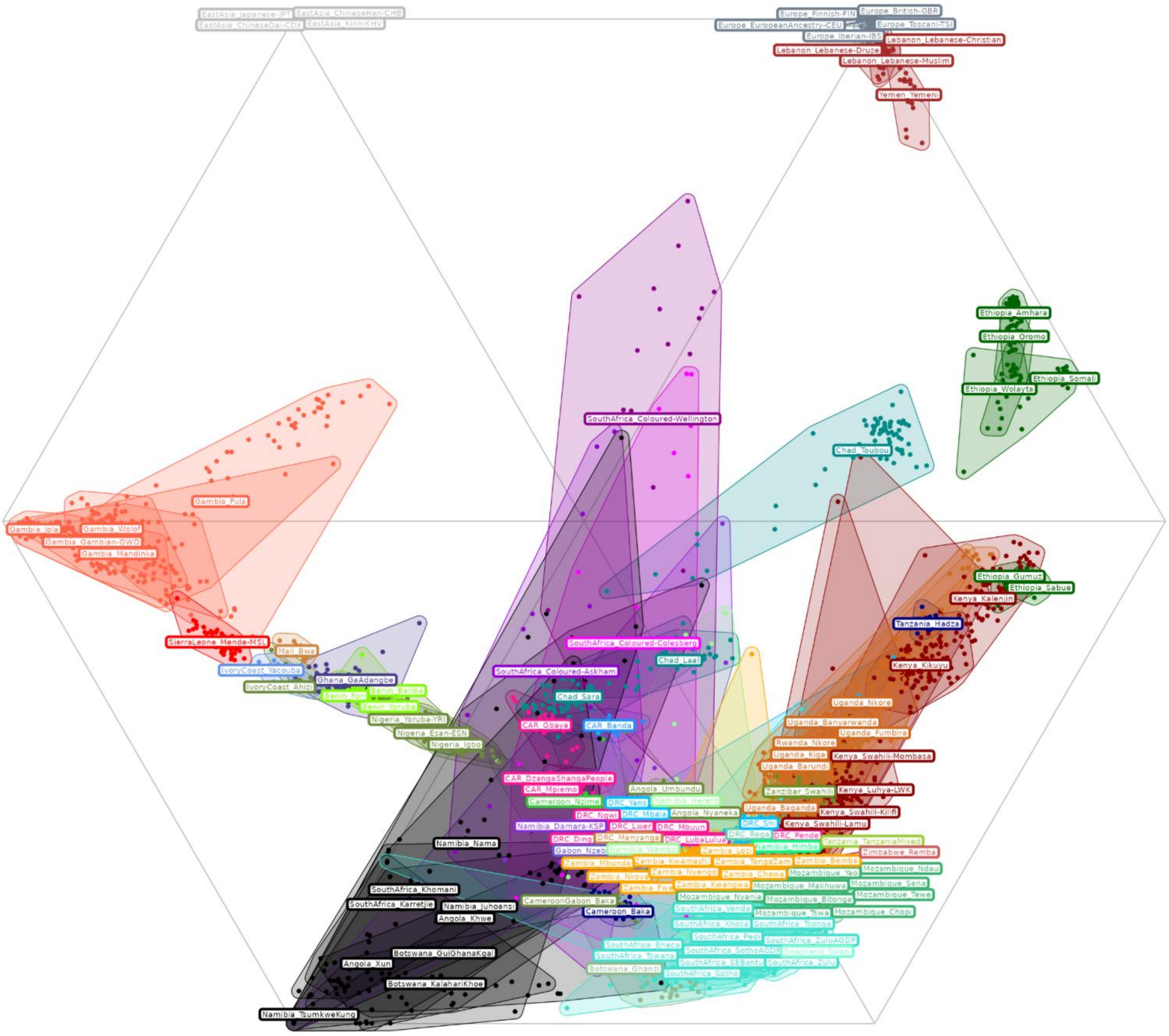

Supplementary Fig. 24 | Ternary diagrams of ADMIXTURE results at K=6.
Figure showing a ternary diagram in hexagonal shape for ADMIXTURE results at K=6 (Supplementary Fig. 19) in **a,** each group and **b**, each population included in the AfricanNeo dataset. Each corner of the hexagon corresponds to each ancestry as follows: East Asian-related ancestry on the top-left corner; European-related ancestry on the top-right corner; eastern African-related ancestry on the central-right corner; Bantu-speaking-related ancestry on the bottom-right corner; Khoe-San-speaking-related ancestry on the bottom-left corner; and western African-related ancestry on the central-left corner. Each individual is one dot from each population (shadow area), and individuals close to each corner suggest high ancestry proportions for that ancestry.

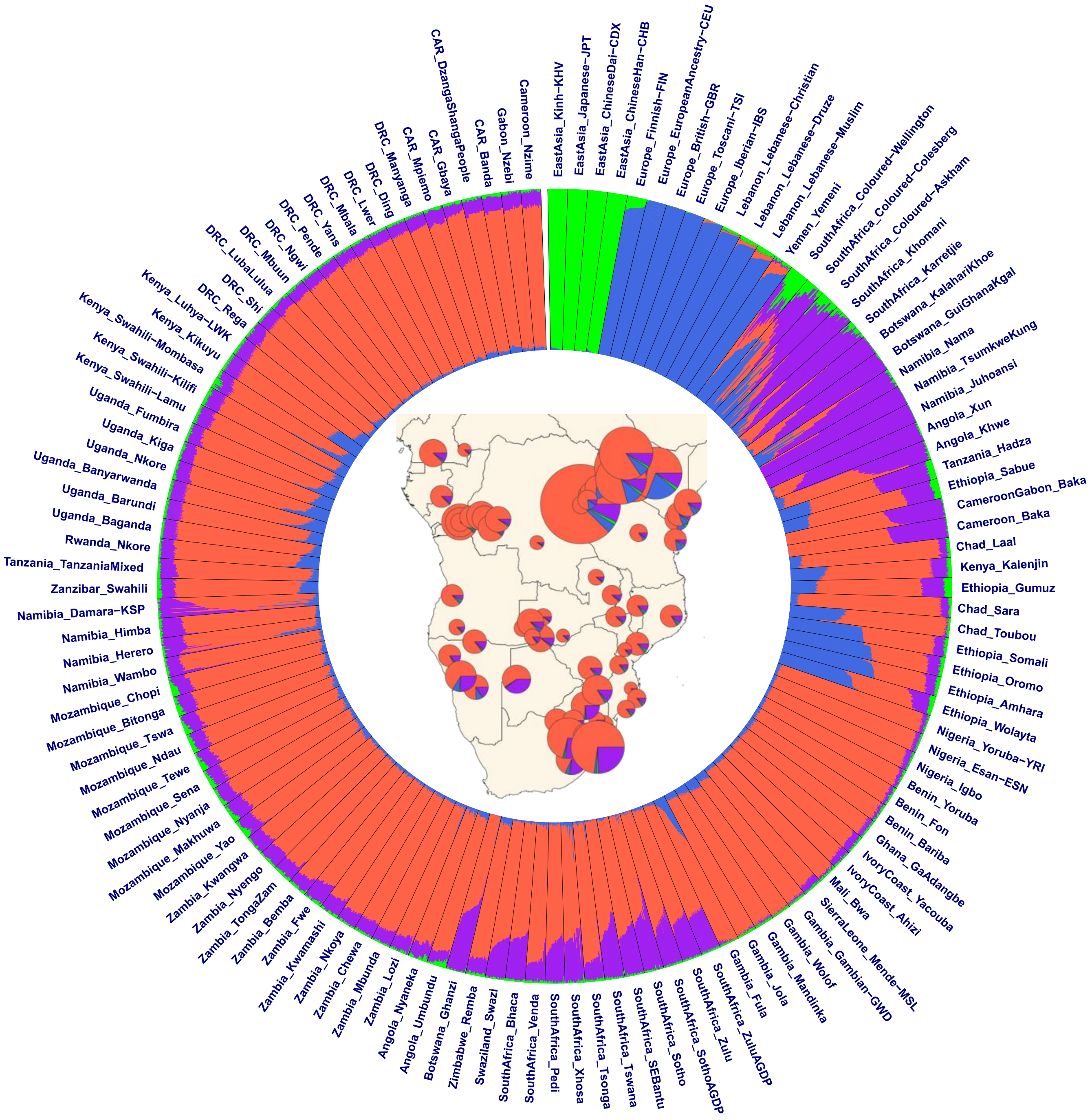

Supplementary Fig. 25 | Bar plots of ADMIXTURE results for K=4.

Panel figure showing average admixture proportions estimated using unsupervised ADMIXTURE analyses at K=4 on the basis of all the populations included in the AfricanNeo dataset (Supplementary Table 3). BSP are in the left part of the plot while comparative populations are in the right part of the plot. The central part of the figure includes the map presented in Supplementary Fig. 22b.

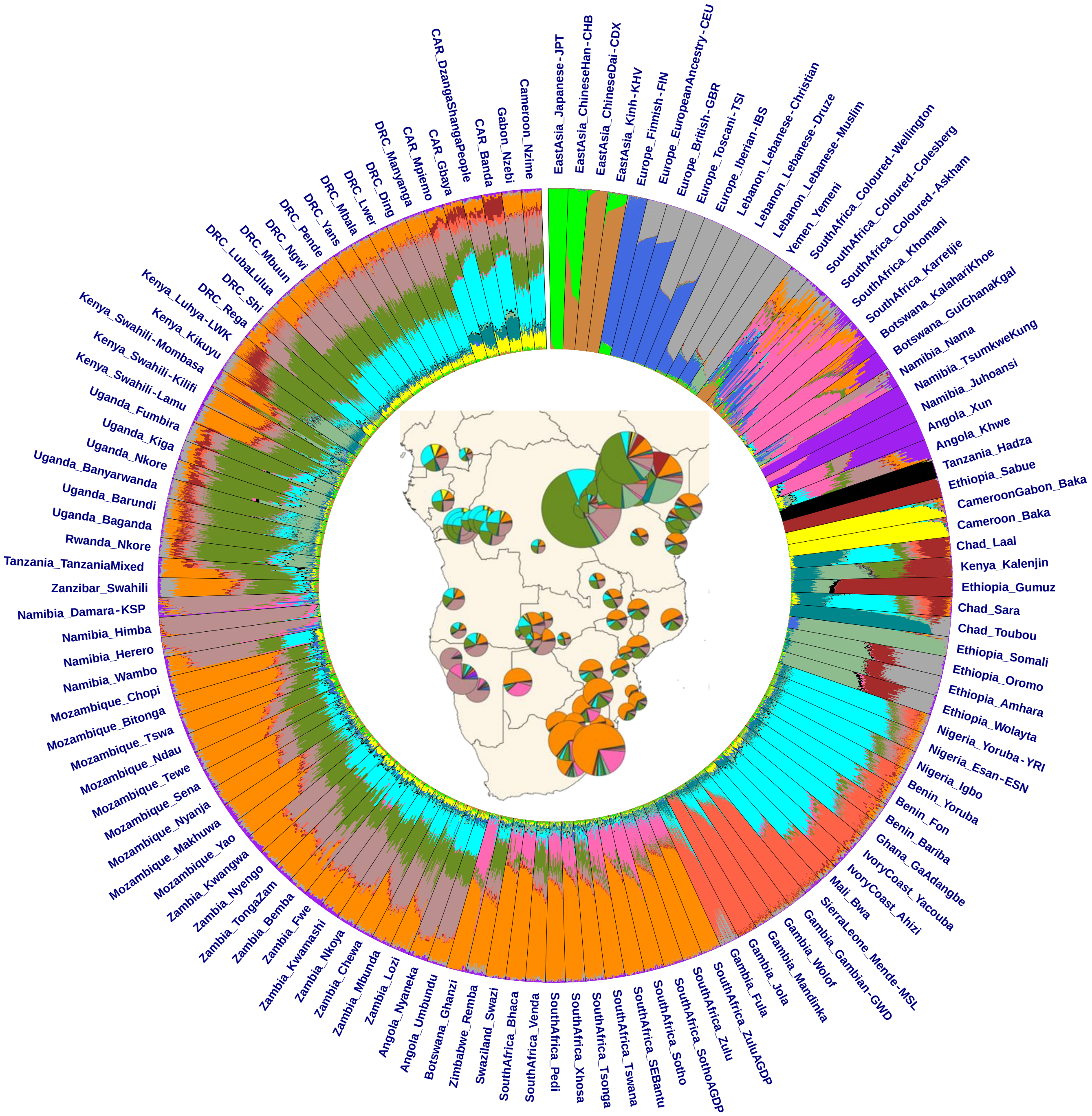

Supplementary Fig. 26 | Bar plots of ADMIXTURE results for K=16.
Panel figure showing average admixture proportions estimated using unsupervised ADMIXTURE analyses at K=16 on the basis of all the populations included in the AfricanNeo dataset (Supplementary Table 4). The colors of each K-group are matching the same colors in Supplementary Fig. 21. To better visualize the figure, the width of each population was set to be equal regardless of its sample size using AncestryPainter. BSP are in the left part of the plot while comparative populations are in the right part of the plot. The central part of the figure includes the map presented in Supplementary Fig. 23.

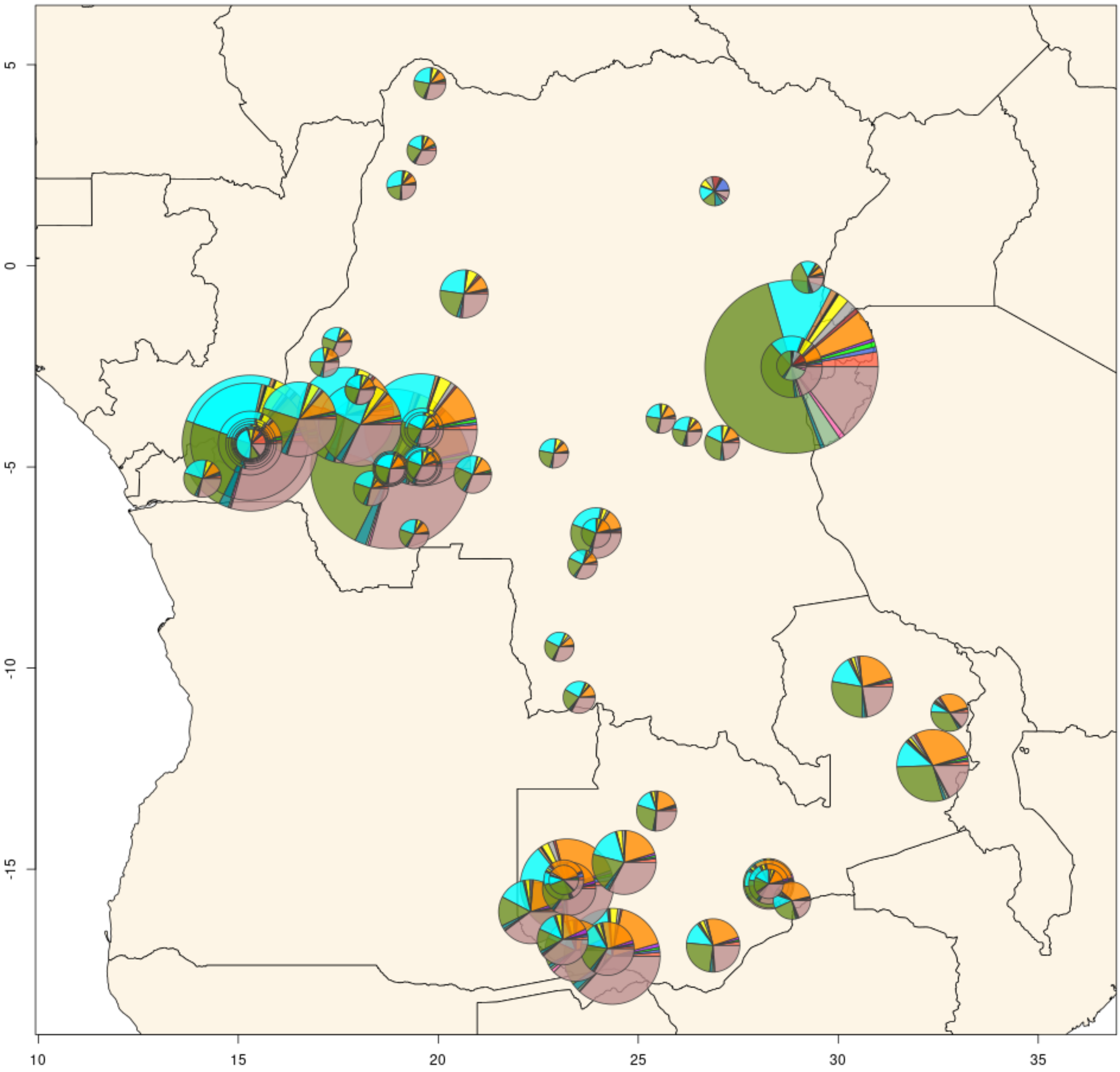

Supplementary Fig. 27 | Pie charts of ADMIXTURE results for K=16 only for DRC and Zambia. Panel figure showing unsupervised ADMIXTURE plot at K=16 only for all the genotyped BSP from DRC and Zambia (Supplementary Table 4). The smallest pie charts represent populations with only one sample while the biggest pie chart represents the Shi population with a sample size of 130 samples.

3.4. F-statistics analyses

a

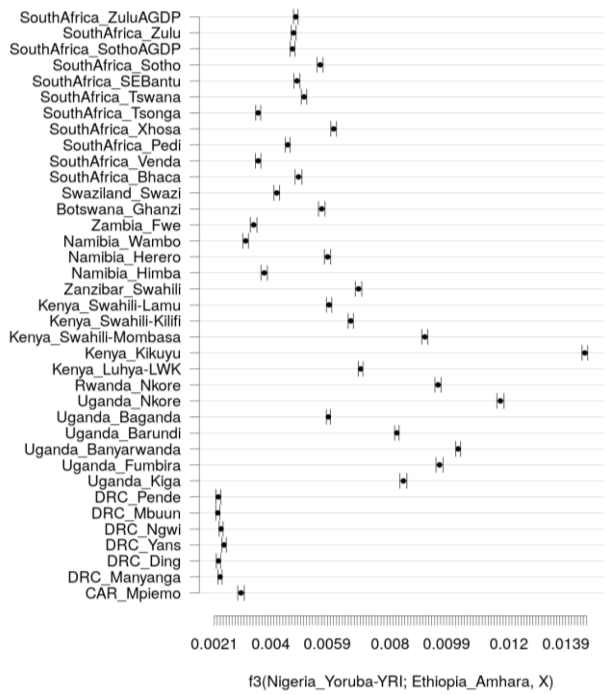

b

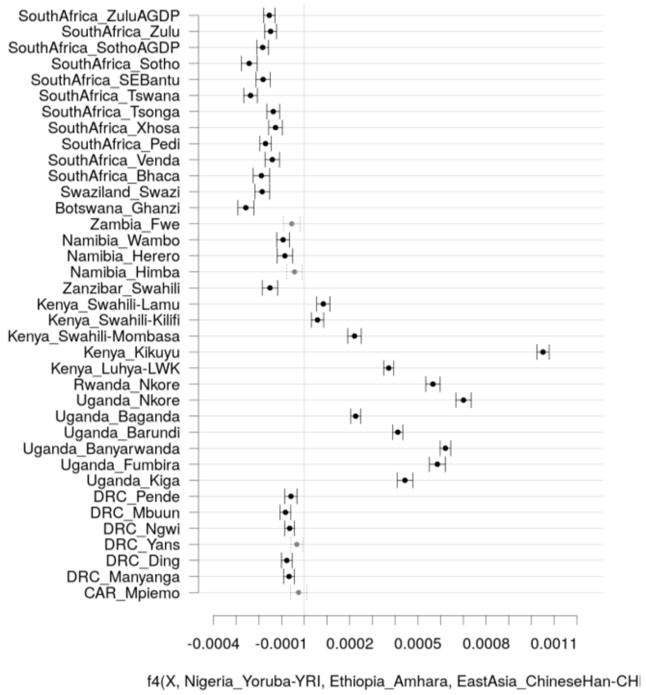

c

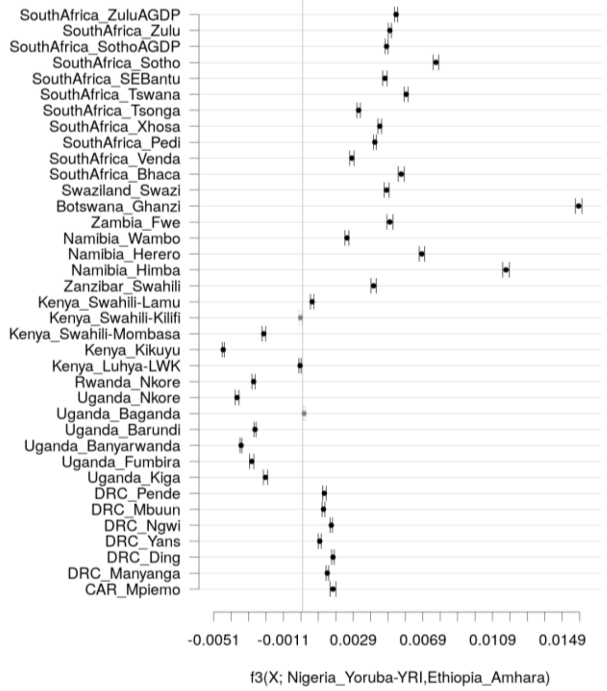

Supplementary Fig. 28 | F3- and f4-statistics tests used to estimate Afro-Asiatic admixture in BSP.

Positive values of **a**, f3-statistics in the form f3(Yoruba; Amhara, *Target*) and **b**, f4-statistics in the form f4(*Target*, Yoruba; Amhara, CHB) indicate increasing genetic affinity between the Amhara population in Ethiopia and each of the BSP indicated in the Y-axis. **c**, As a verification, we calculated f3-statistics in the form f3(*Target;* Yoruba, Amhara) (Supplementary Tables 5–6). Most positive values in (a) change to negative values. This indicates that the target has the highest affinity with both the Yoruba and Amhara. These populations are Eastern BSP from Kenya, Rwanda and Uganda. Their Yoruba-Amhara mixed ancestry reflects the original WCA ancestry that they brought via the migration of BSP and Afro-Asiatic admixture, respectively, in agreement with other genetic evidence that we discuss in the main text. Bars indicate the standard error of each mean. Values of the f-statistics significantly higher than zero (P-value < 0.05) are indicated with black color. BSP are in the same order in f3- and f4-statistics plots.

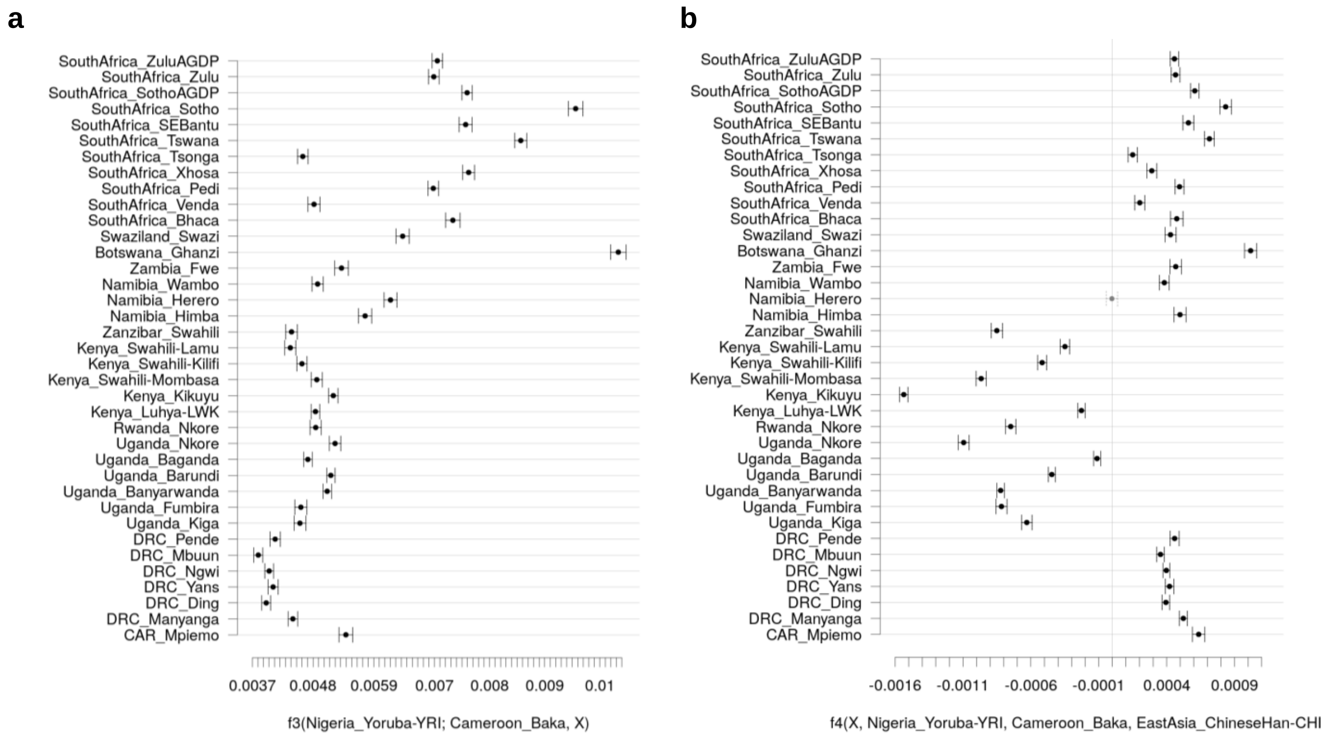

Supplementary Fig. 29 | F3- and f4-statistics tests used to estimate wRGH admixture in BSP. Positive values of **a**, f3-statistics in the form f3(Yoruba; Baka, *Target*) and **b**, f4-statistics in the form f4(*Target*, Yoruba; Baka, CHB) indicate increasing genetic affinity between the wRHG Baka population to each of the studied BPS indicated in the Y-axis (Supplementary Tables 5–6). Bars indicate the standard error (SE) of the mean value. Values of the statistics significantly different from zero (*P*-value <0.05) are indicated with black color. BSP are in the same order in the Y-axis of both plots. Note that two groups of BSP show affinity with western RHG, western and southern African BSP. This most likely reflects the hunter-gatherer ancestry present in southern African BSP. Differential affinities for western and southern African BSP are visible in Supplementary Fig. 32.

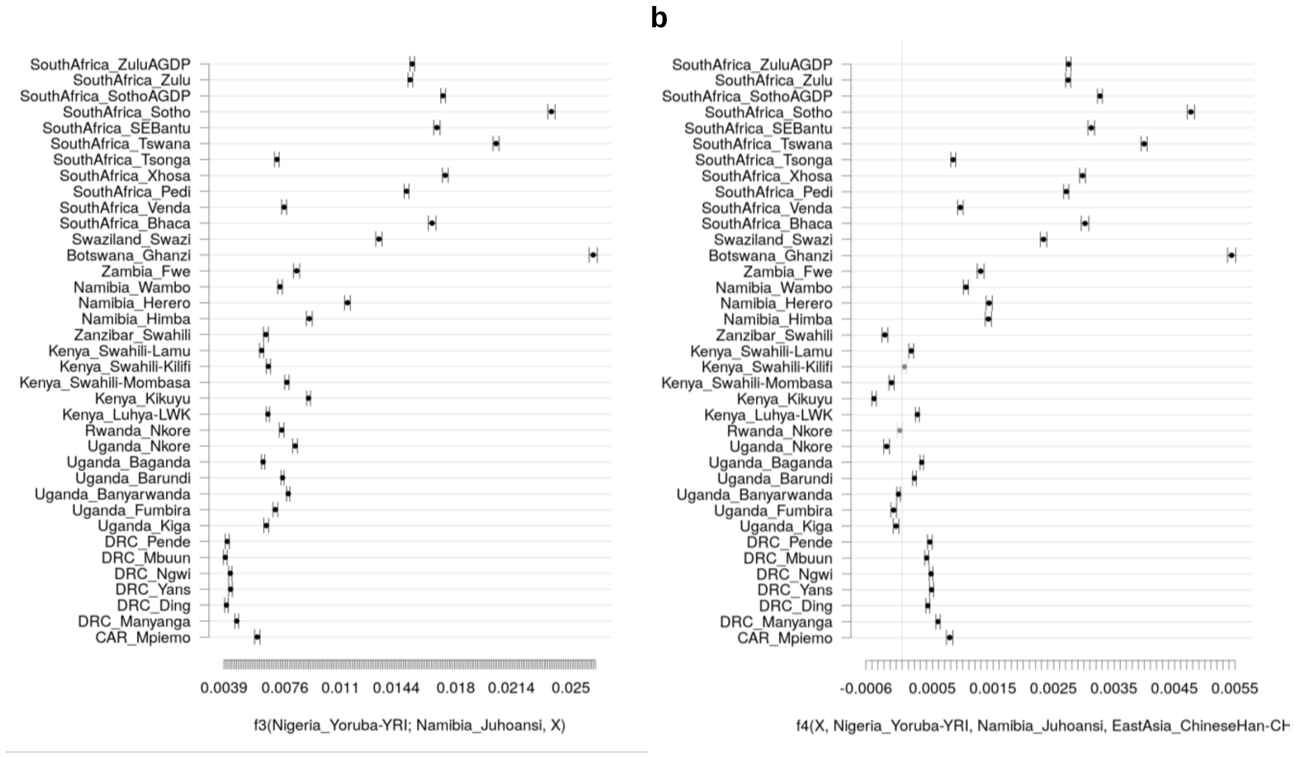

Supplementary Fig. 30 | F3- and f4-statistics tests used to estimate Khoe-San admixture in BSP. Positive values of **a**, f3-statistics in the form f3(Yoruba; Ju'hoansi, *Target*) and **b**, f4-statistics in the form f4(*Target*, Yoruba; Ju'hoansi, CHB) indicate increasing genetic affinity between the Ju'hoansi population in Namibia to each of the BSP indicated in the Y-axis (Supplementary Tables 5–6). Bars indicate the standard error of the mean. Values of the statistics significantly higher than zero (P-value < 0.05) are indicated with black color. BSP are in the same order in (a) and (b) plots.

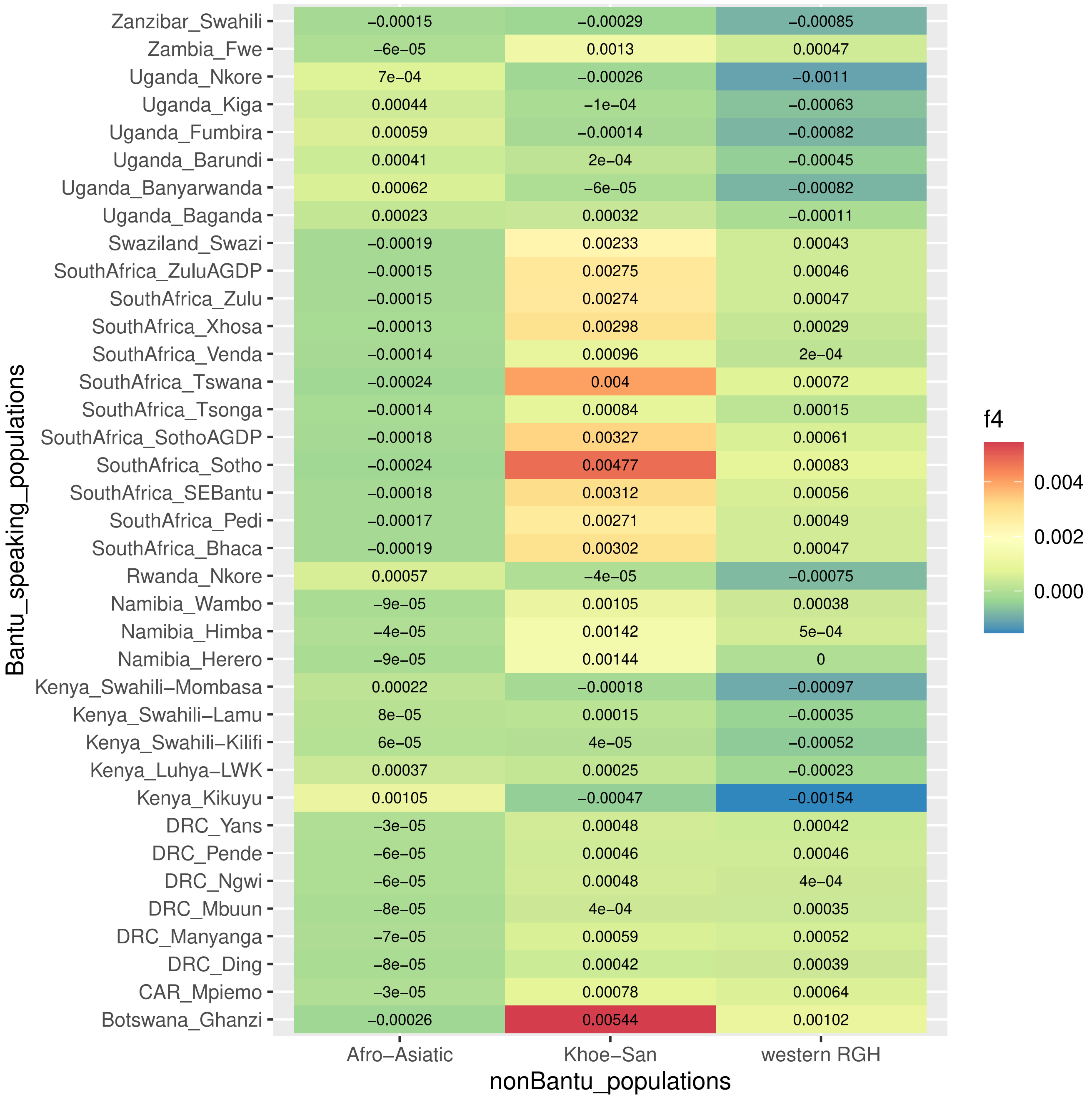

Supplementary Fig. 31 | Genetic affinity of BSP to non-BSP estimated using f4-statistics. Genetic affinity of BSP to nonBantu ancestries calculated in the form f4(*Target*, Yoruba; Baka, CHB). The non-Bantu ancestry is represented respectively by the Afro-Asiatic-speaking population from Ethiopia (Amhara), the western RGH Baka population and the Khoe-San-speaking population (Ju'hoansi). Mean *f*4 values are indicated, and the standard errors of the mean are reported in Supplementary Fig.s 28–30 and Supplementary Table 6.

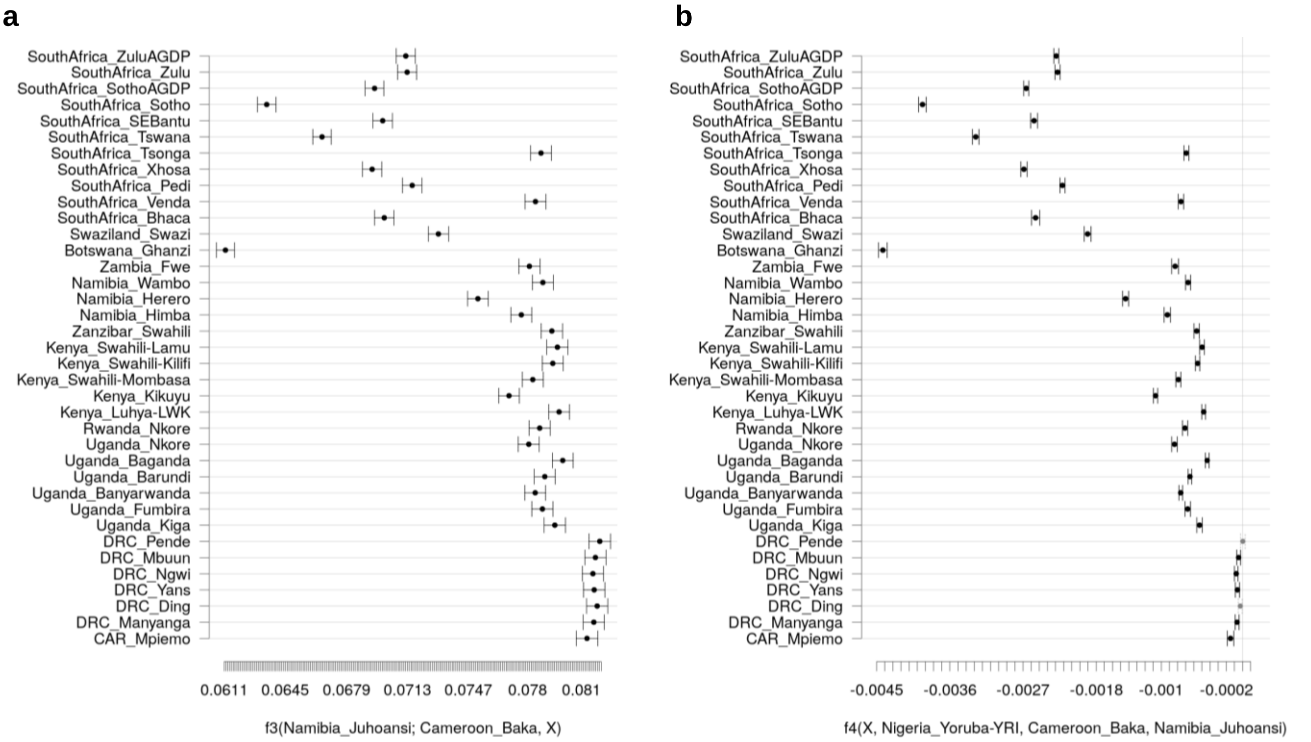

Supplementary Fig. 32 | F3- and f4-statistics tests used to estimate hunter-gatherer admixture in BSP.

To disentangle the differential contribution of different hunter-gatherer groups, we performed **a**, f3 tests in the form f3(Ju'hoansi; Baka, *Target*), and **b,** f4 tests in the form f4(*Target*, Yoruba; Baka, Ju'hoansi). Both tests indicate that western BSP have a closer affinity to Baka groups than southern African BSP. Bars indicate the standard error of the mean. Values of the statistics significantly different from zero (*P*-value < 0.05) are indicated with black color. BSP are in the same order in f3 and f4 plots.

3.5. Ancestry-specific analyses

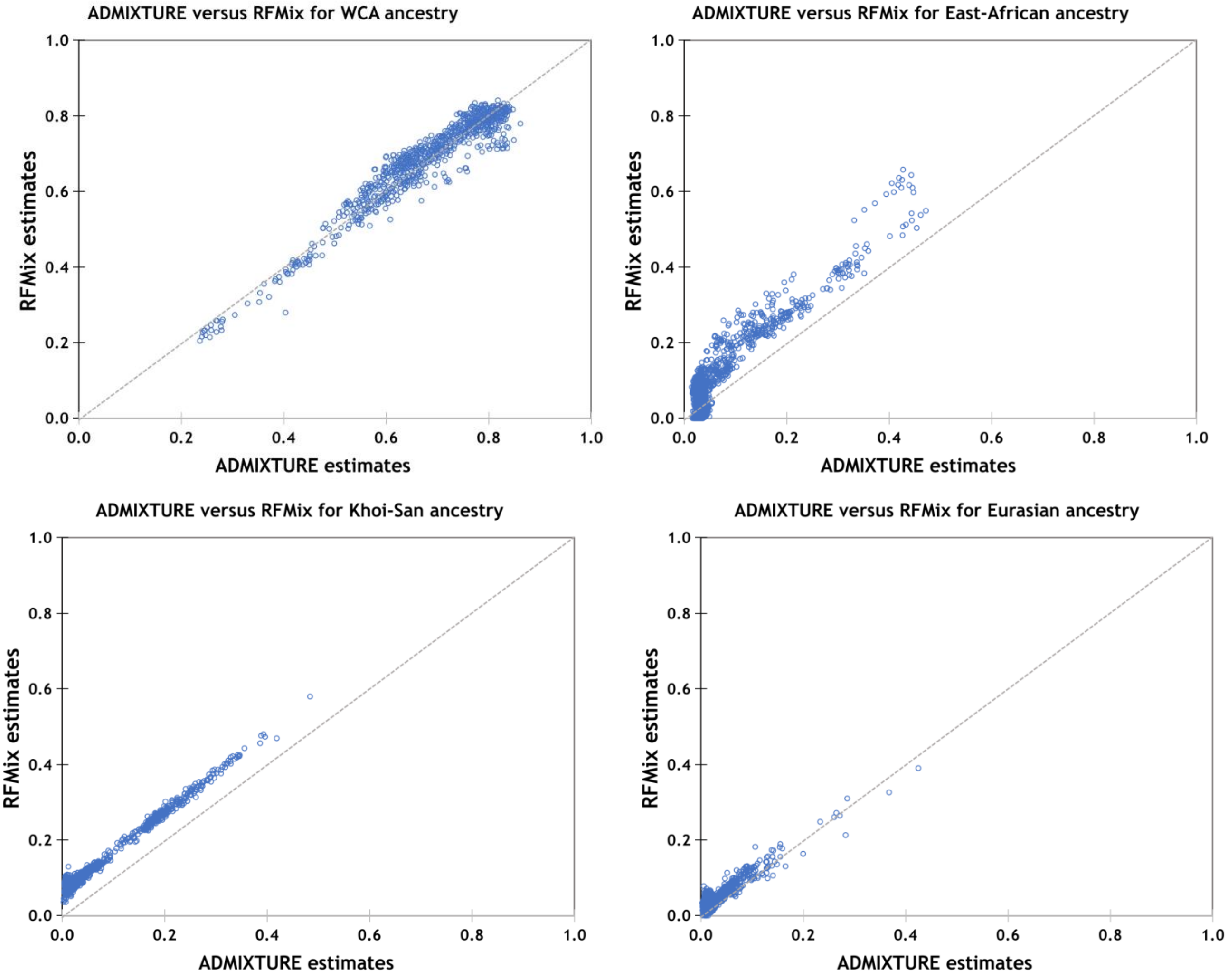

Supplementary Fig. 33 | Comparisons between estimated admixture fractions
Figure showing for each ancestry, comparisons between estimated admixture fractions using genotype-based ADMIXTURE and haplotype-based RFMix for all BSP individuals.

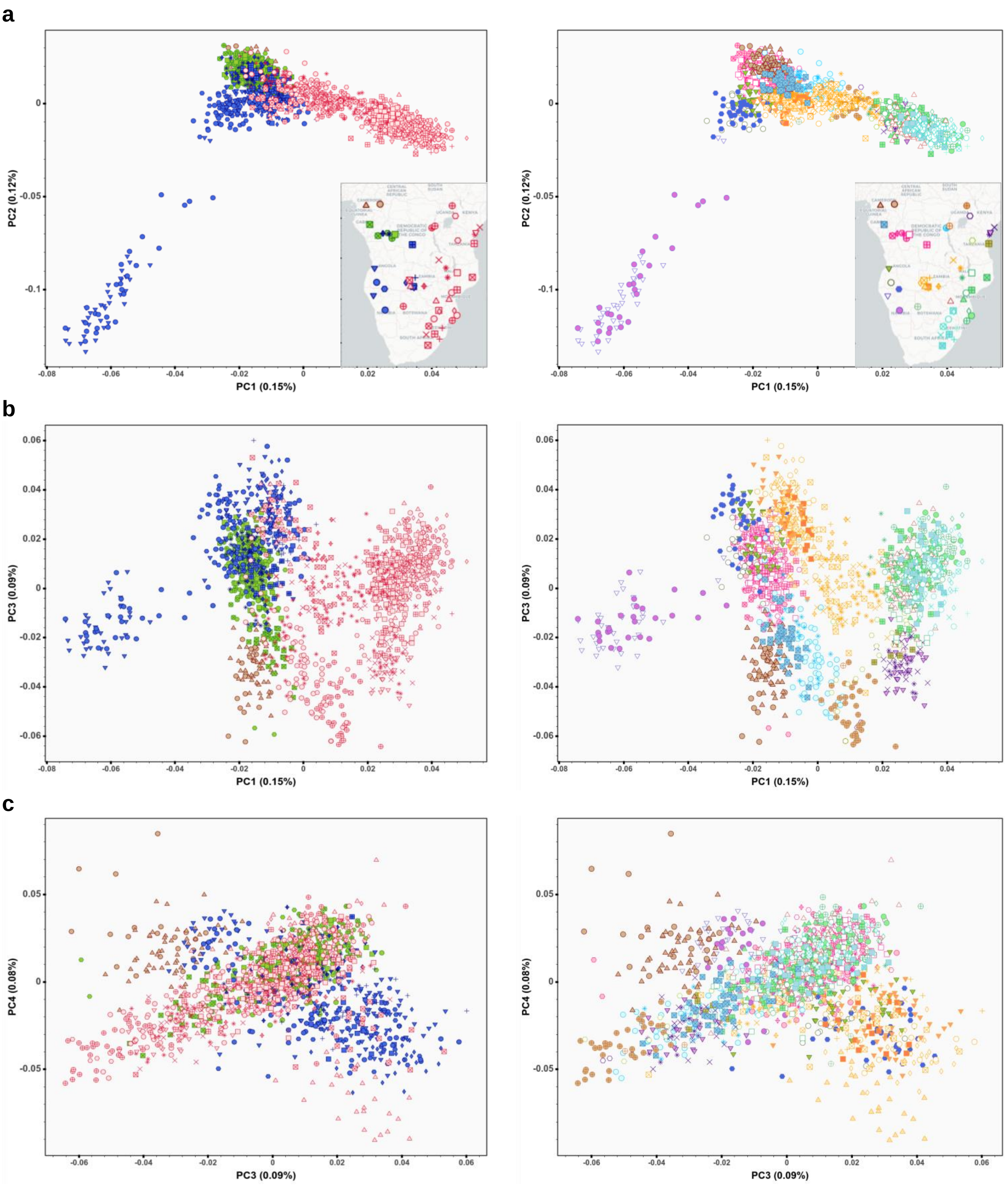

Supplementary Fig. 34 | Ancestry-specific (AS-)PCA for the masked Only-BSP dataset.
Figure showing AS-PCA plot of BSP included in the AfricanNeo dataset after masking and imputation of populations with 70% WCA-related ancestry, without using the Procrustes approach. PC projections were obtained for each group (left column) and for each population (right column) between: **a**, PC1 vs PC2; **b**, PC1 vs PC3; and **c**, PC3 vs PC4. Interactive plots are available at Github [113] (Suppl_Fig_34_ASPCA_[Groups or Pop.s].html).

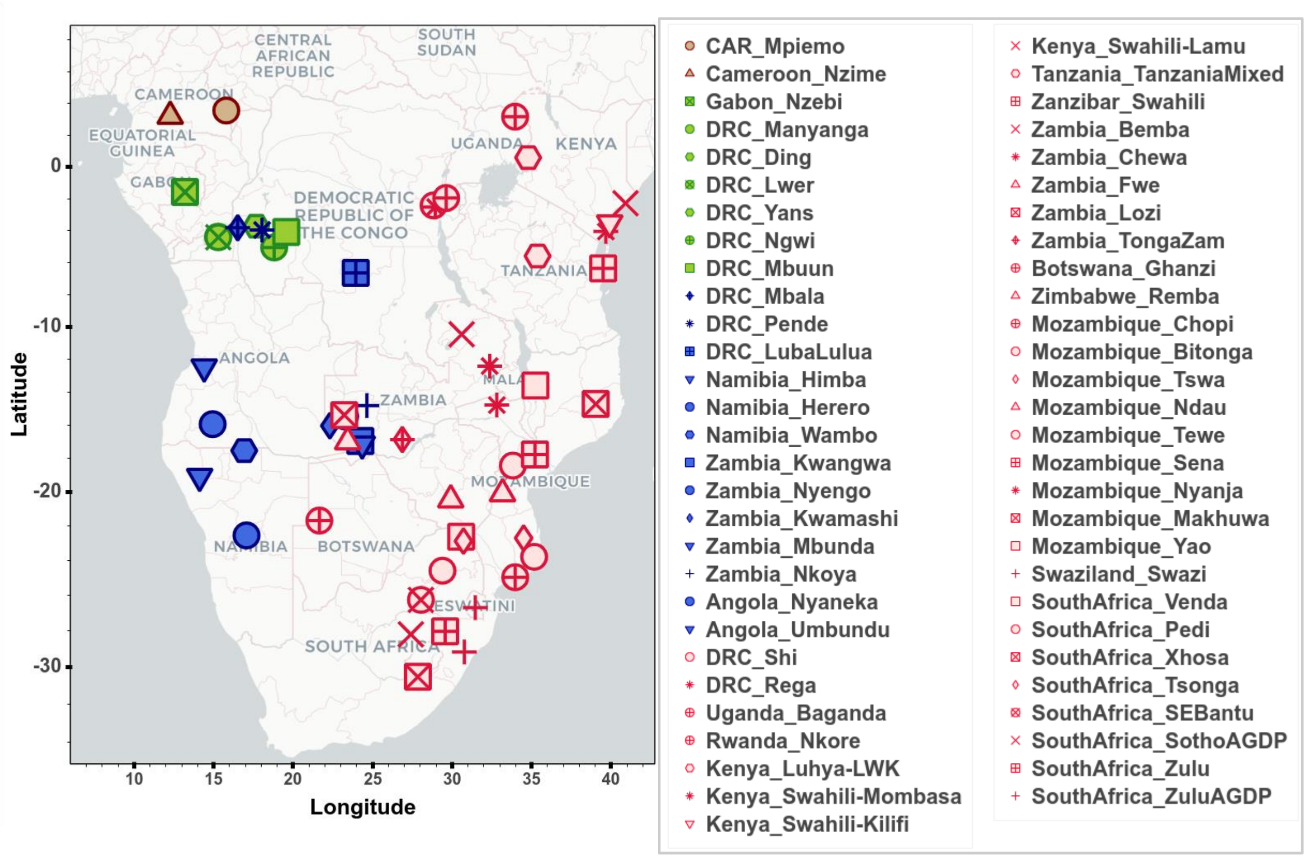

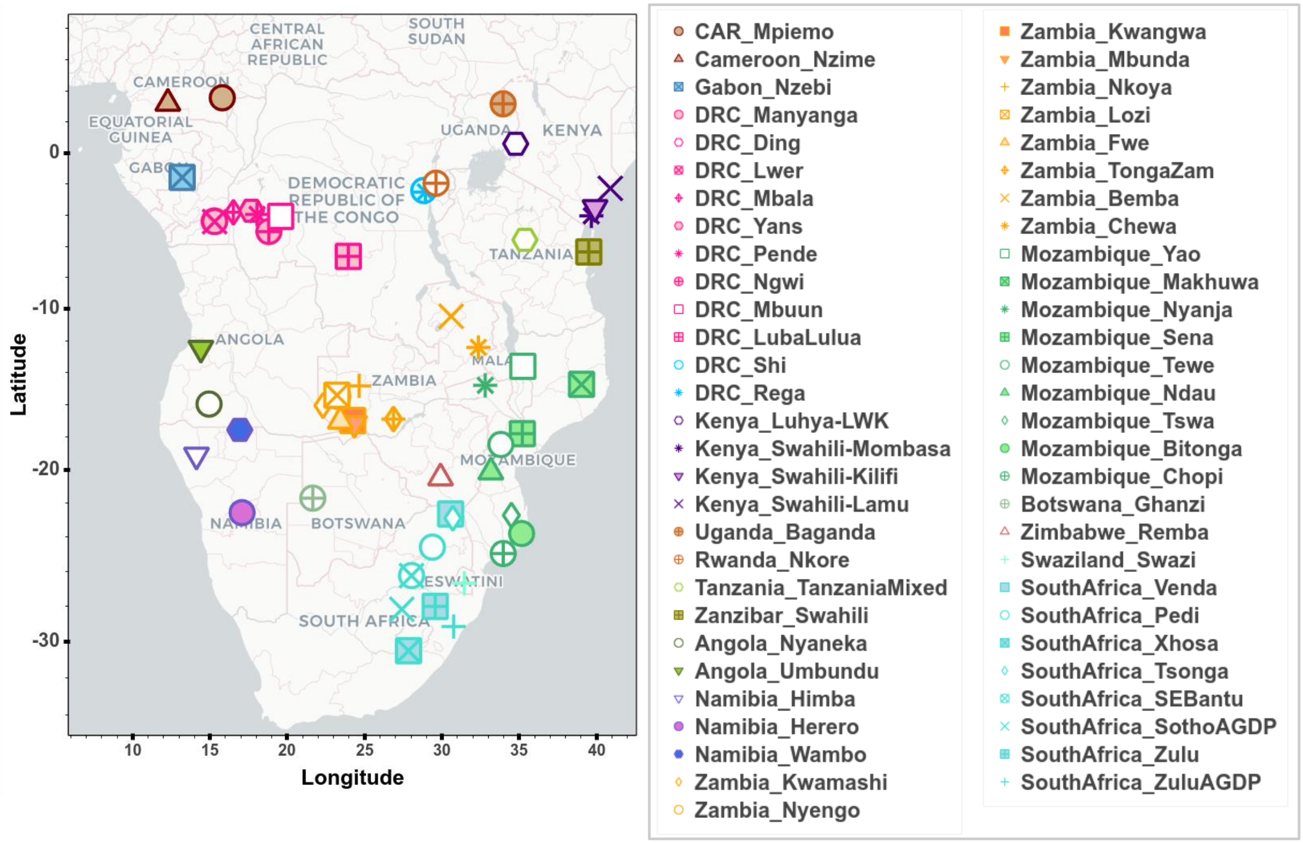

Legends and maps for each group (top) and each population (bottom) in Supplementary Fig. 34. Vector basemap and map tiles were provided by CartoDB (© CARTO 2023).

3.6. Genome-wide runs of homozygosity (ROH) estimates

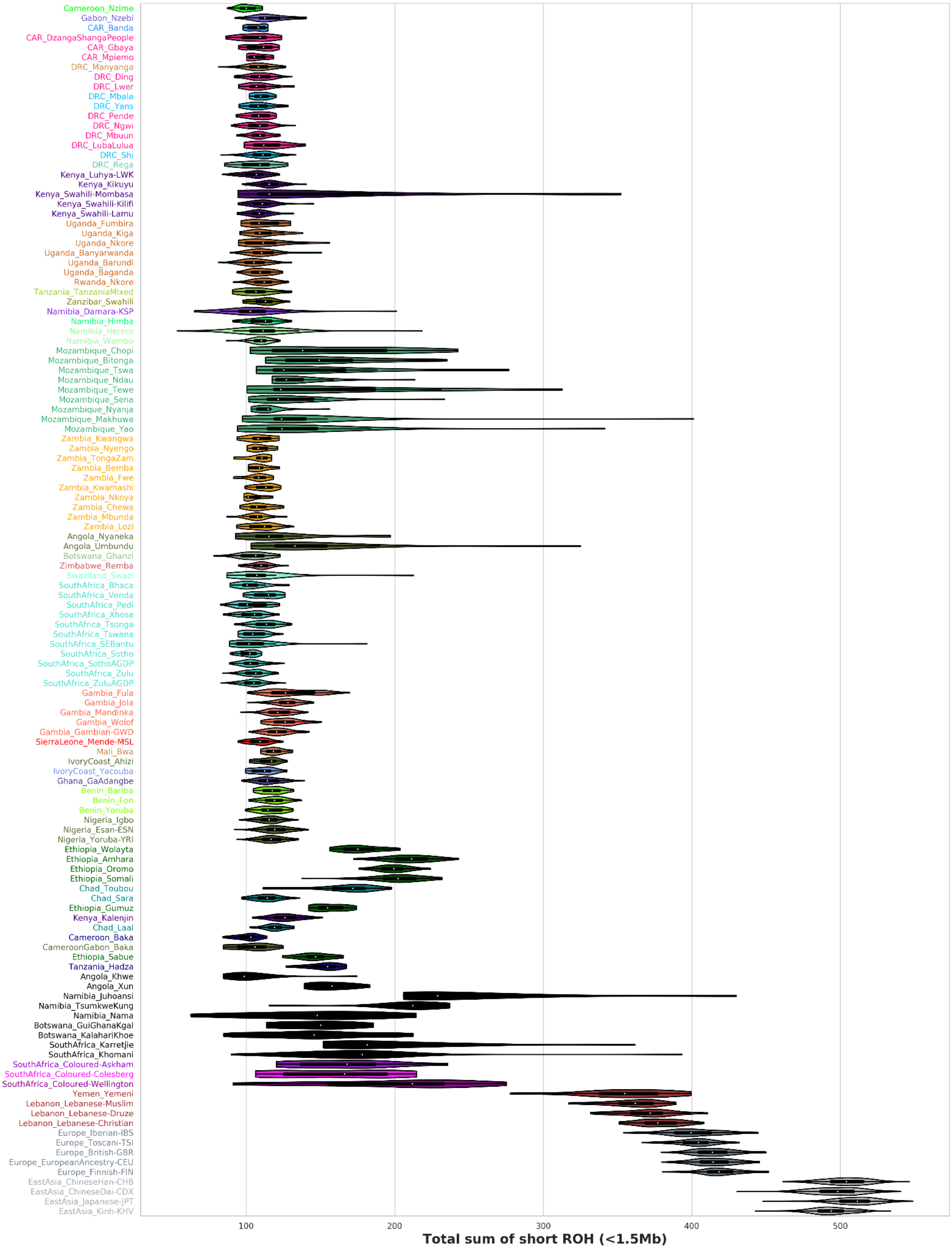

Supplementary Fig. 35 | Total sum of short ROH length for the AfricanNeo dataset.
Figure showing violin plots of the total sum of short ROH length (shorter than 1.5 Mb) in African and Eurasian populations included in the AfricanNeo dataset (mean values and +/-SD values were included in Supplementary Table 7).

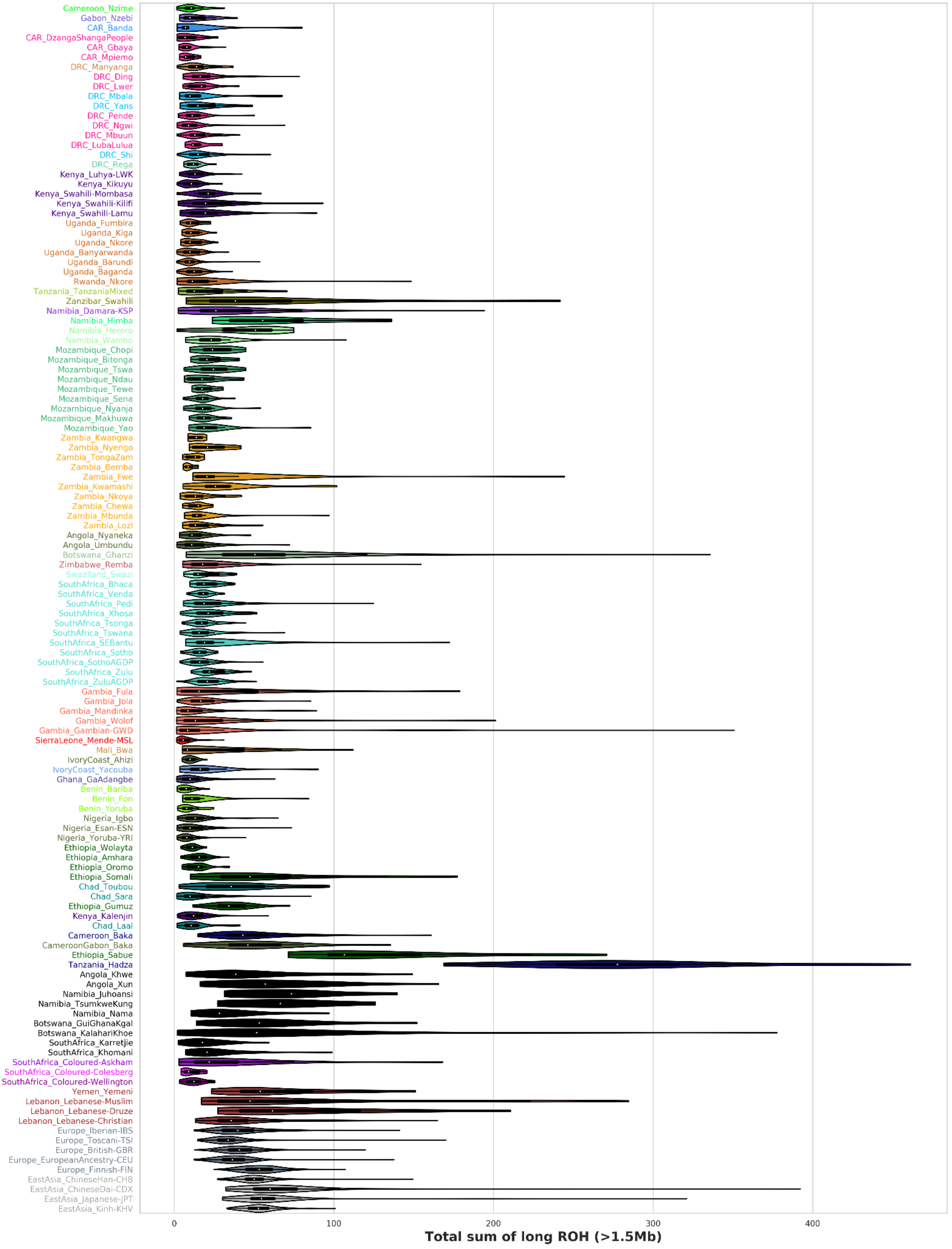

Supplementary Fig. 36 | Total sum of long ROH length for the AfricanNeo dataset.
Figure showing violin plots of the total sum of long ROH length (for segments longer than 1.5 Mb) in African and Eurasian populations included in the AfricanNeo dataset (mean values and +/-SD values were included in Supplementary Table 7).

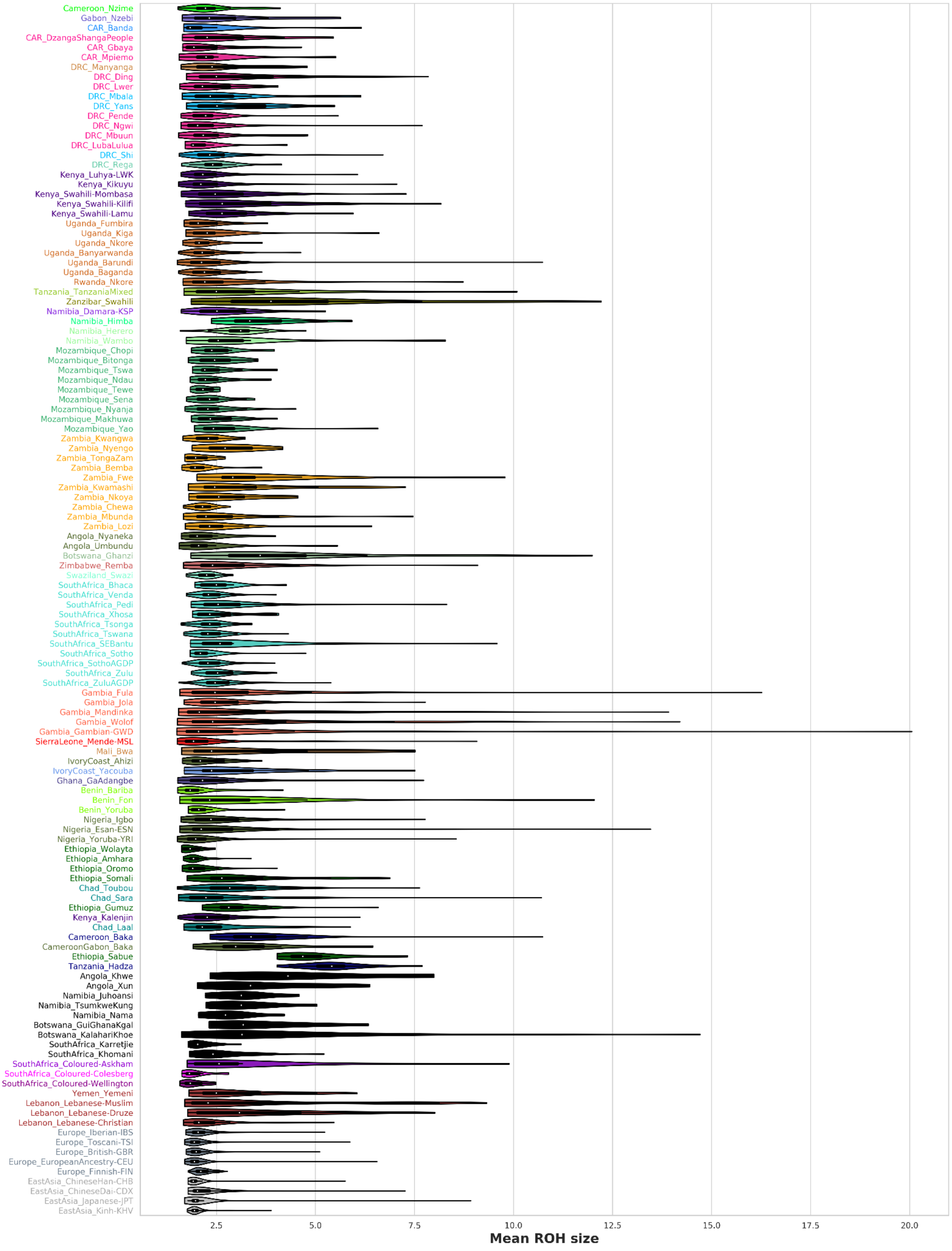

Supplementary Fig. 37 | Mean of long ROH for the AfricanNeo dataset.
Figure showing violin plots of the mean of long ROH (for segments longer than 1.5 Mb) in African and Eurasian populations included in the AfricanNeo dataset (mean values and +/-SD values were included in Supplementary Table 7).

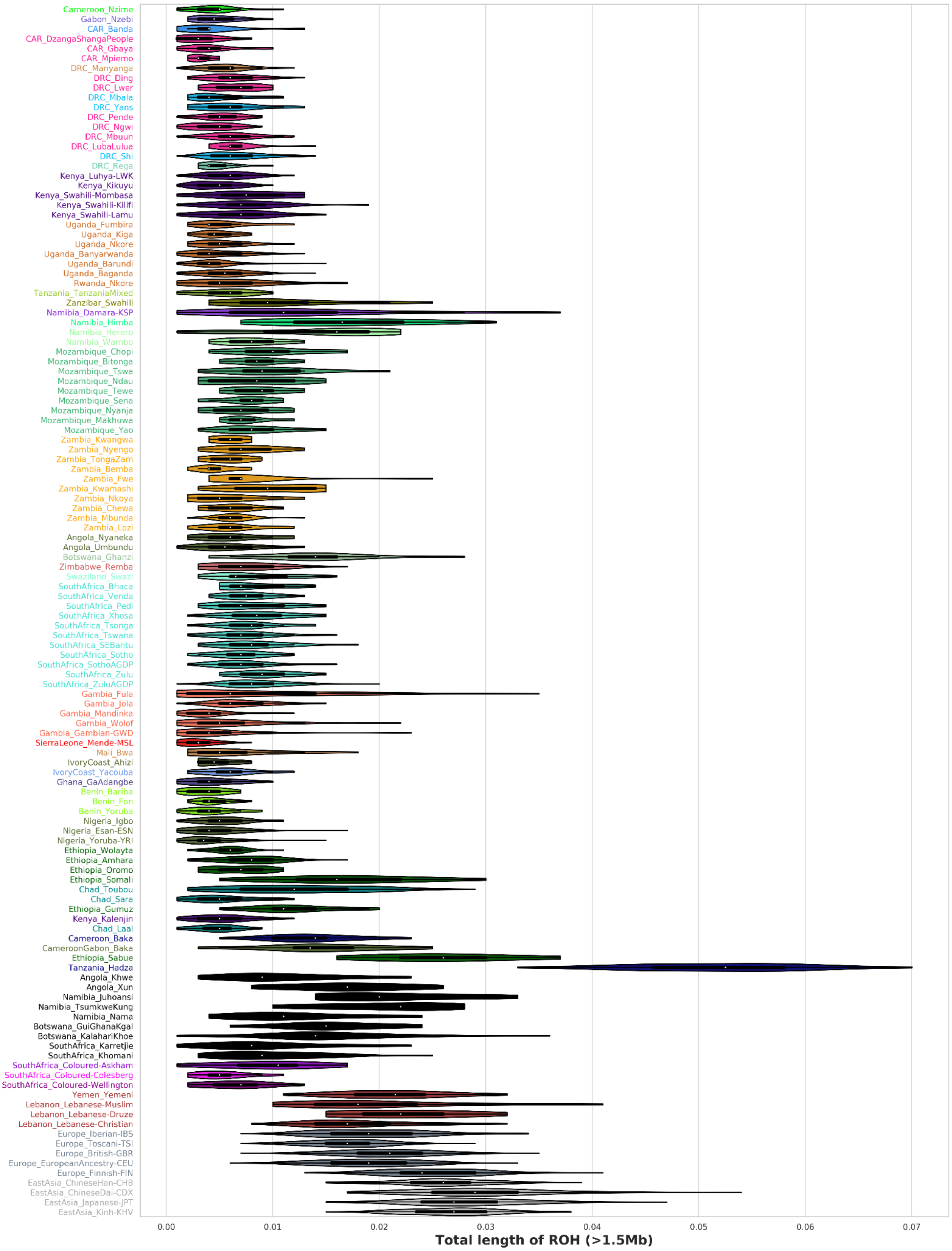

Supplementary Fig. 38 | Total length of long ROH for the AfricanNeo dataset.
Figure showing violin plots of the total length of long ROH (for segments longer than 1.5 Mb) in African and Eurasian populations included in the AfricanNeo dataset (mean values and +/-SD values were included in Supplementary Table 7).

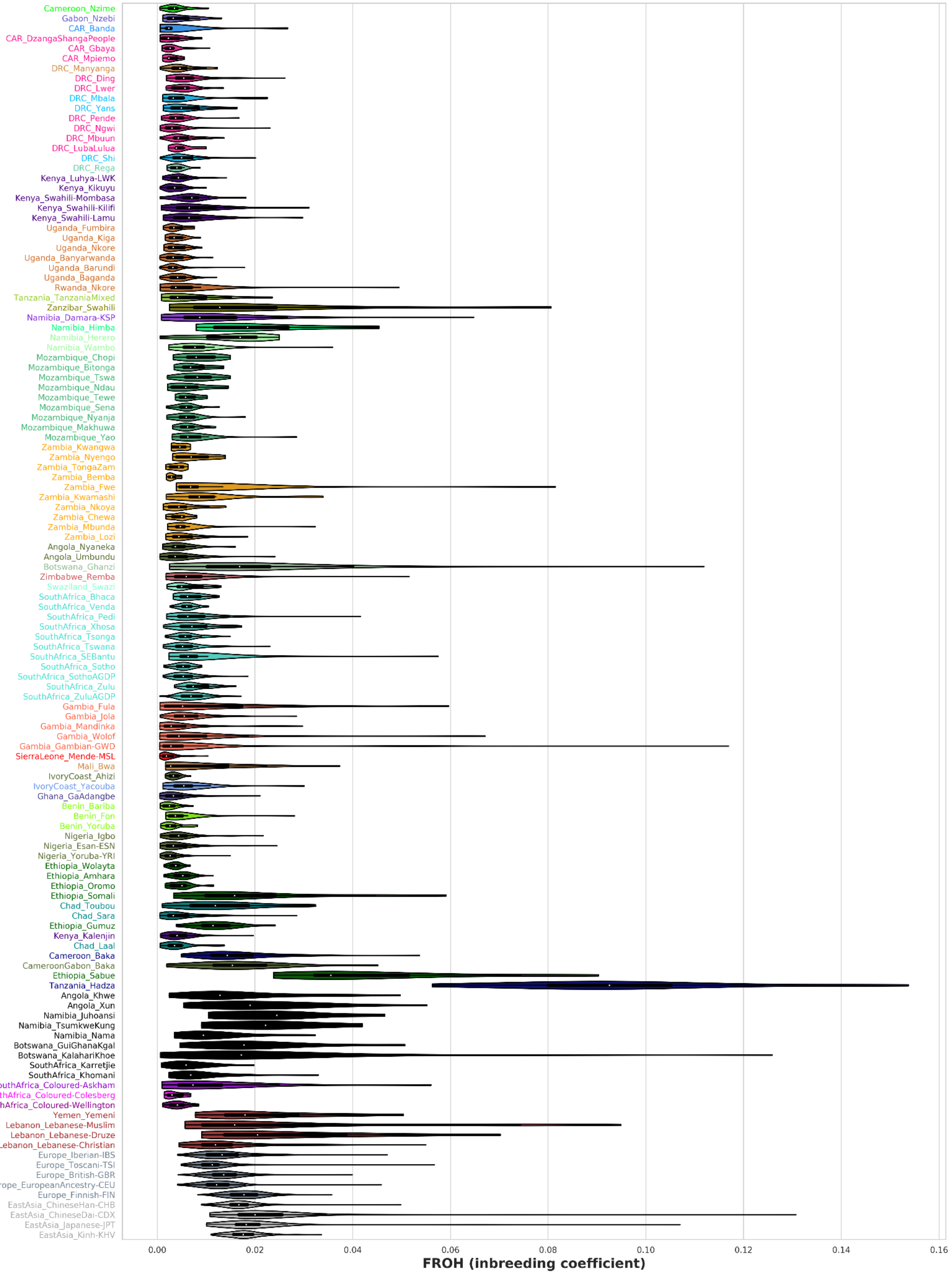

Supplementary Fig. 39 | Genomic inbreeding coefficient for the AfricanNeo dataset.
Figure showing estimated genomic inbreeding coefficient (F_{ROH}) in African and Eurasian populations included in the AfricanNeo dataset (mean values and +/-SD values were included in Supplementary Table 7).

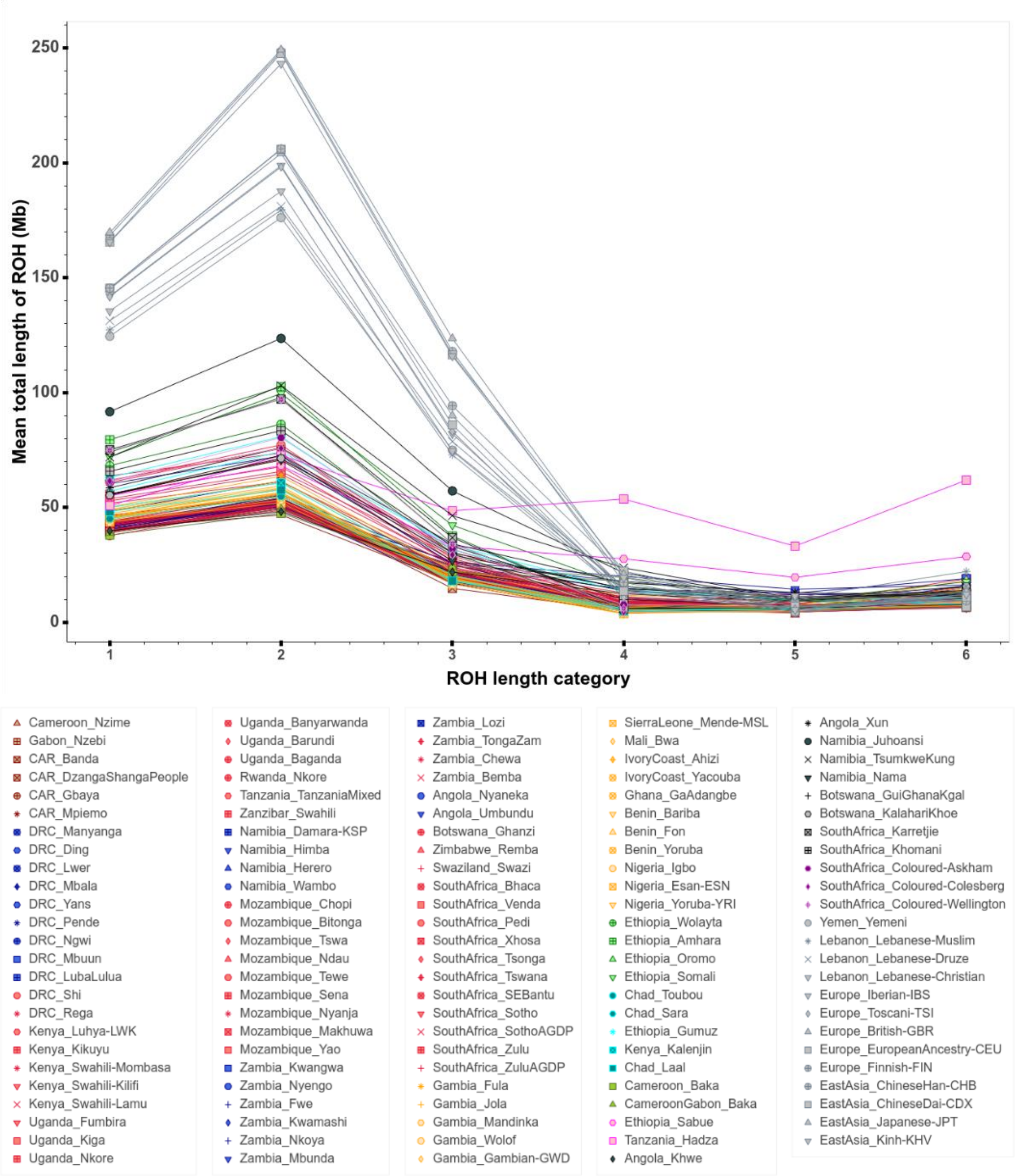

Supplementary Fig. 40 | All categories of ROH length for the AfricanNeo dataset.
Figure showing averages for each category of ROH length (for segments longer than 0.3 Mb and shorter than 10 Mb) in each studied population included in the AfricanNeo dataset (mean values and +/-SD values were included in Supplementary Table 7). Violin plots of each population and for each category of ROH length are presented in Supplementary Fig.s 42–47. To better visualize the results of each population, interactive plots are available at Github [113] (Suppl_Fig_40_All_ROH.html). Separate results for only BSP were also presented in Supplementary Fig. 41.

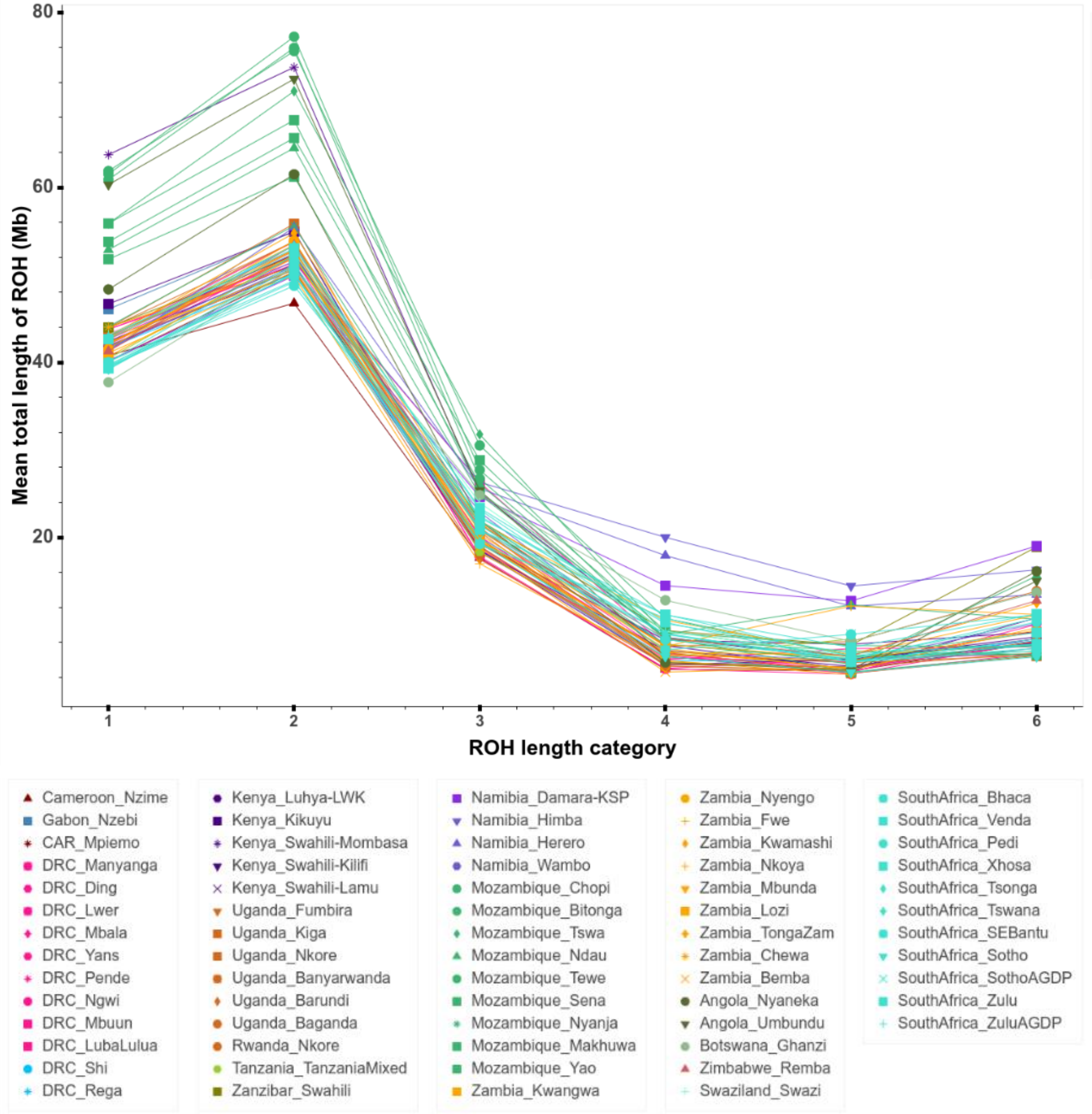

Supplementary Fig. 41 | All categories of ROH length for the Only-BSP dataset.
Figure showing averages in each BSP for each category of ROH length (for segments longer than 0.3 Mb and shorter than 10 Mb). Violin plots of each population and for each category of ROH length are presented in Supplementary Fig. 42–47, while the mean and +/-SD values are presented in Supplementary Table 7. To better visualize the results of each BSP, interactive plots are available at Github [113] (Suppl_Fig_41_BSP_ROH.html).

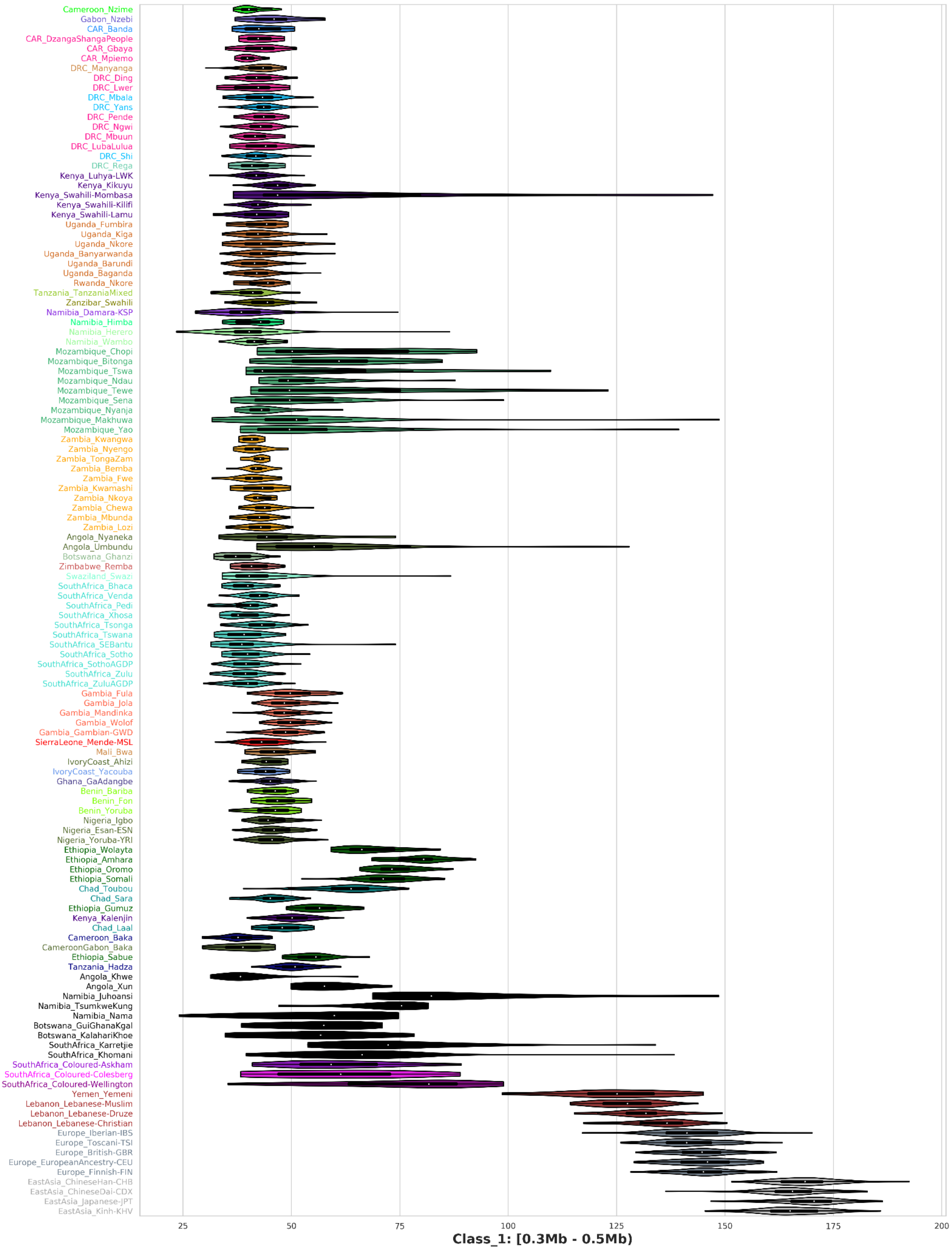

Supplementary Fig. 42 | Violin plots for category 1 of ROH length.
Category 1 of ROH length for segments longer than 0.3 Mb and shorter than 0.5 Mb in populations included in the AfricanNeo dataset (see mean and +/-SD values in Supplementary Table 7).

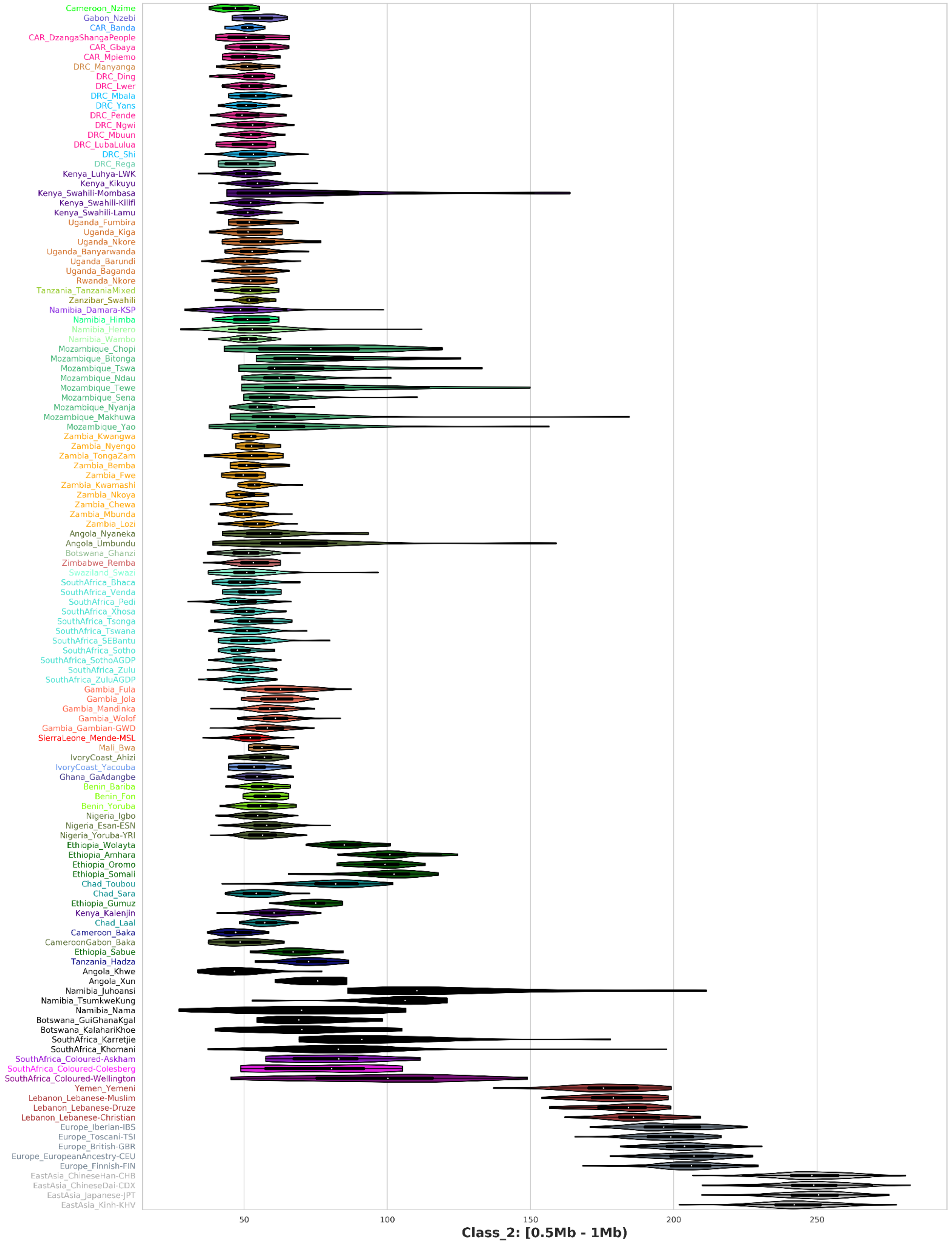

Supplementary Fig. 43 | Violin plots for category 2 of ROH length.
Category 2 of ROH length for segments longer than 0.5 Mb and shorter than 1.0 Mb in populations included in the AfricanNeo dataset (see mean and +/-SD values in Supplementary Table 7).

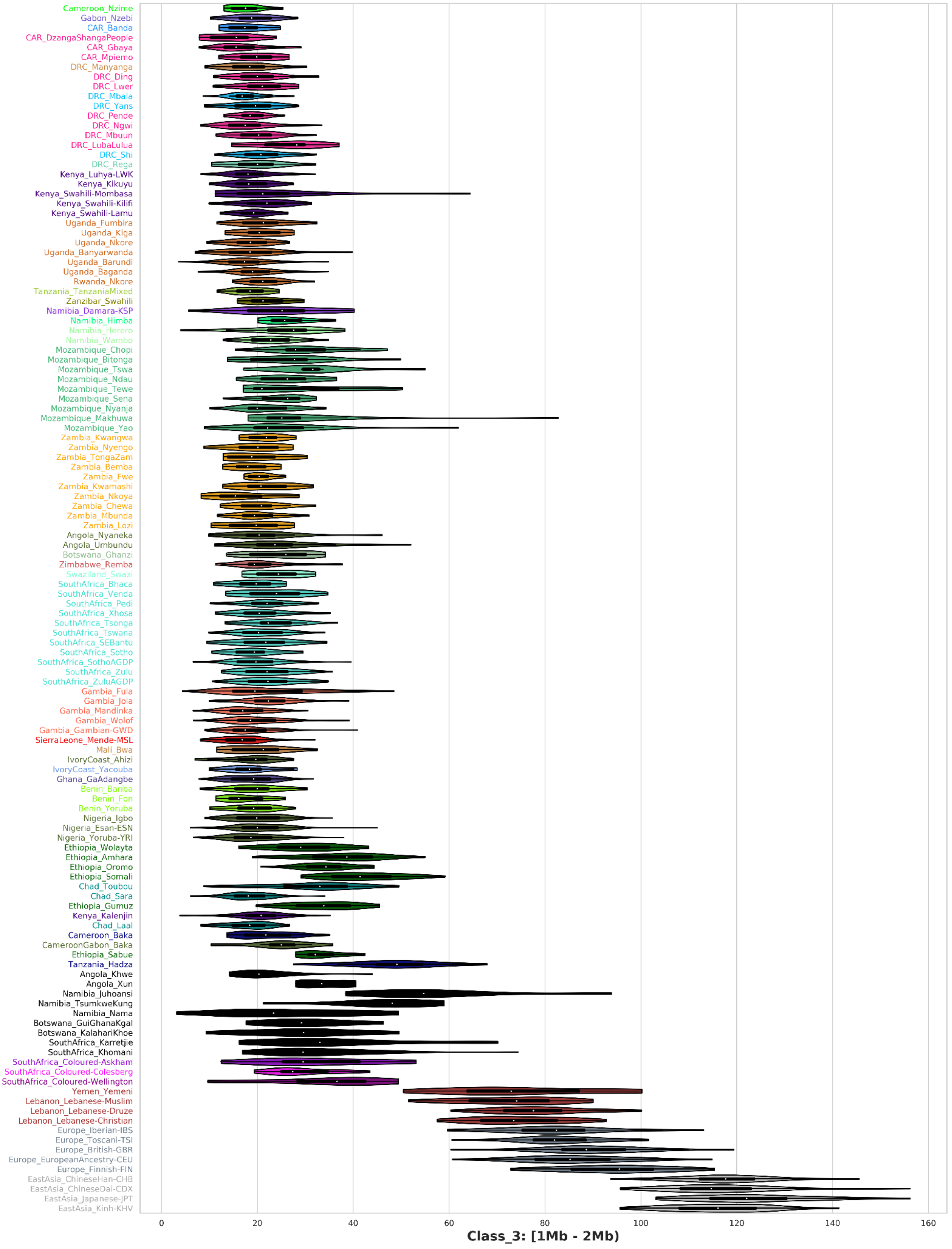

Supplementary Fig. 44 | Violin plots for category 3 of ROH length.
Category 3 of ROH length for segments longer than 1.0 Mb and shorter than 2.0 Mb in populations included in the AfricanNeo dataset (see mean and +/-SD values in Supplementary Table 7).

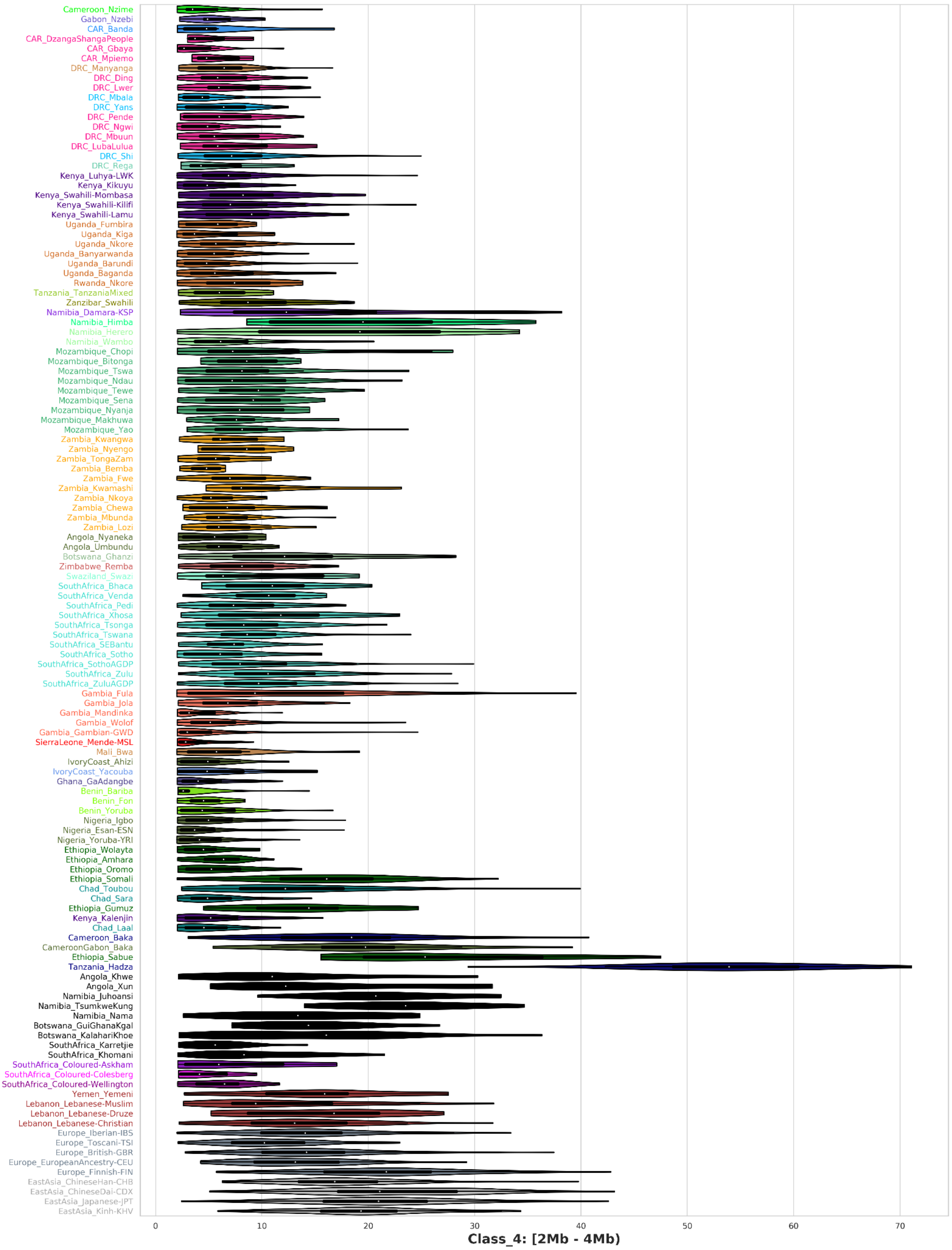

Supplementary Fig. 45 | Violin plots for category 4 of ROH length.

Category 4 of ROH length for segments longer than 2.0 Mb and shorter than 4.0 Mb in populations included in the AfricanNeo dataset (see mean and +/-SD values in Supplementary Table 7).

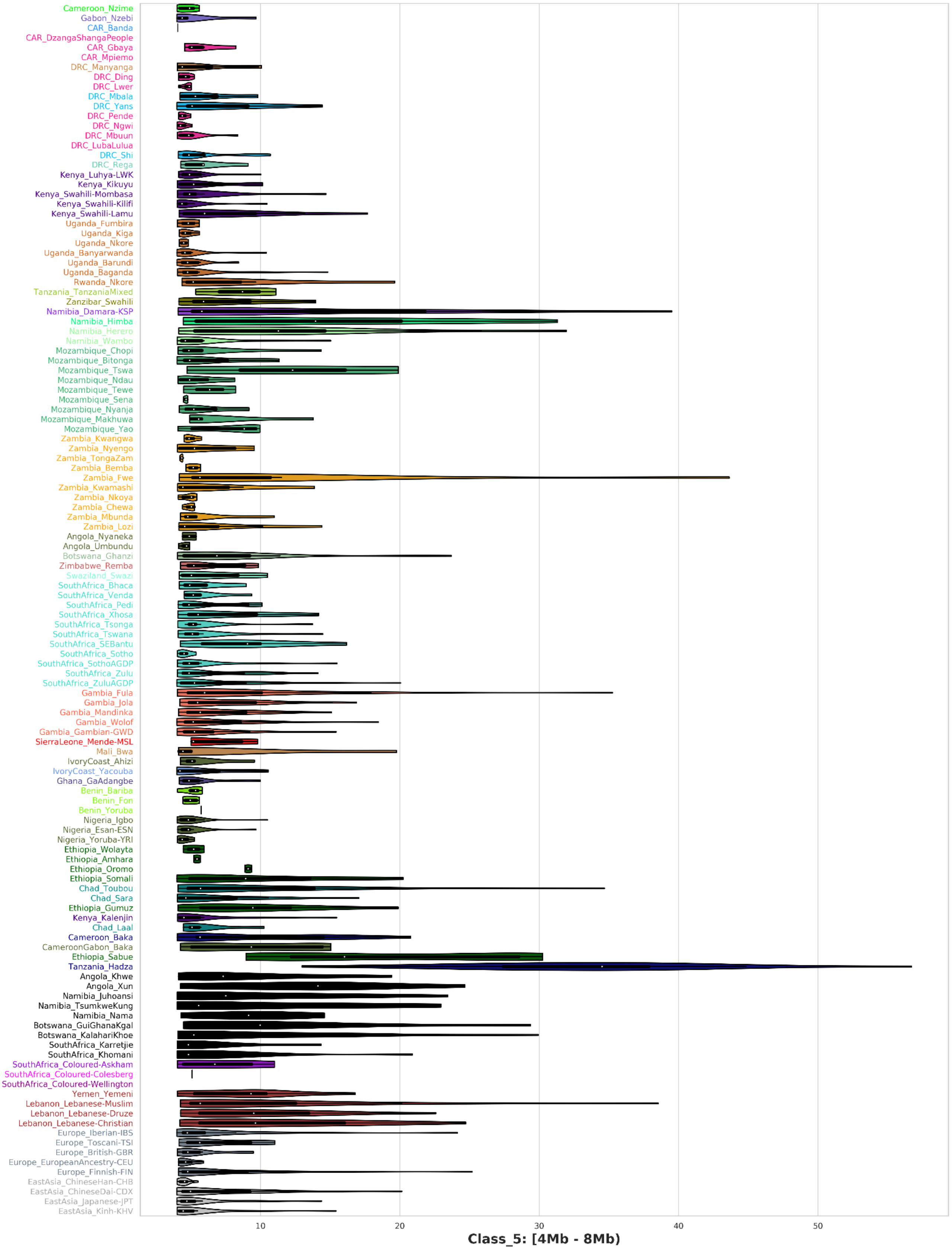

Supplementary Fig. 46 | Violin plots for category 5 of ROH length.
Category 5 of ROH length for segments longer than 4.0 Mb and shorter than 8.0 Mb in populations included in the AfricanNeo dataset (see mean and +/-SD values in Supplementary Table 7).

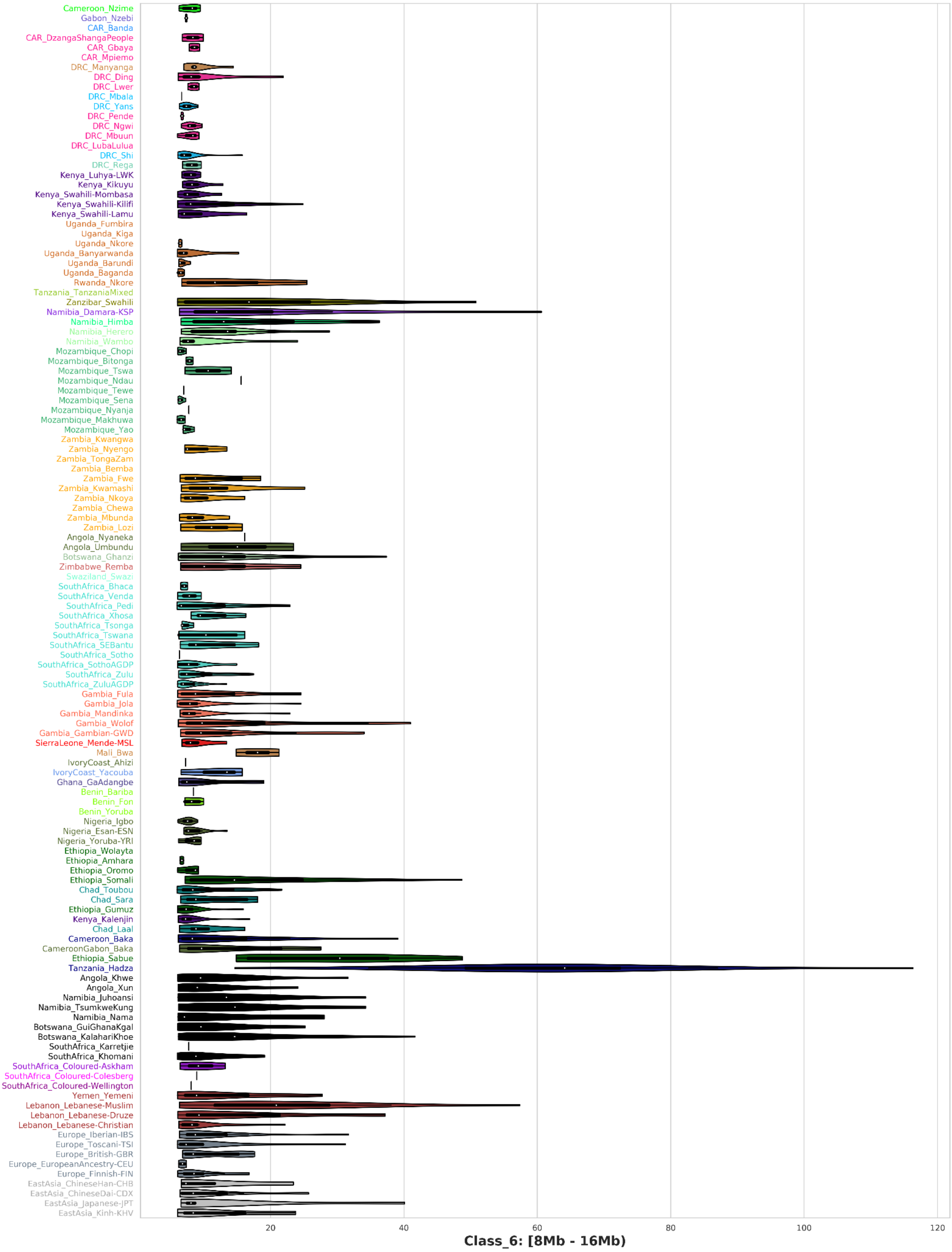

Supplementary Fig. 47 | Violin plots for category 6 of ROH length.
Category 6 of ROH length for segments longer than 8.0 Mb and shorter than 16.0 Mb in populations included in the AfricanNeo dataset (see mean and +/-SD values in Supplementary Table 7).

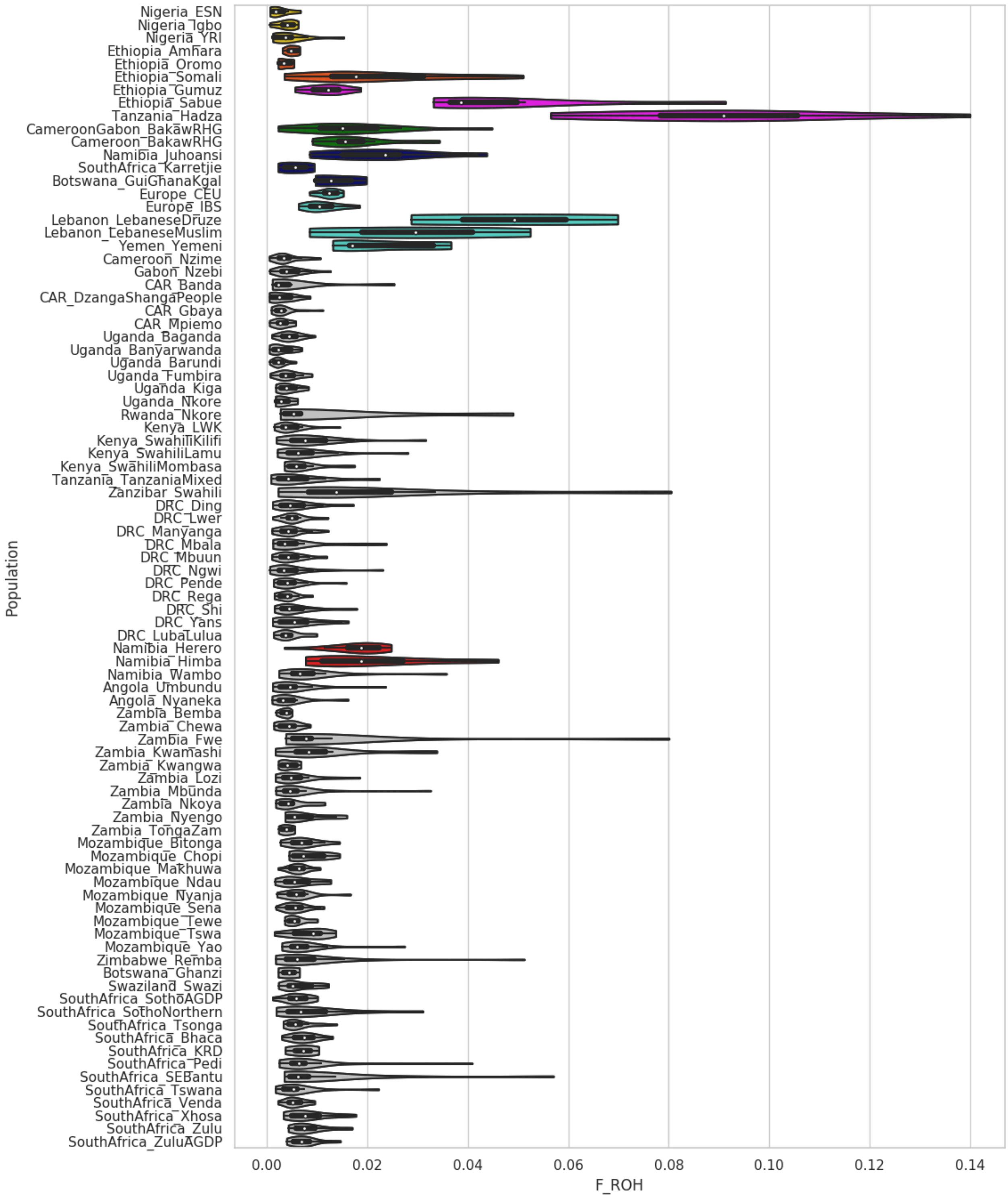

Supplementary Fig. 48 | F_{ROH} estimates after masking the AfricanNeo dataset.

Figure showing estimated ROH-based genomic inbreeding coefficient (F_{ROH}) of selected populations included in the dataset after masking and imputation of the AfricanNeo dataset. We highlight Himba and Herero populations in red due to their higher F_{ROH} values (on average 0.019 ±0.012SD and 0.019 ±0.007SD, respectively.

3.7. Estimated effective population sizes and demographic founder events

a

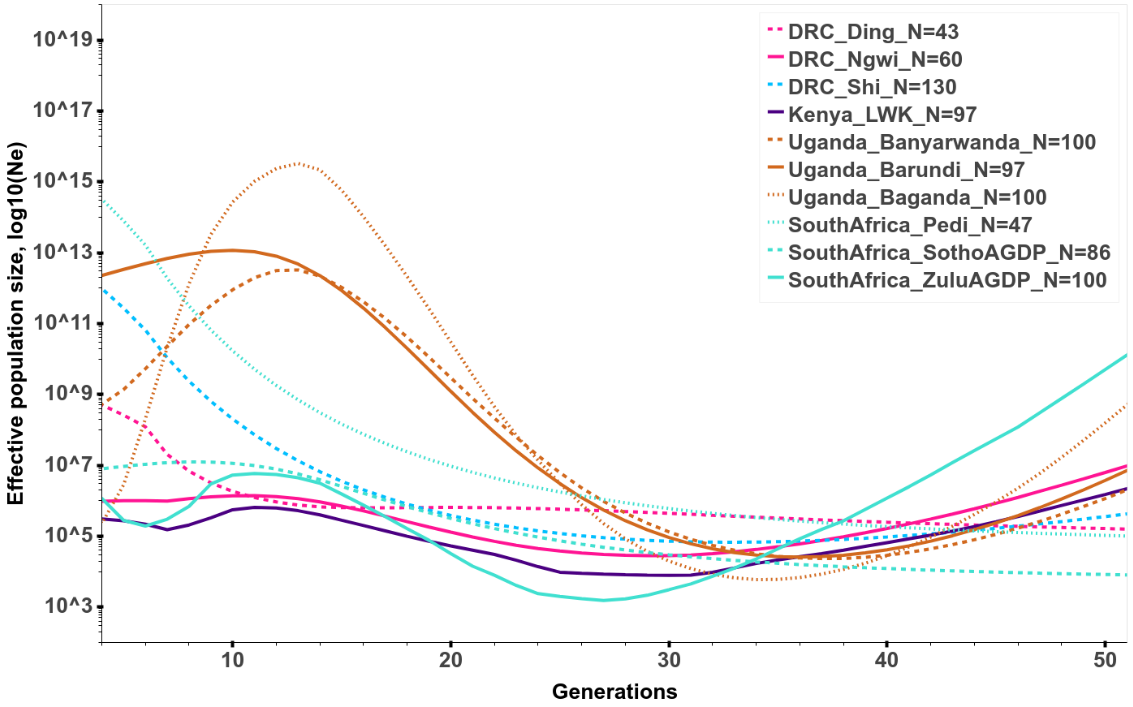

b

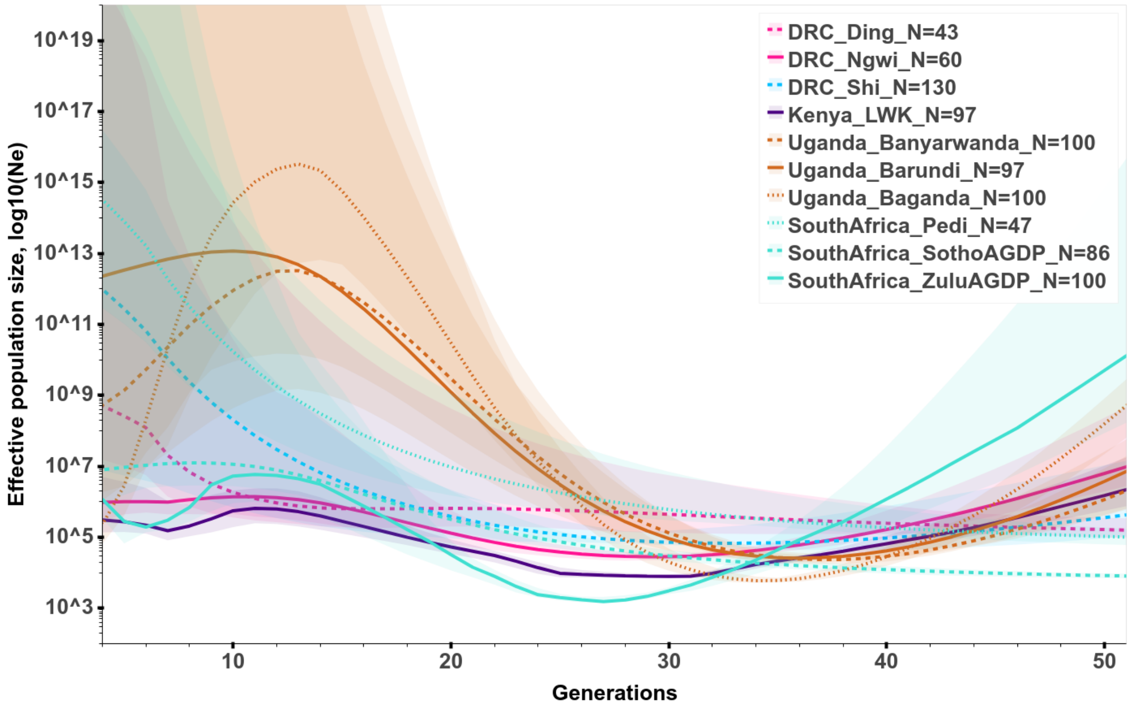

Supplementary Fig. 49 | IBDNe results for selected BSP included in the AfricanNeo dataset. Figure showing **a**, estimated effective population sizes (N_e) in selected BSP (with sample sizes larger than 40 samples) estimated using IBDNe for the last 50 generations. **b**, Confidence intervals were included in the bottom panel. The shaded area is between the lower and the upper 95% confidence interval. To better visualize the results of each population, interactive plots are available at Github [113] (Suppl_Fig_49[a–b]_IBDNe.html).

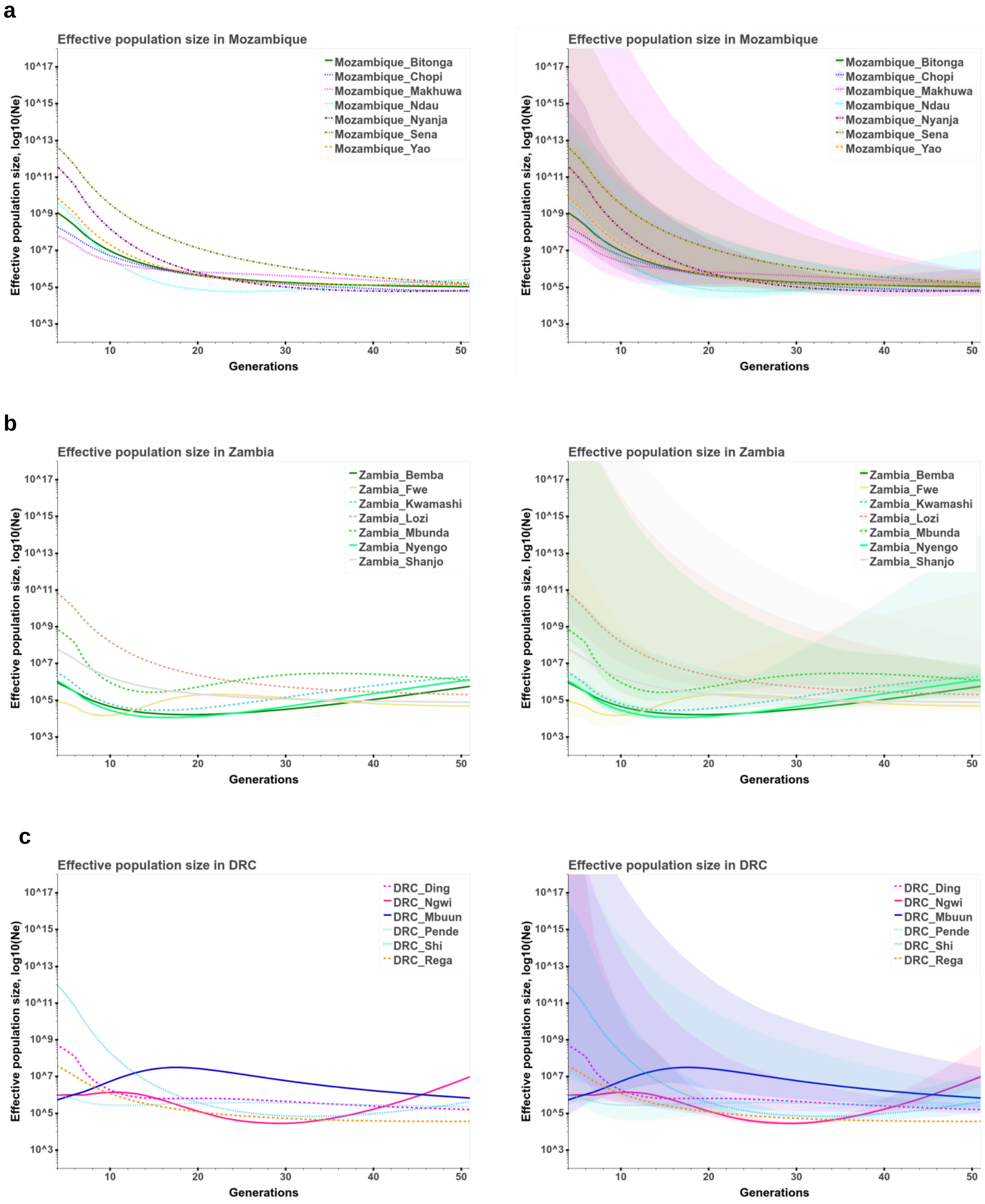

a
Effective population size in Mozambique
Mozambique_Bitonga
Mozambique_Chopi
Mozambique_Makhuwa
Mozambique_Ndau
Mozambique_Nyanja
Mozambique_Sena
Mozambique_Yao
Effective population size, log10(Ne)
10^17
10^15
10^13
10^11
10^9
10^7
10^5
10^3
10
20
30
40
50
Generations
b
Effective population size in Zambia
Zambia_Bemba
Zambia_Fwe
Zambia_Kwamashi
Zambia_Lozi
Zambia_Mbunda
Zambia_Nyengo
Zambia_Shanjo
c
Effective population size in DRC
DRC_Ding
DRC_Ngwi
DRC_Mbuun
DRC_Pende
DRC_Shi
DRC_Rega

d

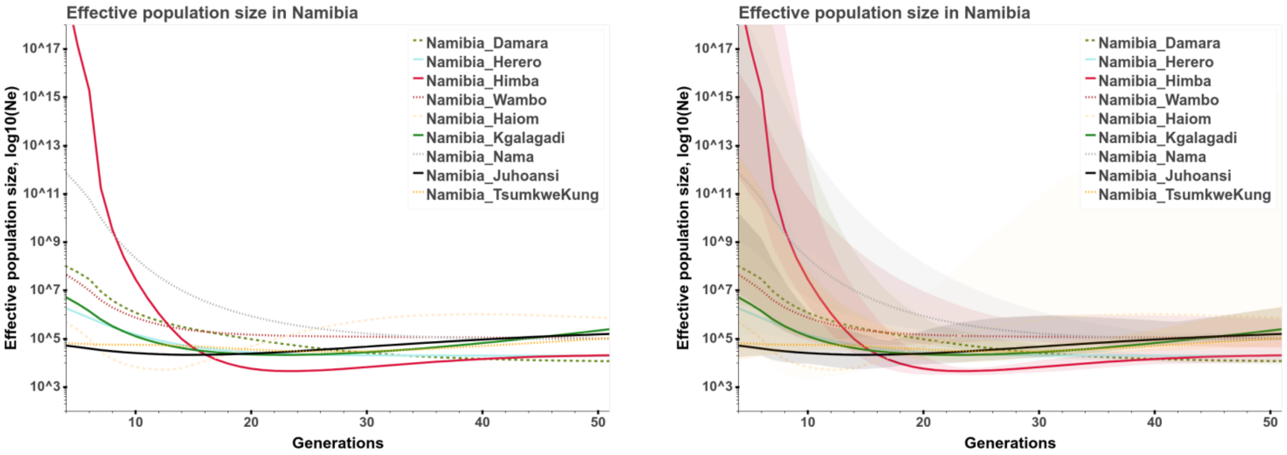

e

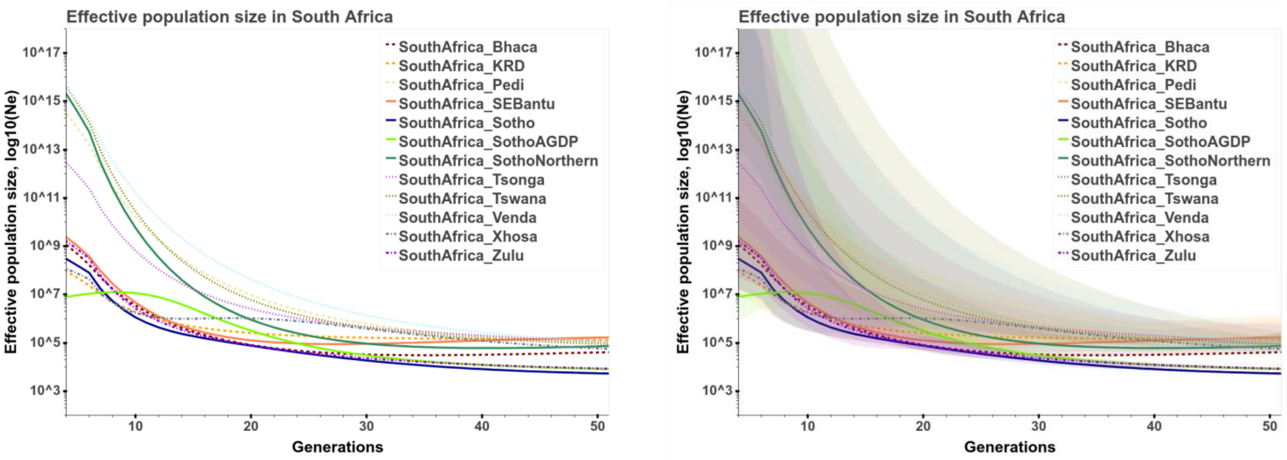

f

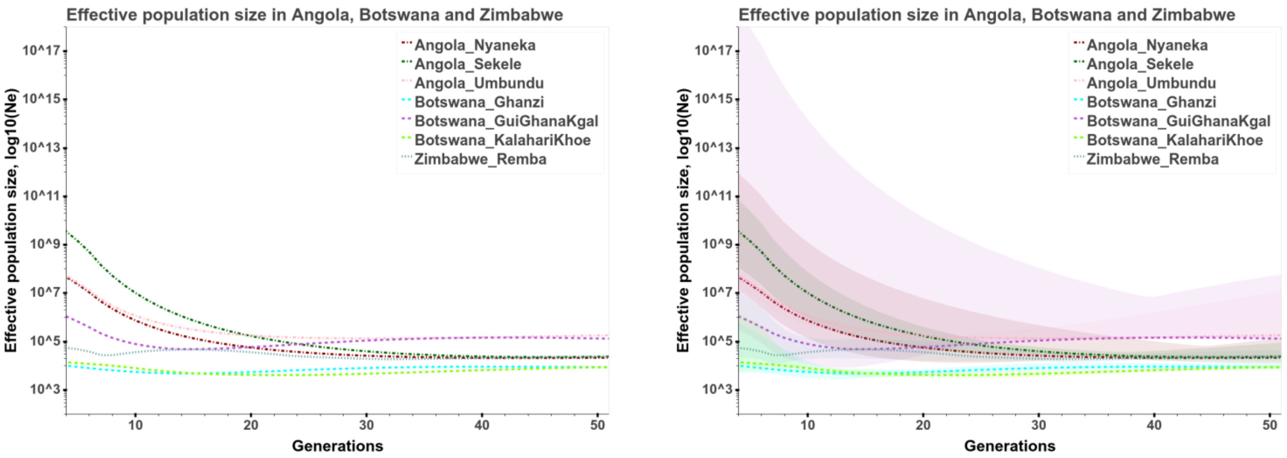

Supplementary Fig. 50 | IBDNe results for BSP from African regions.

Figure showing estimated effective population sizes (N_e) of BSP from six selected African regions estimated using IBDNe for the last 50 generations: **a**, Mozambique, **b**, Zambia, **c**, DRC, **d**, Namibia, **e**, South Africa, and **f**, Angola, Botswana and Zimbabwe. Confidence intervals were included in the right column. The shaded area is between the lower and the upper 95% confidence interval. We caution against the interpretation of IBDNe results for populations with low population sizes. A plot with IBDNe results of populations with high sample sizes (>40) is included in figure Supplementary Fig. 47. Admixture might also influence IBDNe results and we illustrate the effect of admixture in selected BSP in Supplementary Fig. 51.

a

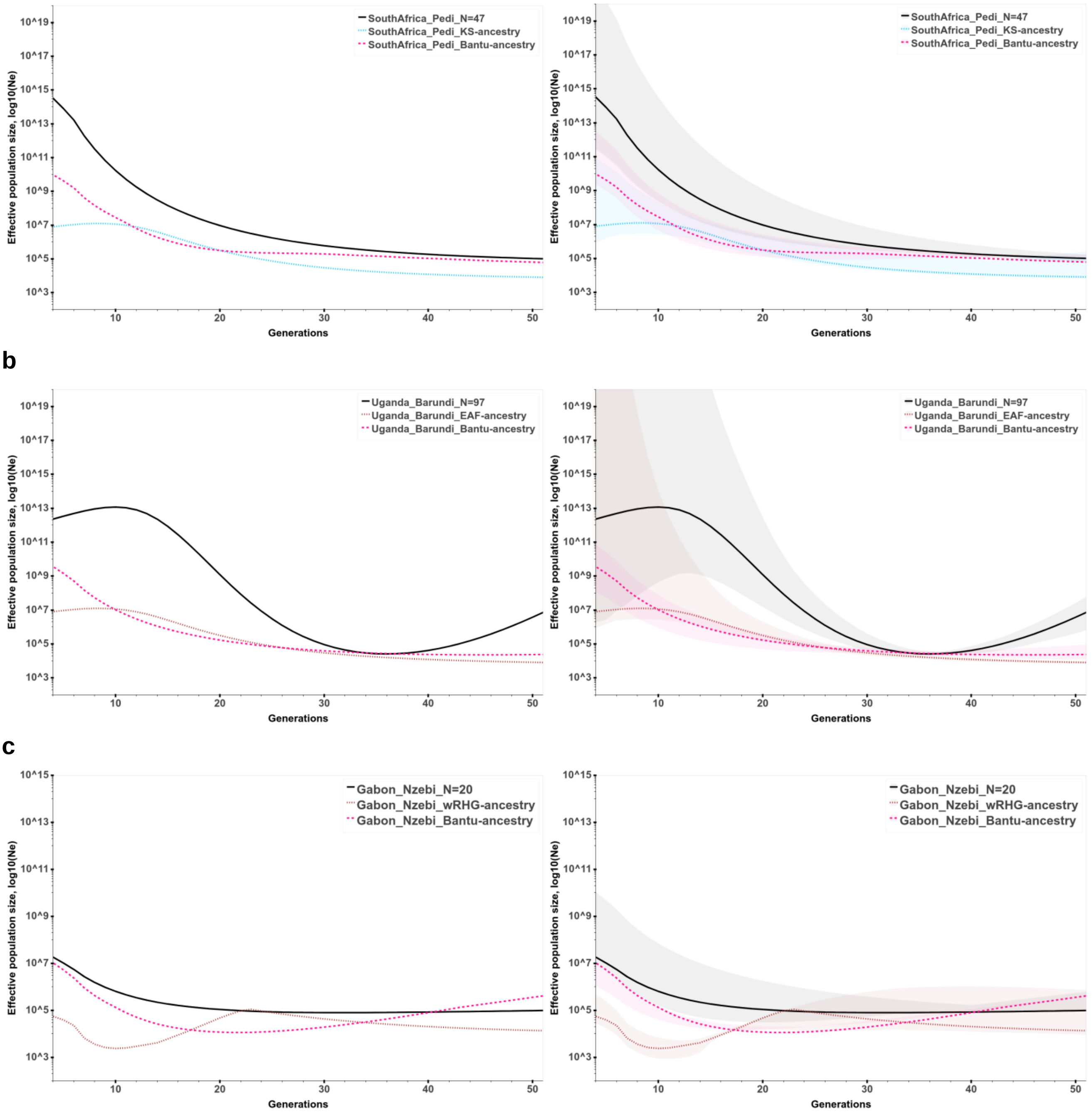

Supplementary Fig. 51 | Ancestry-specific IBDNe results for selected BSP.
Estimated ancestry-specific effective population sizes (Ne) of selected BSP after using AS-IBDNe. Figure showing the results for **a**, Pedi population from South Africa; **b**, Barundi population from Uganda; and **c**, Nzebi population from Gabon. The lines show estimated effective population sizes for the full population (black line) and for each ancestry (West-Central-Africa-related ancestry in pink, Khoe-San-related ancestry in blue; and East-African ancestry in brown). The shaded areas in the plots to the right show 95% bootstrap confidence intervals of each estimation.

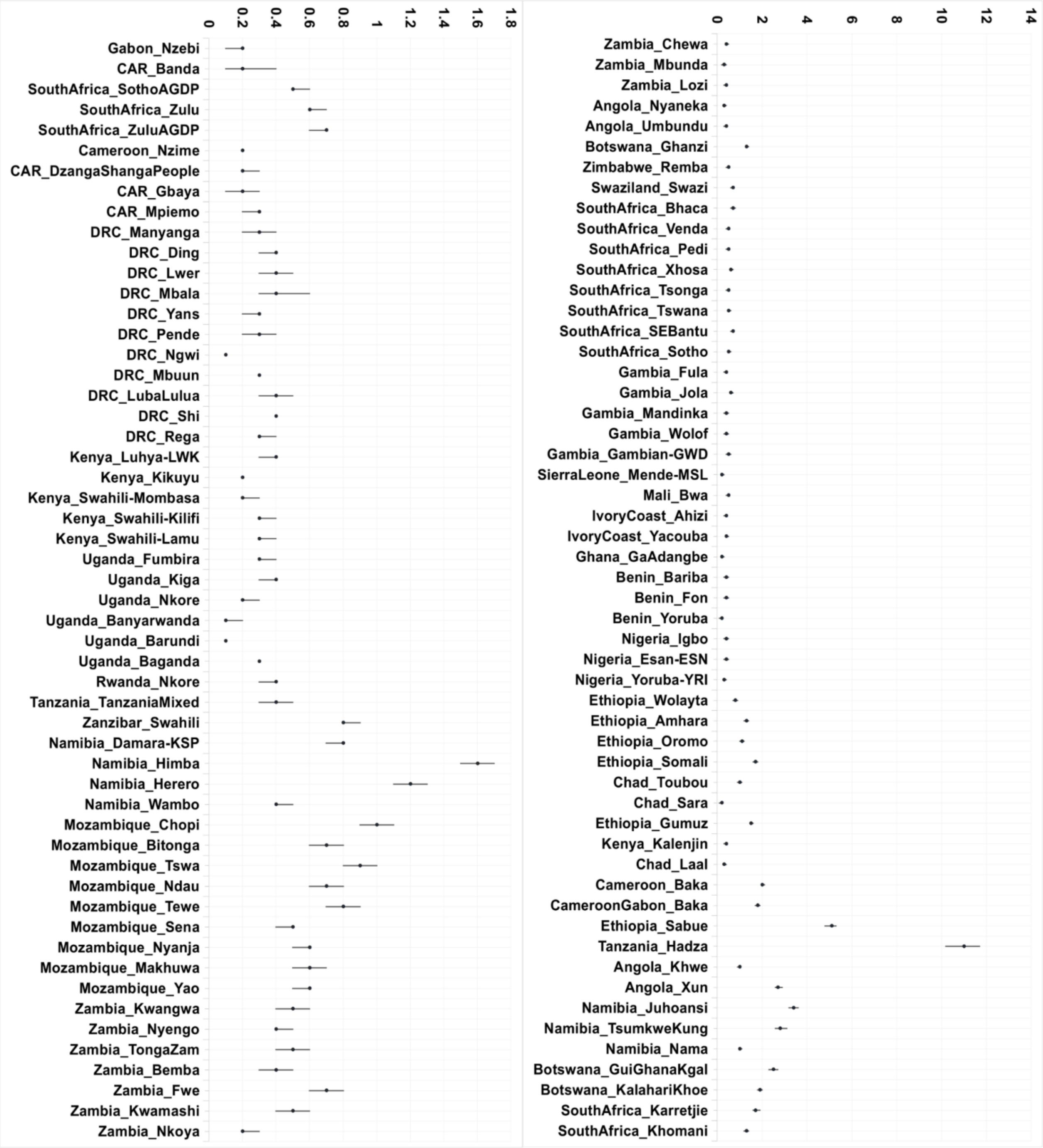

Supplementary Fig. 52 | Intensity of founder events in sub-Saharan African populations.
Figure showing the intensity of the founder event (I_f in %, each dot; and 95%CI, each line) calculated for sub-Saharan African populations included in the AfricanNeo dataset using ASCEND analysis. To better see the results, the plot was divided into two plots, and the X-axes have different ranges in each plot. Further details of all the results were included in Supplementary Table 8.

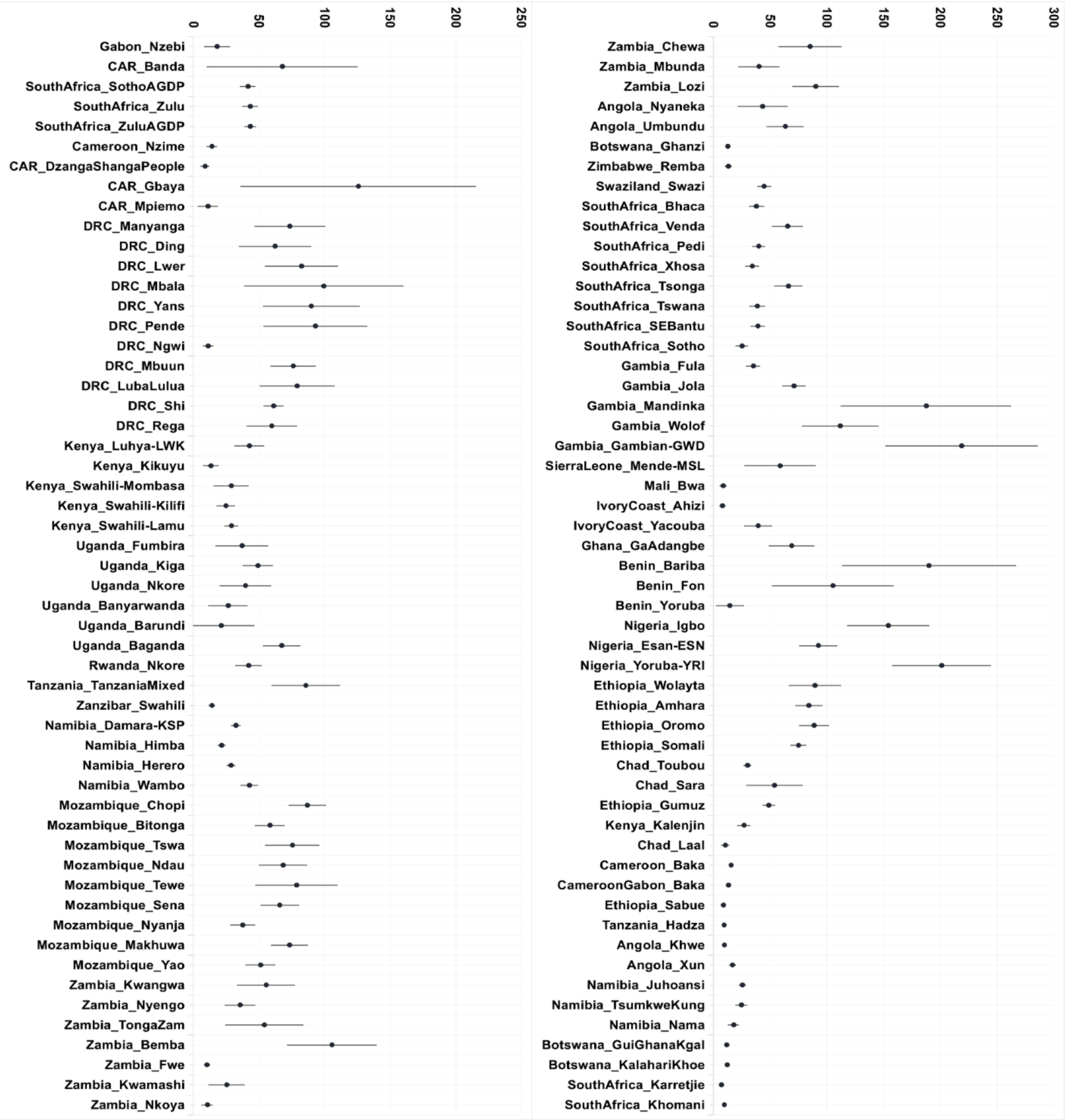

Supplementary Fig. 53 | Timing of founder ages in sub-Saharan African populations.
Figure showing the timing of the founder event (T_f in generations, each dot; and 95%CI, each line) calculated for sub-Saharan African populations included in the AfricanNeo dataset using ASCEND analysis. To better see the results, the plot was divided into two plots and the x-axes have different ranges in each plot. Further details of the results of each population were included in Supplementary Table 8.

3.8. Patterns of isolation-by-distance for the unmasked and masked datasets

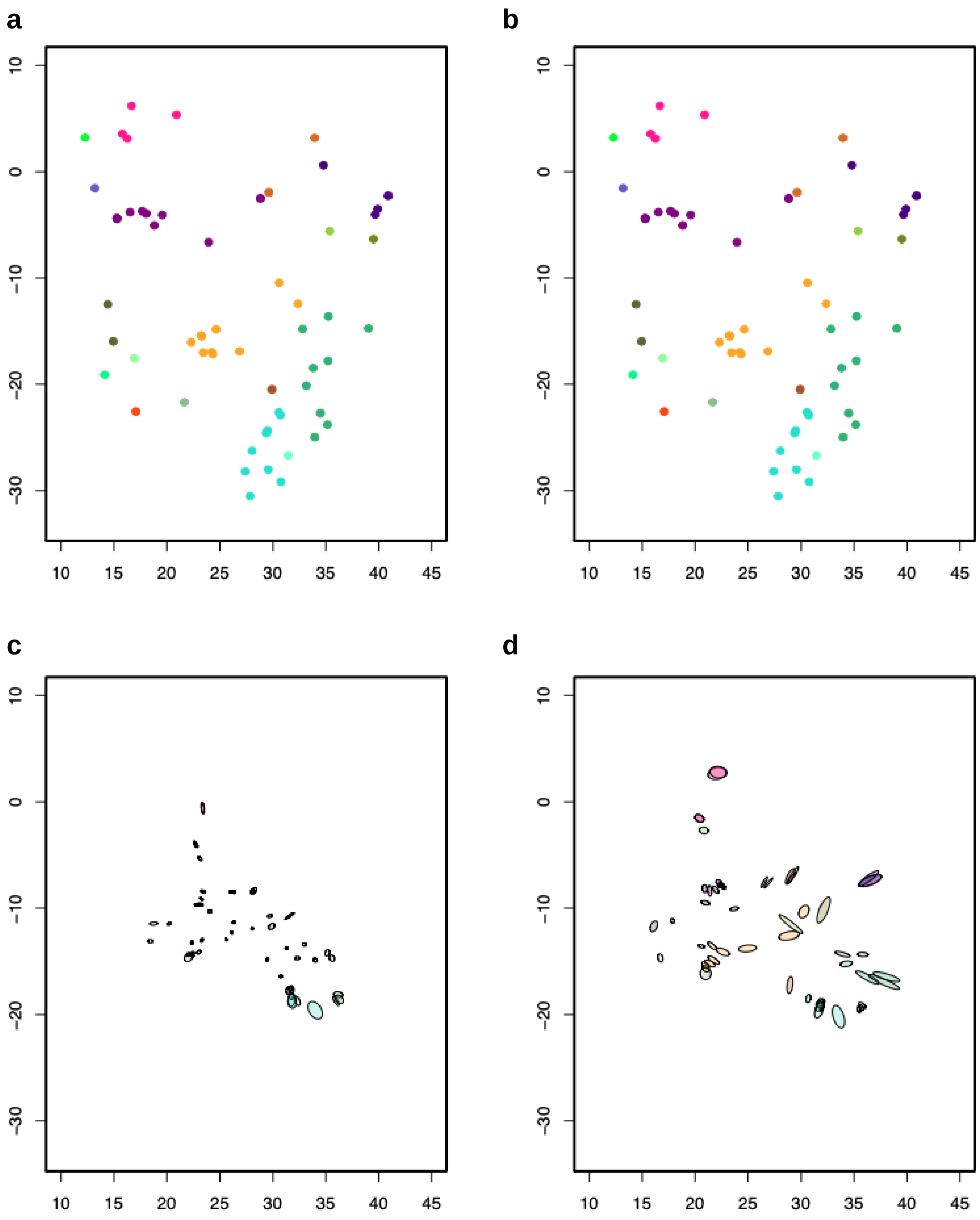

Supplementary Fig. 54 | SpaceMix for the unmasked dataset with no population text overlays. Figure showing SpaceMix tests for the unmasked Only-BSP dataset, no population text overlays. X- and Y-axes are latitude and longitude in the geogenetic space, respectively. Panel showing the results of each IBD model, and the assumptions of each model were: **a**, No migration and no admixture; **b**, No migration but admixture; **c**, Migration but no admixture; and **d**, Both migration and admixture.

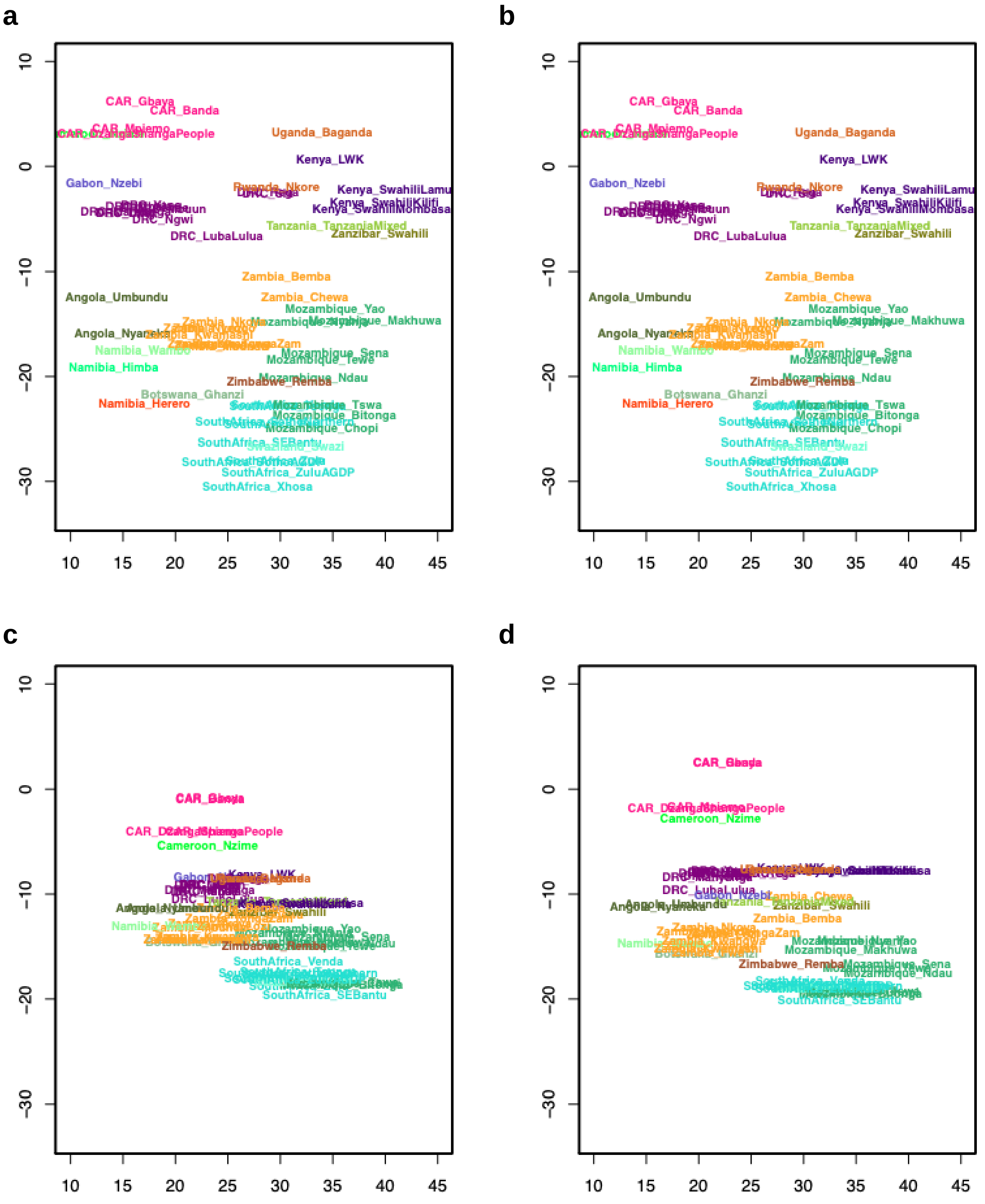

Supplementary Fig. 55 | SpaceMix for the unmasked dataset with text instead of dots.
Figure showing SpaceMix results for the unmasked Only-BSP dataset, text instead of dots. X- and Y-axes are latitude and longitude in the geogenetic space, respectively. Panel showing the results of each IBD model, and the assumptions of each model were: **a**, No migration and no admixture; **b**, No migration but admixture; **c**, Migration but no admixture; and **d**, Both migration and admixture.

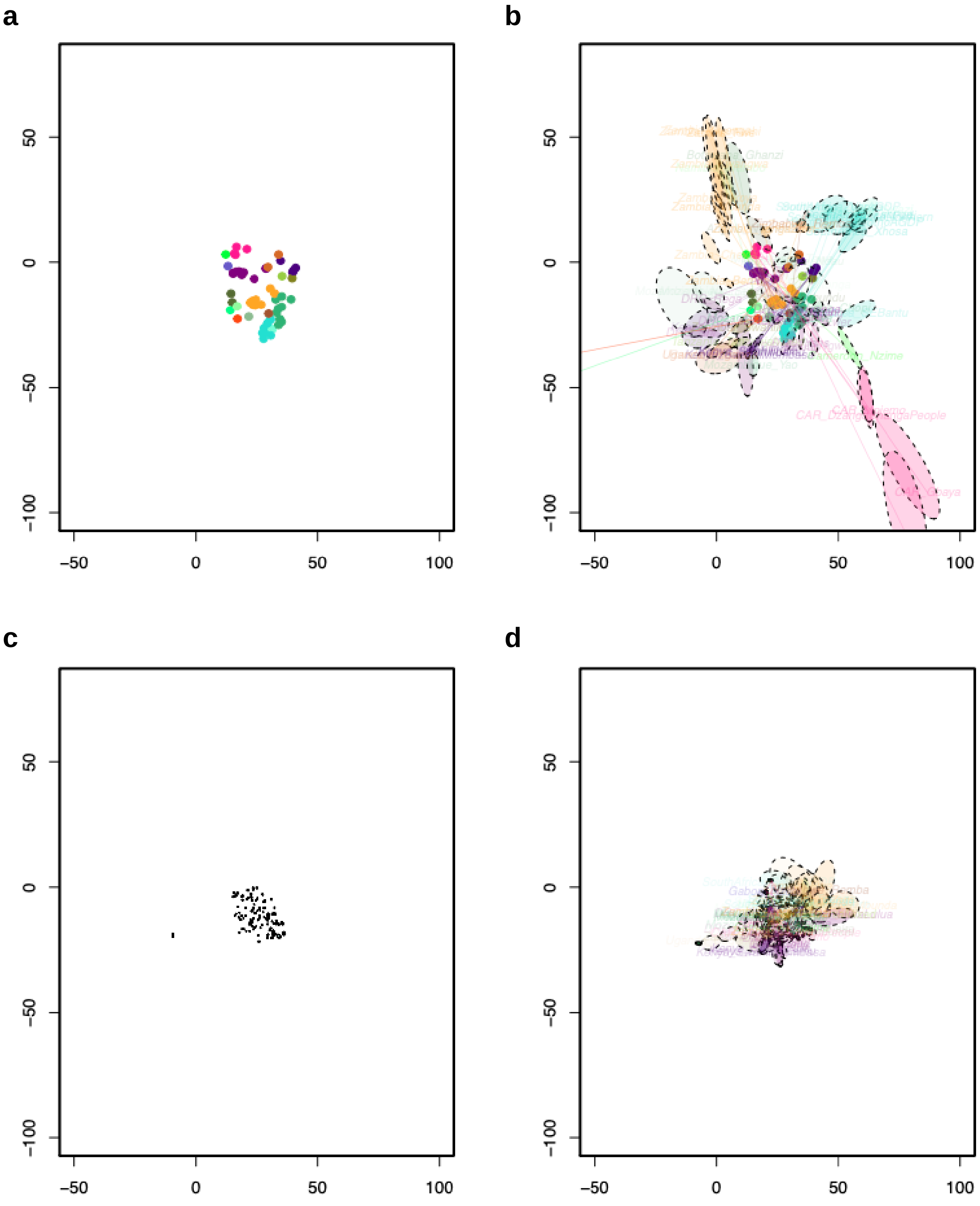

Supplementary Fig. 56 | SpaceMix for the unmasked dataset with sources of admixture. Figure showing SpaceMix tests for the unmasked Only-BSP dataset, sources of admixture were indicated with a dashed ellipsis. X- and Y-axes are latitude and longitude in the geogenetic space, respectively. Panel showing the results of each IBD model, and the assumptions of each model were: **a**, No migration and no admixture; **b**, No migration but admixture; **c**, Migration but no admixture; and **d**, Both migration and admixture.

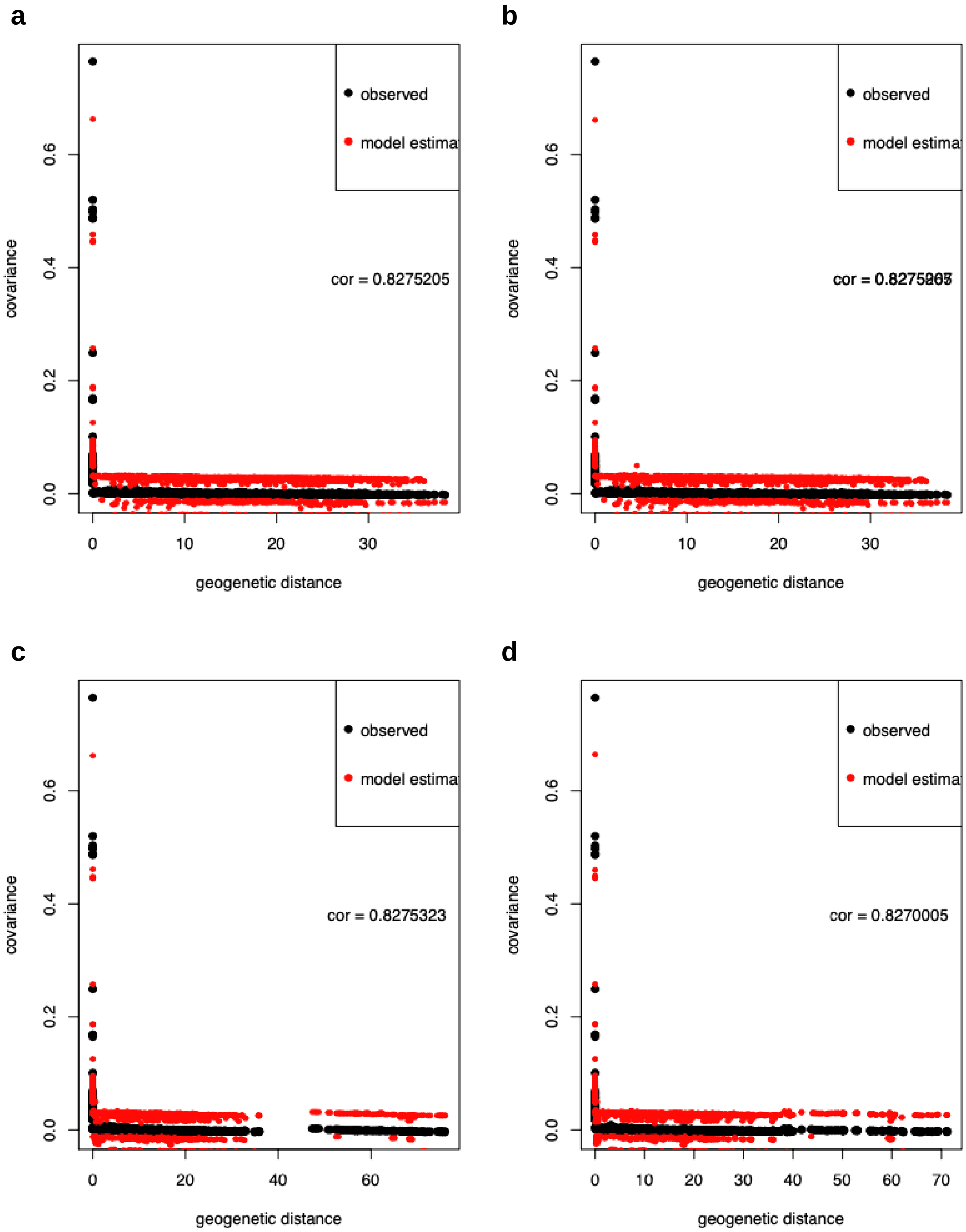

Supplementary Fig. 57 | SpaceMix for the unmasked dataset with correlations of each tested model.

Figure showing SpaceMix tests for the unmasked Only-BSP dataset, highlighting the correlation between the observed data and the data estimated from the IBD model. We computed the Pearson correlation between the two series (see the “cor” values on each plot). Panel showing the results of each IBD model, and the assumptions of each model were: **a**, No migration and no admixture; **b**, No migration but admixture; **c**, Migration but no admixture; and **d**, Both migration and admixture.

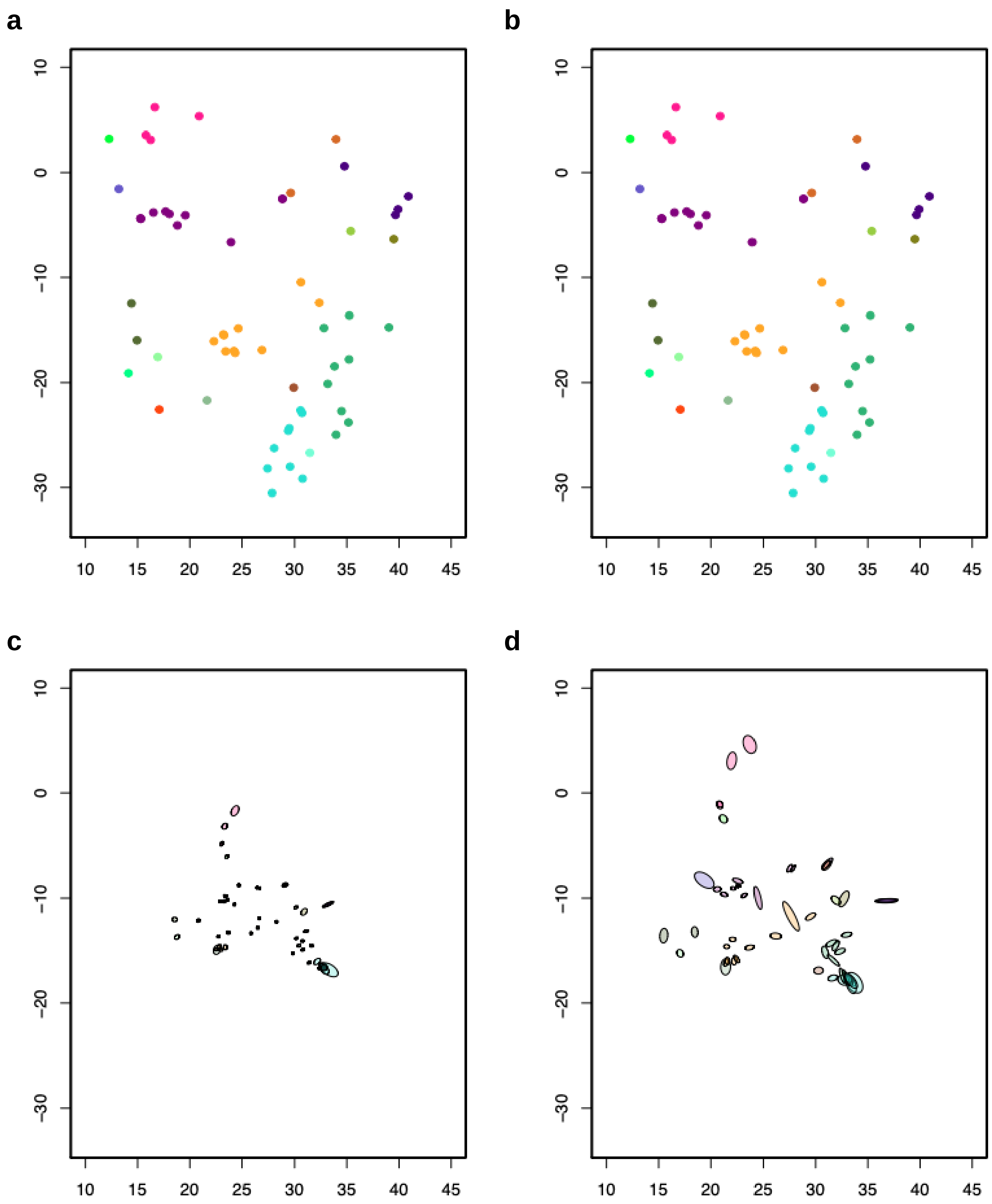

Supplementary Fig. 58 | SpaceMix for the masked dataset with no population text overlays. Figure showing SpaceMix tests for the masked and imputed Only-BSP dataset. X- and Y-axes are latitude and longitude in the geogenetic space, respectively. Panel showing the results of each IBD model, and the assumptions of each model were: **a**, No migration and no admixture; **b**, No migration but admixture; **c**, Migration but no admixture; and **d**, Both migration and admixture.

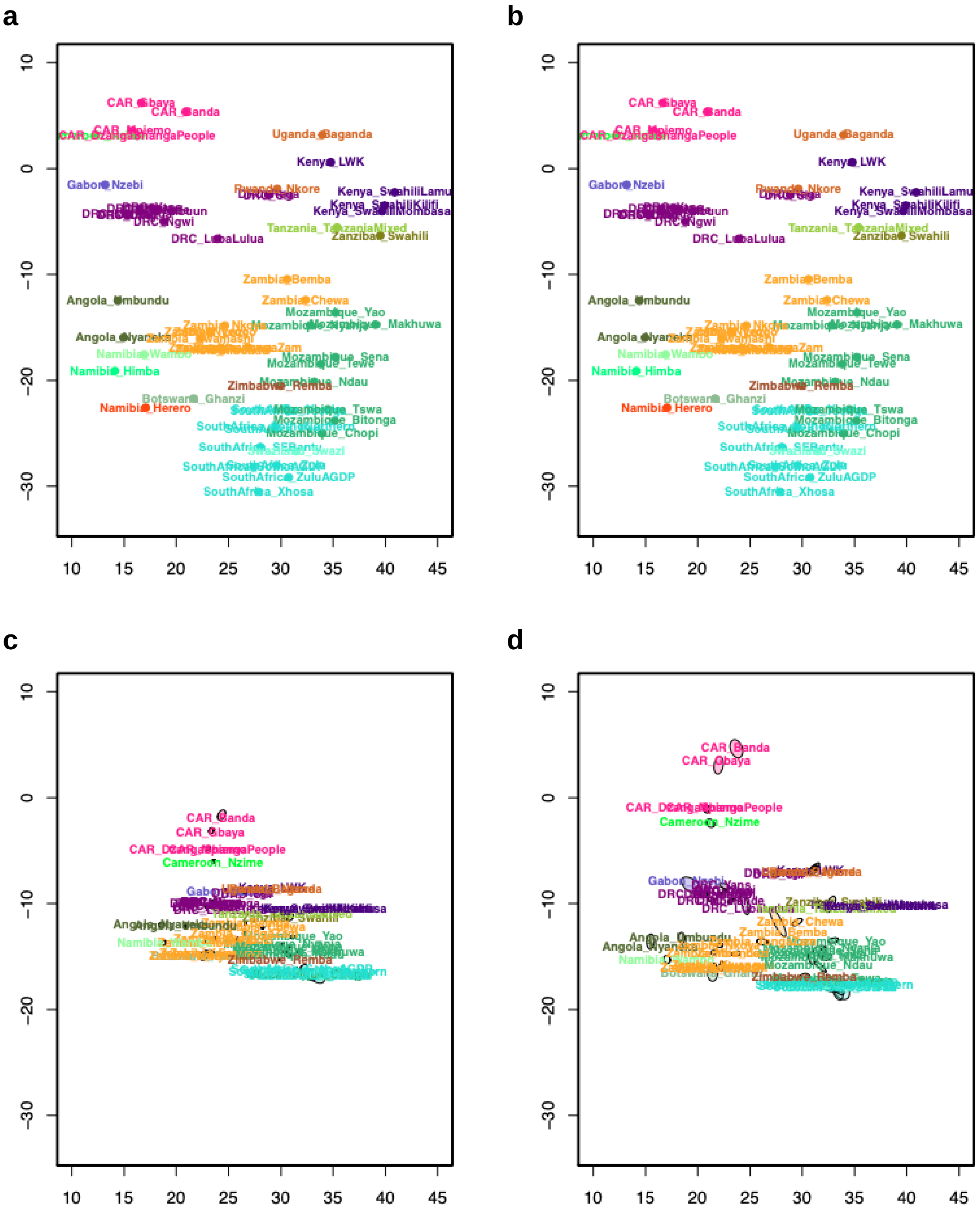

Supplementary Fig. 59 | SpaceMix for the masked dataset with text instead of dots.
Figure showing SpaceMix tests for the masked and imputed Only-BSP dataset with text overlay for each population. X- and Y-axes are latitude and longitude in geogenetic space respectively. Panel showing the results of each IBD model, and the assumptions of each model were; **a**, No migration and no admixture; **b**, No migration but admixture; **c**, Migration but no admixture; and **d**, Both migration and admixture.

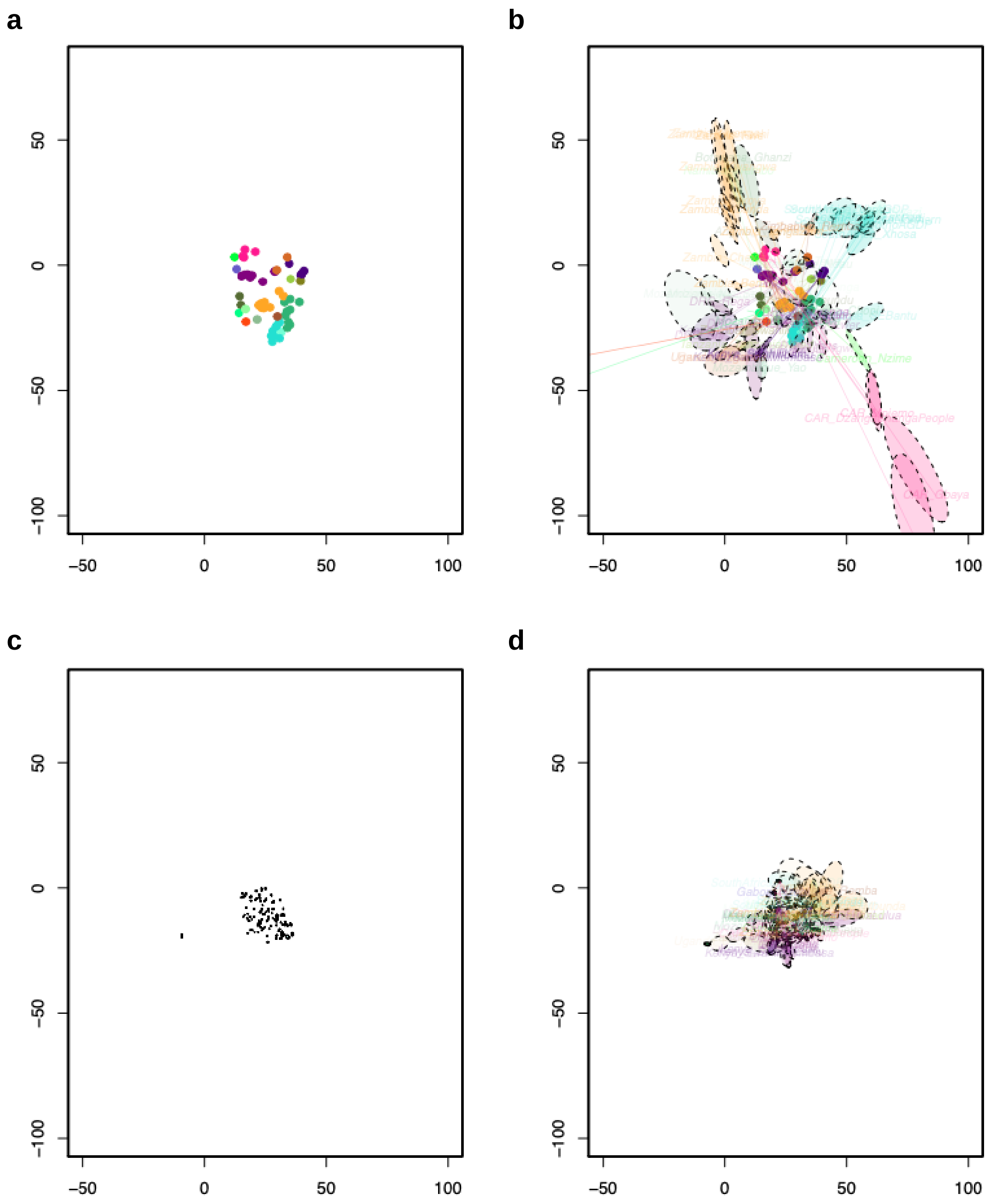

Supplementary Fig. 60 | SpaceMix for the masked dataset with sources of admixture. Figure showing SpaceMix tests for the masked and imputed Only-BSP dataset with sources of admixture indicated with a dashed ellipsis. X- and Y-axes are latitude and longitude in the geogenetic space, respectively. Panel showing the results of each IBD model, and the assumptions of each model were: **a**, No migration and no admixture; **b**, No migration but admixture; **c**, Migration but no admixture; and **d**, Both migration and admixture.

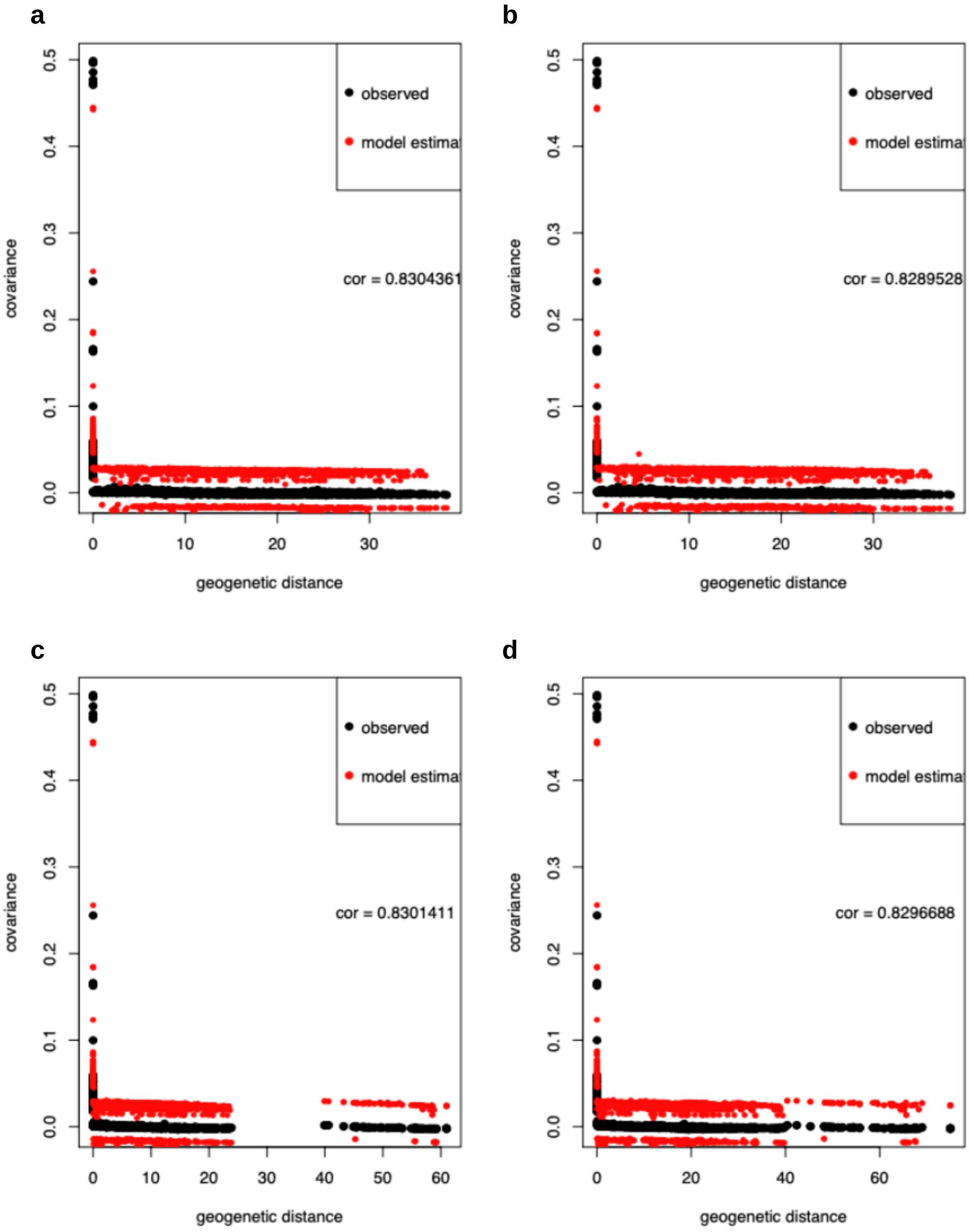

Supplementary Fig. 61 | SpaceMix for the masked dataset with correlations of each model. Figure showing SpaceMix tests for the masked and imputed Only-BSP dataset, highlighting the correlation between the observed data and the data estimated from the model. “Cor” means Pearson correlation between the two series. Panel showing the results of each IBD model, and the assumptions of each model were: **a**, No migration and no admixture; **b**, No migration but admixture; **c**, Migration but no admixture; and **d**, Both migration and admixture.

3.9. Patterns of haplotype diversity

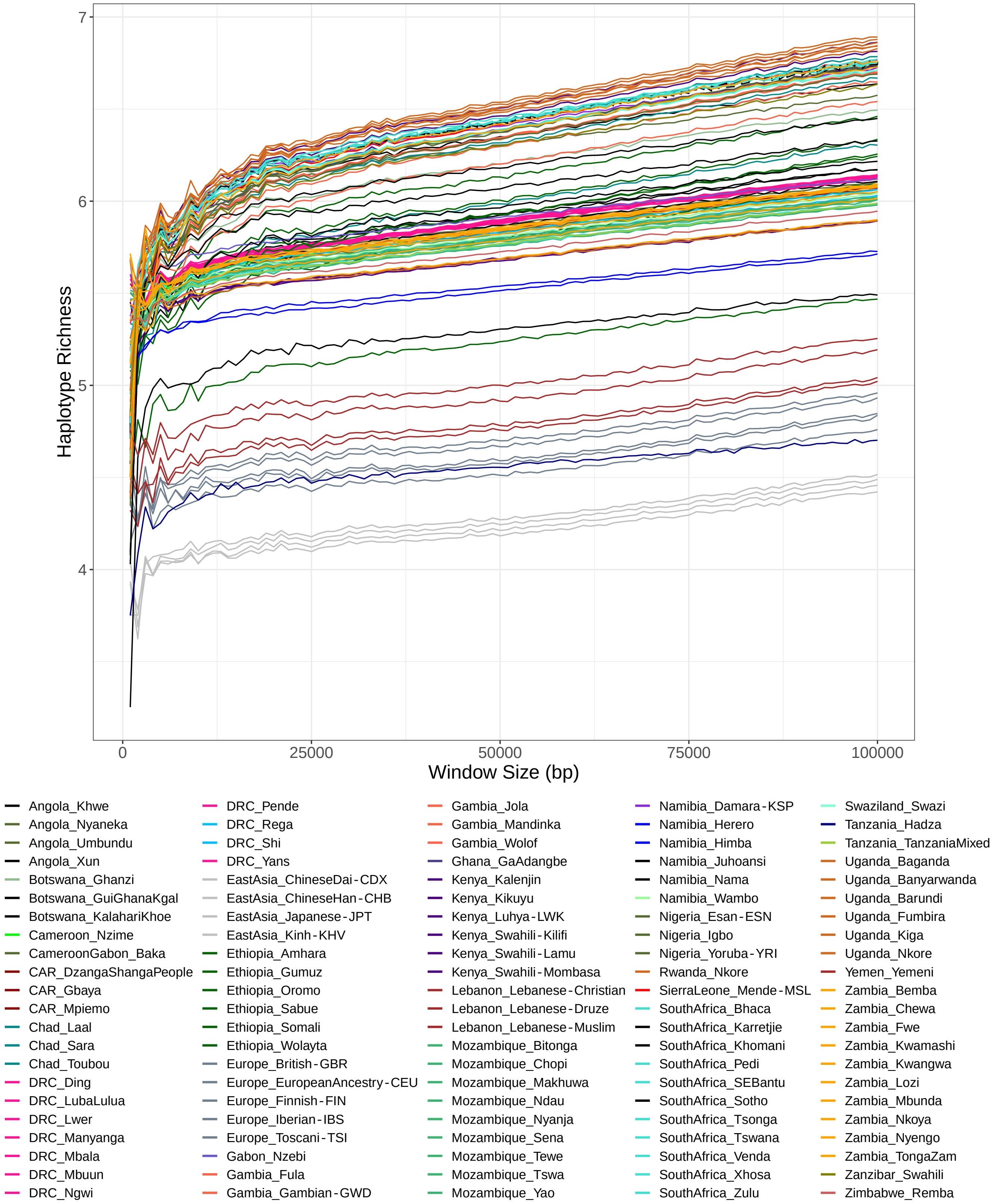

Supplementary Fig. 62 | Haplotype richness (HR) for the AfricanNeo dataset.
Figure showing estimated haplotype richness for the unmasked AfricanNeo dataset, HR for the y-axis and the window size (S) for the X-axis.

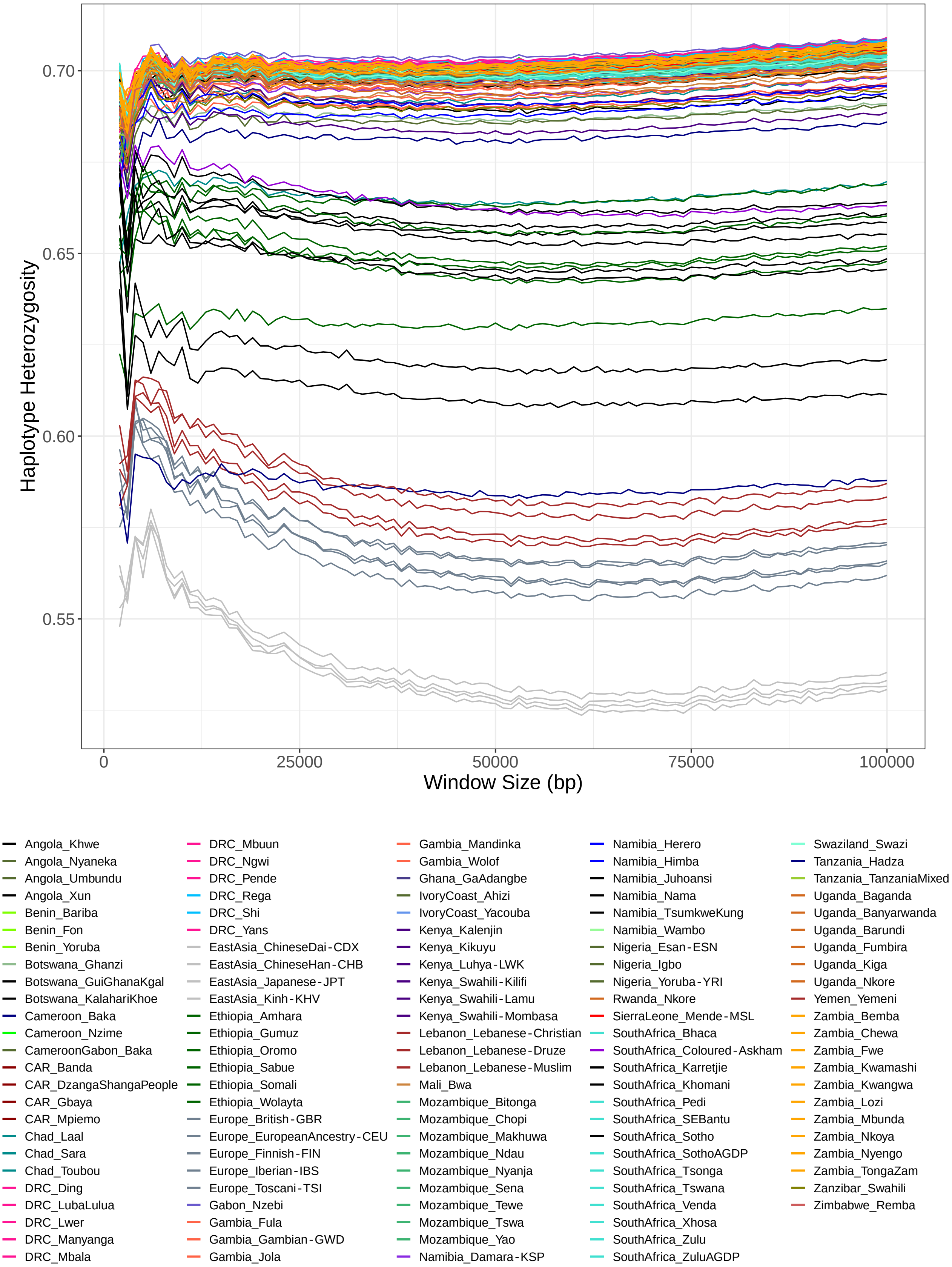

Supplementary Fig. 63 | Haplotype heterozygosity (HH) for the AfricanNeo dataset.
Figure showing estimated haplotype heterozygosity for the unmasked AfricanNeo dataset, HH on the y-axis and the window size (S) on the X-axis.

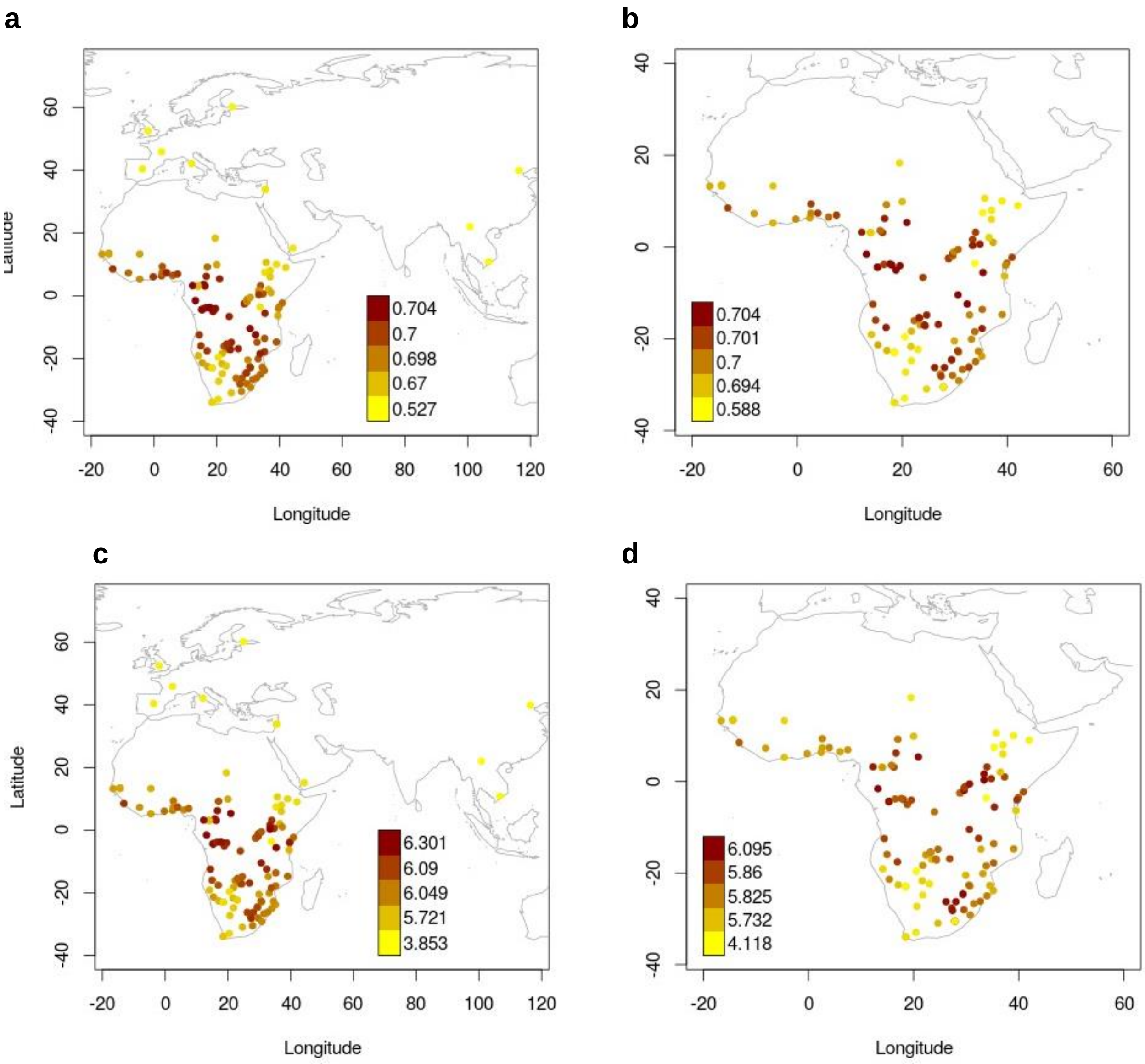

Supplementary Fig. 64 | Maps of haplotype diversity for the AfricanNeo data.
Geographical distribution of haplotype heterozygosity (HH) and haplotype richness (HR) estimated for a window size of (S) 50 kb on the basis of populations included in the unmasked AfricanNeo dataset. Figure showing: **a,** HH results for all studied populations included in the AfricanNeo dataset; **b**, HH results for sub-Saharan African populations included in the Only-African dataset; **c**, HR results for all studied populations included in the AfricanNeo dataset; and **d,** HR results for sub-Saharan African populations included in the Only-African dataset.

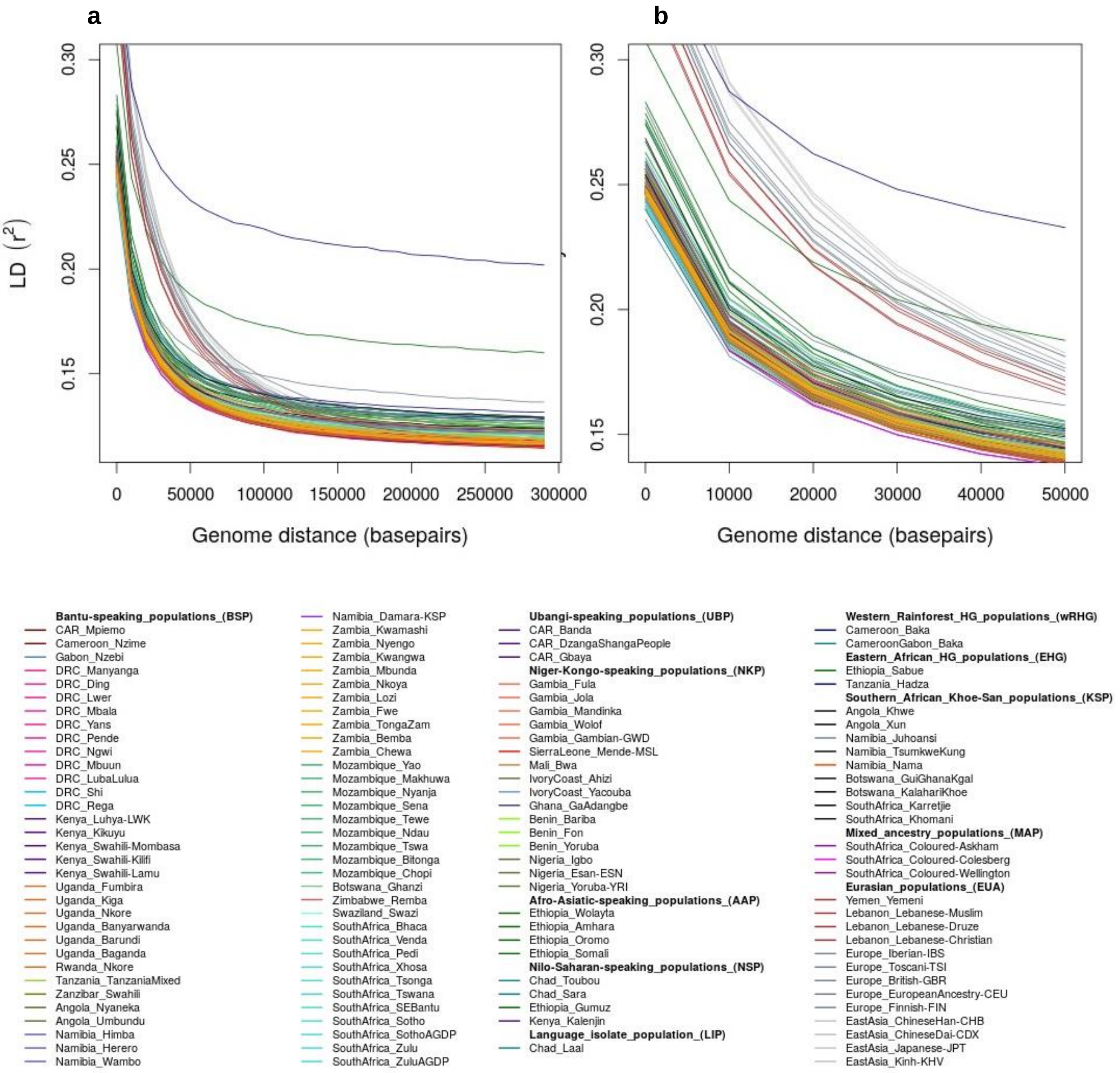

Supplementary Fig. 65 | Linkage-disequilibrium decay of the unmasked AfricanNeo dataset. Figure showing **a**, the patterns of LD-decay with genomic distance for all the populations included in the unmasked AfricanNeo dataset (124 African and Eurasian populations). LD-decay figure was built from the mean value of r^2 for pairs of sites in 30-distance bins. Eurasian populations have lower long-distance LD than some African populations. **b**, Figure after zooming into a short distance of LD decay of up to 50 kb.

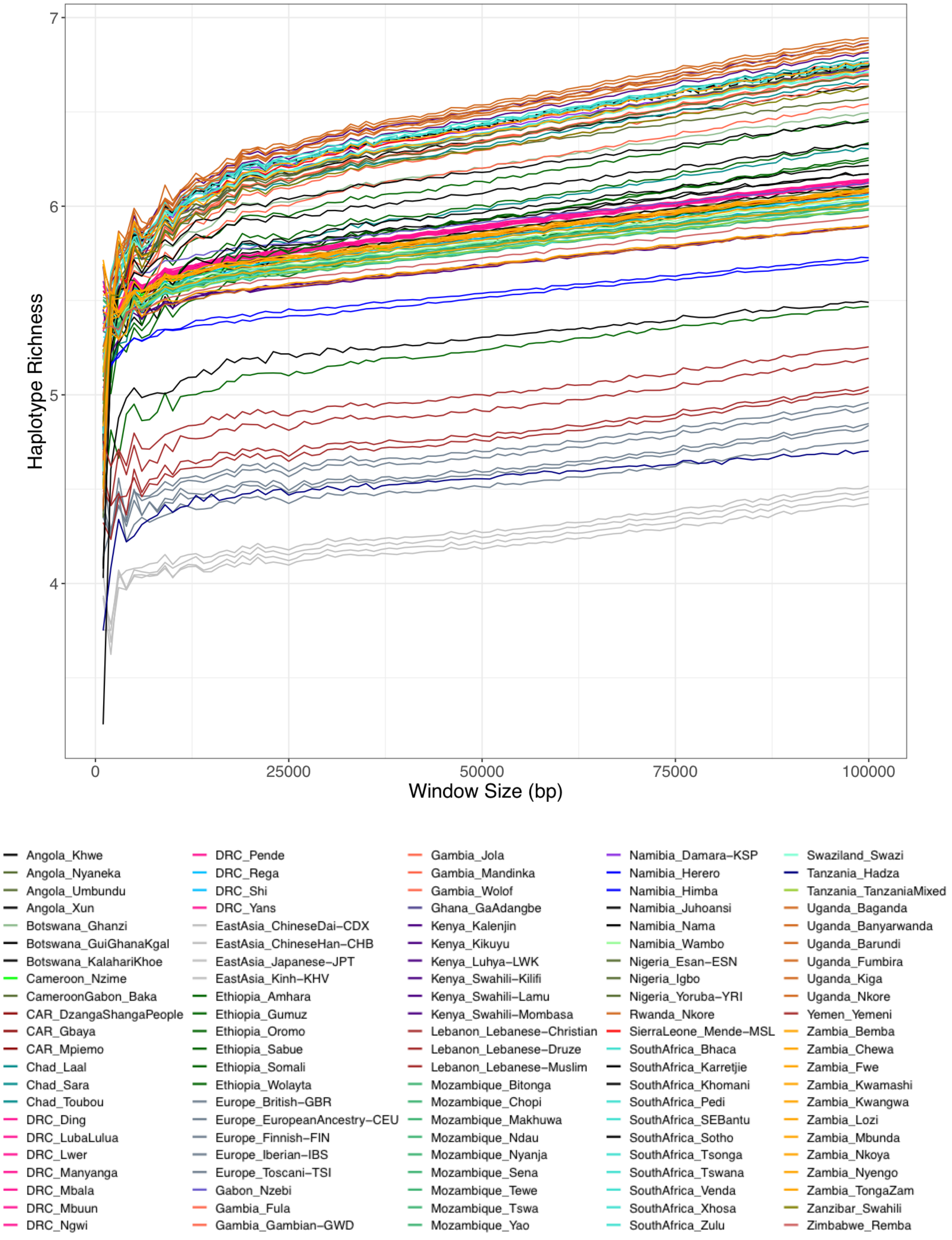

Supplementary Fig. 66 | Haplotype richness (HR) with masked AfricanNeo dataset.
Figure showing estimated haplotype richness with masked data of BSP and unmasked data of Eurasian populations included in the AfricanNeo dataset, HH on the y-axis and the window size (S) on the x-axis.

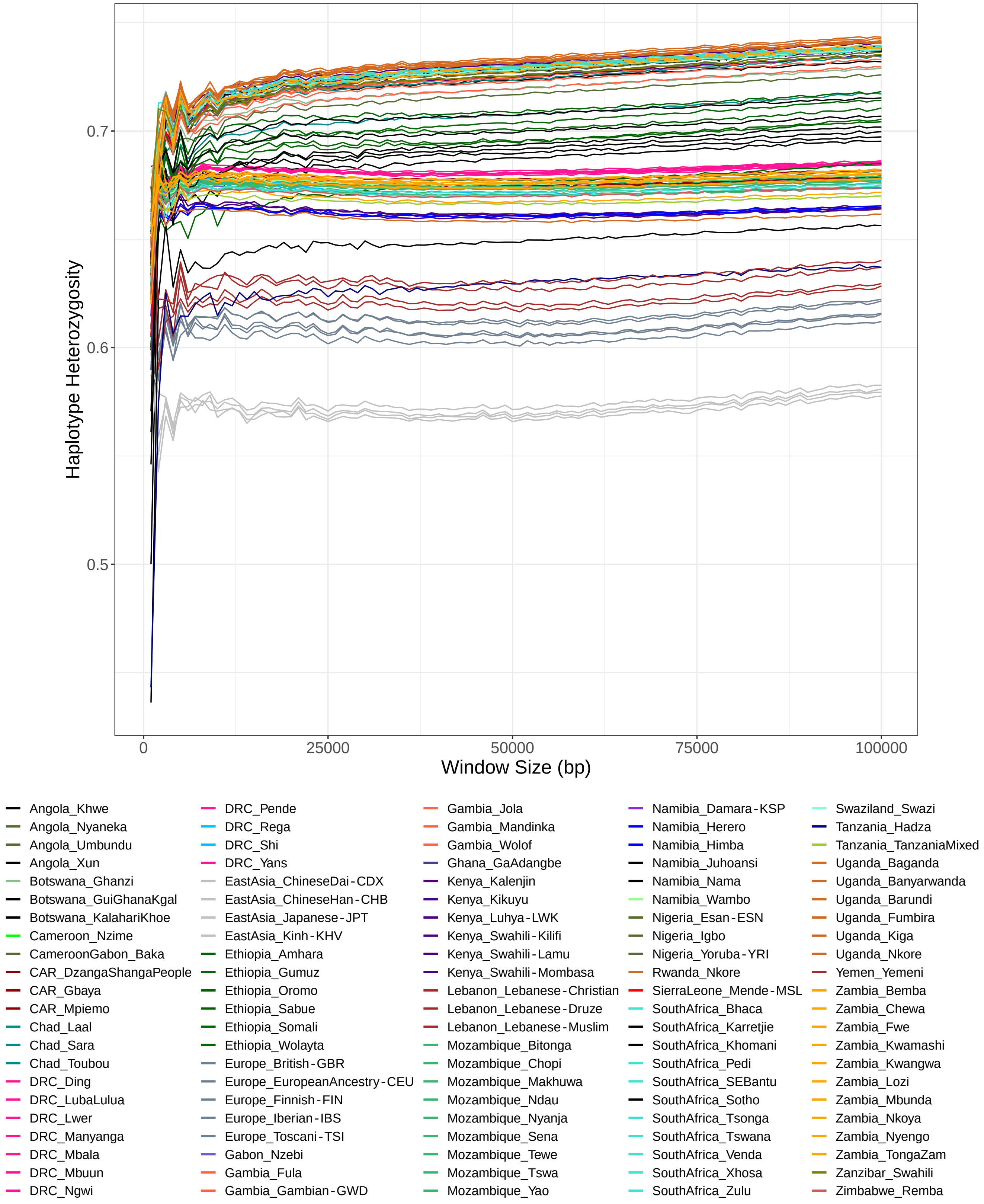

Supplementary Fig. 67 | Haplotype heterozygosity (HH) with masked AfricanNeo dataset. Figure showing estimated haplotype heterozygosity (HH) with masked data of BSP and unmasked data of Eurasian populations included in the AfricanNeo dataset, HH on the y-axis and the window size (S) on the x-axis.

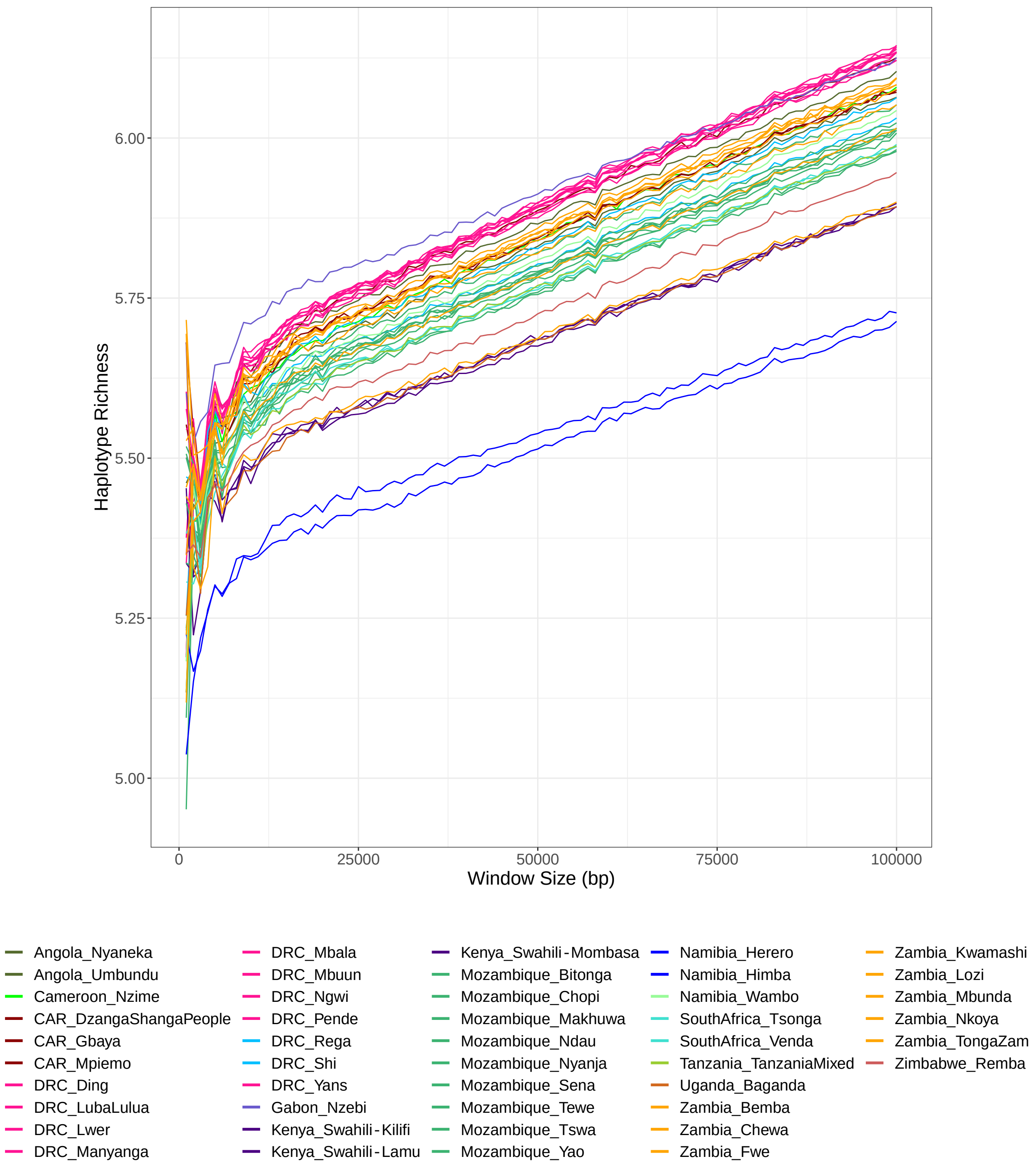

Supplementary Fig. 68 | Haplotype richness (HR) for the masked Only-BSP dataset.
Figure showing estimated haplotype richness for populations included in the masked Only-BSP dataset, HR on the y-axis and the window size (S) on the x-axis.

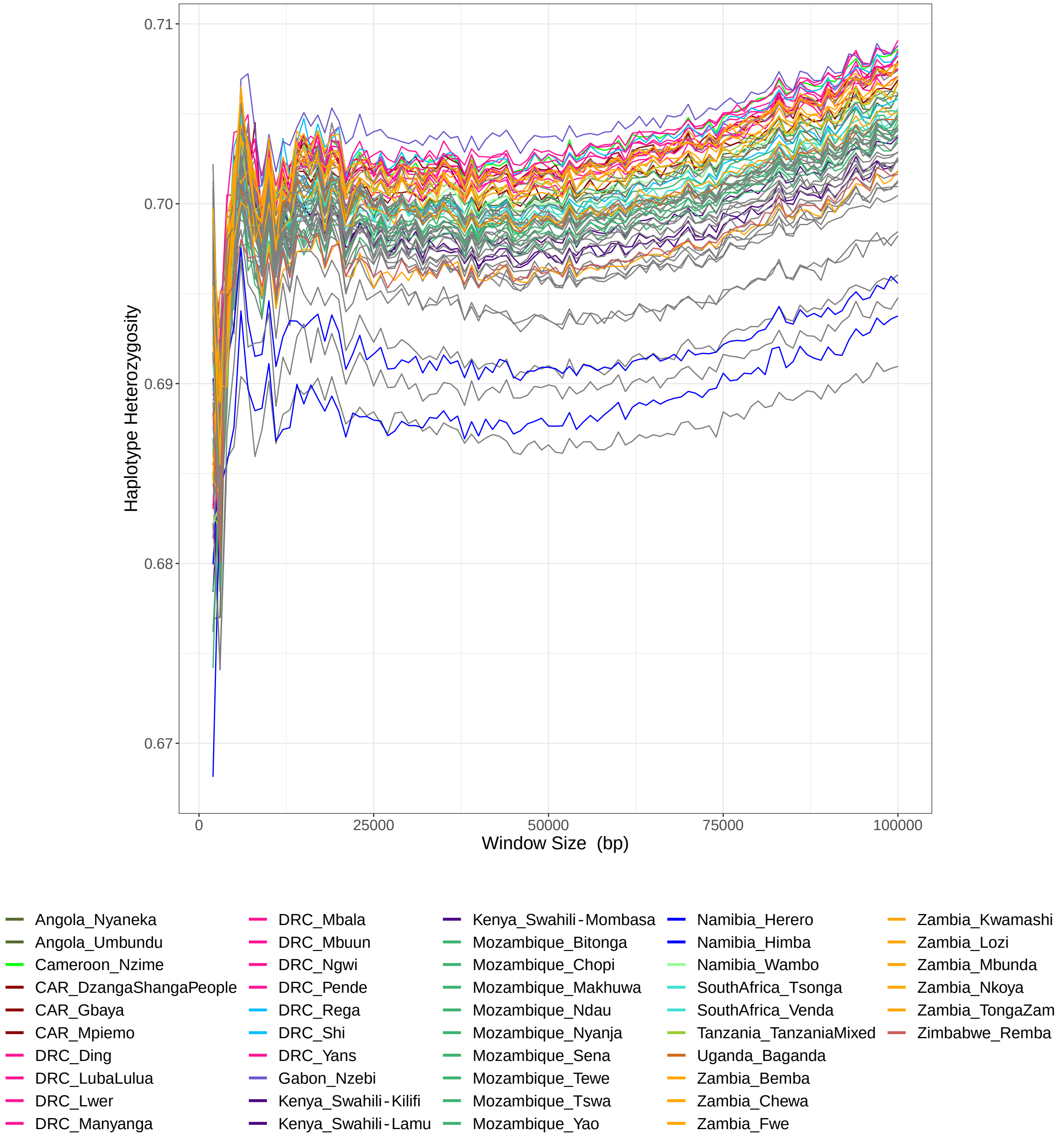

Supplementary Fig. 69 | Haplotype heterozygosity (HH) for the masked Only-BSP.
Figure showing estimated haplotype heterozygosity for populations included in the masked Only-BSP dataset, HH on the y-axis and the window size (S) on the x-axis.

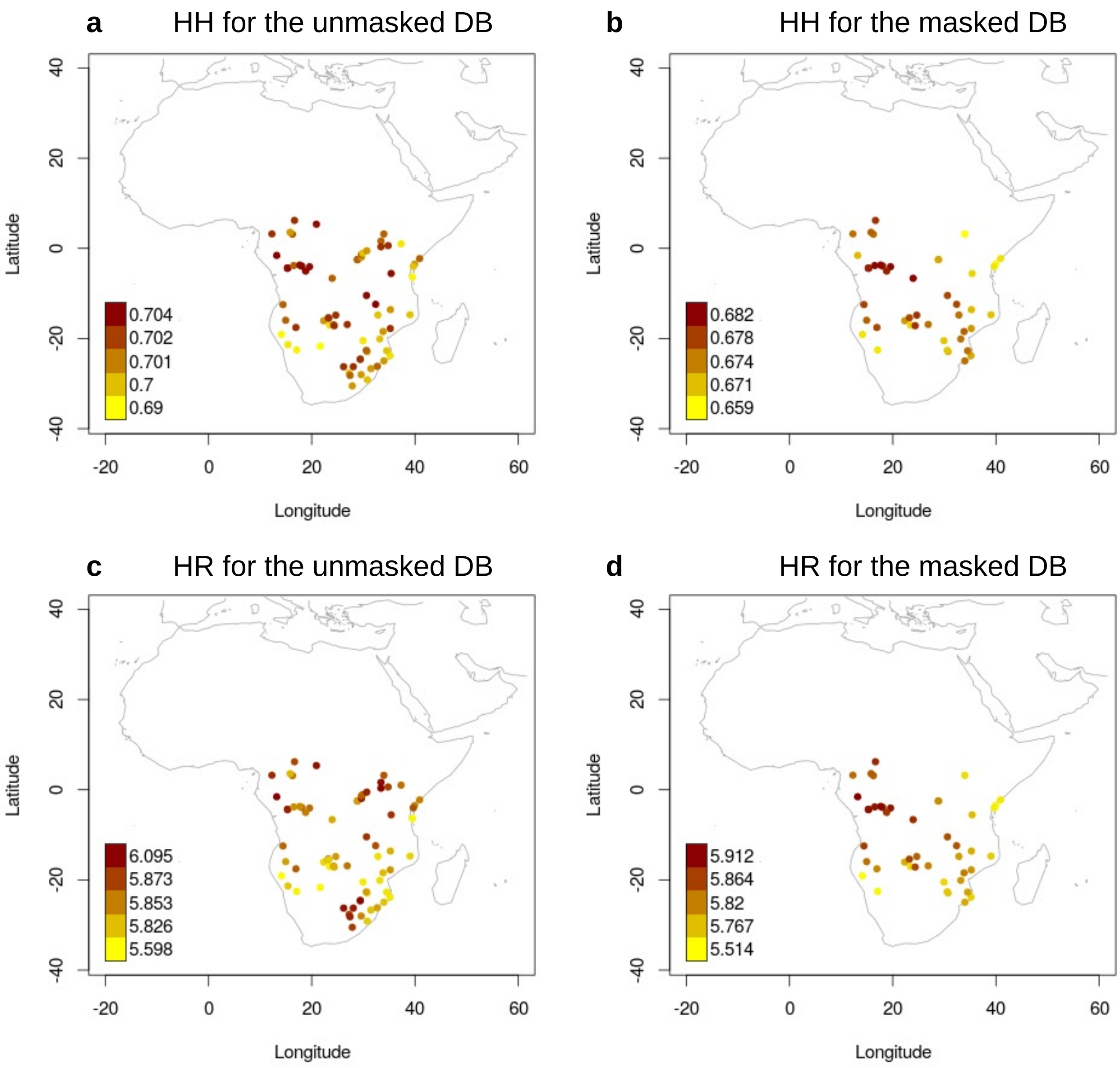

Supplementary Fig. 70 | Maps of haplotype diversity for the Only-BSP dataset.
Geographical distribution of haplotype heterozygosity and haplotype richness were estimated for the Only-BSP database (DB) using a window size (S) of 50 kb. Figure showing **a**, HH estimates for the unmasked Only-BSP database; **b**, HH estimates for the masked Only-BSP database; **c**, HR estimates for the unmasked Only-BSP database; and **d**, HR estimates for the masked Only-BSP database.

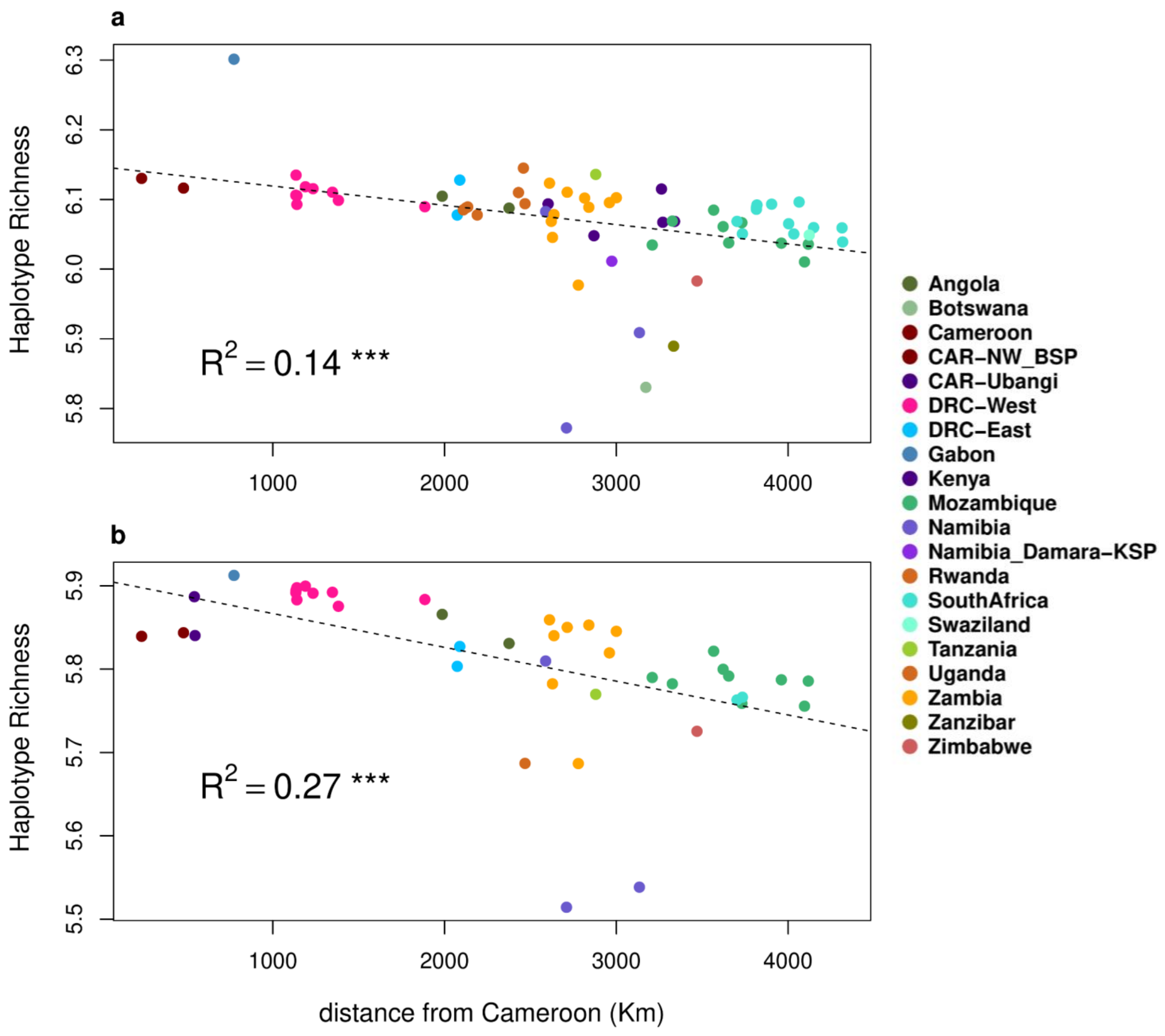

Supplementary Fig. 71 | Haplotype richness plotted against distance from Cameroon.
Figure showing haplotype richness (HR) values were plotted against the distance from Cameroon for the BSP in the dataset, calculated on the basis of **a**, the unmasked Only-BSP dataset and **b**, the masked Only-BSP dataset.

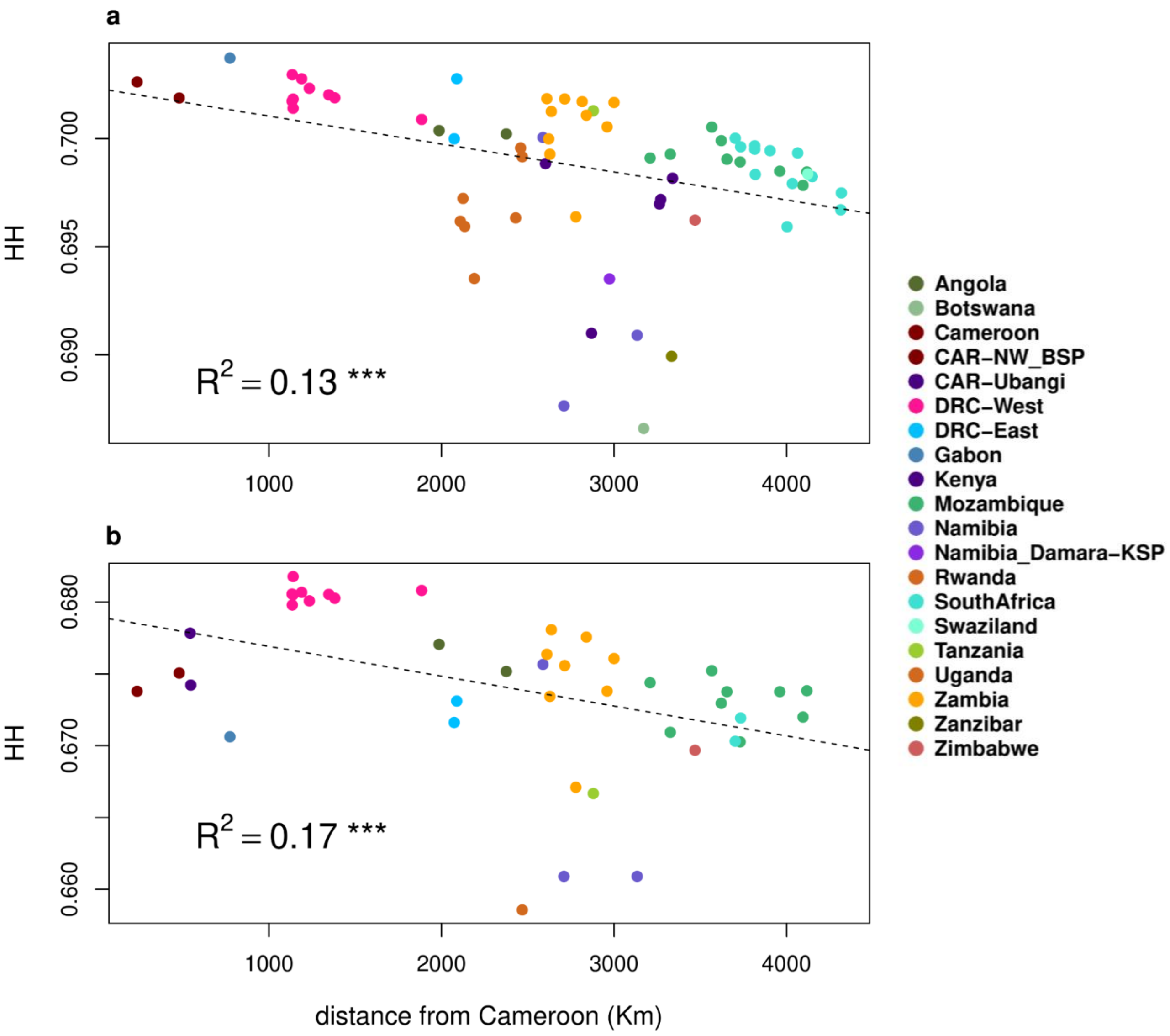

Supplementary Fig. 72 | Haplotype heterozygosity plotted against distance from Cameroon. Figure showing haplotype heterozygosity (HH) plotted against distance from Cameroon for the BSP in the dataset, calculated on the basis of **a**, the unmasked Only–BSP data and **b**, the masked Only–BSP dataset.

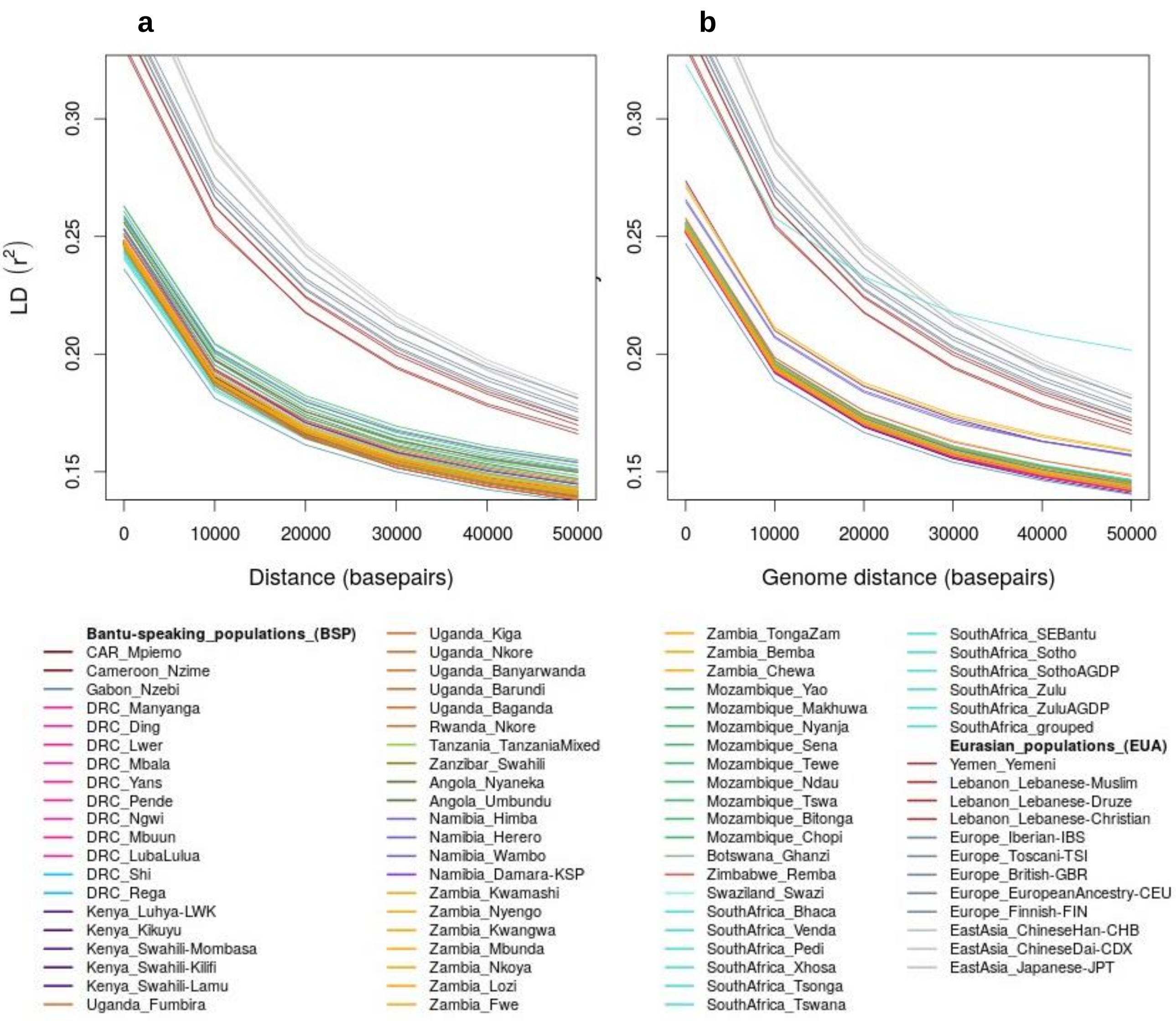

Supplementary Fig. 73 | Linkage-disequilibrium (LD)-decay of Only-BSP dataset.
LD-decay with genomic distance for BSP and Eurasian reference populations from the **a**, unmasked Only-BSP dataset (*n* = 67 populations); and **b**, the masked Only-BSP dataset (*n* = 51 populations) that includes BSP with at least 70% West-Central African (WCA) ancestry. For this plot, the minimum sample size was set to 7 individuals (e.g. Pedi population from South Africa), and South African populations with less than 3 individuals were grouped into one group called "SouthAfrica_grouped" (*n* = 11 populations). Results for the masked Only-BSP dataset show slightly higher LD-decay in comparison with the unmasked Only-BSP dataset.

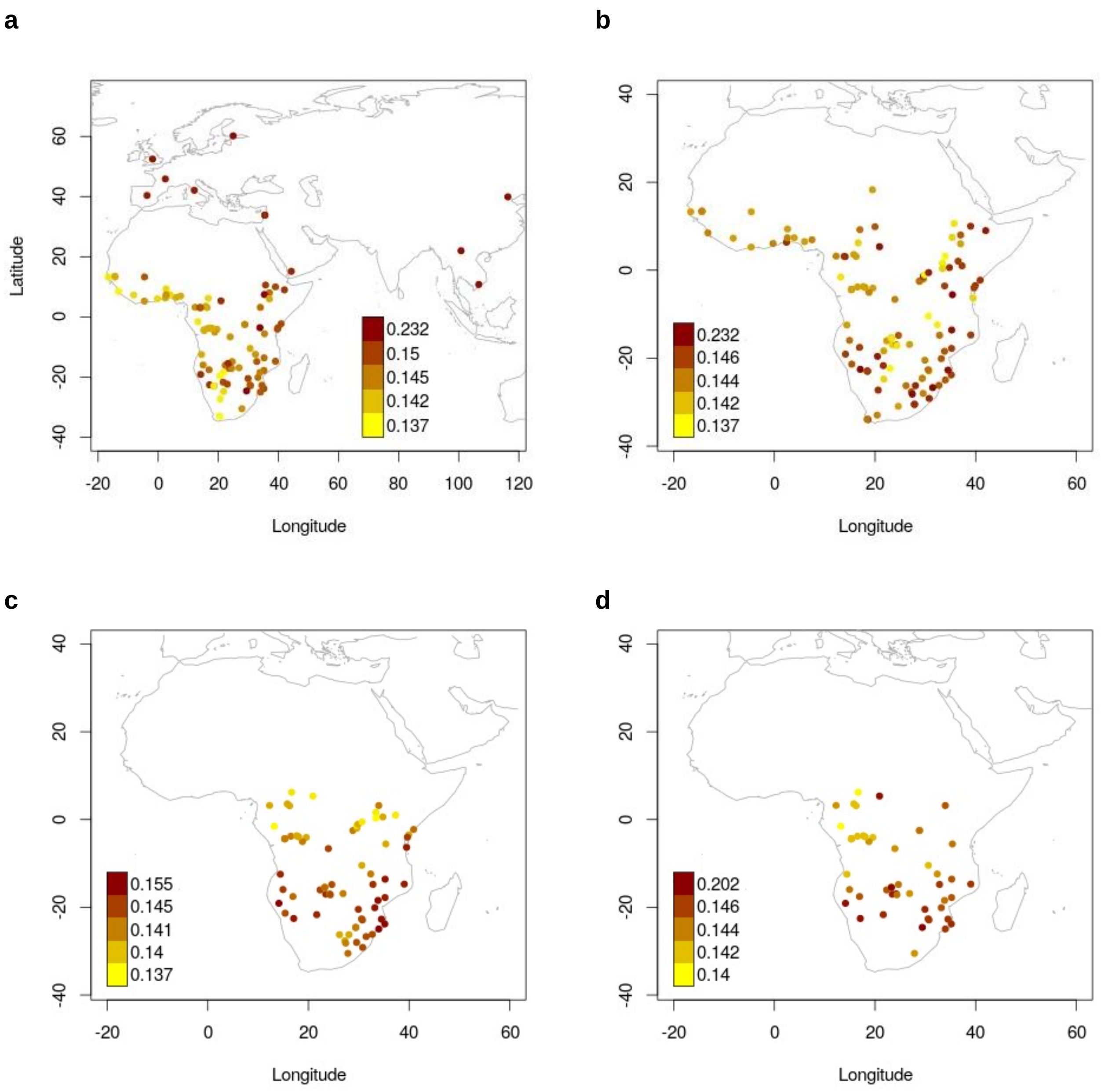

Supplementary Fig. 74 | Spatial distribution of LD-decay in each studied dataset.
Spatial distribution of linkage-disequilibrium (LD) estimates as r^2 at 50 Kb. Figure showing the results for **a**, BSP and worldwide reference populations included in the unmasked AfricanNeo dataset; **b**, Unmasked Only-African dataset; **c**, Unmasked Only-African dataset of selected African populations (*n* = 70 populations; with a minimum sample size of 10 individuals) (also Supplementary Fig. 4a). **d**, Masked Only-BSP dataset (*n* = 49 populations) that includes BSP with at least 70% of West-Central African-related ancestry (also Supplementary Fig. 4b). For this plot, the minimum sample size was 7 individuals (SouthAfrica_Pedi) and South African populations with less than 3 samples were grouped into one group called "SouthAfrica_grouped" (*n* = 11 individuals in total; and the position of this group was set as latitude= -30.51 and longitude= 27.84). Values of the colour scale are the quantiles 0.00, 0.25, 0.50, 0.75, and 1.0 of r^2 distribution in each set of populations.

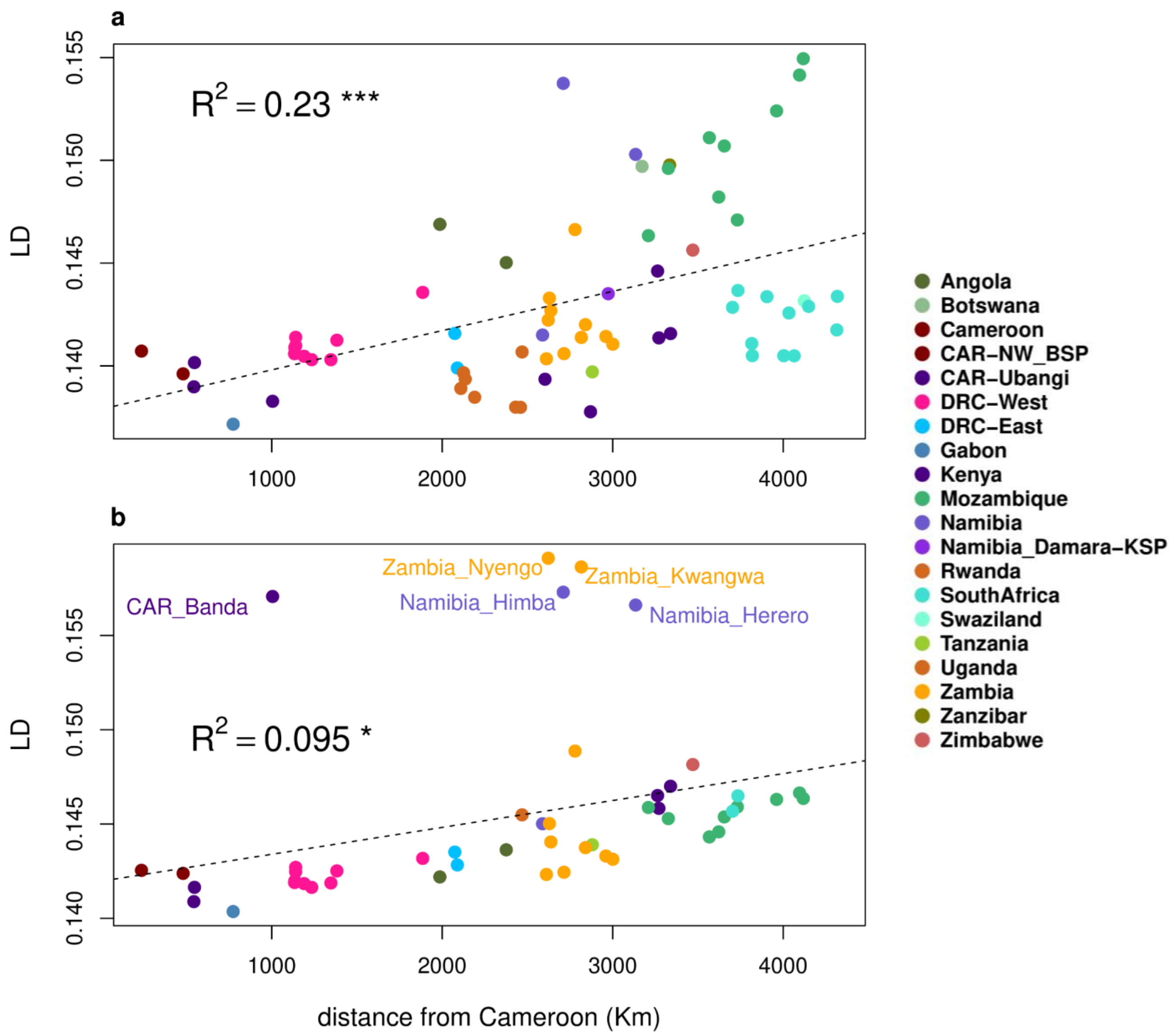

Supplementary Fig. 75 | Increase of LD patterns with geographical distances in studied BSP. Increase of LD estimates with geographical distance from Cameroon to the sampling location of each BSP and three Ubangi-speaking populations. **a,** Unmasked dataset of selected populations (70 populations in total). **b**, Masked dataset of selected populations (49 populations in total; SoutAfrica_Pedi with LD > 0.27 was excluded from this analysis). For each studied population, the LD value at 50kb was used in the correlation. Spatial distances were calculated as the spherical distance from each population and a centroid position located in the center of Cameroon. The dotted line represents the linear relationship between LD estimates and geographical distances.

3.10. Maximum likelihood trees based on population allele frequencies

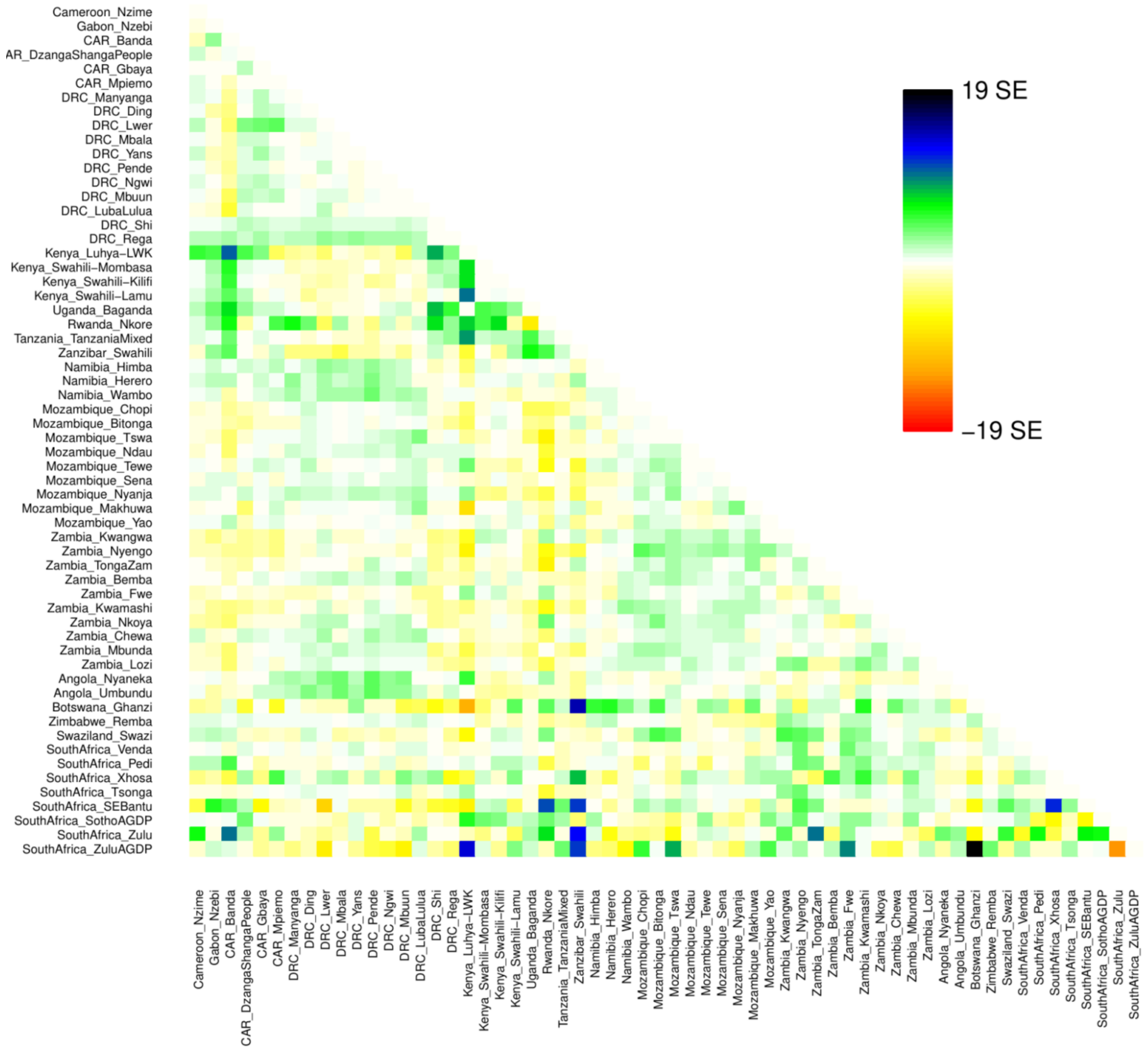

Supplementary Fig. 76 | Coancestry matrix for the masked and imputed Only-BSP dataset. Figure showing pairwise coancestry matrix summarizing genetic differences between pairwise populations included in the masked Only-BSP dataset obtained in TreeMix analysis (Supplementary Fig. 76) using default options for plotting.

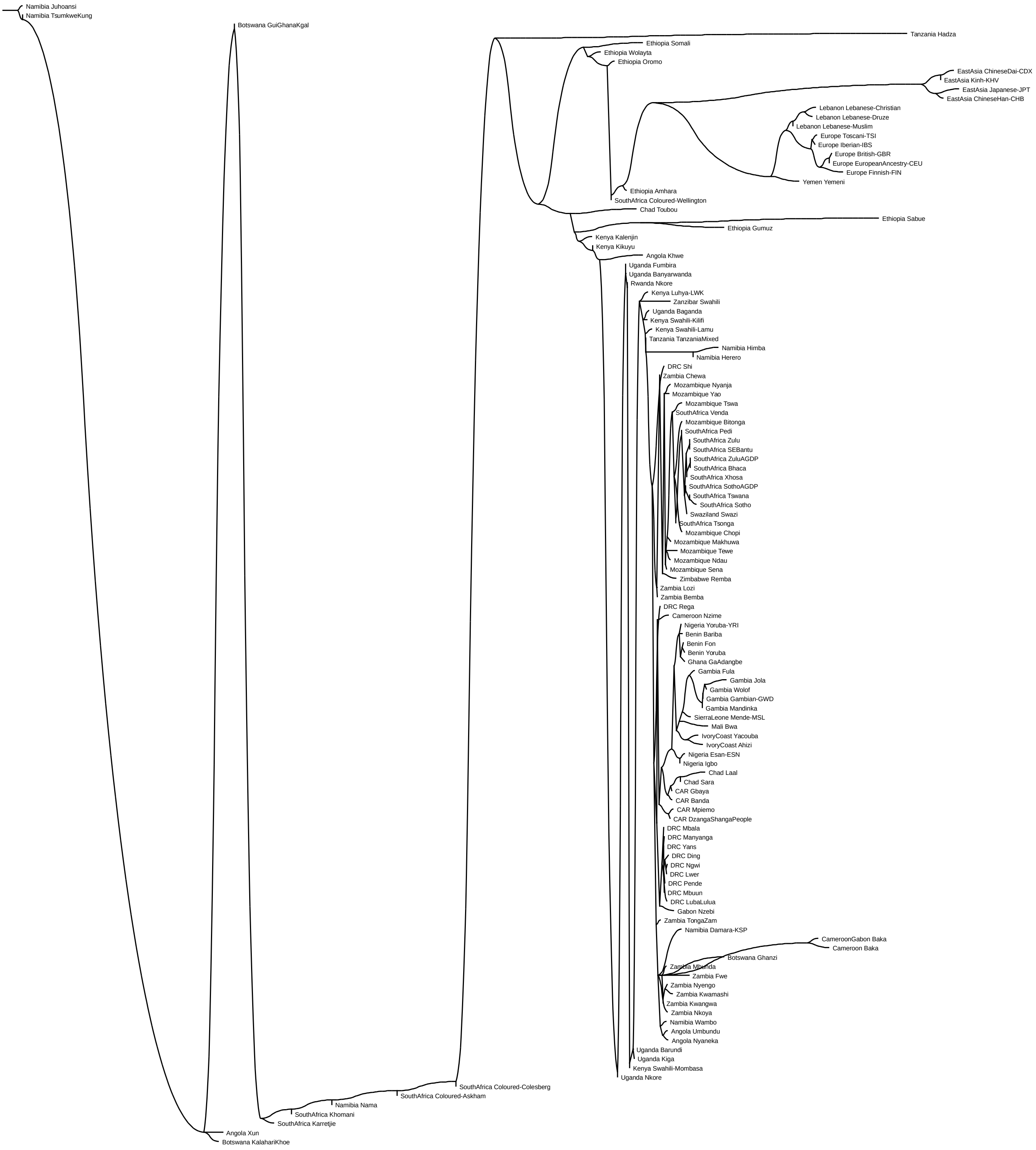

Supplementary Fig. 77 | TreeMix analysis for the unmasked AfricanNeo dataset.
Figure showing the tree was built from the covariance matrix of the population allele frequencies with TreeMix including all the populations of the unmasked AfricanNeo dataset.

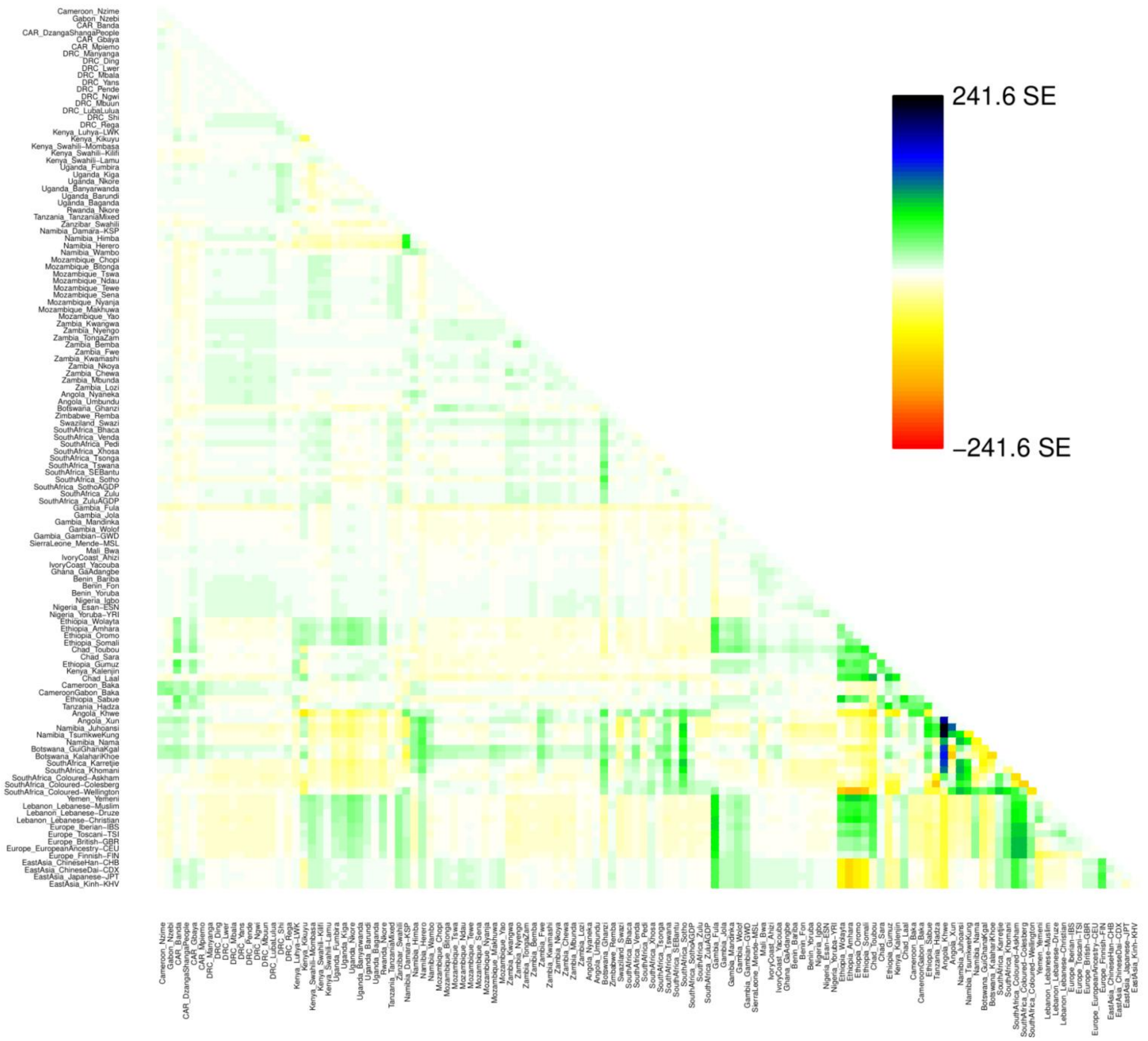

Supplementary Fig. 78 | Coancestry matrix for the unmasked AfricanNeo dataset.

Figure showing pairwise coancestry matrix summarizing genetic differences between pairwise populations included in the unmasked AfricanNeo dataset obtained in TreeMix analysis (Supplementary Fig. 77) using default options for plotting.

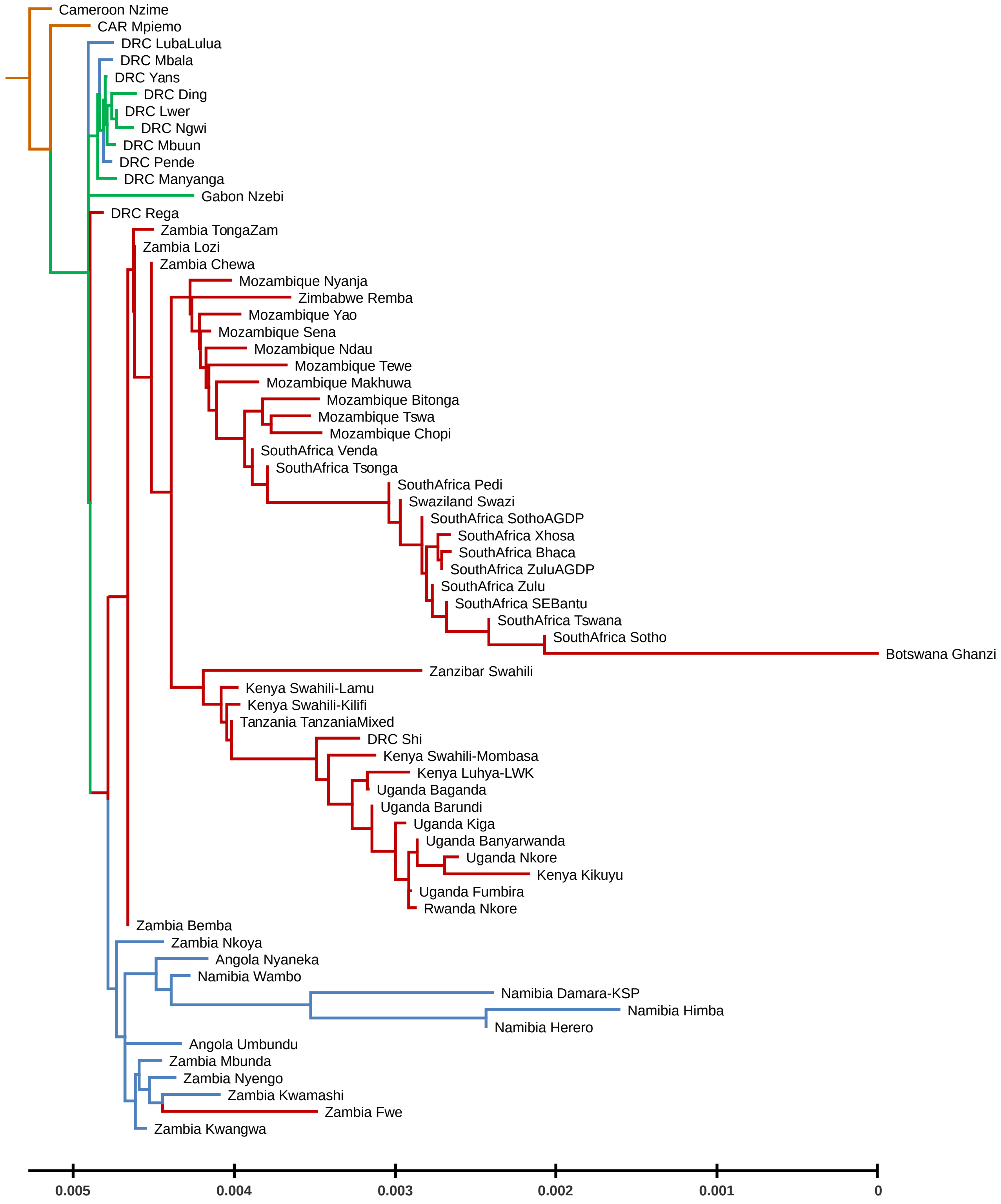

Supplementary Fig. 79 | TreeMix analysis for the unmasked Only-BSP dataset.
Figure showing population tree results for the unmasked Only-BSP dataset in a rectangular shape. The coancestry matrix of the inferred maximum likelihood (ML)-tree was included in Supplementary Fig. 80.

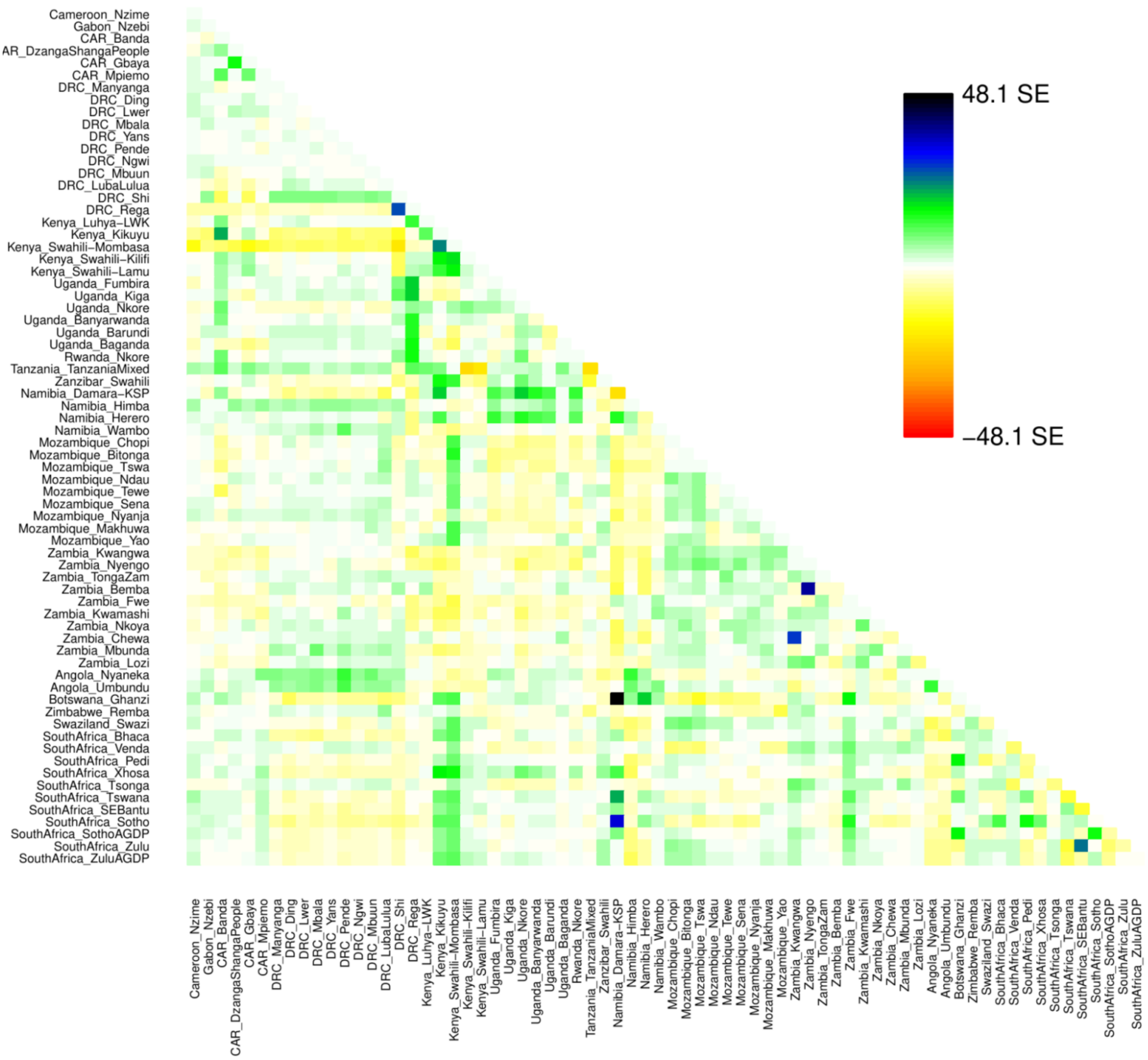

Supplementary Fig. 80 | Coancestry matrix for the unmasked Only-BSP dataset.

Figure showing pairwise coancestry matrix summarizing genetic differences between pairwise populations included in the unmasked Only-BSP dataset estimated using TreeMix analysis (Extended Data Fig. 7) using default options for plotting.

3.11. Visualizing migration routes in sub-Saharan Africa

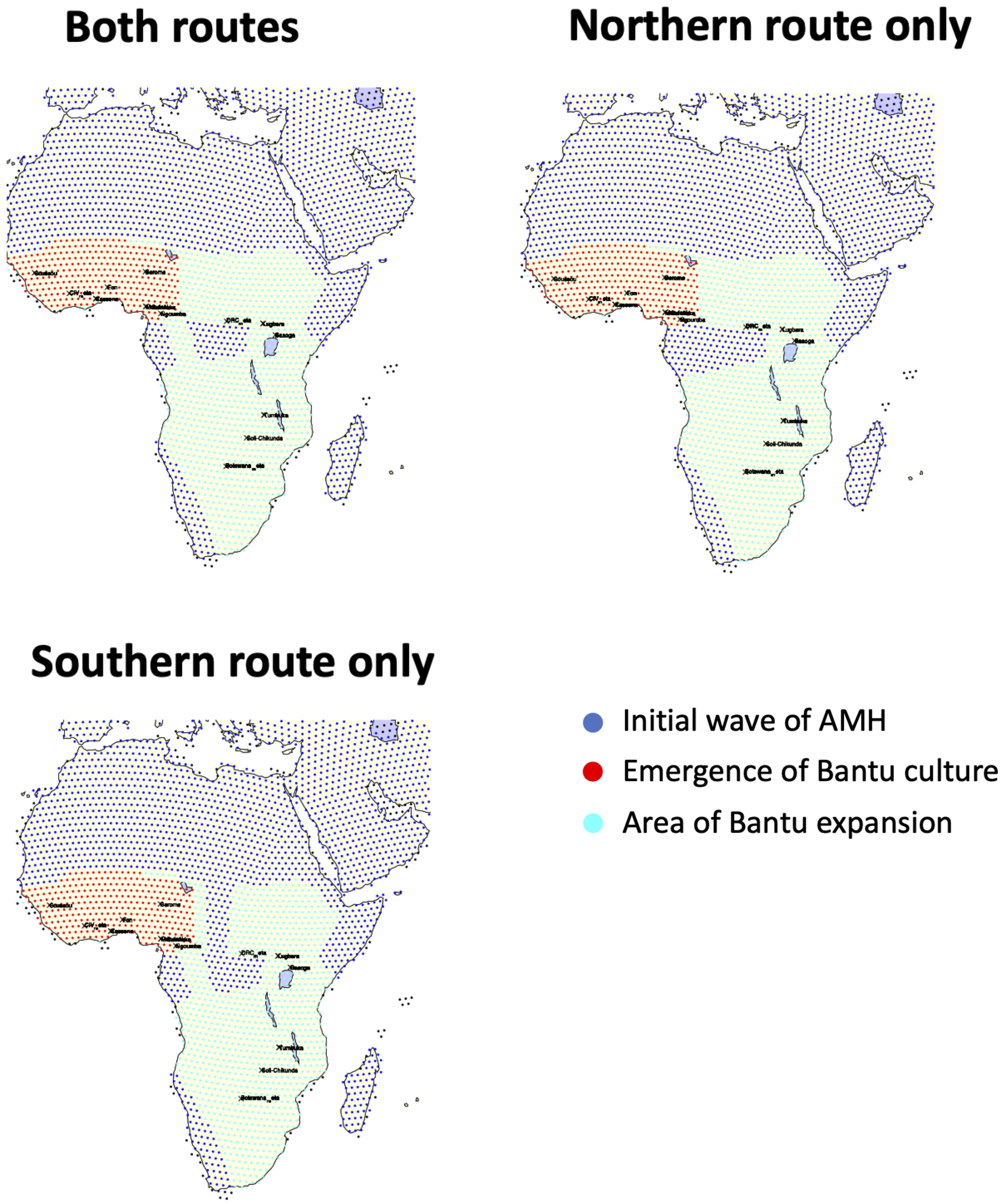

Supplementary Fig. 81 | Demographic scenarios used in our spatially explicit framework
Figure showing the three demographic scenarios considered in the spatially explicit framework. Each dot represents a local population, and the color indicates whether the deme is part of the initial wave of anatomically modern humans (AMH) alone (in blue), part of the area where Bantu culture emerged (in red) and subsequently spread (in cyan), shown against the continental contours and major water bodies for reference. Black dots and labels indicate the location and name of the populations with whole genome sequences included in the analysis.

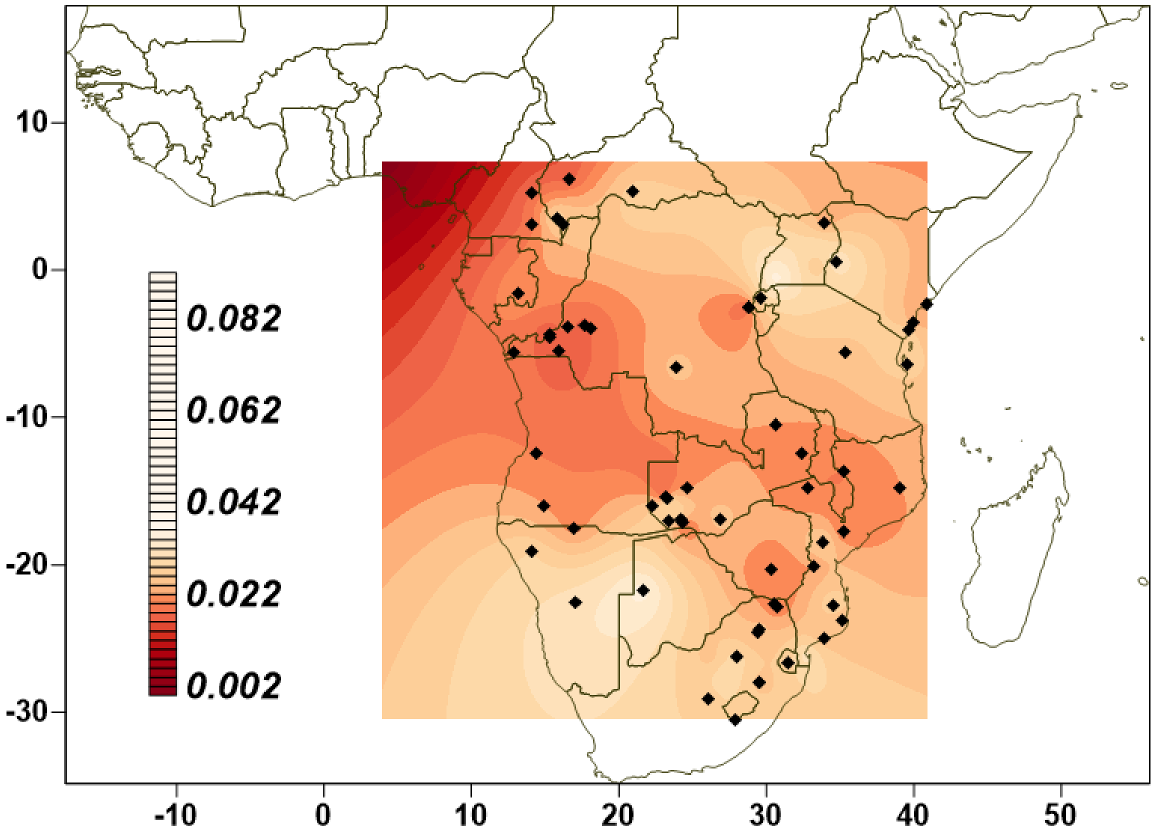

Supplementary Fig. 82 | F_{ST} matrix for the masked Only-BSP dataset.
Figure showing F_{ST} distances of one population in Cameroon and one BSP (black diamonds) from the masked and imputed Only-BSP dataset. Grid layer was created using Surfer Golden Software (© Golden Software, Inc 2023).

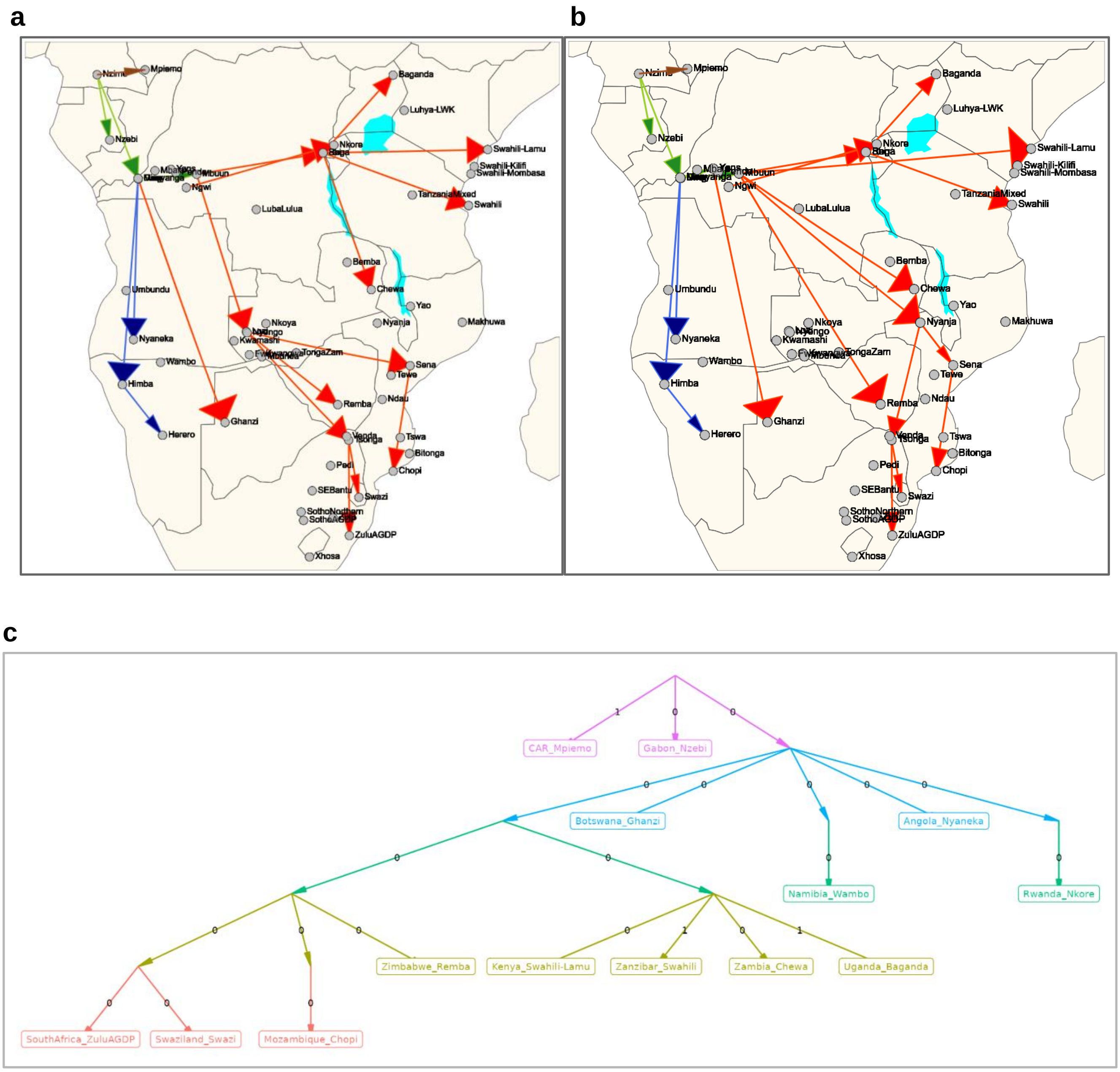

Supplementary Fig. 83 | F_{ST} map for the masked Only-BSP dataset.
F_{ST} values were estimated on the basis of the masked Only-BSP dataset. **a**, Figure showing F_{ST} map for all the BSP included in the AfricanNeo dataset, and **b**, for the BSP included in the AfricanNeo dataset except for the Lozi population from Zambia. Arrow colors correspond to studied four Bantu-speaking groups: north-western Bantu (or NW-BSP in brown), west-western Bantu (or WW-BSP in green), south-western Bantu (or SW-BSP in dark blue), and eastern Bantu speakers (or E-BSP in red). **c**, Admixture graph for the masked Only-BSP dataset. Figure showing admixture graph created using qpGraph for the masked Only-BSP dataset including populations analyzed in Supplementary Fig. 83a. Interactive plots are available at Github [113] (Suppl_Fig_83[a–b]_Fst_Map.html).

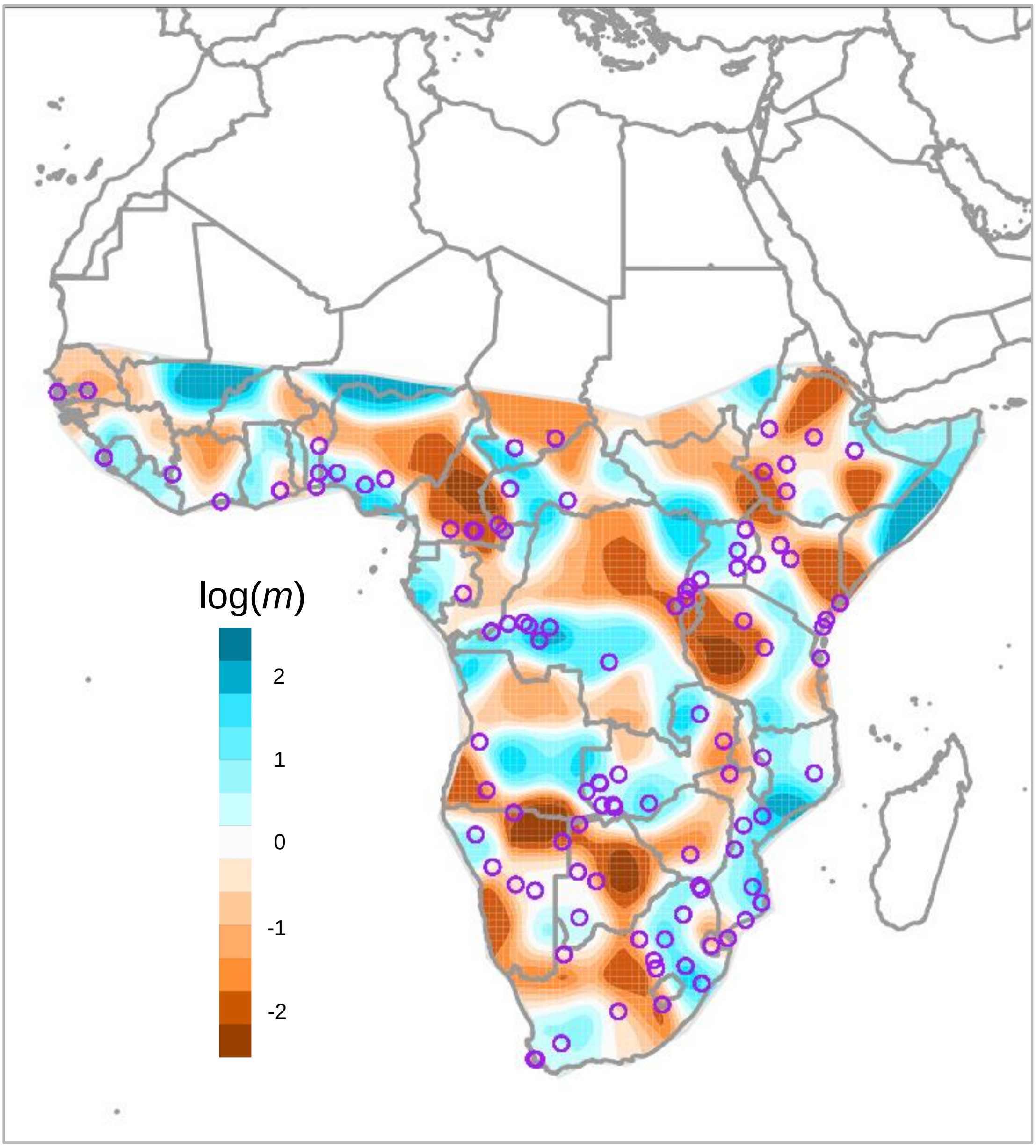

Supplementary Fig. 84 | EEMS for the unmasked Only-African dataset.
Figure showing EEMS results on the basis of unmasked Only-African dataset using 200 demes (also **Supplementary Fig. 2c**). Blue areas are regions with high effective migration rates within the dataset, whilst brown areas indicate regions of inferred low effective migration rates (i.e. patterns of historic barriers to human migration). Purple circles indicate the sampling locations of each studied population.

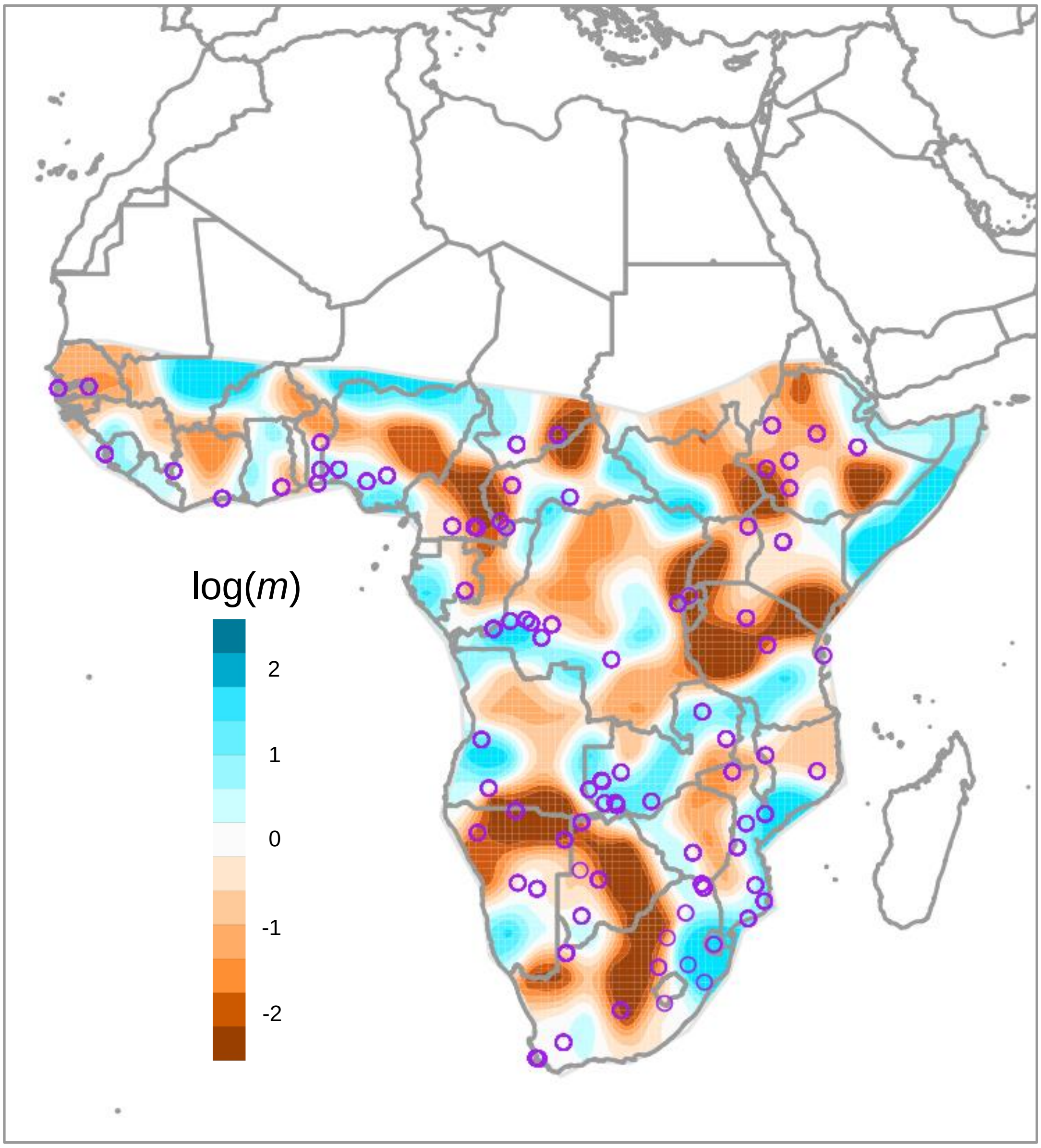

Supplementary Fig. 85 | EEMS for the masked Only-African dataset.
EEMS for the Only-African dataset after masking data of BSP. Blue areas indicate regions with high effective migration rates within the dataset whilst brown areas indicate regions of inferred low effective migration rates (i.e. patterns of historic barriers to human migration). Purple circles indicate the sampling locations of each studied population.

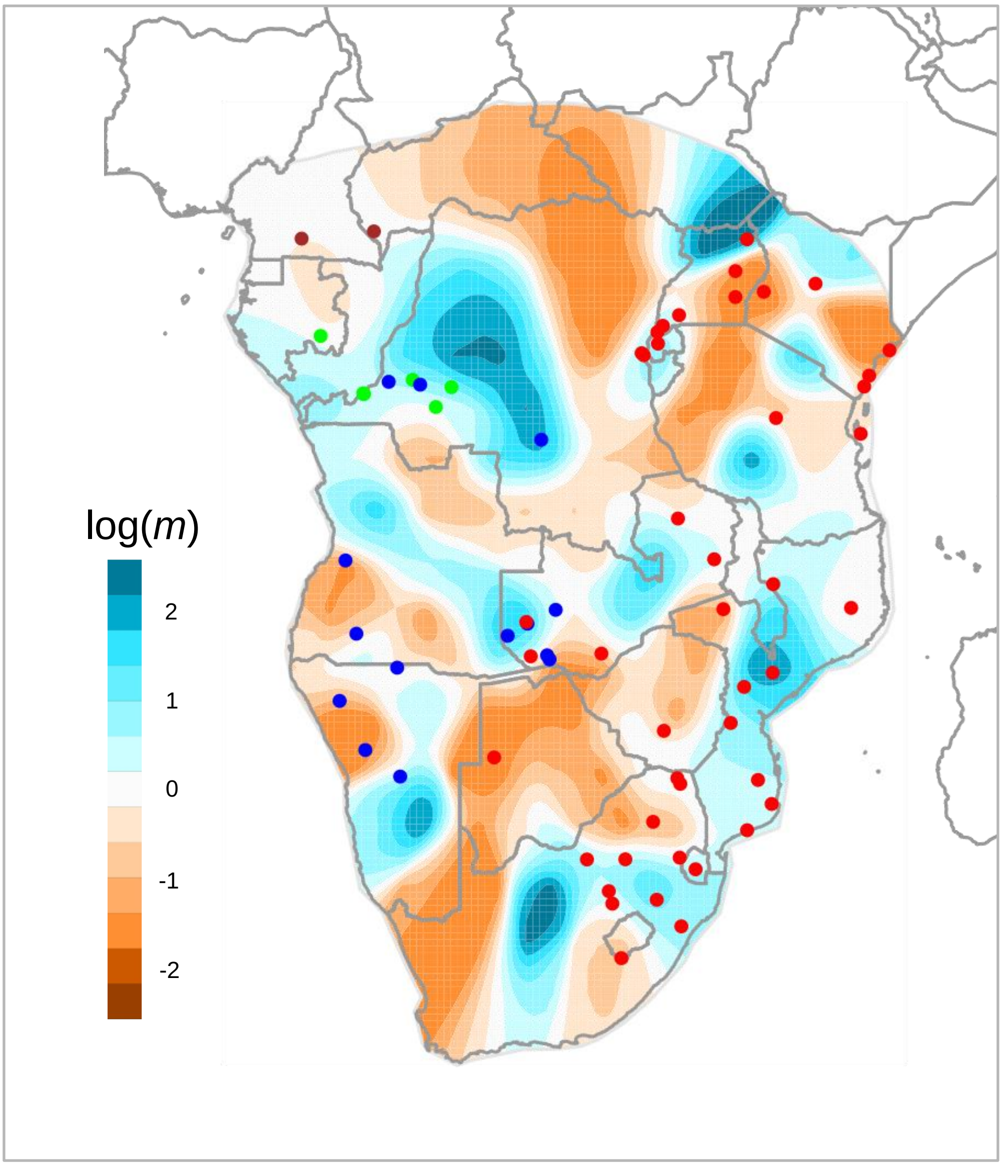

Supplementary Fig. 86 | EEMS for the unmasked Only-BSP dataset.
Figure showing EEMS results on the basis of unmasked Only-BSP dataset using 200 demes (also **Supplementary Fig. 5c**). Purple circles indicate the sampling locations of each studied population. Blue areas are regions with high effective migration rates within the dataset whilst brown areas indicate regions of inferred low effective migration rates (i.e. patterns of historic barriers to human migration). Each dot was coloured according to the following classification: north-western Bantu 2 (in brown), west-western Bantu (in green), south-western Bantu (in dark blue), and eastern Bantu speakers (in red).

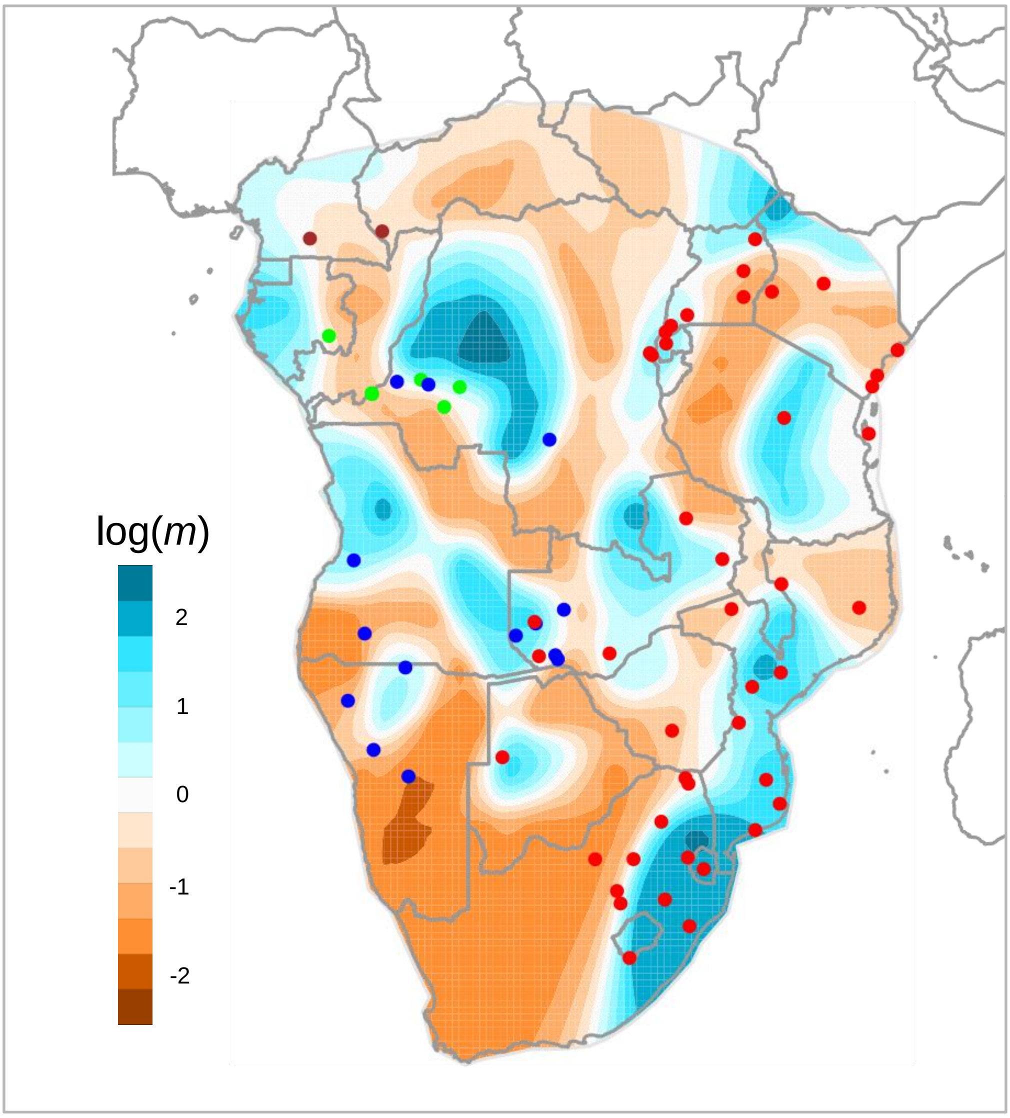

Supplementary Fig. 87 | EEMS on the basis of the masked Only-BSP dataset.
Figure showing EEMS results on the basis of only masked and imputed data of BSP. Purple circles indicate the sampling locations of each studied population. Blue areas are regions with high effective migration rates within the dataset whilst brown areas indicate regions of inferred low effective migration rates (i.e. patterns of historic barriers to migration).

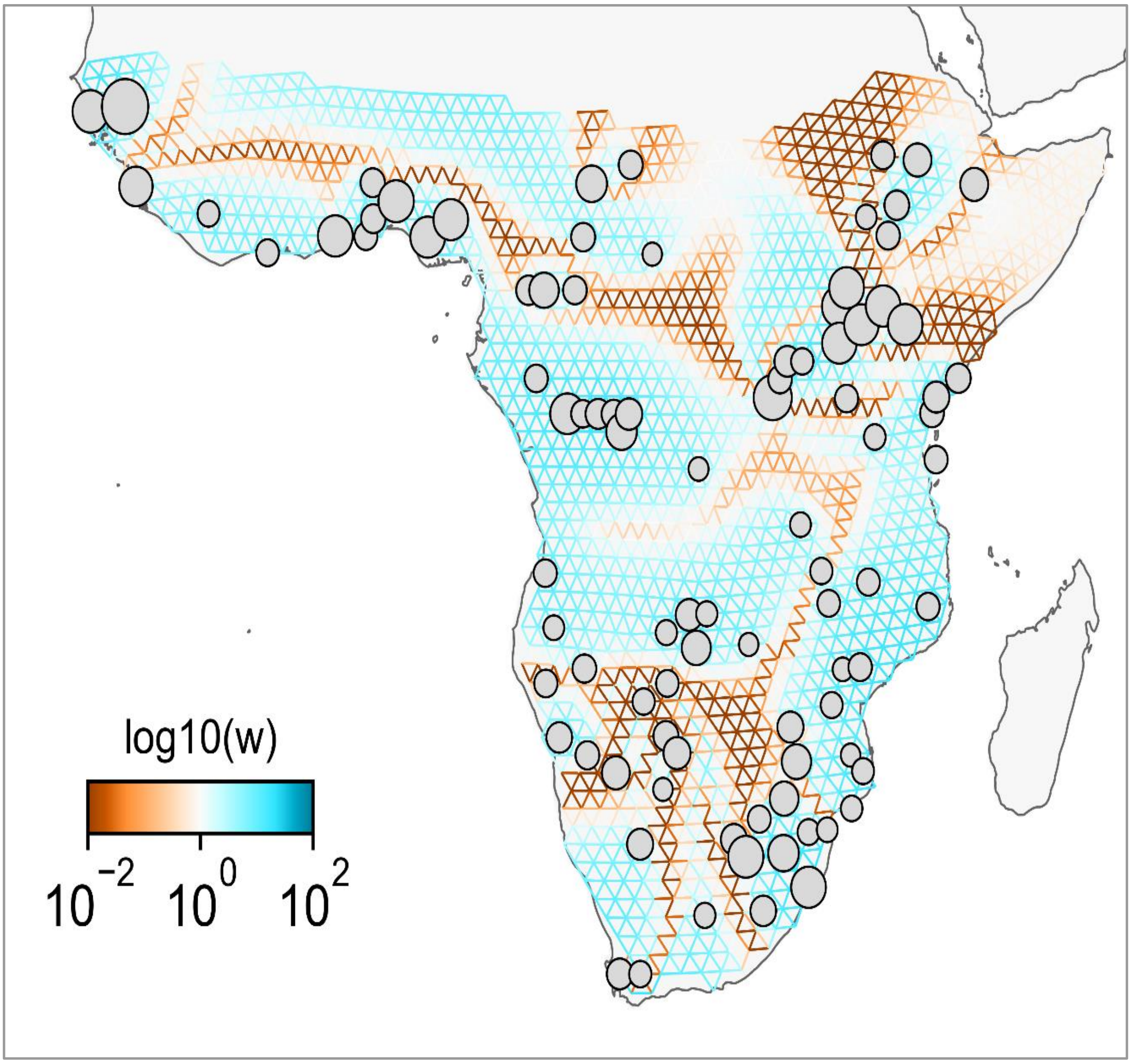

Supplementary Fig. 88 | FEEMS of the unmasked AfricanNeo dataset.
Figure showing FEEMS results on the basis of the full list of sub-Saharan African populations included in the AfricanNeo dataset. Each studied population is represented with one dot, and the size of the dots is in relation to the sample size of each population.

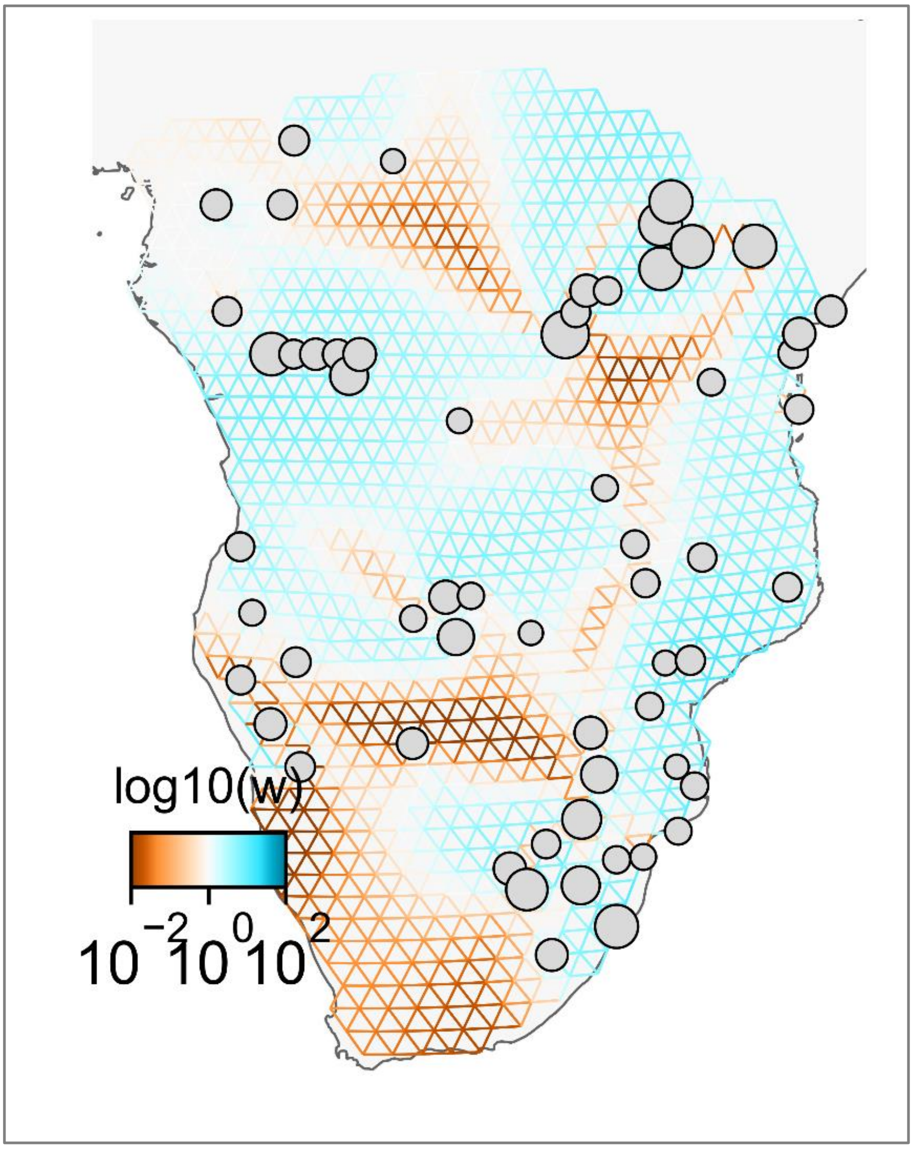

Supplementary Fig. 89 | FEEMS on the basis of the unmasked Only-BSP dataset.

Figure showing FEEMS results on the basis of the unmasked Only-BSP dataset. Each studied population is represented with one dot, and the size of the dots is in relation to the sample size of each population.

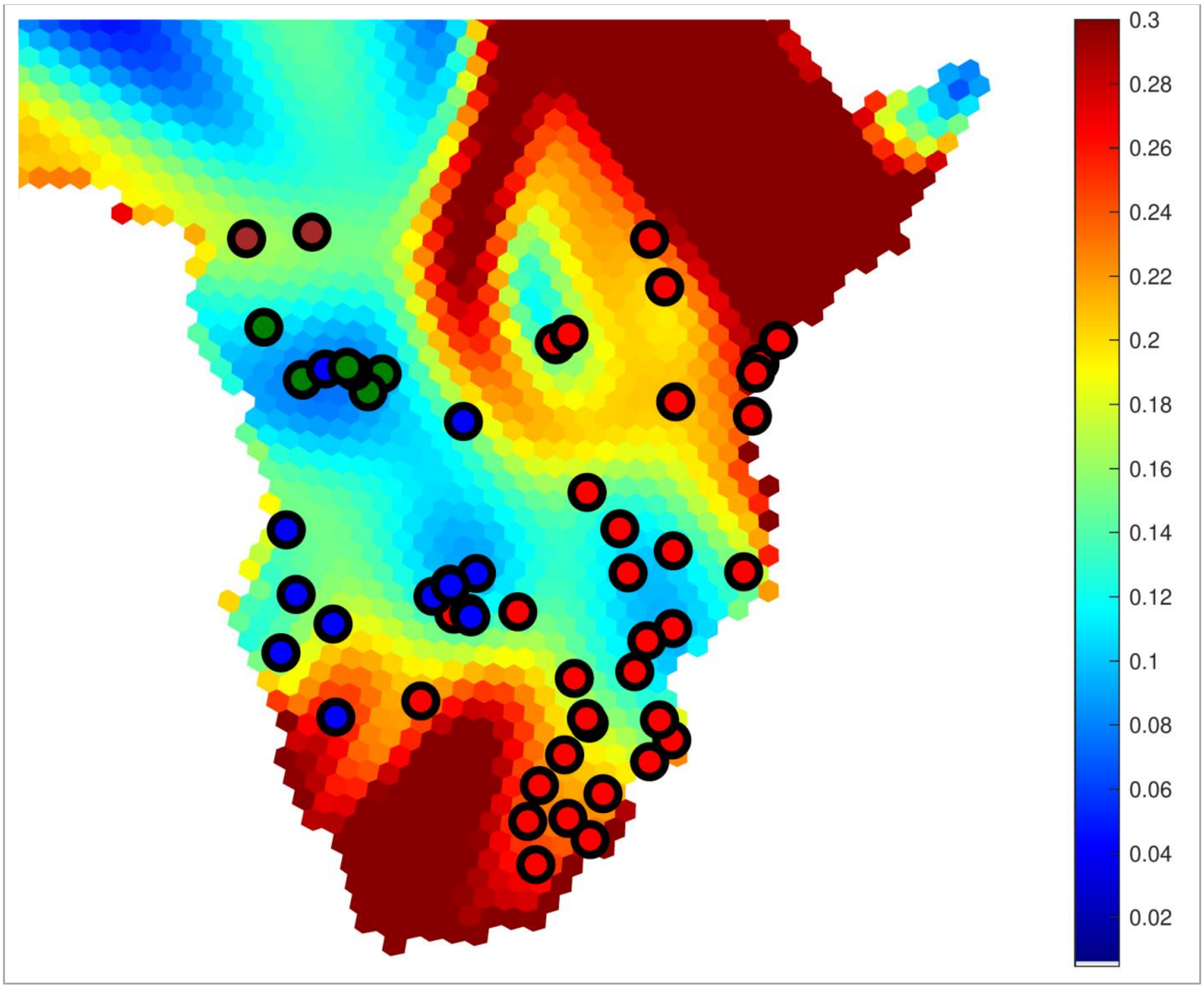

Supplementary Fig. 90 | Spatial visualization of genetic barriers analysis on a grid.
Figure showing barriers to migration using F_{ST} as the distance for the masked and imputed BSP dataset. High values (in red) indicate sharp changes in alleles between populations, and thus are indicative of barriers to gene flow whilst low values (in blue) indicate the opposite. Hexagons of the grid were plotted with a color scale representing the F_{ST} gradient.

a b

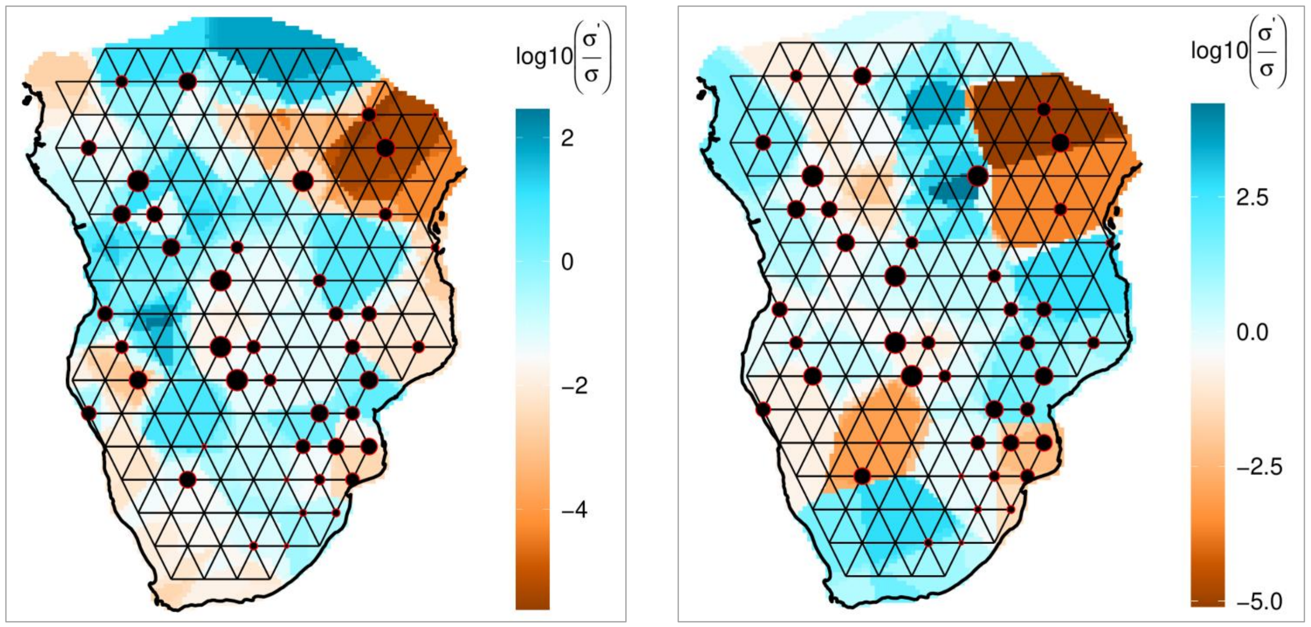

Supplementary Fig. 91 | Dispersal surfaces based on shared IBD tracts.
Figure showing MAPS results on the basis of IBD segments estimated from the only masked and imputed Only-BSP dataset. We examined IBD length categories of **a**, 2–4cM for older generations and **b**, >6 cM for recent generations. MAPS approach transforms the symmetric migration rate (m) estimated using EEMS into dispersal distance (σ) by scaling with the grid step-size (Al-Asadi et al. 2019). Like in EEMS and FEEMS plots, light blue areas show higher migration and brown areas show lower migration. The size of each circle is proportional to the number of genetic samples.

3.12. Estimated admixture dates in BSP

a

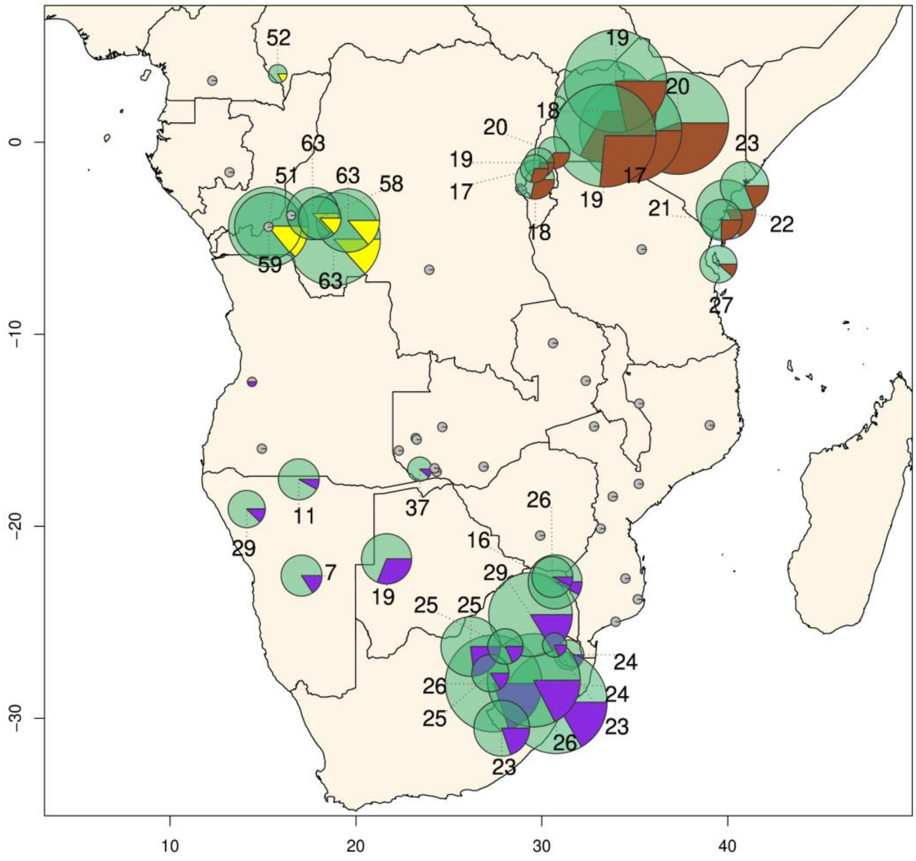

b

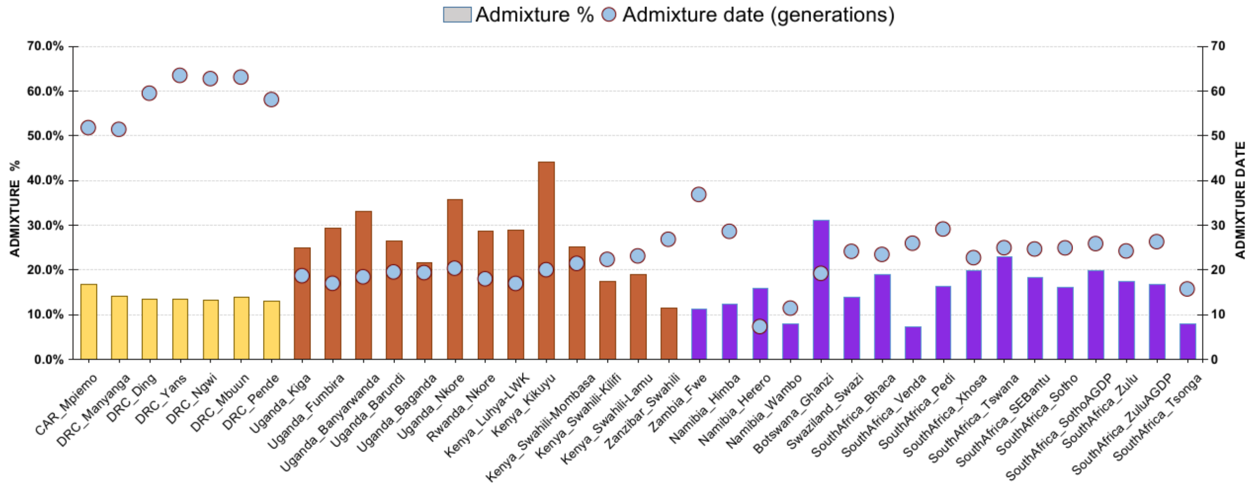

Supplementary Fig. 92 | MOSAIC results for BSP with admixture.

Figure showing MOSAIC results for all studied BSP computed using a two-way admixture model (also **Supplementary Fig. 2b**). Figure showing **a,** pie charts and locations of BSP with inferred admixture and **b,** bar plots for the admixture proportions and dates (gray circles). Each ancestry in (a) has different colors: west-central African-related ancestry in green; western rainforest hunter-gatherer ancestry in yellow; Afro-Asiatic-speaking ancestry in brown; and Khoe-San-speaking ancestry in purple. The size of the charts is in relation to the sample size of each BSP. The colors in (b) indicate western rainforest hunter-gatherer ancestry in yellow; Afro-Asiatic-speaking ancestry in brown; and Khoe-San ancestry in purple.

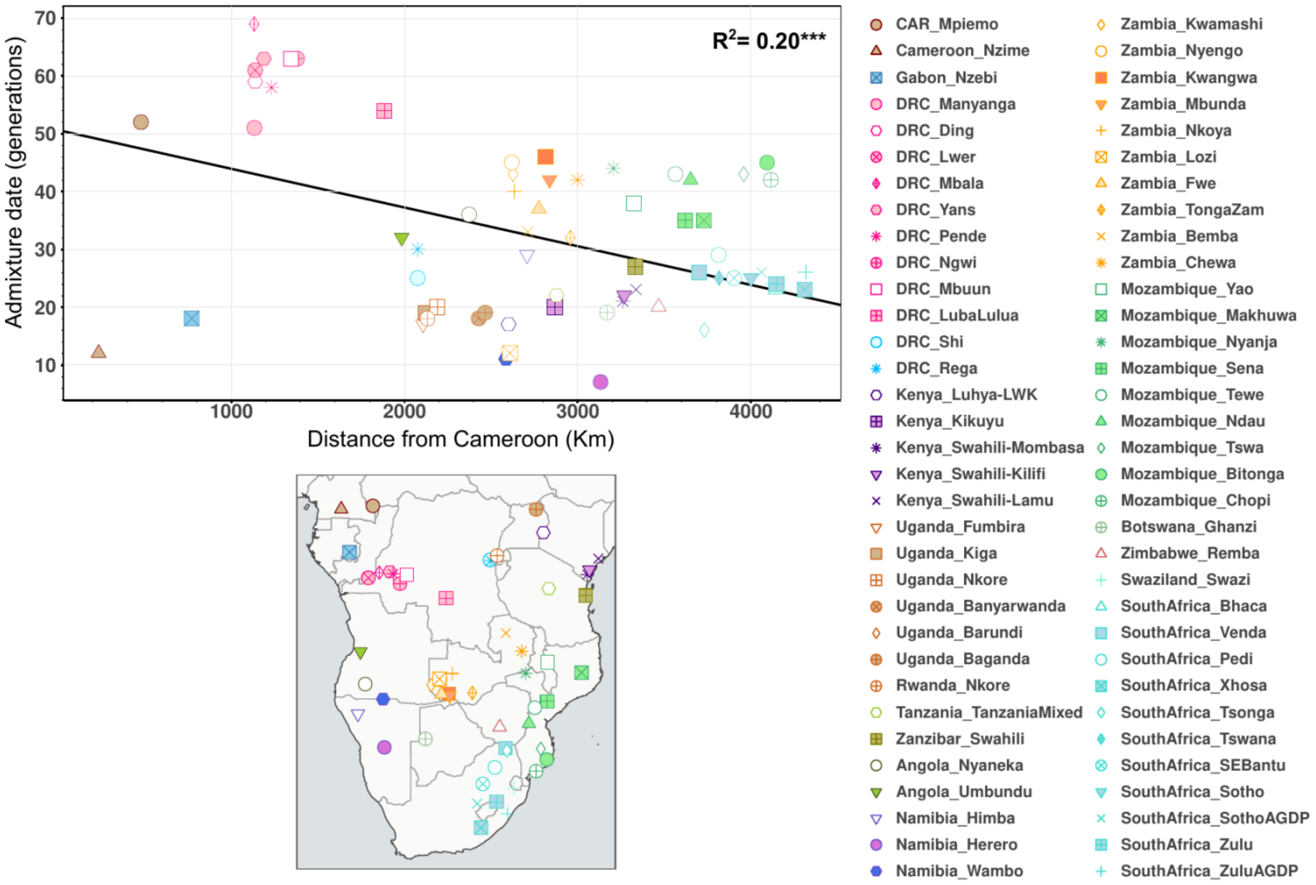

Supplementary Fig. 93 | Admixture dates versus geographical distances from Cameroon. Figure showing admixture dates estimated using MOSAIC analyses for BSP from Supplementary Fig. 11.1 plotted against geographical distances from Cameroon (also Supplementary Fig. 2C). The markers have different shapes for each Bantu-speaking group: north-western (a circle), western (a diamond), and eastern (triangle) group. The "western" subgroup represented by diamonds comprises west-western and south-western BSP. Vector basemap and map tiles were provided by CartoDB (© CARTO 2023).

3.13. Comparisons between aDNA individuals and modern-day African populations

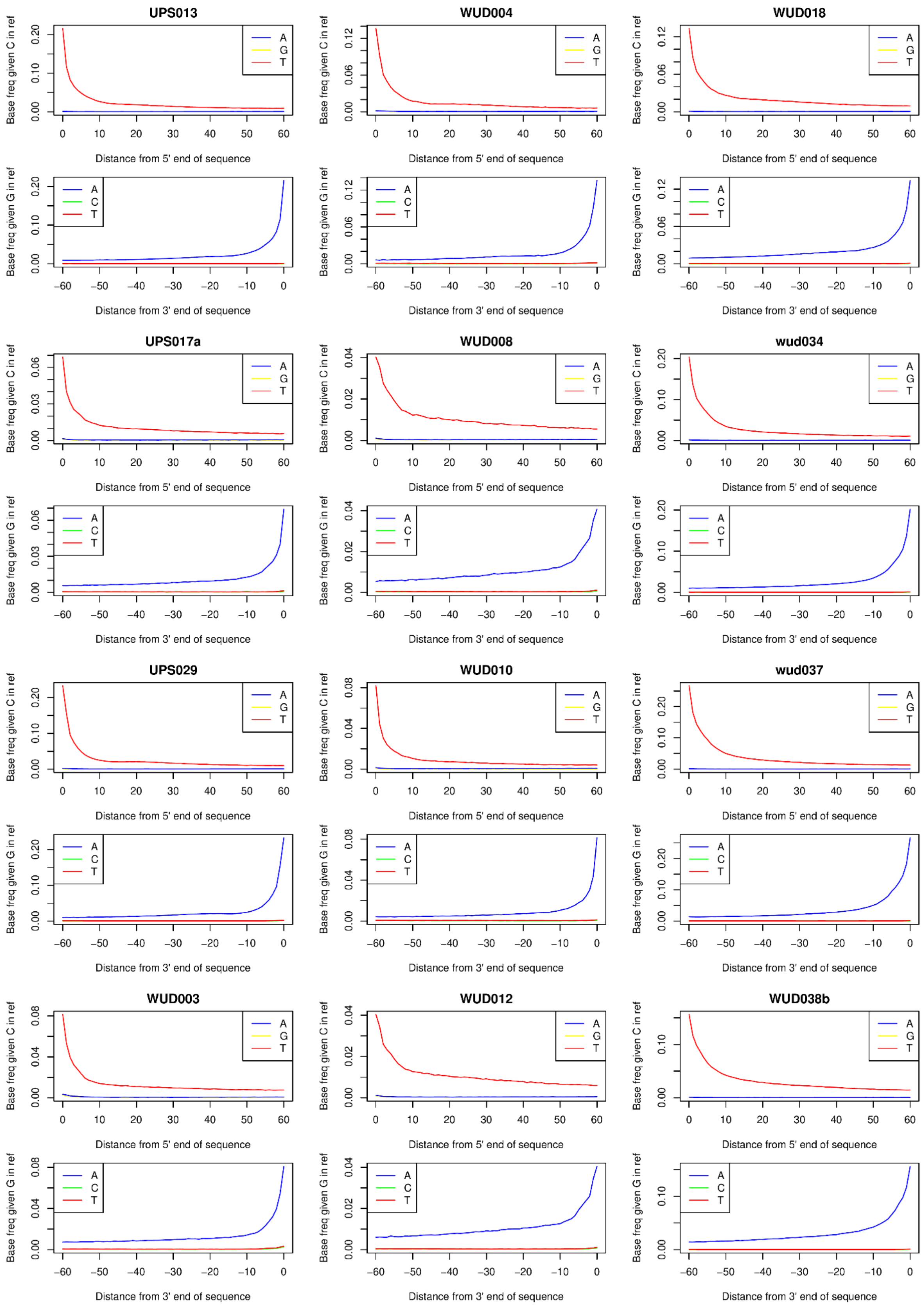

Supplementary Fig. 94 | Nucleotide misincorporation patterns of 12 aDNA individuals.
The observed patterns of the 12 newly sequenced ancient individuals (**Supplementary Table 14**) increasing in deamination at the 5’ and 3’ fragment ends are typical for ancient DNA.

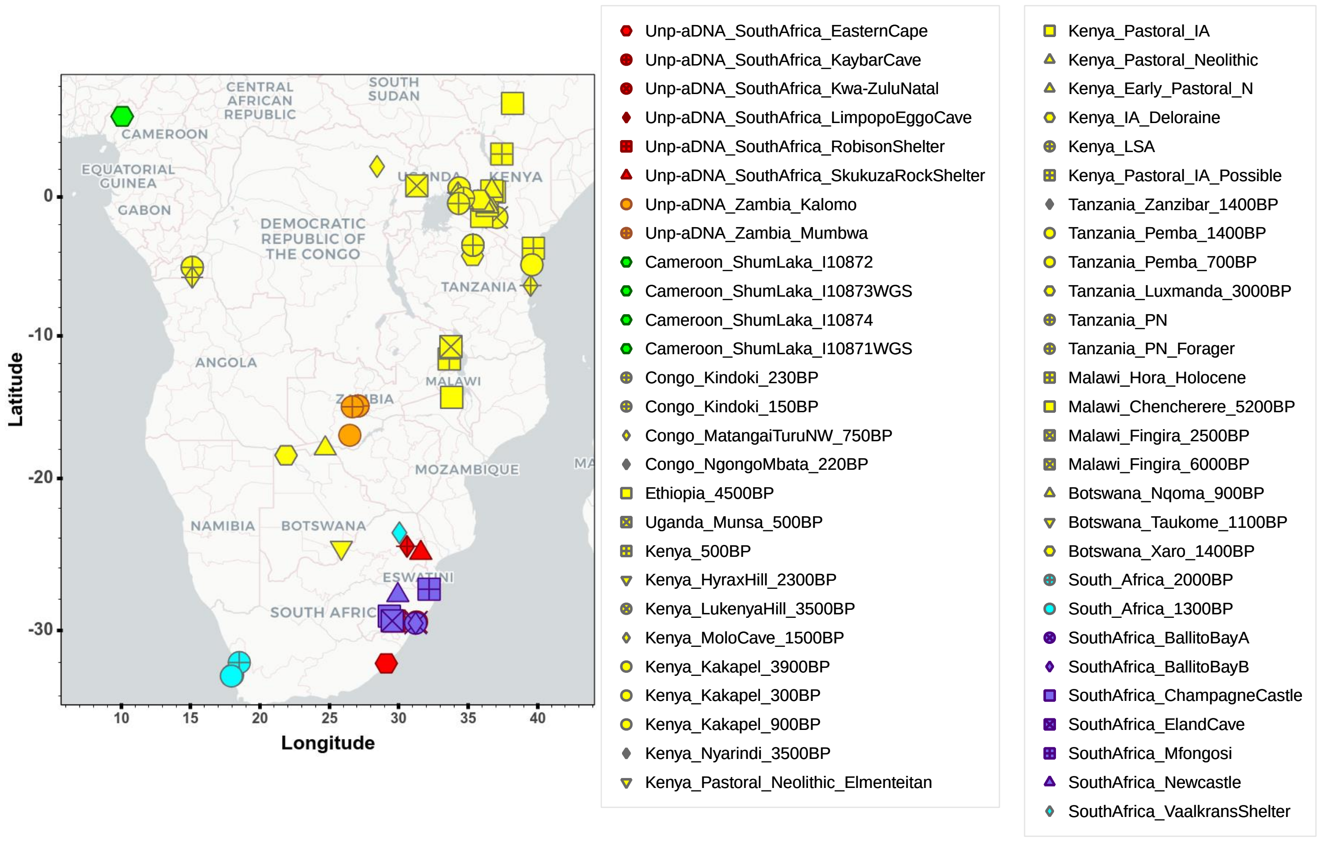

Supplementary Fig. 95 | Geographical locations of aDNA individuals included in this study. Figure showing the locations of the 12 aDNA individuals analyzed for the first time in this study (Supplementary Table 14) and 83 aDNA individuals from previous aDNA studies [23,57–62] (Supplementary Table 13; 95 aDNA individuals in total). To better visualize the figure, we highlighted new aDNA individuals from Zambia in orange circles, new aDNA individuals from South Africa in red, aDNA individuals from previous studies are indicated by other colors and symbols (yellow, blue, green, and purple markers). To better visualize the locations, dates, and ID of each aDNA individual, interactive plots are available at Github [113] (Suppl_Fig_95_aDNA_Map.html). Vector basemap and map tiles were provided by CartoDB (© CARTO 2023).

a

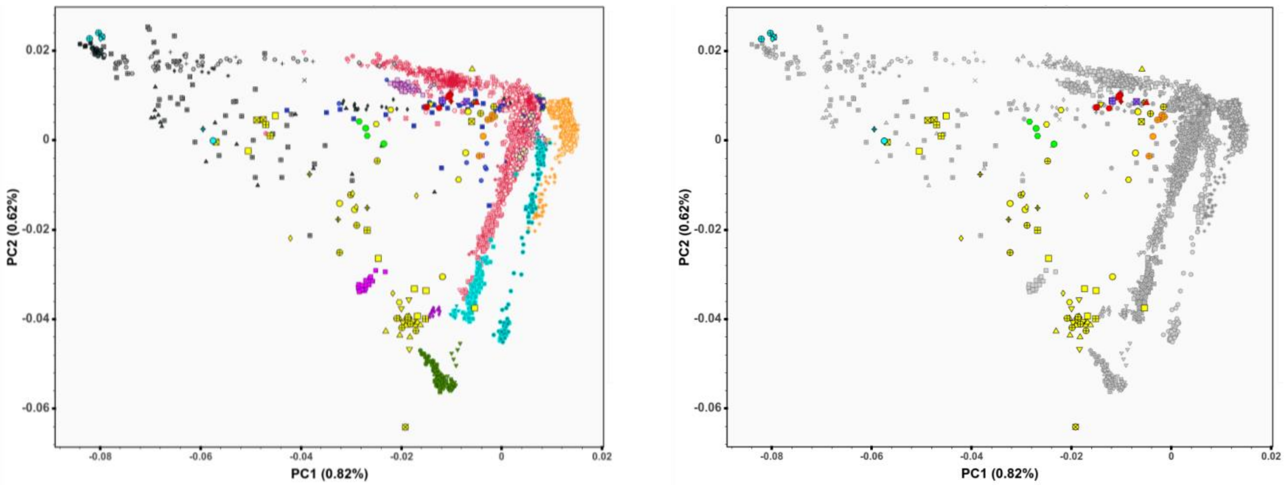

b

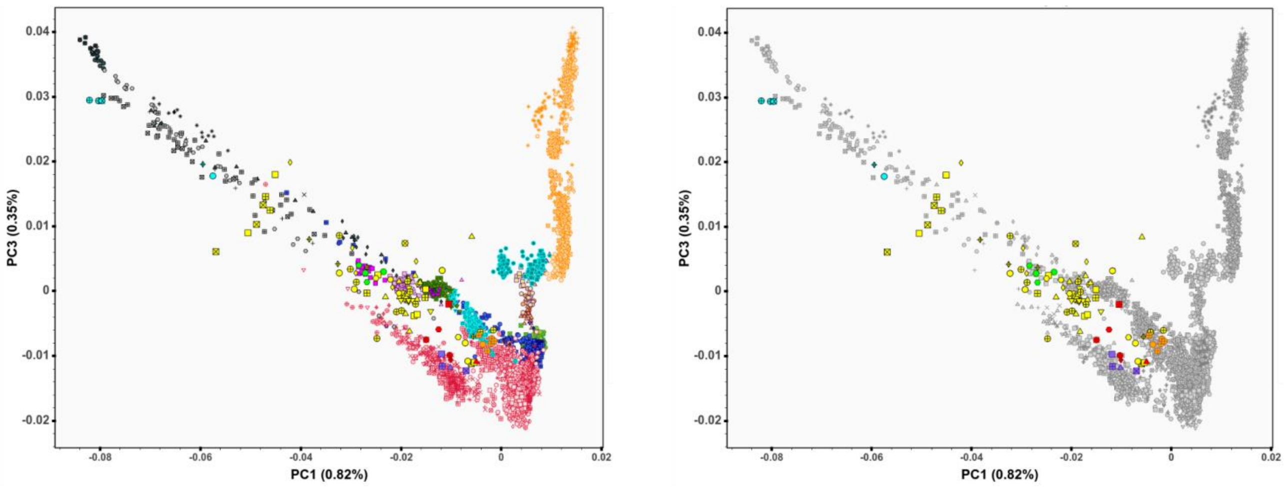

c

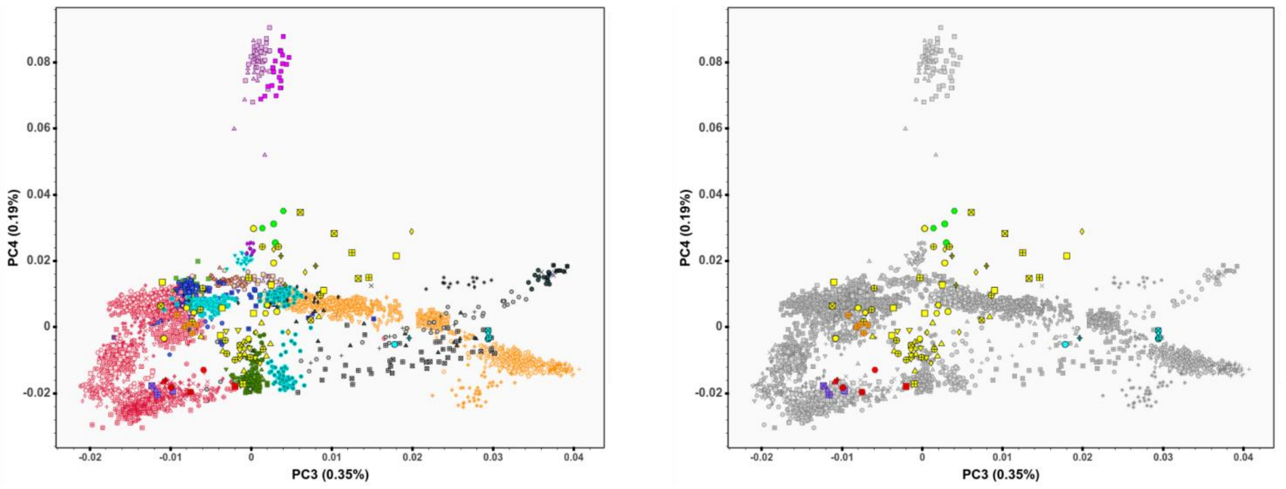

Supplementary Fig. 96 | PCA of aDNA and modern African populations.

Figure showing PCA plot of ancient DNA samples with a background of present-day populations included in the Only-African dataset. **a**, PC1 vs PC2; **b**, PC1 vs PC3; and **c**, PC3 vs PC4. In the left column, groups of present-day populations were highlighted with colors (the same as in Supplementary Fig. 4), and in the right column, present-day samples were highlighted in gray. Ancient DNA samples were highlighted with the colors presented in Supplementary Fig. 95. Interactive plots are available at Github [113] (Suppl_Fig_96_PCA_aDNA_[Group or Population]_[Colour or Gray].html). Legend is presented in the next page.

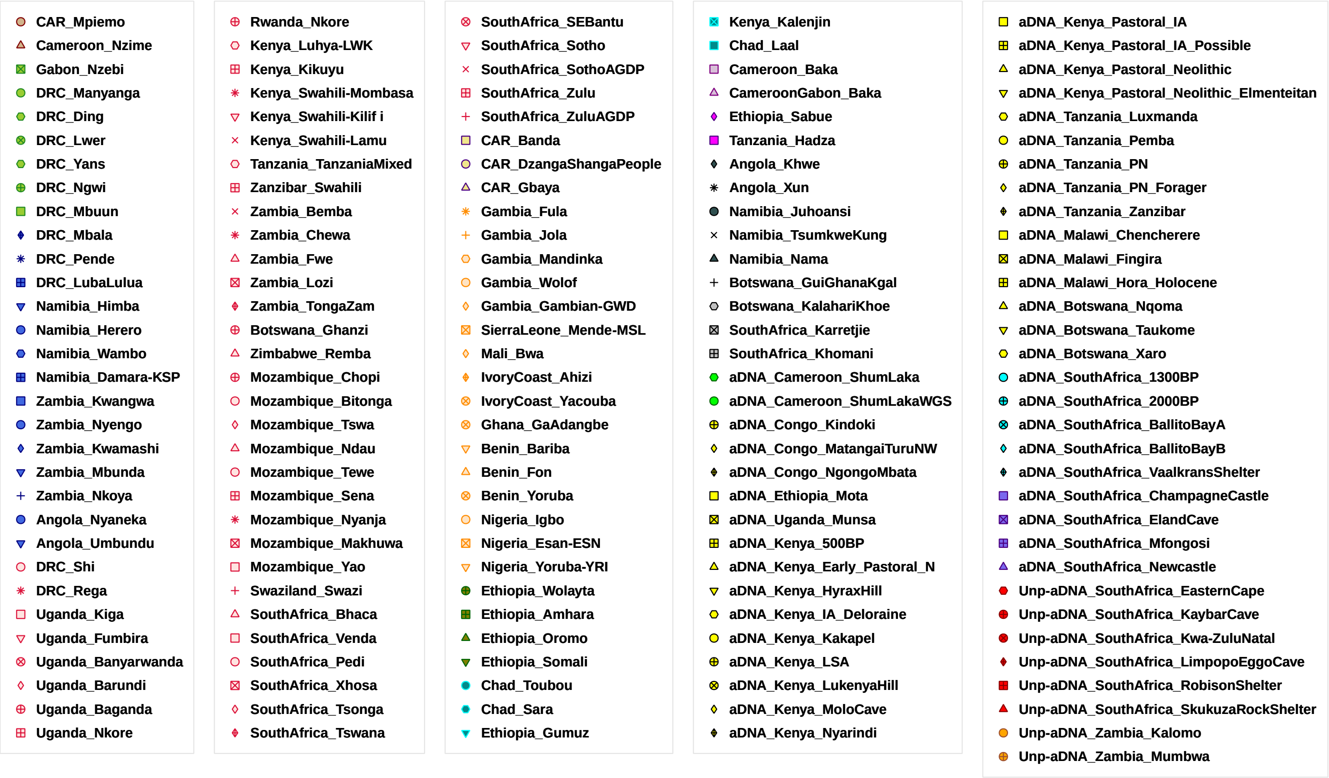

Legend for the modern populations and ancient individuals presented in Supplementary Fig. 96.

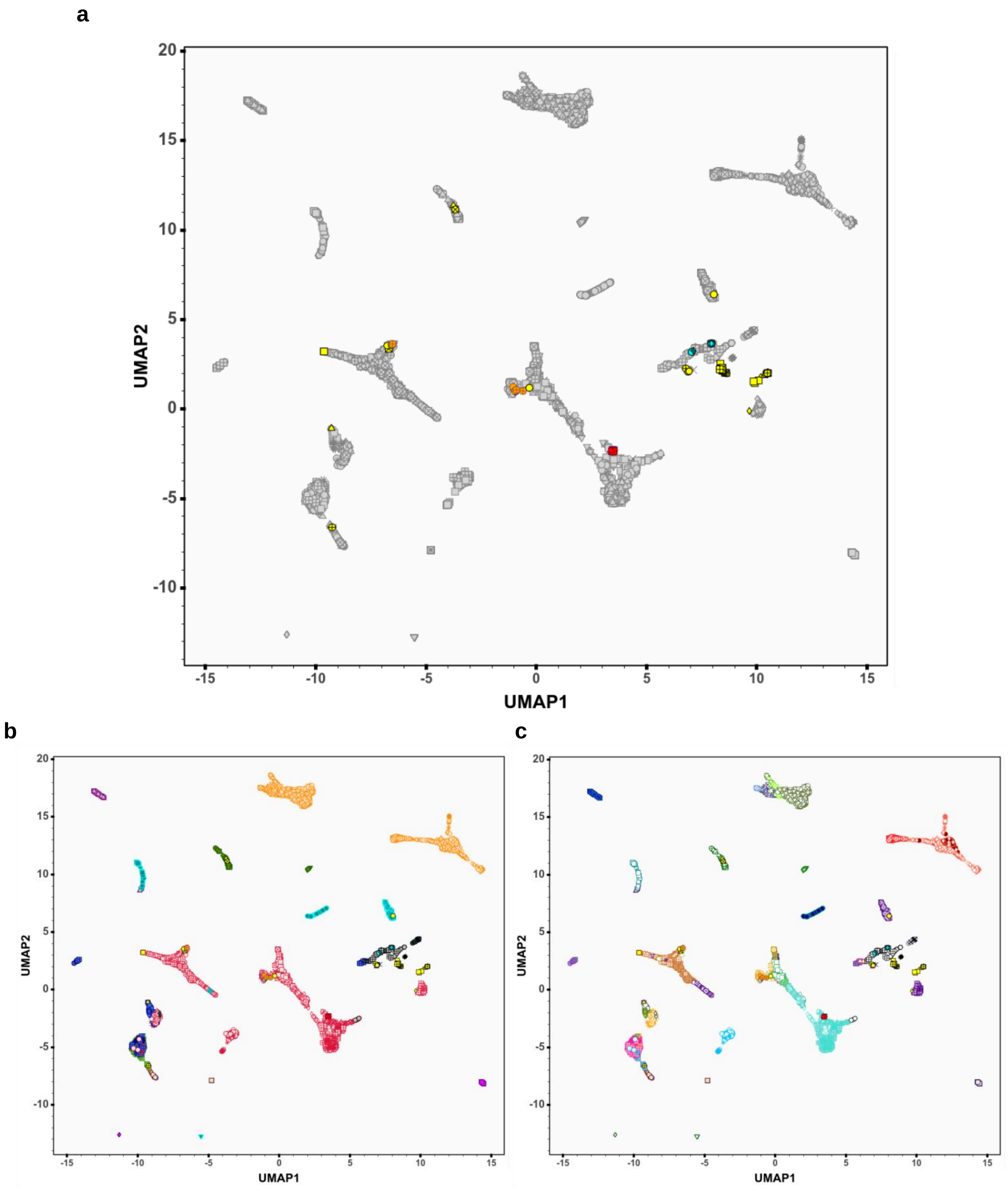

Supplementary Fig. 97 | PCA-UMAP of aDNA individuals and present-day African populations. PCA-UMAP plot of ancient DNA samples with a background of sub-Saharan African populations included in the AfricanNeo dataset **a**, in gray on the top, and on the bottom with colors **b**, for each group and **c**, for each population. To better visualize the results, interactive plots are available at Github [113] (Suppl_Fig_97[a–c]_PCA_aDNA.html). The legends are the same as in Supplementary Fig. 96.

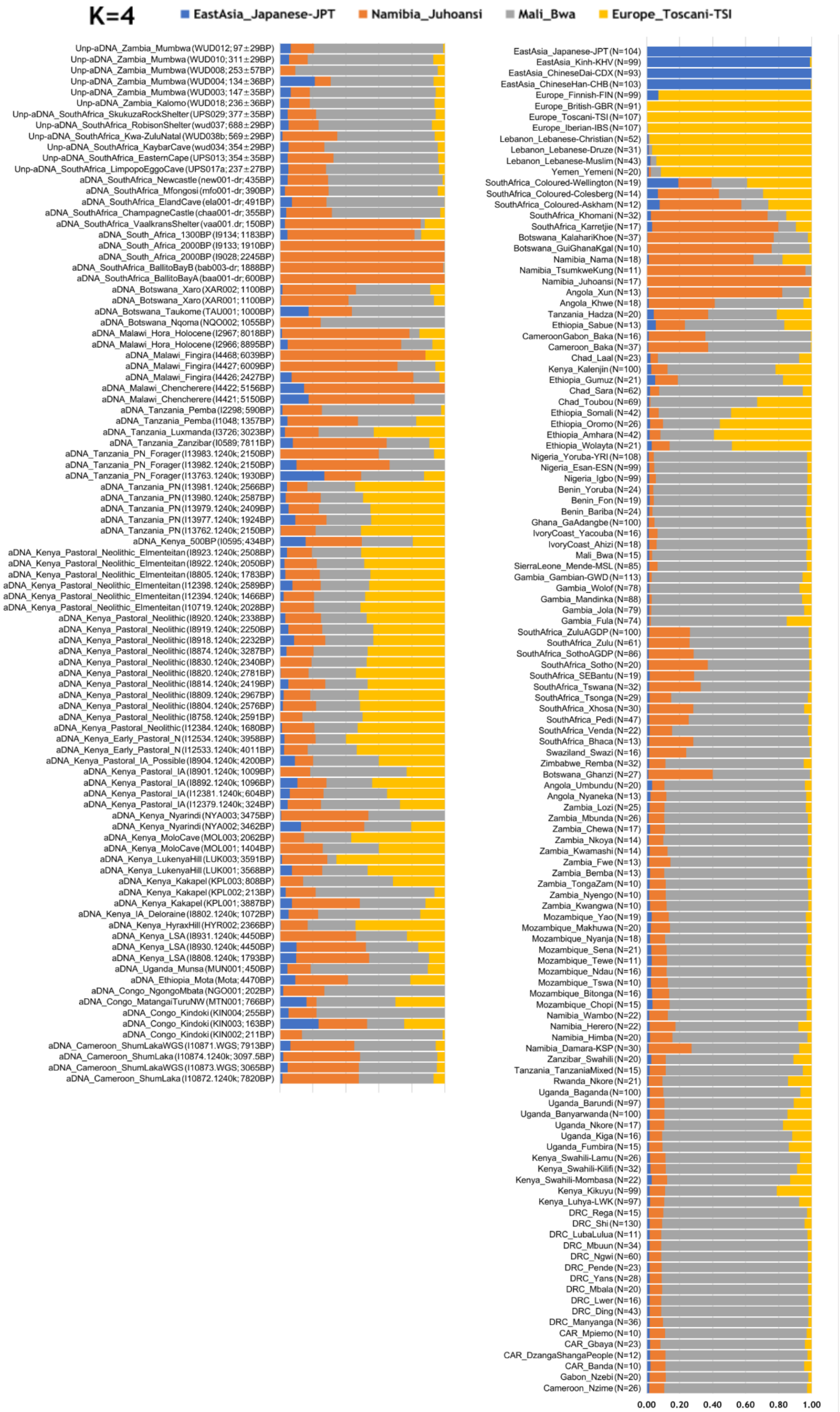

Supplementary Fig. 98 | ADMIXTURE results at K=4 of ancient and modern African populations. Figure showing ADMIXTURE results at K=4 of ancient and modern African and Eurasian populations. For a better comparison, figure showing the averages for each aDNA individual and for each modern population (sample sizes in parenthesis).

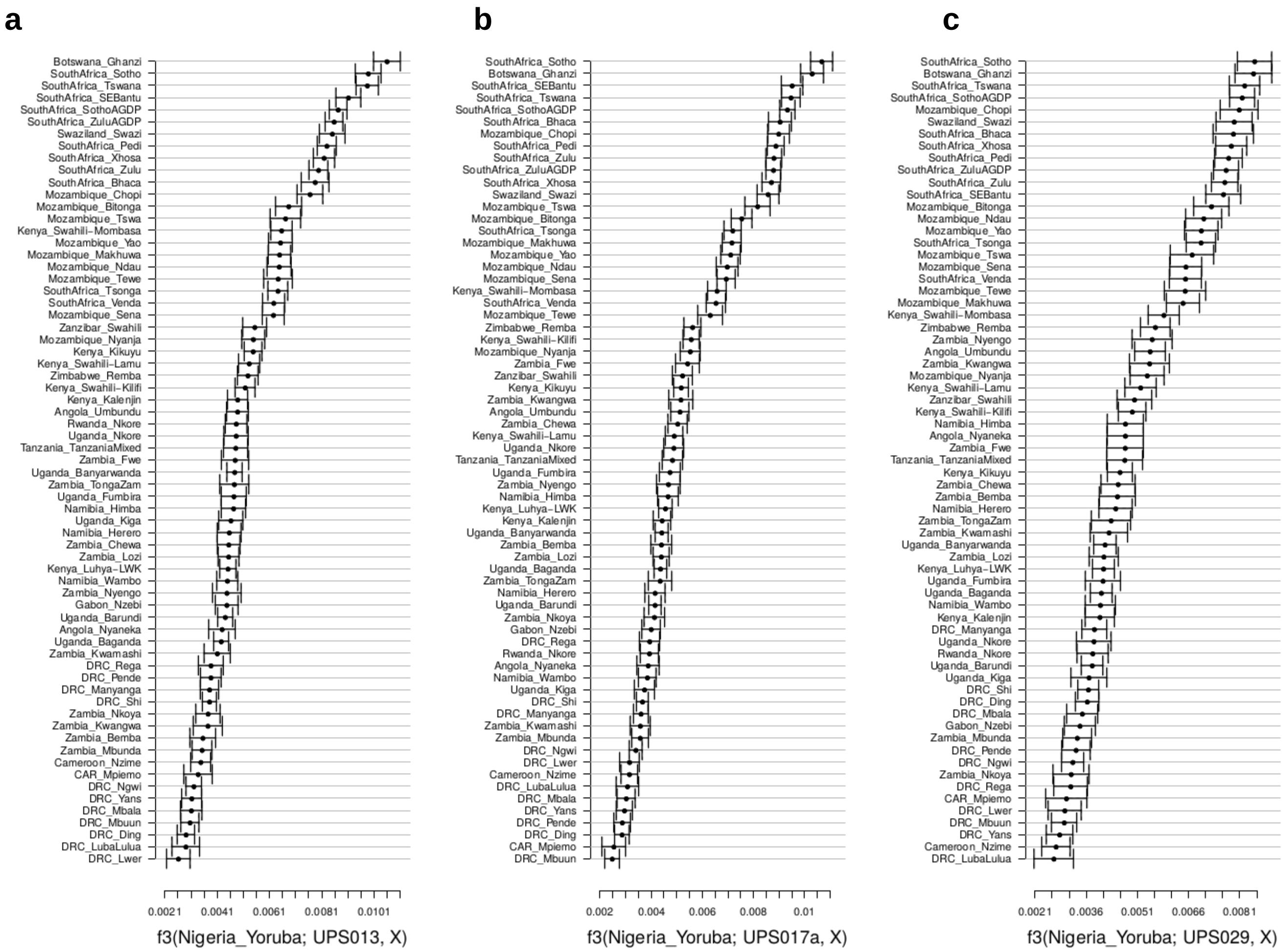

Supplementary Fig. 99 | Genetic affinity of UPS013, UPS017a and UPS029 to modern BSP. Positive values of f3-statistics in the form f3(Yoruba; ancient-sample, BSP) indicate increasing genetic affinity between modern BSP and three ancient individuals from current-day South Africa: **a**, UPS013, **b**, UPS017a, and **c**, UPS029. Bars indicate the standard error of the mean (Supplementary Table 15). Values of the statistic significantly higher than zero (*P*-value < 0.05) are indicated with black color. In each plot, BSP indicated in the Y-axis are ordered according to decreasing values of the f3-statistics. For these three ancient individuals (UPS013, UPS017a, and UPS029) the radio-carbon calibrated ages are 1465-1648 CE, 1649-1806 CE, and 1461-1634 CE, respectively, and the genome coverage percentages are 0.484, 0.942, and 0.367, respectively (Supplementary Table 14).

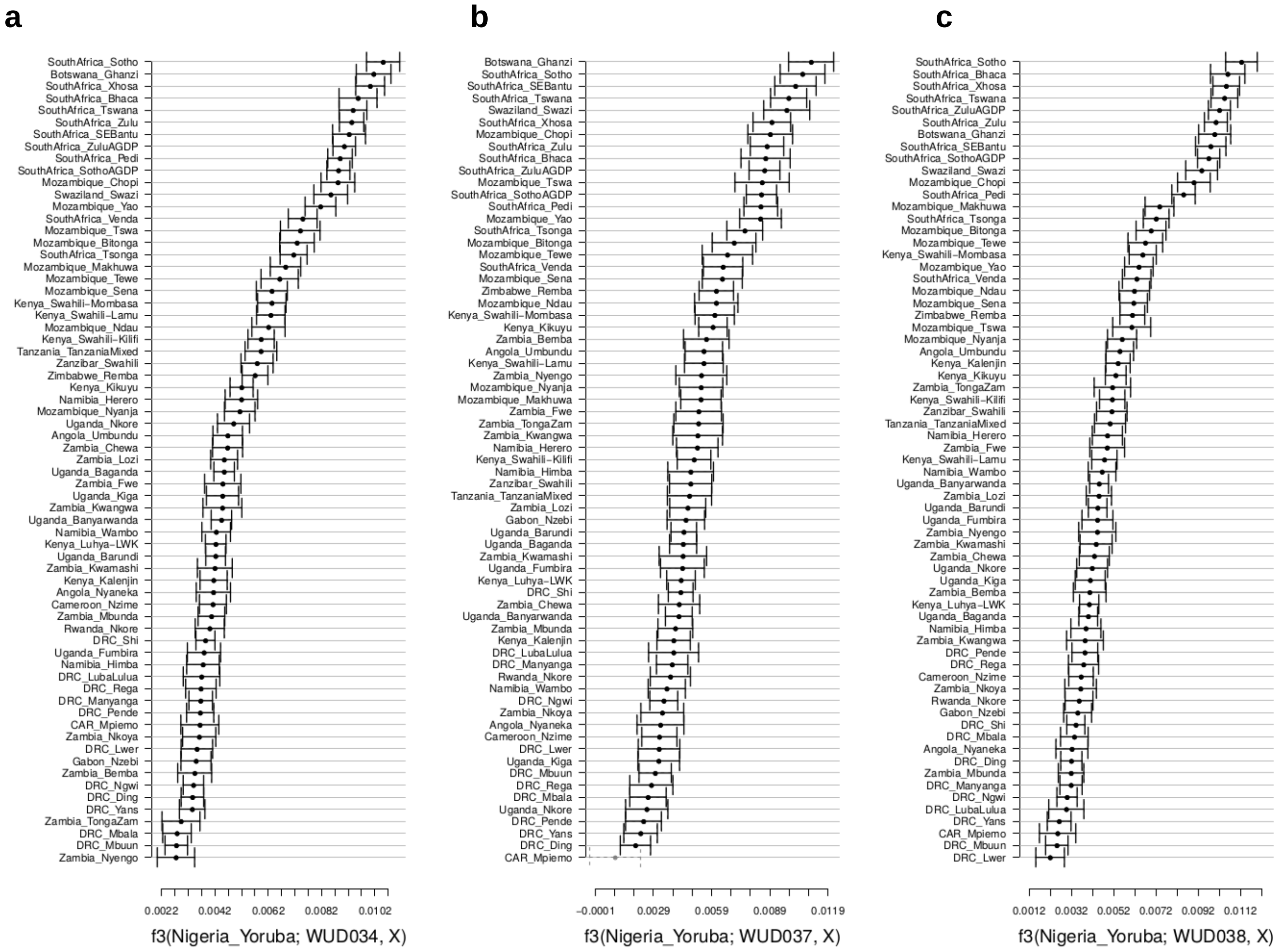

Supplementary Fig. 100 | Genetic affinity of WUD034, WUD037 and WUD038b to modern BSP. Positive values of f3-statistics in the form f3(Yoruba; ancient-sample, BSP) indicate increasing genetic affinity between modern BSP and three ancient individuals from current-day South Africa: **a**, WUD034, **b,** WUD037, and **c**, WUD038b. Bars indicate the standard error of the mean (Supplementary Table 15). Values of the statistic significantly higher than zero (*P*-value < 0.05) are indicated with black color. In each plot, BSP indicated in the Y-axis are ordered according to decreasing values of the f3-statistics. For the three ancient individuals (WUD034, WUD037, and WUD038b) the radio-carbon calibrated ages are 1487–1646 CE, 1289-1394 CE, and 1327-1445 CE, respectively, and the genome coverage percentages are 0.220, 0.055, and 0.144, respectively (Supplementary Table 14).

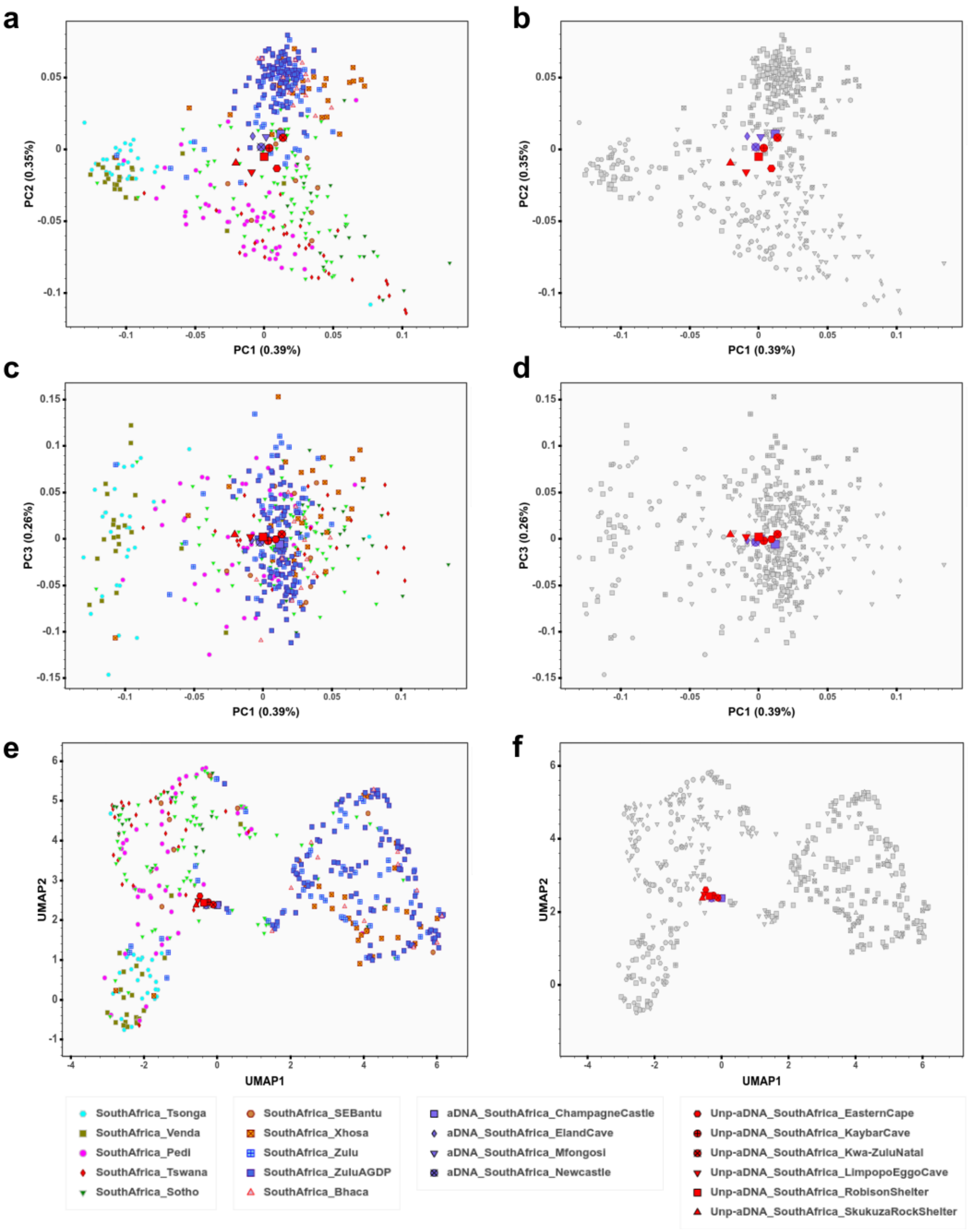

Supplementary Fig. 101 | PCA and PCA-UMAP of ancient individuals and BSP from South Africa. Figure showing **a–d**, PCA (PC1, PC2, and PC3) and **e–f**, PCA-UMAP results of ancient DNA samples onto a background of modern BSP from present-day South Africa. Figure showing in the left column modern BSP with colors and aDNA individuals in red and purple, and in the right column modern BSP in gray and aDNA individuals in red and purple. Interactive plots are available at Github [113] (Suppl_Fig_101[a–f]_PCA_aDNA_SA.html).

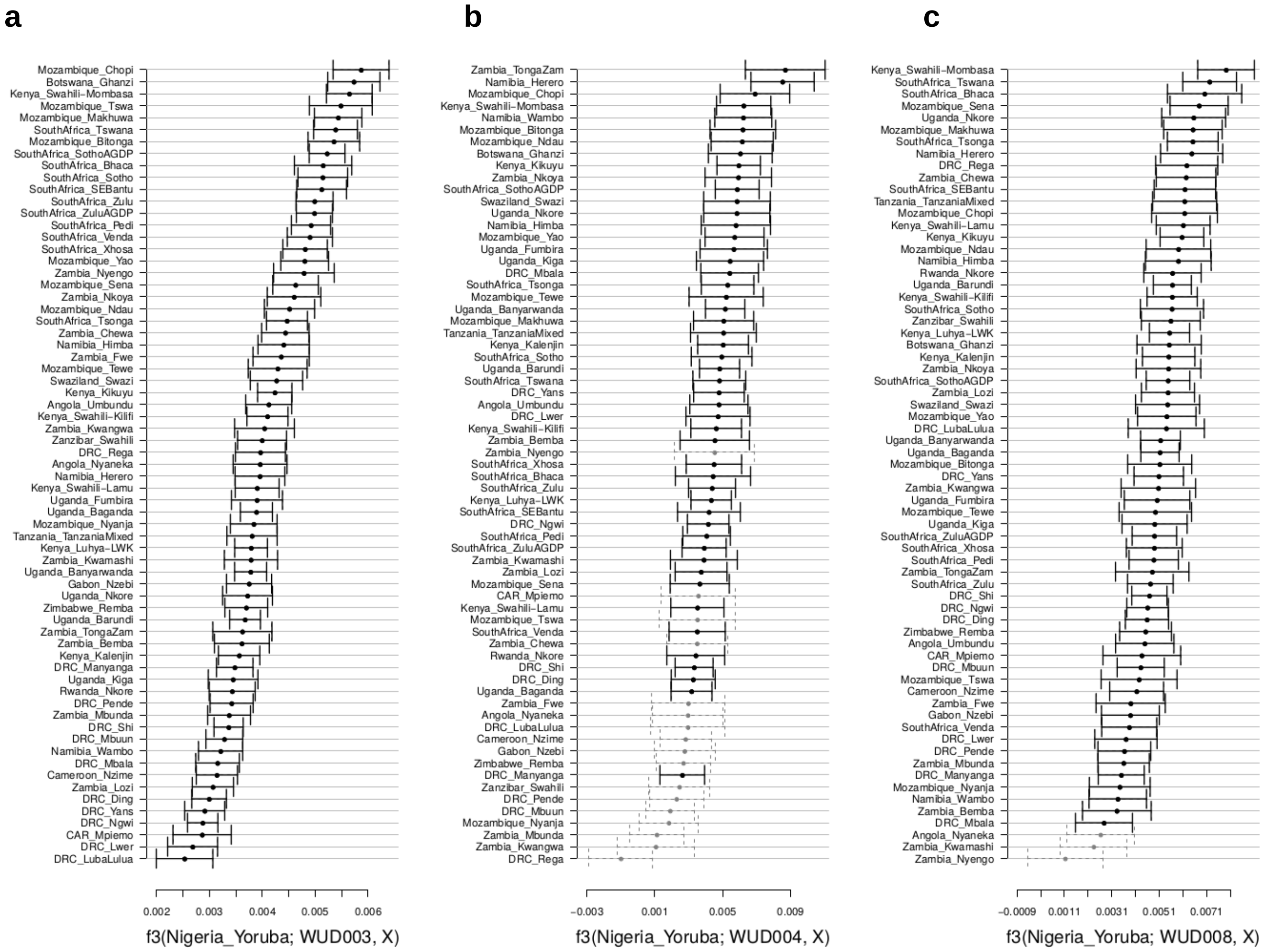

Supplementary Fig. 102 | Genetic affinity of WUD003, WUD004 and WUD008 to modern BSP. Positive values of f3-statistics in the form f3(Yoruba; ancient-sample, BSP) indicate increasing genetic affinity between modern BSP and three ancient individuals from current-day Zambia: **a**, WUD003, **b**, WUD004, and **c**, WUD008. Bars indicate the standard error of the mean (Supplementary Table 15). Values of the statistic significantly higher than zero (*P*-value < 0.05) are indicated with black color. In each plot, BPS indicated in the Y-axis are ordered according to decreasing values of the f3-statistics. For these three ancient individuals (WUD003, WUD004, and WUD008) radio-carbon calibrated age is 1673–1913 CE, 1675–1916 CE and 1506–1880 CE, respectively, and genome coverage percentage is 0.469, 0.020, and 0.036, respectively (Supplementary Table 14).

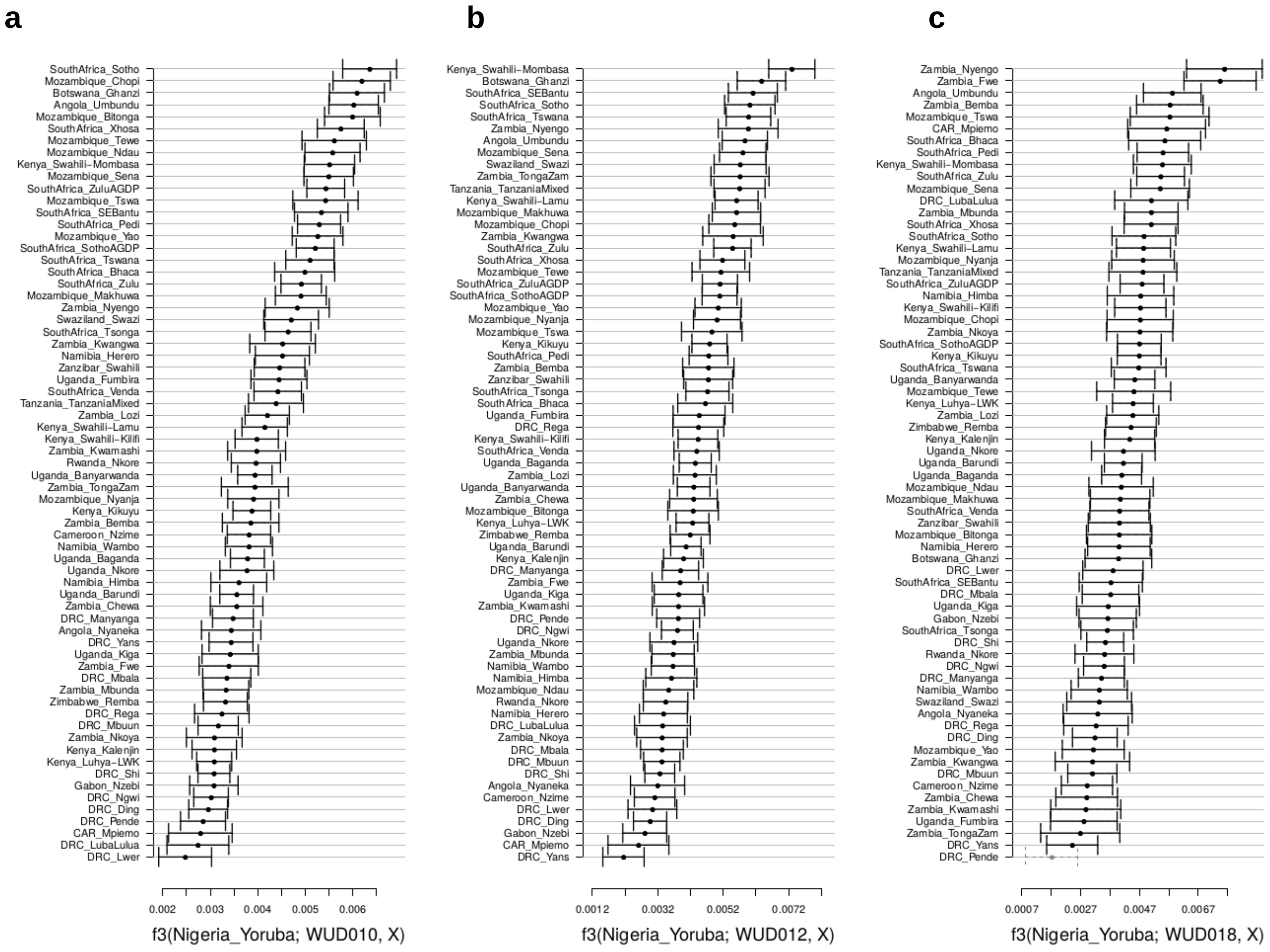

Supplementary Fig. 103 | Genetic affinity of WUD010, WUD012 and WUD018 to modern BSP. Positive values of f3-statistics in the form f3(Yoruba; ancient-sample, BSP) indicate increasing genetic affinity between modern BSP and three ancient individuals from current-day Zambia: **a**, WUD010, **b**, WUD012, and **c**, WUD018. Bars indicate the standard error of the mean. Values of the statistic significantly higher than zero (*P*-value < 0.05) are indicated with black color. In each plot, BSP are ordered according to decreasing values of the f3-statistics. For these three ancient individuals (WUD010, WUD012, and WUD018) radio-carbon calibrated age is 1505–1668 CE, 1698-1950 CE and 1635-1950 CE, respectively, and genome coverage percentage is 0.270, 0.130, and 0.058, respectively (Supplementary Table 14).

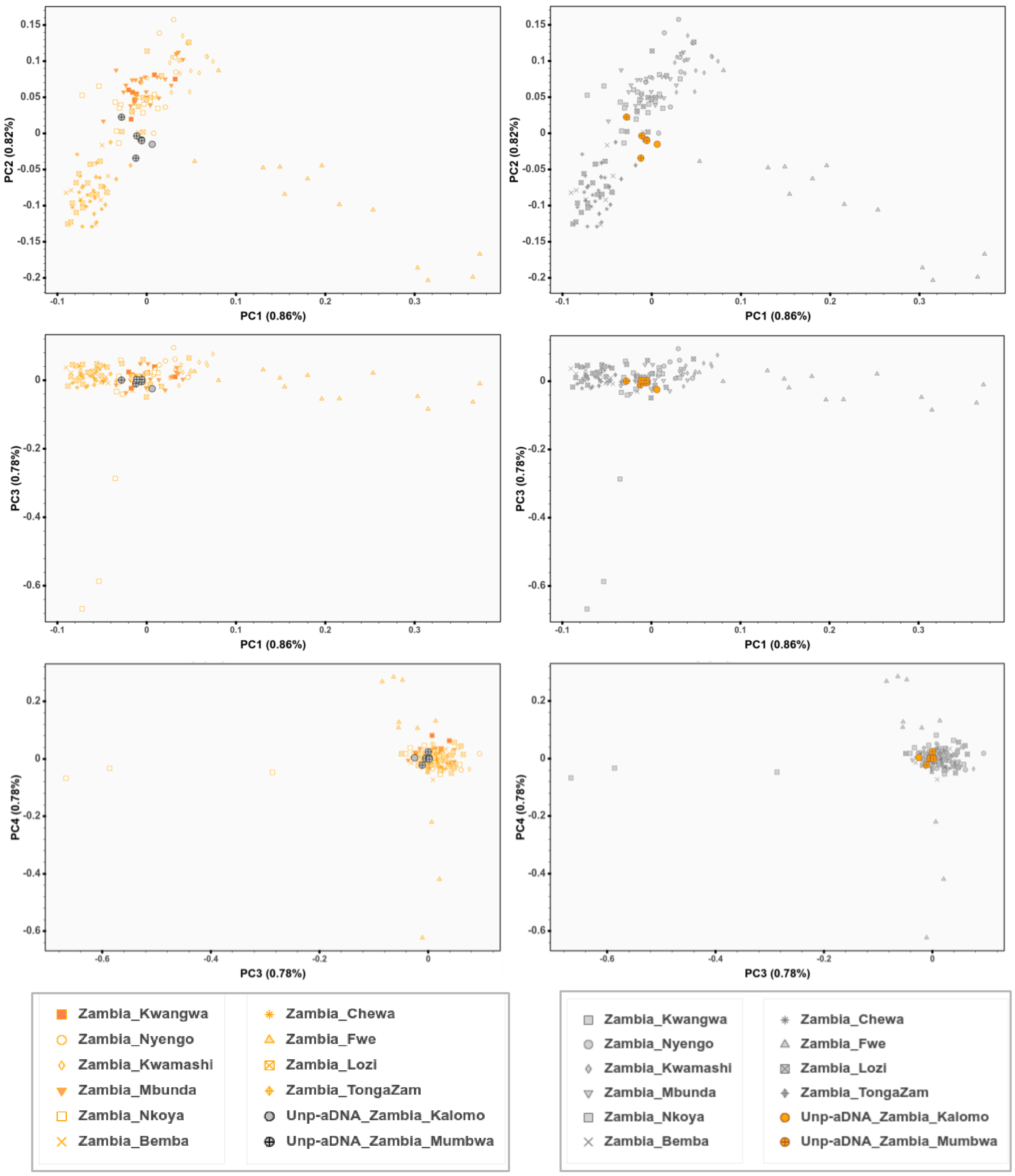

Supplementary Fig. 104 | PCA of ancient individuals and BSP from present-day Zambia. Figure showing PCA plot of ancient DNA samples onto a background of BSP from Zambia. Figure showing in the left column modern BSP in orange and aDNA individuals in gray, and in the right column modern BSP in gray and aDNA individuals in orange.

3.14. PCA results after masking datasets

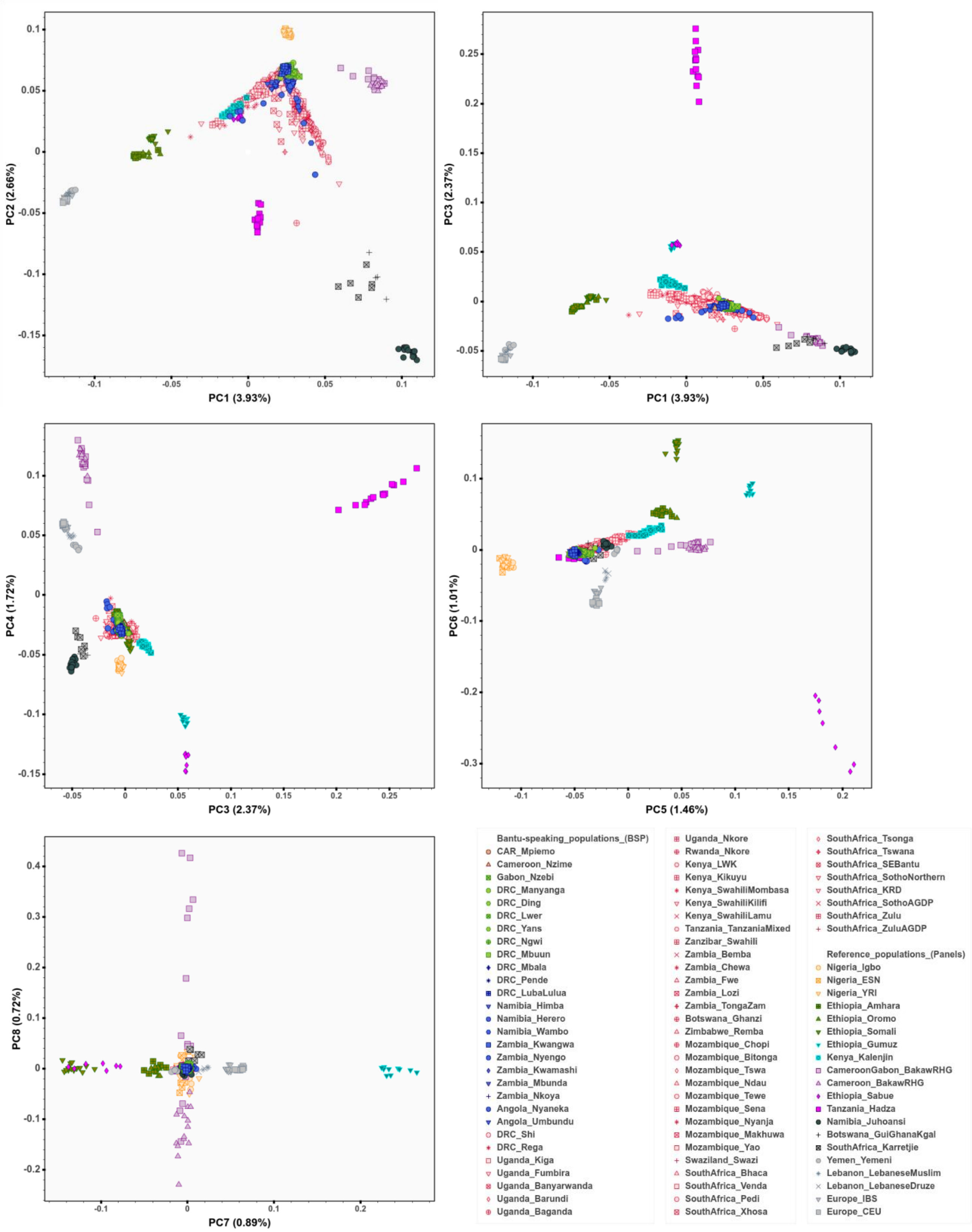

Supplementary Fig. 105 | PCA of BSP and six selected reference panels before using masking. PCA plot of unmasked BSP and unmasked data of selected six reference panels.

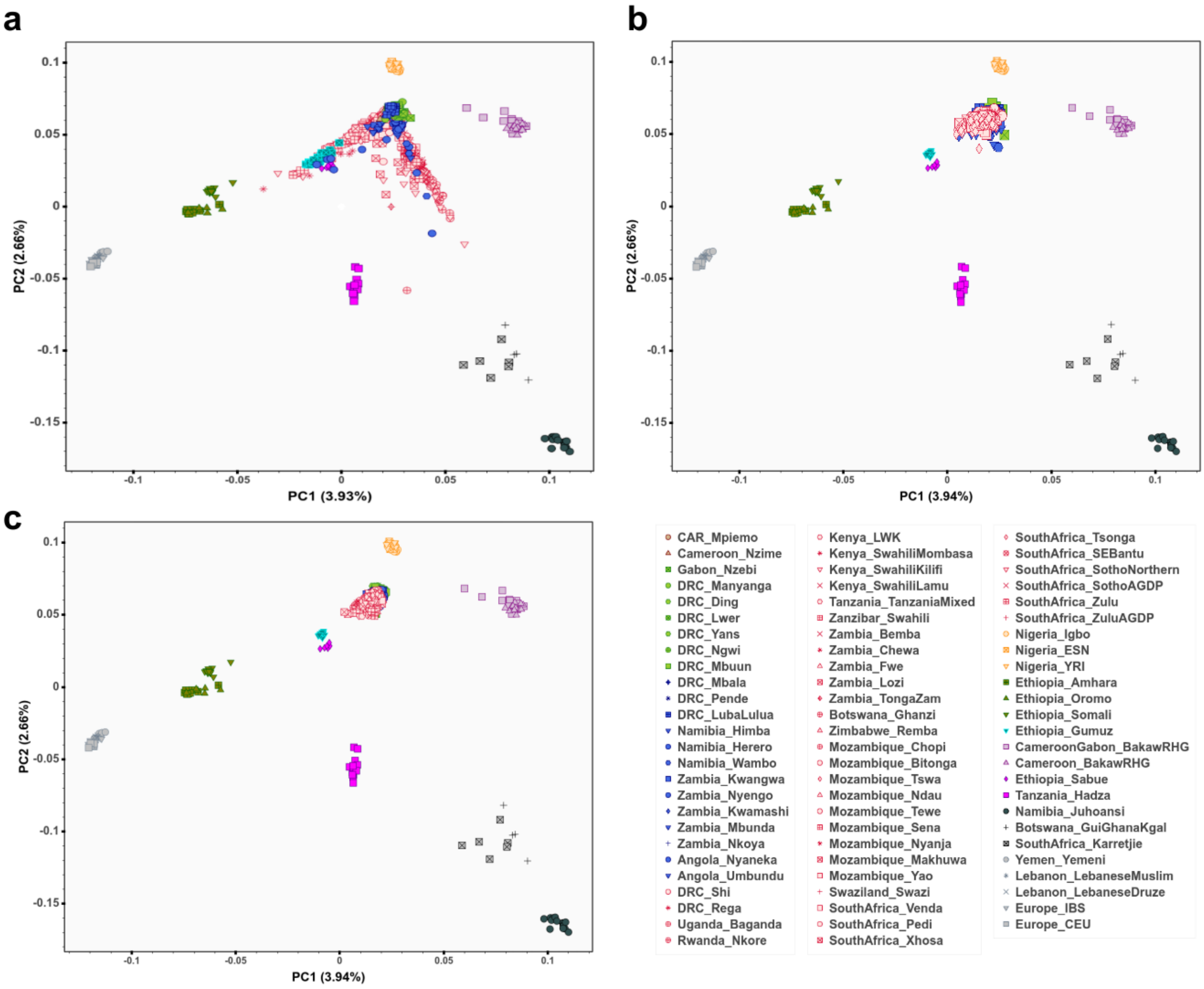

Supplementary Fig. 106 | PCA of masked BSP and six unmasked selected reference panels. PCA plot of masked BSP dataset and unmasked data of selected six reference panels. We used masking phasing and imputation to analyze BSP with at least 70% WCA-related ancestry. **a**, PCA plot of unmasked BSP and unmasked reference datasets. **b**, PCA plot of masked BSP (with 70% WCA and without phasing and imputation) and unmasked reference datasets. **c,** PCA plot of masked BSP (with 70% WCA and after phasing and imputation) and unmasked reference datasets.

a

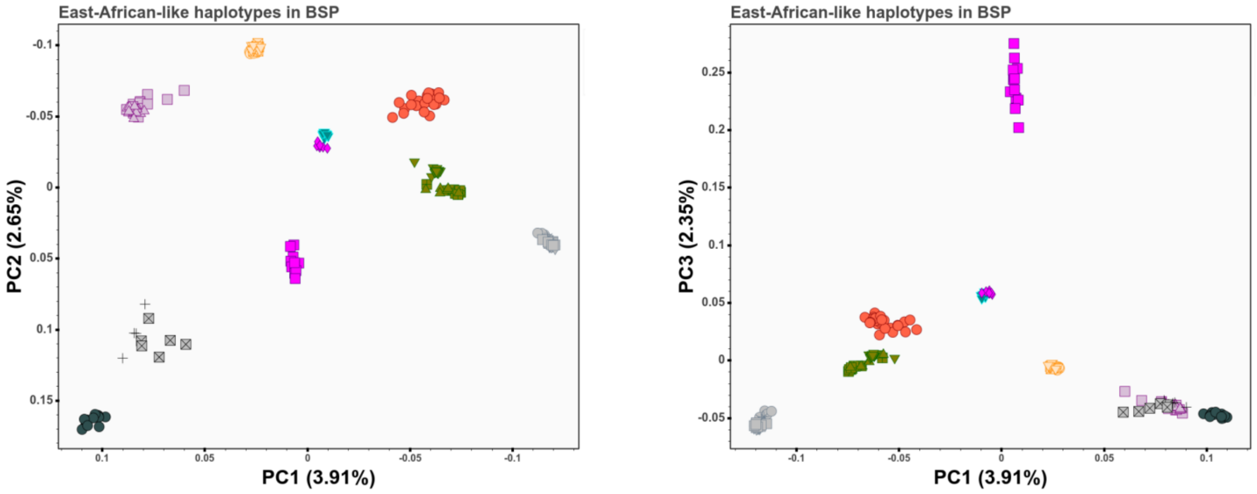
East-African-like haplotypes in BSP
PC2 (2.65%)
PC1 (3.91%)
East-African-like haplotypes in BSP
PC3 (2.35%)
PC1 (3.91%)

b

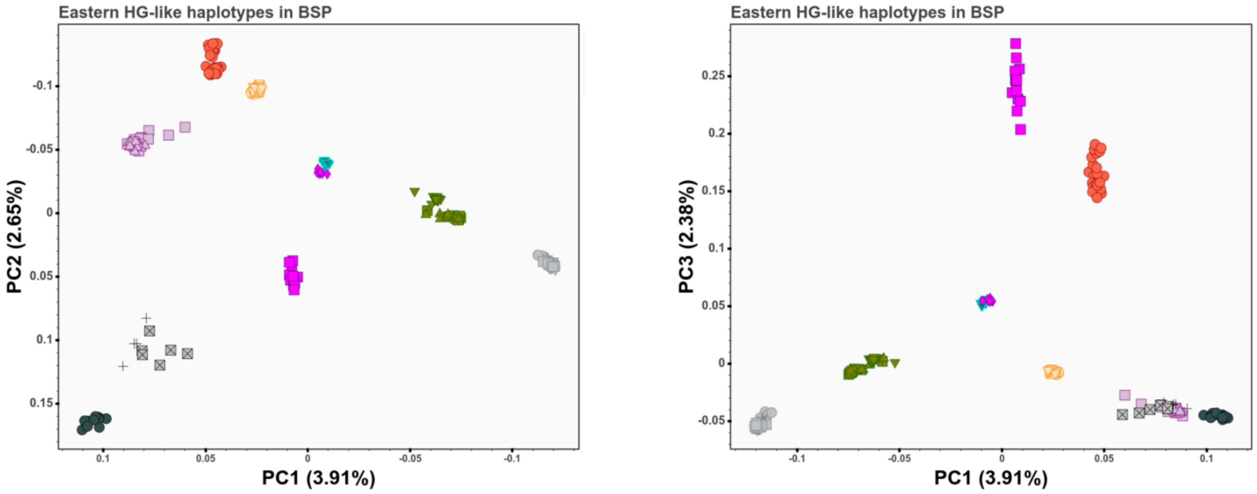
Eastern HG-like haplotypes in BSP
PC2 (2.65%)
PC1 (3.91%)
Eastern HG-like haplotypes in BSP
PC3 (2.38%)
PC1 (3.91%)

C

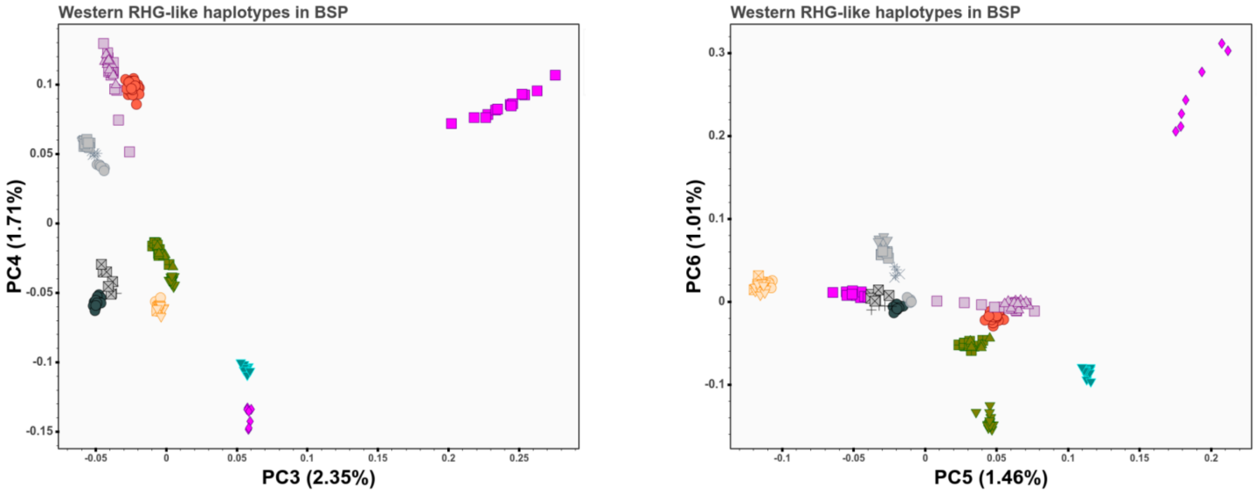
Western RHG-like haplotypes in BSP
PC4 (1.71%)
PC3 (2.35%)
Western RHG-like haplotypes in BSP
PC6 (1.01%)
PC5 (1.46%)

d

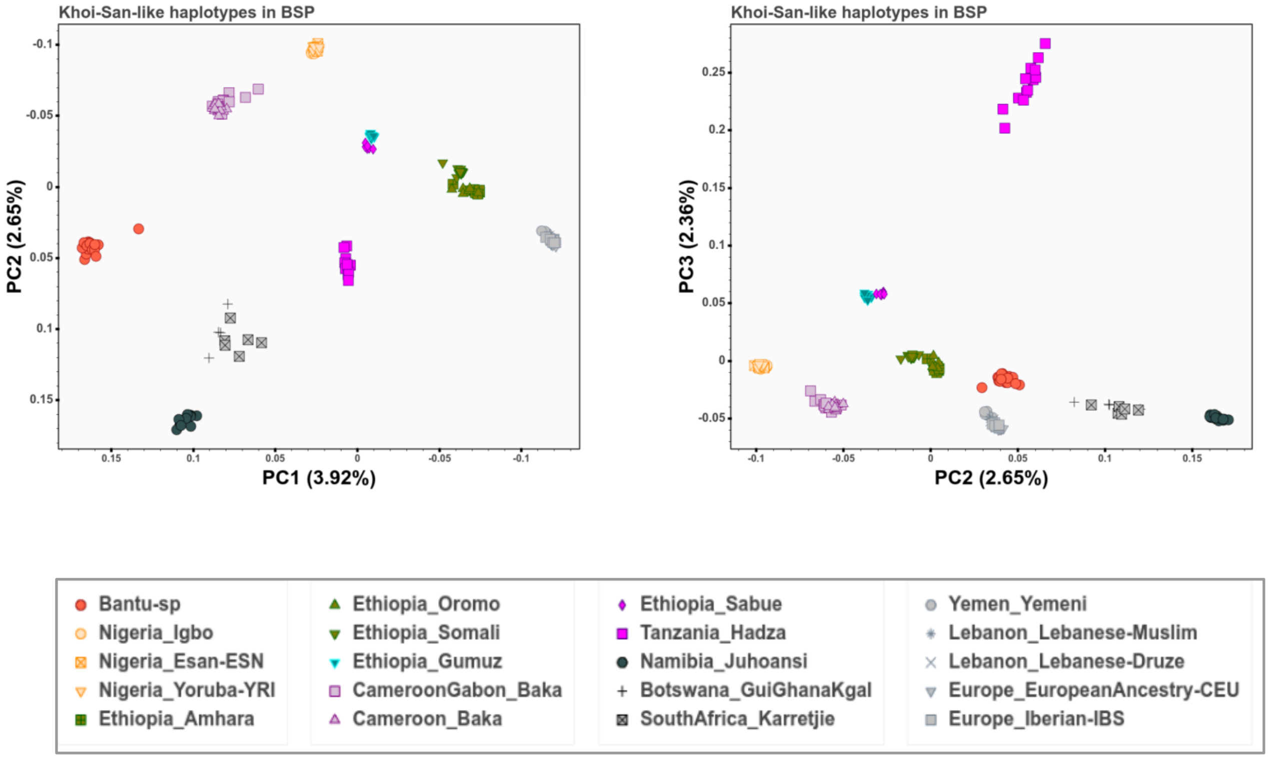

Supplementary Fig. 107 | PCA of masked non-BSP and six unmasked selected reference panels. PCA plot showing non-BSP haplotypes of BSP (red dots)masked for each ancestry that are projected for each ancestry onto the reference groups: **a,** East-African-like haplotypes; **b**, Eastern HG-like haplotypes (e.g., Hadza and Sabue); **c**, Western RHG-like haplotypes (e.g., Baka); and **d**, Khoe-San-like haplotypes. We caution however that we observed previously that ancestry assignments do not work well when shared ancestry with a parental group is below 70% ancestry.

4. Supplementary Information References

75. World Medical Association. World Medical Association Declaration of Helsinki: ethical principles for medical research involving human subjects. *JAMA* **310**, 2191–2194 (2013).
76. Mulder, N. *et al.* H3Africa: current perspectives. *Pharmgenomics. Pers. Med.* **11**, 59–66 (2018).
77. Sherry, S. T. *et al.* dbSNP: the NCBI database of genetic variation. *Nucleic Acids Res.* **29**, 308–311 (2001).
78. Clark, J. D. & Toerien, M. J. Human Skeletal and Cultural Material from a Deep Cave at Chipongwe, Northern Rhodesia. *The South African Archaeological Bulletin* **10**, 107–116 (1955).
79. Ramsey, C. B. Dealing with Outliers and Offsets in Radiocarbon Dating. *Radiocarbon* **51**, 1023–1045 (2009).
80. Hogg, A. G. *et al.* SHCal20 Southern Hemisphere Calibration, 0–55,000 Years cal BP. *Radiocarbon* **62**, 759–778 (2020).
81. Svensson, E. *et al.* Genome of Peştera Muierii skull shows high diversity and low mutational load in pre-glacial Europe. *Curr. Biol.* **31**, 2973–2983.e9 (2021).
82. Dabney, J. *et al.* Complete mitochondrial genome sequence of a Middle Pleistocene cave bear reconstructed from ultrashort DNA fragments. *Proc. Natl. Acad. Sci. U. S. A.* **110**, 15758–15763 (2013).
83. Rohland, N., Glocke, I., Aximu-Petri, A. & Meyer, M. Extraction of highly degraded DNA from ancient bones, teeth and sediments for high-throughput sequencing. *Nat. Protoc.* **13**, 2447–2461 (2018).
84. Meyer, M. & Kircher, M. Illumina sequencing library preparation for highly multiplexed target capture and sequencing. *Cold Spring Harb. Protoc.* **2010**, db.prot5448 (2010).
85. Martin, M. Cutadapt removes adapter sequences from high-throughput sequencing reads. *EMBnet.journal* **17**, 10–12 (2011).
86. Magoč, T. & Salzberg, S. L. FLASH: fast length adjustment of short reads to improve genome assemblies. *Bioinformatics* **27**, 2957–2963 (2011).
87. Li, H. & Durbin, R. Fast and accurate long-read alignment with Burrows–Wheeler transform. *Bioinformatics* **26**, 589–595 (2010).
88. Skoglund, P. *et al.* Genomic diversity and admixture differs for Stone-Age Scandinavian foragers and farmers. *Science* **344**, 747–750 (2014).
89. Kircher, M. Analysis of high-throughput ancient DNA sequencing data. *Methods Mol. Biol.* **840**, 197–228 (2012).
90. Fu, Q. *et al.* A revised timescale for human evolution based on ancient mitochondrial genomes. *Curr. Biol.* **23**, 553–559 (2013).
91. Fu, Q. *et al.* DNA analysis of an early modern human from Tianyuan Cave, China. *Proc. Natl. Acad. Sci. U. S. A.* **110**, 2223–2227 (2013).
92. Jun, G. *et al.* Detecting and estimating contamination of human DNA samples in sequencing and array-based genotype data. *Am. J. Hum. Genet.* **91**, 839–848 (2012).
93. Skoglund, P. *et al.* Origins and genetic legacy of Neolithic farmers and hunter-gatherers in Europe. *Science* **336**, 466–469 (2012).
94. Skoglund, P., Storå, J., Götherström, A. & Jakobsson, M. Accurate sex identification of ancient human remains using DNA shotgun sequencing. *J. Archaeol. Sci.* **40**, 4477–4482 (2013).

95. Li, H. *et al.* The Sequence Alignment/Map format and SAMtools. *Bioinformatics* **25**, 2078–2079 (2009).
96. Weissensteiner, H. *et al.* HaploGrep 2: mitochondrial haplogroup classification in the era of high-throughput sequencing. *Nucleic Acids Research* **44**, W58–W63 (2016).
97. van Oven, M. PhyloTree Build 17: Growing the human mitochondrial DNA tree. *Forensic Science International: Genetics Supplement Series* **5**, e392–e394 (2015).
98. Manichaikul, A. *et al.* Robust relationship inference in genome-wide association studies. *Bioinformatics* **26**, 2867–2873 (2010).
99. Conomos, M. P., Reiner, A. P., Weir, B. S. & Thornton, T. A. Model-free Estimation of Recent Genetic Relatedness. *Am. J. Hum. Genet.* **98**, 127–148 (2016).
100. 1000 Genomes Project Consortium *et al.* A global reference for human genetic variation. *Nature* **526**, 68–74 (2015).
101. Vicente, M., Jakobsson, M., Ebbesen, P. & Schlebusch, C. M. Genetic Affinities among Southern Africa Hunter-Gatherers and the Impact of Admixing Farmer and Herder Populations. *Mol. Biol. Evol.* **36**, 1849–1861 (2019).
102. Brucato, N. *et al.* The Comoros Show the Earliest Austronesian Gene Flow into the Swahili Corridor. *Am. J. Hum. Genet.* **102**, 58–68 (2018).
103. Montinaro, F. *et al.* Complex Ancient Genetic Structure and Cultural Transitions in Southern African Populations. *Genetics* **205**, 303–316 (2017).
104. Crawford, N. G. *et al.* Loci associated with skin pigmentation identified in African populations. *Science* **358**, eaan8433 (2017).
105. Haber, M. *et al.* Chad Genetic Diversity Reveals an African History Marked by Multiple Holocene Eurasian Migrations. *Am. J. Hum. Genet.* **99**, 1316–1324 (2016).
106. Lopez, M. *et al.* Genomic Evidence for Local Adaptation of Hunter-Gatherers to the African Rainforest. *Current Biology* **29**, 2926–2935.e4 (2019).
107. Sjöstrand, A. E. *et al.* Taste perception and lifestyle: insights from phenotype and genome data among Africans and Asians. *Eur. J. Hum. Genet.* **29**, 325–337 (2021).
108. Fortes-Lima, C. *et al.* Genome-wide Ancestry and Demographic History of African-Descendant Maroon Communities from French Guiana and Suriname. *Am. J. Hum. Genet.* **101**, 725–736 (2017).
109. Verdu, P. African Pygmies. *Curr. Biol.* **26**, R12–4 (2016).
110. Patin, E. *et al.* The impact of agricultural emergence on the genetic history of African rainforest hunter-gatherers and agriculturalists. *Nat. Commun.* **5**, 3163 (2014).
111. Pickrell, J. K. *et al.* Ancient west Eurasian ancestry in southern and eastern Africa. *Proc. Natl. Acad. Sci. U. S. A.* **111**, 2632–2637 (2014).
112. Bokeh Development Team, C. M. Bokeh: Python library for interactive visualization. Preprint at (2014).
113. Fortes-Lima, C., Hammarén, R., Burgarella, C. & Schlebusch, C. M. Online interactive plots presented in Fortes-Lima et al. 2023. *Schlebusch-lab at Github* https://github.com/Schlebusch-lab/Expansion_of_BSP_peer-reviewed_article.
114. Alexander, D. H. & Lange, K. Enhancements to the ADMIXTURE algorithm for individual ancestry estimation. *BMC Bioinformatics* **12**, 246 (2011).
115. Behr, A. A., Liu, K. Z., Liu-Fang, G., Nakka, P. & Ramachandran, S. pong: fast analysis and visualization of latent clusters in population genetic data. *Bioinformatics* **32**, 2817–2823 (2016).
116. Feng, Q., Lu, D. & Xu, S. AncestryPainter: A Graphic Program for Displaying Ancestry Composition of

Populations and Individuals. *Genomics Proteomics Bioinformatics* **16**, 382–385 (2018).
117. McCarthy, S. *et al.* A reference panel of 64,976 haplotypes for genotype imputation. *Nat. Genet.* **48**, 1279–1283 (2016).
118. Howie, B. N., Donnelly, P. & Marchini, J. A flexible and accurate genotype imputation method for the next generation of genome-wide association studies. *PLoS Genet.* **5**, e1000529 (2009).
119. Marchini, J., Howie, B., Myers, S., McVean, G. & Donnelly, P. A new multipoint method for genome-wide association studies by imputation of genotypes. *Nat. Genet.* **39**, 906–913 (2007).
120. Ceballos, F. C., Hazelhurst, S. & Ramsay, M. Runs of homozygosity in sub-Saharan African populations provide insights into complex demographic histories. *Human Genetics* **138**, 1123–1142 (2019).
121. Ceballos, F. C., Joshi, P. K., Clark, D. W., Ramsay, M. & Wilson, J. F. Runs of homozygosity: windows into population history and trait architecture. *Nat. Rev. Genet.* **19**, 220–234 (2018).
122. McQuillan, R. *et al.* Evidence of inbreeding depression on human height. *PLoS Genet.* **8**, e1002655 (2012).
123. Browning, B. L. & Browning, S. R. A fast, powerful method for detecting identity by descent. *Am. J. Hum. Genet.* **88**, 173–182 (2011).
124. Chiang, C. W. K., Ralph, P. & Novembre, J. Conflation of Short Identity-by-Descent Segments Bias Their Inferred Length Distribution. *G3* **6**, 1287–1296 (2016).
125. Browning, S. R. *et al.* Ancestry-specific recent effective population size in the Americas. *PLoS Genet.* **14**, e1007385 (2018).
126. Fenner, J. N. Cross-cultural estimation of the human generation interval for use in genetics-based population divergence studies. *Am. J. Phys. Anthropol.* **128**, 415–423 (2005).
127. Huang, L. *et al.* Haplotype variation and genotype imputation in African populations. *Genet. Epidemiol.* **35**, 766–780 (2011).
128. Kumar, S., Stecher, G., Li, M., Knyaz, C. & Tamura, K. MEGA X: Molecular Evolutionary Genetics Analysis across computing platforms. *Mol. Biol. Evol.* **35**, 1547–1549 (2018).
129. Pless, E., Eckburg, A. M. & Henn, B. M. Predicting environmental and ecological drivers of human population structure. *Mol. Biol. Evol.* **40**, (2023).
130. Zhou, Y., Browning, S. R. & Browning, B. L. A Fast and Simple Method for Detecting Identity-by-Descent Segments in Large-Scale Data. *Am. J. Hum. Genet.* **106**, 426–437 (2020).
131. Loh, P.-R. *et al.* Reference-based phasing using the Haplotype Reference Consortium panel. *Nat. Genet.* **48**, 1443–1448 (2016).
132. Tadmor, U., Haspelmath, M. & Taylor, B. Borrowability and the notion of basic vocabulary. *Diachronica* **27**, 226–246 (2010).
133. De Schryver, G.-M., Grollemund, R., Branford, S. & Bostoen, K. A. G. Introducing a state-of-the-art phylogenetic classification of the Kikongo Language Cluster. *Afr. Linguist.* **21**, 87–162 (2015).
134. Pacchiarotti, S., Chousou-Polydouri, N. & Bostoen, K. Untangling the West-Coastal Bantu mess : identification, geography and phylogeny of the Bantu B50-80 languages. *Africana Linguistica* **25**, 155–229 (2019).
135. Kaiping, G. A., Steiger, M. S. & Chousou-Polydouri, N. Lexedata: A toolbox to edit CLDF lexical datasets. *Journal of Open Source Software* **7**, 4140 (2022).
136. Forkel, R. *et al.* Cross-Linguistic Data Formats, advancing data sharing and re-use in comparative linguistics. *Sci Data* **5**, 180205 (2018).

137. Phillipson, L. *African archaeology*. (Cambridge University Press, 2005).
138. Mitchell, P. Hunter-gatherer archaeology in southern Africa. *Before Farming* vol. 2002 1–36 Preprint at https://doi.org/10.3828/bfarm.2002.1.3 (2002).
139. Newman, J. L. *The Peopling of Africa: A Geographic Interpretation*. (Yale University Press, 1995).
140. Vicente, M. *et al.* Population history and genetic adaptation of the Fulani nomads: inferences from genome-wide data and the lactase persistence trait. *BMC Genomics* **20**, 915 (2019).
141. Černý, V., Fortes-Lima, C. & Tříska, P. Demographic history and admixture dynamics in African Sahelian populations. *Hum. Mol. Genet.* **30**, R29–R36 (2021).
142. Fortes-Lima, C. *et al.* Demographic and Selection Histories of Populations Across the Sahel/Savannah Belt. *Mol. Biol. Evol.* **39**, msac209 (2022).
143. Uren, C. *et al.* Fine-Scale Human Population Structure in Southern Africa Reflects Ecogeographic Boundaries. *Genetics* **204**, 303–314 (2016).
144. Lachance, J. *et al.* Evolutionary history and adaptation from high-coverage whole-genome sequences of diverse African hunter-gatherers. *Cell* **150**, 457–469 (2012).
145. Gopalan, S. *et al.* Hunter-gatherer genomes reveal diverse demographic trajectories following the rise of farming in East Africa. *Curr Biol* **32**, 1852–1860.e5 (2022).
146. Li, H. & Durbin, R. Inference of human population history from individual whole-genome sequences. *Nature* **475**, 493–496 (2011).
147. Ardlie, K. G., Kruglyak, L. & Seielstad, M. Patterns of linkage disequilibrium in the human genome. *Nat. Rev. Genet.* **3**, 299–309 (2002).
148. Maho, J. F. The online version of the New Updated Guthrie List, a referential classification of the Bantu languages. *Unpublished Manuscript. Available at: http://goto. glocalnet. net/maho/papers. html* 1–124 (2009).
149. Schlebusch, C. M., Naidoo, T. & Soodyall, H. SNaPshot minisequencing to resolve mitochondrial macro- haplogroups found in Africa. *Electrophoresis* (2009).
150. Schlebusch, C. M., Lombard, M. & Soodyall, H. MtDNA control region variation affirms diversity and deep sub-structure in populations from southern Africa. *BMC Evol. Biol.* **13**, 56 (2013).
