# Supplementary material for: The genetic legacy of the expansion of Bantu-speaking peoples in Africa
Source: Nature. 2023 Nov 29;625(7995):540–7. doi: 10.1038/s41586-023-06770-6 (PMC10794141; doi:10.1038/s41586-023-06770-6)
Supplement: Supplementary file 2 — Reporting Summary [file 41586_2023_6770_MOESM2_ESM.pdf]

nature portfolio

Corresponding author(s): Carina Schlebusch

Last updated by author(s): Sep 18, 2023

Reporting Summary

Nature Portfolio wishes to improve the reproducibility of the work that we publish. This form provides structure for consistency and transparency in reporting. For further information on Nature Portfolio policies, see our Editorial Policies and the Editorial Policy Checklist.

Statistics

For all statistical analyses, confirm that the following items are present in the figure legend, table legend, main text, or Methods section.

n/a	Confirmed	
☐	☒	The exact sample size (*n*) for each experimental group/condition, given as a discrete number and unit of measurement
☐	☒	A statement on whether measurements were taken from distinct samples or whether the same sample was measured repeatedly
☐	☒	The statistical test(s) used AND whether they are one- or two-sided<br>*Only common tests should be described solely by name; describe more complex techniques in the Methods section.*
☒	☐	A description of all covariates tested
☒	☐	A description of any assumptions or corrections, such as tests of normality and adjustment for multiple comparisons
☐	☒	A full description of the statistical parameters including central tendency (e.g. means) or other basic estimates (e.g. regression coefficient) AND variation (e.g. standard deviation) or associated estimates of uncertainty (e.g. confidence intervals)
☐	☒	For null hypothesis testing, the test statistic (e.g. *F*, *t*, *r*) with confidence intervals, effect sizes, degrees of freedom and *P* value noted<br>*Give P values as exact values whenever suitable.*
☒	☐	For Bayesian analysis, information on the choice of priors and Markov chain Monte Carlo settings
☒	☐	For hierarchical and complex designs, identification of the appropriate level for tests and full reporting of outcomes
☒	☐	Estimates of effect sizes (e.g. Cohen's *d*, Pearson's *r*), indicating how they were calculated

Our web collection on statistics for biologists contains articles on many of the points above.

Software and code

Policy information about availability of computer code

Data collection	OxCal v4.4, ContamMix, SHCal20, HaploGrep2, GenomeStudio v2.0.3, PLINK v1.9
Data analysis	ADMIXTOOLS v2, Eigensoft v7.2.1, ADMIXTURE v1.3.0, SHAPEIT v2.r904, RFMix v1.5.4, MOSAIC v1.4, KIN v1.4, IBDNe (ibdne.19Sep19.268.jar), PLINK v1.9, SpaceMix v0.13, ASCEND v10, Beagle v4.1, TreeMix v1.13, in-house R and Python scripts (were included here: https://github.com/Schlebusch-lab/Expansion_of_BSP_peer-reviewed_article), EEMS, FEEMS, GenGrad.

For manuscripts utilizing custom algorithms or software that are central to the research but not yet described in published literature, software must be made available to editors and reviewers. We strongly encourage code deposition in a community repository (e.g. GitHub). See the Nature Portfolio guidelines for submitting code & software for further information.

Data

Policy information about availability of data

All manuscripts must include a data availability statement. This statement should provide the following information, where applicable:

- Accession codes, unique identifiers, or web links for publicly available datasets
- A description of any restrictions on data availability
- For clinical datasets or third party data, please ensure that the statement adheres to our policy

Novel genome-wide genotype data of modern-day African populations (*.tped and *.tfam files) and whole-genome data of aDNA individuals (*.bam files) generated in this study will be made available through the European Genome-phenome Archive (EGA) data repository (EGA accessory numbers: EGAS00001007519 and

EGAS00001007515). Controlled access policies guided by participant consent agreements will be implemented by the AfricanNeo Data Access Committee (DAC accessory number: EGAC00001003398).

Research involving human participants, their data, or biological material

Policy information about studies with human participants or human data. See also policy information about sex, gender (identity/presentation), and sexual orientation and race, ethnicity and racism.

Reporting on sex and gender	Biological samples were obtained from any participants of both sex, and sex or gender were not a factor in the sampling collection.
Reporting on race, ethnicity, or other socially relevant groupings	New samples presented in this study were collected in large-scale sampling campaigns conducted in fourteen sub-Saharan African countries (1,763 individuals in total). Participants were recruited based on self identification as member of a specific ethno-linguist group in Africa.
Population characteristics	Biological samples were collected from healthy adults.
Recruitment	All the individuals who participated in sample collection provided informed consent.
Ethics oversight	Ethical permits and sampling permission were obtained in African countries and the study as a whole was approved by the Swedish ethical review board (DNR-2021-01448). Granted ethics was approval by: the Human Research Ethics Committee (Medical) (University of the Witwatersrand, South Africa; protocol Nr. M180656); the Biomedical Research Ethics Board (University of Zambia,Zambia; protocol number: 004-08-07); the Faculty of Natural and Agricultural Sciences Ethics Committee (University of Pretoria, South Africa; protocol number: EC160429-024 and 259/2016); the Swedish National Ethics Committee (Sweden; protocol number: Dnr 2019-05244), and the Minister of Arts and Culture (DRC; protocol number: Nr 091/CAB/MIN/CA/PKB/2018).

Note that full information on the approval of the study protocol must also be provided in the manuscript.

Field-specific reporting

Please select the one below that is the best fit for your research. If you are not sure, read the appropriate sections before making your selection.

☒ Life sciences ☐ Behavioural & social sciences ☐ Ecological, evolutionary & environmental sciences

For a reference copy of the document with all sections, see nature.com/documents/nr-reporting-summary-flat.pdf

Life sciences study design

All studies must disclose on these points even when the disclosure is negative.

Sample size	Our analyses are based on the data we collected from modern and ancient individuals. Standard errors are reported to describe ranges of our analyses.
Data exclusions	We exclude individuals with low SNP-genotyping rates for modern samples or low coverage for aDNA individuals.
Replication	We replicated estimates for admixture patterns by running ADMIXTURE analyses for each K-group by using 10 independent runs, with a random seed for each K-group. Results were not externally replicated.
Randomization	We used randomization for our simulations.
Blinding	Blinding was not applicable in this study. The study design did not allocate samples to specific groups such as "cases" or "controls".

Reporting for specific materials, systems and methods

We require information from authors about some types of materials, experimental systems and methods used in many studies. Here, indicate whether each material, system or method listed is relevant to your study. If you are not sure if a list item applies to your research, read the appropriate section before selecting a response.

Materials & experimental systems

n/a	Involved in the study
☒	☐ Antibodies
☒	☐ Eukaryotic cell lines
☐	☒ Palaeontology and archaeology
☒	☐ Animals and other organisms
☒	☐ Clinical data
☒	☐ Dual use research of concern
☒	☐ Plants

Methods

n/a	Involved in the study
☒	☐ ChIP-seq
☒	☐ Flow cytometry
☒	☐ MRI-based neuroimaging

Palaeontology and Archaeology

Specimen provenance

The 12 new ancient human remains in this study came from various caves and rock shelters in Zambia and South Africa. We obtained permission from the South African Heritage Resources Agency (SAHRA) to sample and export bones for ancient DNA analyses. Nine WUD samples (permit number: 2789) are from the Raymond A. Dart Archaeological Human Remains Collection (Dart Collection) located at the School of Anatomical Sciences, University of the Witwatersrand (Johannesburg, South Africa). Three UPS samples (permit number: 2804) are from the Archaeological Human remains Collection (Pretoria Bone Collection) situated within the Department of Anatomy, University of Pretoria (Pretoria, South Africa).

For both collections Prof. M. Steyn is the permit holder. The archeological context, morphological assessments, and dating of the remains were described before for six of the samples: WUD034 and WUD037 (C1 and C9 in Meyer et al. 2021; and WUD003, WUD004, WUD008, and WUD010 (Steyn et al 2023). WUD038b sample originated from an archeological site in KwaZulu Natal but is curated in the Dart Collection. WUD012 (Chipongwe Caves) was collected in 1930 by Raymond Dart. WUD012 and WUD018 were originally collected in current-day Zambia and are curated in the Dart Collection, while UPS013, UPS017a, and UPS029 are kept in the Pretoria Bone Collection. Little is known about their archeological contexts.

Specimen deposition

Archeological human remains are housed in the Raymond A. Dart Archaeological Human Remains Collection and the University of Pretoria Bone Collection.

Dating methods

Six samples (WUD038b, WUD012, WUD018, UPS013, UPS017a, and UPS029) were accelerator mass spectrometry (AMS) radiocarbon dated at the Tandem Laboratory (Department of Physics and Astronomy, Uppsala University, Sweden). Radiocarbon dates were calibrated with OxCal 4.4 38 using the atmospheric curve SHCal20 39 and are given at 95.4% probability (2σ).

☐ Tick this box to confirm that the raw and calibrated dates are available in the paper or in Supplementary Information.

Ethics oversight

We obtained all the permissions necessary for ancient DNA analyses from the respective countries.

Note that full information on the approval of the study protocol must also be provided in the manuscript.
